# Axonal sodium channel Na_V_1.2 drives granule cell dendritic GABA release and rapid odor discrimination

**DOI:** 10.1371/journal.pbio.2003816

**Published:** 2018-08-20

**Authors:** Daniel Nunes, Thomas Kuner

**Affiliations:** 1 Champalimaud Research, Champalimaud Centre for the Unknown, Lisbon, Portugal; 2 Functional Neuroanatomy Department, Institute for Anatomy and Cell Biology, Heidelberg University, Heidelberg, Germany; Yale University School of Medicine, United States of America

## Abstract

Dendrodendritic synaptic interactions between olfactory bulb mitral and granule cells represent a key neuronal mechanism of odor discrimination. Dendritic release of gamma-aminobutyric acid (GABA) from granule cells contributes to stimulus-dependent, rapid, and accurate odor discrimination, yet the physiological mechanisms governing this release and its behavioral relevance are unknown. Here, we show that granule cells express the voltage-gated sodium channel α-subunit Na_V_1.2 in clusters distributed throughout the cell surface including dendritic spines. Deletion of Na_V_1.2 in granule cells abolished spiking and GABA release as well as inhibition of synaptically connected mitral cells (MCs). As a consequence, mice required more time to discriminate highly similar odorant mixtures, while odor discrimination learning remained unaffected. In conclusion, we show that expression of Na_V_1.2 in granule cells is crucial for physiological dendritic GABA release and rapid discrimination of similar odorants with high accuracy. Hence, our data indicate that neurotransmitter-releasing dendritic spines function just like axon terminals.

## Author summary

In axonal nerve terminals, neurotransmitter release is triggered by a localized Ca^2+^ nanodomain generated by voltage-gated calcium channels in response to an action potential, which in turn is mediated by voltage-gated sodium channels. Dendritic neurotransmitter release has been thought to work differently, mainly depending on Ca^2+^ entering directly through N-methyl-D-aspartate (NMDA) receptors, a subtype of ligand-gated ion channel. To further investigate how dendritic neurotransmitter is released, we studied granule cells in the olfactory bulb of mice, which establish inhibitory dendrodendritic synapses with mitral cells. We show that granule cells express voltage-gated sodium channels predominantly localized in dendrites and spines. Down-regulation of these channels precludes action potential firing in granule cells and strongly reduces mitral cell inhibition. Behaviorally, these mice require more time to discriminate highly similar odorants at maximal accuracy. Therefore, the inhibition of mitral cells relies on neurotransmitter released from the dendrites of granule cells by a mechanism that resembles axonal neurotransmitter release much more than previously thought.

## Introduction

Inhibitory interactions in the olfactory bulb (OB) play a crucial role for spatiotemporal processing of olfactory information [1–5]. The inhibitory network forming the mammalian OB circuitry [6–9] is dominated by a reciprocal dendrodendritic synapse established between mitral cells (MCs) and granule cells (GCs) [10–15]. This synapse has been shown to define olfactory discrimination time in a stimulus-dependent manner [16]. The structural basis for this behavioral function is represented by two adjacent and inversely oriented synaptic contacts formed between an MC lateral dendrite and a GC spine: an asymmetric contact mediating glutamatergic excitation of the GC and a symmetric contact mediating GABAergic inhibition of the MC [11]. Intense research has addressed the physiological properties of this synapse [17–22], but important aspects of fast and synchronous neurotransmitter release from GC spines and its molecular underpinnings remain poorly understood. A key issue concerns the mechanisms translating the glutamatergic excitatory postsynaptic potential (EPSP) into gamma-aminobutyric acid (GABA) release from the GC spine.

In axonal nerve terminals, fast and synchronous neurotransmitter release is triggered by a highly localized cytoplasmatic Ca^2+^ nanodomain generated by voltage-dependent calcium channels (VDCCs) in response to an action potential invading the presynaptic terminal [23–27]. Mainly, the Ca^2+^ tail current during the repolarization phase of the action potential boosts Ca^2+^ entry and thereby drives neurotransmitter release [25]. This axonal mechanism of neurotransmitter release constitutes a hallmark of synaptic transmission, because it ensures fast and synchronous release [25,26]. Dendritic release is thought to function differently. Previous work at the dendrodendritic synapse suggested that N-methyl-D-aspartate receptor (NMDAR)-mediated Ca^2+^ inflow into the GC spine directly triggers GABA release [17–19,28]. However, such a dendritic release mechanism would require Ca^2+^ to diffuse over a large distance (approximately 1 μm) from the glutamatergic postsynaptic density to the GABAergic active zone [11], implying a large Ca^2+^ domain directly driving neurotransmitter release. Yet such large Ca^2+^ domains are unlikely to exist in GCs, because the high intrinsic buffer capacity of GCs [29] limits Ca^2+^ diffusion, and EGTA does not affect dendrodendritic inhibition [20]. Alternatively, the glutamatergic EPSP may be sufficiently strong to directly activate VDCCs located next to GABAergic vesicles [20]. Specifically, the Na^+^ inward current provided by α-amino-3-hydroxy-5-methyl-4-isoxazolepropionic acid receptors (AMPARs) may either directly trigger a local Ca^2+^ spike [20] or relieve Mg^2+^ block of NMDARs and thereby contribute to the local Ca^2+^ signal causing GABA release [19]. Other conductances, such as transient receptor potential-canonical (TRPC) channels [30] or T-type VDCCs [21], could amplify this process. Nevertheless, none of these mechanisms can provide a sufficiently steep voltage gradient to boost Ca^2+^ entry via the tail current mechanism. Only one report pointed toward a role of voltage-gated sodium channels (VGSCs) in GABA release from GCs [19]: tetrodotoxin (TTX) application decreased dendrodendritic inhibition when applying short depolarizing pulses to MCs, while application of long depolarizing pulses increased it. Because this study relied on applying depolarizing square pulses (3 and 50 ms) that have no similarity with the time course and shape of an action potential, and furthermore used 0 mM Mg^2+^ and TTX in the external solution, these results describe a functional state that cannot be considered physiological. Hence, the contribution of VGSCs to physiological GABA release from GCs remains unknown.

Although dendritic expression of the VGSC Na_V_1.6 α-subunit has been demonstrated in hippocampal neurons [31], the molecular identity of dendritic VGSCs and, in particular, their contribution to dendritic transmitter release remain poorly described. Furthermore, the network and behavioral consequences of dendritic VGSC function remain unknown. To address these issues, we evaluated the expression of VGSCs (reviewed in [32]) in mouse OB GCs and targeted the function of VGSCs in GC dendritic processing. Using 3D-immunohistochemistry [33], we found that GCs exclusively express the Na_V_1.2 α-subunit strongly clustered across the entire GC surface, including the spine heads. We specifically knocked down Na_V_1.2 expression in GCs using viral short hairpin RNA (shRNA) delivery. Na^+^-current and GC spiking were abolished in knockdown GCs. Dendrodendritic inhibition of MCs was strongly attenuated. Discrimination of highly similar odorants required more time for accurate discrimination, yet discrimination of dissimilar odors and odor discrimination learning remained unaffected in Na_V_1.2 knockdown mice. These results establish that VGSCs play a pivotal role in synchronous GABA release under physiological conditions at the dendrodendritic synapse that is required for fast odor discrimination.

## Results

### Expression of VGSCs in the mouse OB

**Fig 1:**
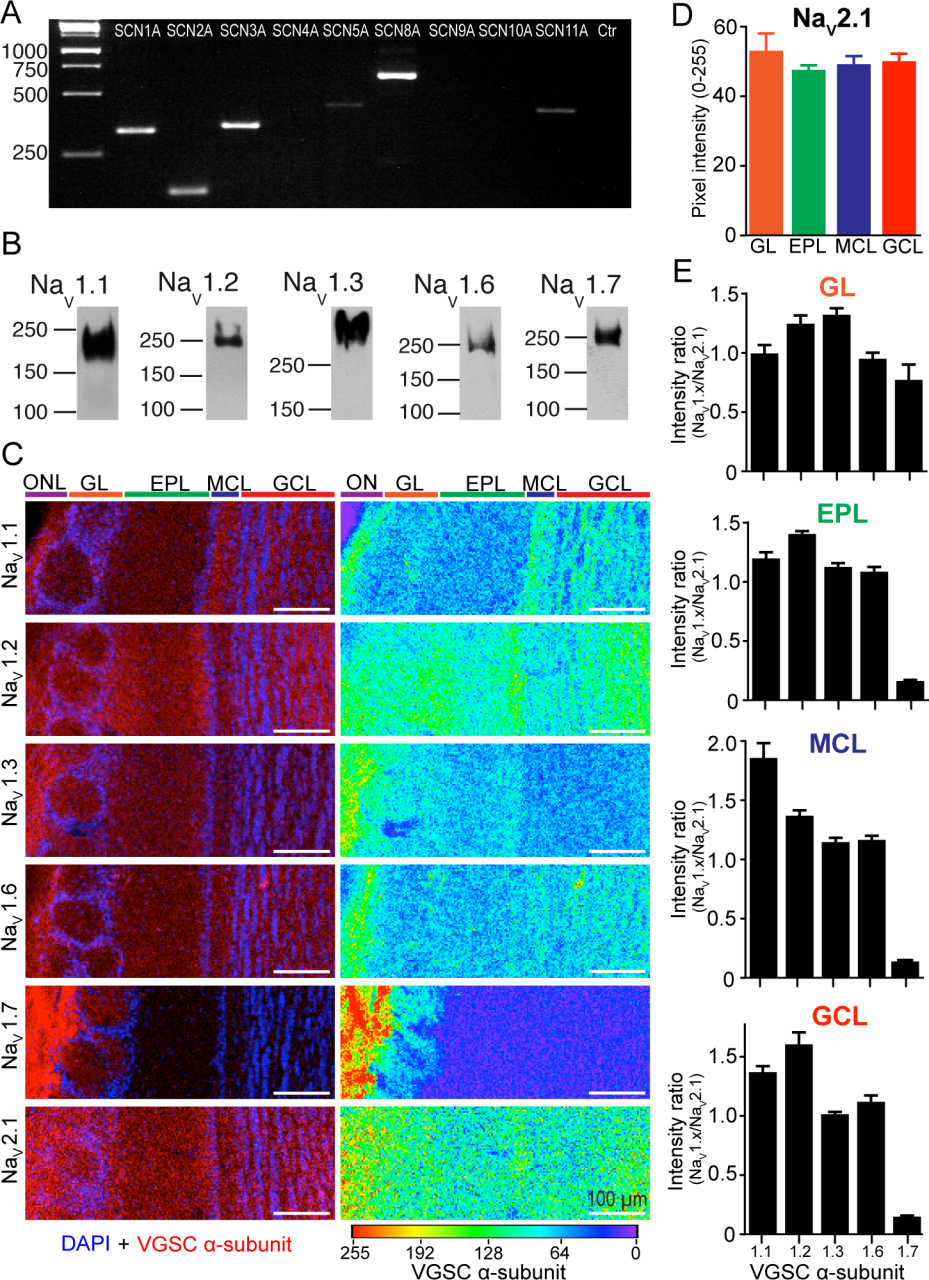
Expression of VGSC in the OB. (A) rT-PCR amplification of VGSC α-subunit cDNAs from whole OB tissue. (B) Western blot of VGSC α-subunit protein expression determined from whole OB tissue. (C) Single-frame confocal images of the distribution of VGSCs α-subtypes Na_V_1.1, Na_V_1.2, Na_V_1.3, Na_V_1.6, Na_V_1.7, and the β-subtype Na_V_2.1 in the OB. VGSCs subtypes (red), nuclei (DAPI, blue). To facilitate comparison of expression levels, grayscale values are represented with the rainbow palette (right column of panels). (D) Average pixel intensity of the Na_V_2.1 subtype across the OB layers. ROIs of 100 × 100 pixels were randomly designed in each layer (*n* = 8), except for the MCL, where the ROI was 20 × 500 pixels (*n* = 5). Expression was similar across layers (GL = 53.13 ± 4.95, EPL = 47.63 ± 1.28, MCL = 49.20 ± 2.40, GCL = 50.13 ± 2.17; ANOVA, *F* = 0.57, *p* = 0.64). (E) Average pixel intensity ratio of VGSCs α-subtypes across OB layers. The intensity ratio was calculated relative to the Na_V_2.1 subtype intensity. Color code: ONL (purple), GL (orange), (green), MCL (blue, includes internal plexiform layer), GCL (red). Data used in the generation of this figure can be found in S1 Data. DAPI, 4,6-diamidino-2-phenylindole; EPL, external plexiform layer; GCL, granule cell layer; GL, glomerular layer; MCL, mitral cell layer; OB, olfactory bulb; ONL, olfactory nerve layer; ROI, region of interest; rT-PCR, reverse transcriptase PCR; VGSC, voltage-gated sodium channel

To clarify the role of VGSCs in GC function, we first analyzed the expression of VGSC α-subunits in the mouse OB (Fig 1). Reverse transcriptase PCR (rT-PCR) showed that the SCN1A, SCN2A, SCN3A, and SCN8A mRNAs were abundant in the OB, while only negligible amounts of SCN5A and SCN11A mRNA could be detected (Fig 1A). Because SCN5A (Na_V_1.5) is expressed in cardiac myocytes [34] and in limbic regions [35], and SCN11A (Na_V_1.9) is expressed only in dorsal root ganglia (DRG) neurons [36], we focused our subsequent protein analysis on a subset of four VGSC α-subunits known to be widely expressed in the brain [32]. Western blot analysis revealed the expression of five α-subunits in the mouse OB (Fig 1B), four of which (Na_V_1.1, Na_V_1.2, Na_V_1.3, and Na_V_1.6) correspond to the genes identified by rT-PCR (SCN1A, SCN2A, SCN3A, and SCN8A, respectively). Additionally, the VGSC α-subunit Na_V_1.7 was prominently expressed in the OB despite the absence of its mRNA (SCN9A). Furthermore, we performed immunohistochemistry using subunit-specific antibodies to assess the distribution of the VGSC α-subunits in the mouse OB (Fig 1C). We used the auxiliary β-subunit Na_V_2.1 to normalize the signal intensity of the VGSC α-subunits, because it is expressed ubiquitously with the α-subunits [32]. Indeed, we have observed that the signal intensity of the β-subunit Na_V_2.1 is homogeneous across the OB layers (Fig 1D; ANOVA, *F* = 0.57, *p* = 0.64). Our region of interest (ROI)-based analysis of the signal intensity of the VGSC α-subunits (Fig 1E) suggests that Na_V_1.2 is most abundantly expressed in the OB, in particular in the granule cell layer (GCL) and in the external plexiform layer (EPL). Na_V_1.1 is strongly expressed in the mitral cell layer (MCL) and olfactory nerve layer (ONL), while Na_V_1.3 mostly occurs in the ONL. Na_V_1.6 is strongly expressed in the ONL and shows a weak and punctate expression in the GCL. The Na_V_1.7 subunit is strongly expressed in the ONL, consistent with its expression in olfactory sensory neurons [37] and the absence of its mRNA in our OB sample (see Fig 1A). The monoclonal antibodies against Na_V_1.1 and Na_V_1.2 target epitopes on the N-terminus of these α-subunits that share a high sequence similarity. We used human embryonic kidney 293 (HEK293) cells transfected with a plasmid expressing the Na_V_1.1 and Na_V_1.2 epitope sequences to confirm the specificity of these antibodies (S1 Fig).

In conclusion, we have identified the distribution of VGSC α-subunits in the mouse OB, while it remains unclear which subunits are expressed in GCs.

### GCs exclusively express the Na_V_1.2 VGSC α-subunit

**Fig 2:**
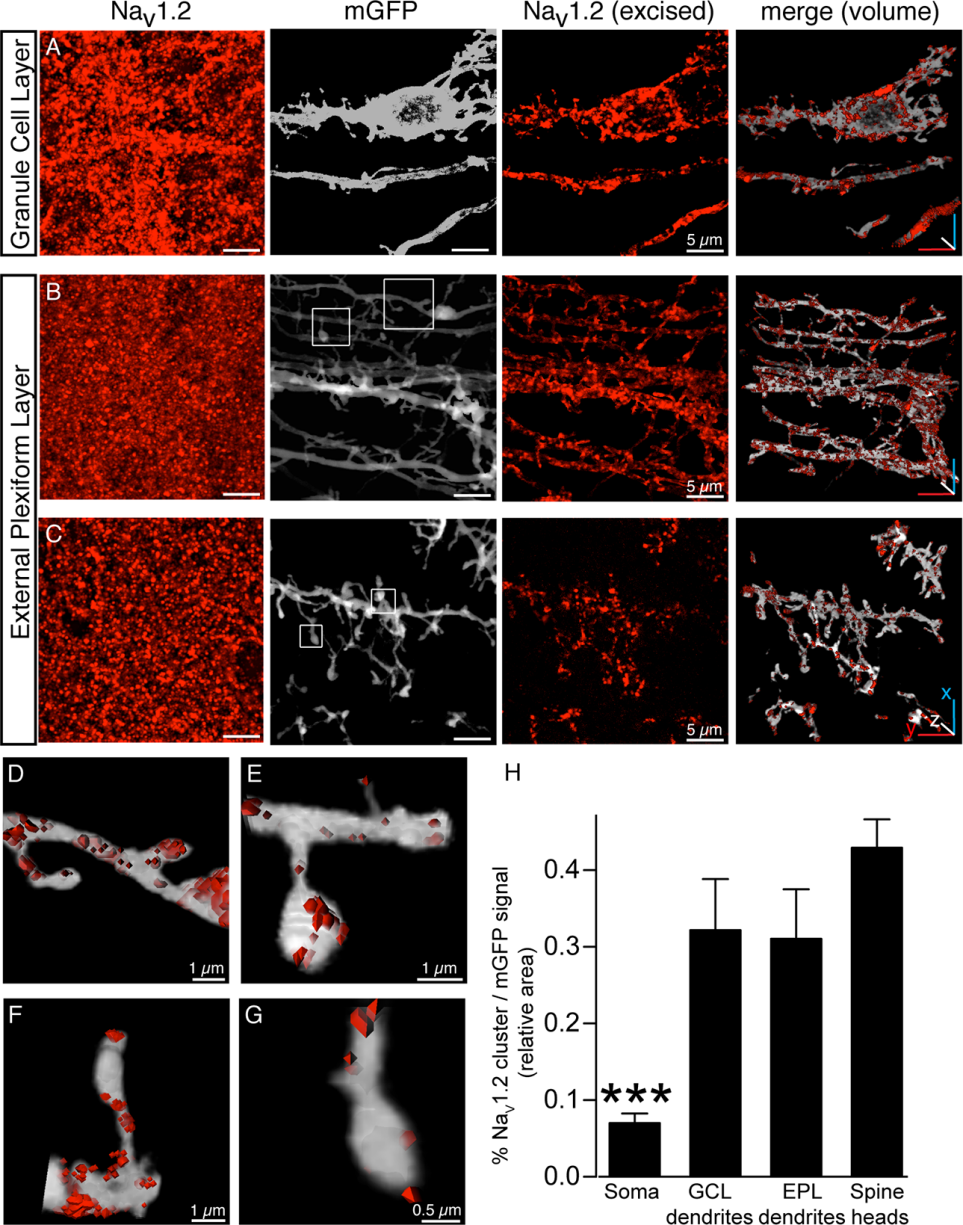
Clustered expression of VGSC subtype Na_V_1.2 in GCs. Detailed view of Na_V_1.2 expression in three layers of the OB. The mGFP signal delineating the GC plasma membrane was used as a 3D template to excise the Na_V_1.2 antibody signals belonging to the GC (see Materials and methods). Images represent maximum-intensity projections of 50–75 serial confocal sections; only merged images are snapshots of volumes rendered in 3D. (A) Region within the GCL containing a cell body and dendritic stem. (B, C) Dendritic shafts and gemmules imaged in the EPL of the OB at a more internal (B) and external (C) position with regard to the EPL. (D-G) Detailed visualization of 3D reconstructions of dendritic shafts and gemmules identified by squares in B and C. Na_V_1.2 clusters are displayed as surface-rendered objects. Image width corresponds to approximately 7 μm. Representative examples were taken from six independent experiments. (H) Quantification of the percentage ratio of the area of mGFP and the area of Na_V_1.2 clusters in soma, GCL dendrites, EPL dendrites, and spine heads. The measurements were taken from 5 independent experiments (*n* = 5 for each condition). The expression of Na_V_1.2 in GC cell bodies is significantly less than in dendrites or spine heads (ANOVA, *F* = 9.101, *p* = 0.0010). Data used in the generation of this figure can be found in S1 Data. EPL, external plexiform layer; GC, granule cell; GCL, granule cell layer; mGFP, membrane-bound green fluorescent protein; OB, olfactory bulb; VGSC, voltage-gated sodium channel.

To identify the VGSC α-subunits expressed in mature GCs, 3D-immunohistochemistry [33] of sparsely prelabeled GCs was performed. To achieve this, the GCL of 3-week-old mice was stereotaxically injected [16] with recombinant adeno-associated virus 2/1 (rAAV2/1) encoding membrane-bound green fluorescent protein (mGFP) [38]. After 3 weeks of expression, tissue containing prelabeled GCs was immunostained with antibodies against the VGSC α-subunit of interest. Only cells with clear GC morphology (soma diameter of <10 μm, dendrite projecting into the EPL [39]) were considered for further analysis. The immunosignal residing inside the mGFP-delimited GC was excised, thereby delineating the expression pattern of α-subunits in individual GCs. Strikingly, only the Na_V_1.2 α-subunit was expressed consistently in GCs at detectable levels (Fig 2 and S2 Fig). The other subunits found to be present in the GCL (Na_V_1.1, Na_V_1.3, and Na_V_1.6) are likely expressed in axons of MCs and cell types other than GCs [40,41]. In some instances, we observed a random partial overlap between Na_V_1.x and mGFP (S2 Fig) owing to technical limitations of 3D-immunohistochemistry [33]. To further corroborate our finding, we used phrixotoxin-3, a selective antagonist of Na_V_1.2 at low nanomolar concentrations [42], to block Na^+^ currents in GCs (S3A–S3C Fig). This experiment showed a 75% ± 10% (*n* = 4) reduction of the Na+ current at −30 mV and further reduction to 96% ± 0.8% after adding 1 μM TTX. The incomplete block by phrixotoxin-3 was due to the low concentration that had to be used to selectively block Na_V_1.2 (1 nM, IC_50_ is 0.6 nM [42]). In contrast to GCs, phrixotoxin-3 caused only a minor reduction of Na^+^ currents elicited in MCs (S3D–S3F Fig). Hence, phrixotoxin-3 block of the Na^+^ current is consistent with the expression of Na_V_1.2 reported by immunohistochemistry.

Na_V_1.2 was found in a strongly clustered arrangement in the cell body (Fig 2A), dendritic shaft (Fig 2B), and in the gemmules (Fig 2C) of the GCs. Close-up 3D reconstructions of the gemmules revealed clusters of Na_V_1.2 immunoreactivity within the spines (Fig 2D and 2E). To quantify the distribution of Na_V_1.2 clusters in GCs, we determined the ratio of the Na_V_1.2 cluster area relative to the area delineated by mGFP expression (Fig 2H). This analysis revealed the highest density of Na_V_1.2 clusters within the spine heads and dendrites. At GC somas, the cluster density was significantly lower (soma: 7% ± 1.3%; GCL dendrites: 32.2% ± 6.7%; EPL dendrites: 31.1% ± 6.5%; 43% ± 3.7% ANOVA, *F* = 9.101, *p* = 0.001). This distribution pattern predicts a dominant function of Na_V_1.2 channels in the dendrites and particularly within gemmules of GCs. While Na_V_1.2 has been described as an axonal subunit [32], this expression pattern is remarkable for axonless GCs, suggesting that the Na_V_1.2 α-subunit might trigger synchronous GABA release from GC spine heads.

### Knockdown of the Na_V_1.2 subunit abolishes action potential firing in GCs

**Fig 3:**
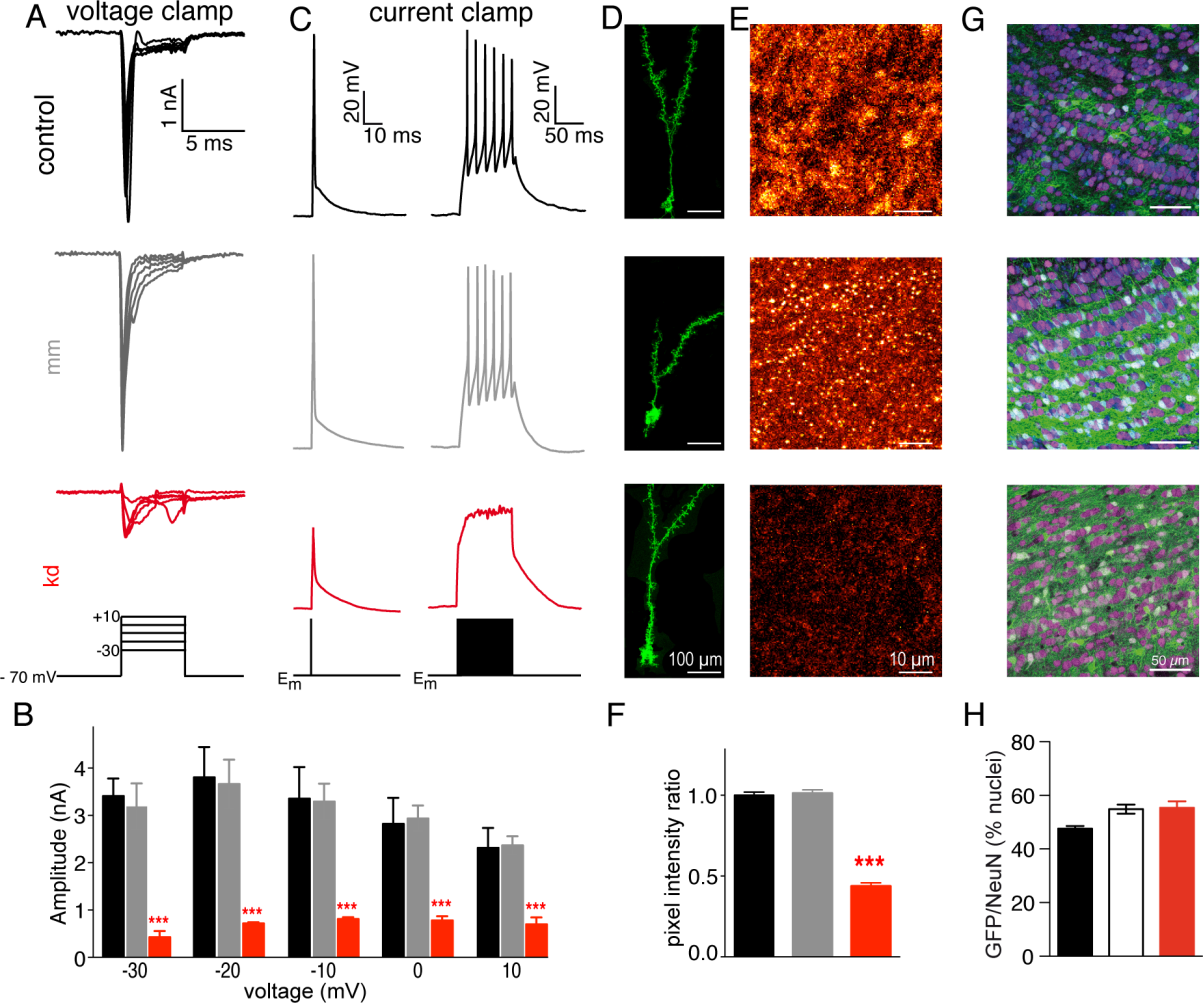
shRNA#14 strongly reduces Na^+^-currents and abolishes action potential firing in GCs. (A) Representative traces of whole-cell voltage-clamp recordings of control cells (black), rAAV-mmshRNA#14-infected (gray), and rAAV-shRNA#14-infected (red) GCs. All recordings were done at 34 ± 1 °C. Voltage-clamp recordings were made in bath solution containing 10 mM TEA. The voltage traces depict voltage steps from −30 mV to +10 mV. (B) Quantification of the Na^+^ currents (color code as in A). No differences were observed between control cells (*n* = 6) and rAAV-mmshRNA#14-infected cells (*n* = 6; ANOVA, *F* = 64.59, *p* < 0.001), but Na^+^ currents in GCs infected with rAAV-shRNA#14 (*n* = 6) were strongly reduced (ANOVA, *F* = 64.59, *p* < 0.01). (C) Whole-cell current-clamp recordings (color code as in A). Single action potential firing was assessed by 1-ms square pulses of somatic current injections (800 pA for examples shown). Multispiking was stimulated by current injection lasting 100 ms. (D) Morphological appearance of the GCs upon expression of mGFP (control, upper panel) or mGFP with shRNAmm (middle panel) or shRNA#14 (lower panel). The images represent maximum-intensity projections of a confocal stack extending over 80 to 120 image frames. Grayscale levels adjusted for display. (E) Na_V_1.2 expression in the OB. Maximum-intensity projections of confocal stacks extending over 20 consecutive image frames of stainings against Na_V_1.2 in control (upper panel), shRNAmm (middle panel), and shRNA#14 (lower panel). Grayscale levels adjusted for display. (F) Quantification of the intensity ratio relative to control levels of the Na_V_1.2 subtype in control, shRNAmm, and shRNA#14. MIPs of 37.53 μm × 37.53 μm (100 × 100 pixels), as in (E), were used (*n* = 12 per condition), obtained from two independent experiments. The intensity ratio in the shRNA#14 condition was reduced in comparison to the control and shRNAmm conditions (ANOVA, *F* = 265.2, *p* < 0.001). (G) rAAV-mediated transduction efficiency of the GCL. Representative confocal image stacks (MIP of 10 image frames) of the GCL, from OB horizontal sections, of mice stereotaxically injected with rAAV-eGFP (“control,” *n* = 4 mice), rAAV-mmshRNA#14 (“mm,” *n* = 6 mice), or rAAV-shRNA#14 (“kd,” *n* = 6 mice). DAPI-stained nuclei (blue), anti-NeuN antibody labels neuronal nuclei (magenta), and GFP reveal transduced GCs (green). (H) Efficiency of neuronal transduction by rAAV: control: 47.57% ± 0.93%, mm: 54.92% ± 1.73%, kd: 55.45% ± 2.35%; ANOVA, *F* = 2.69, *p* = 0.12. Data used in the generation of this figure can be found in S1 Data. DAPI, 4,6-diamidino-2-phenylindole; GC, granule cell; GCL, granule cell layer; GFP, green fluorescent protein; mGFP, membrane-bound GFP; MIP, maximum intensity projection; shRNAmm, rAAV, recombinant adeno-associated virus; shRNA, short hairpin RNA; TEA, tetraethylammonium

To abolish Na_V_1.2-dependent Na^+^ currents in GCs, we designed shRNA molecules to specifically target SCN2A (see Materials and methods, S4 Fig). Four different shRNAs bicistronically expressing enhanced green fluorescent protein (eGFP) were cloned in rAAV2/1 vectors and stereotaxically delivered to the GCL. Upon 5 weeks of expression, OB acute slices were prepared to assess Na^+^ currents in eGFP-positive GCs expressing the respective shRNA (S4 Fig). shRNA#14 showed the most potent reduction of the Na^+^ current in GCs (90% ± 3%, *n* = 8) and was therefore chosen for all subsequent experiments. The specificity of shRNA#14 for Na_V_1.2 was further examined in silico (S5 Fig). The Na^+^ current was reliably reduced in comparison to control cells and cells infected with a mismatch shRNA (shRNAmm) (Fig 3A and 3B), suggesting a specific knockdown of the Na_V_1.2 α-subunit in the mouse OB.

The observed reduction of the Na^+^ current amplitude after knockdown abrogated action potential firing in GCs upon somatic current injections (Fig 3C). GCs expressing shRNA#14 failed to fire action potentials, while control cells or cells expressing the shRNAmm fired a single action potential upon 3-ms current injections (Fig 3C, left set of traces) or fired tonically upon longer current injections (Fig 3C, right set of traces). These experiments demonstrate that a selective knockdown of the Na_V_1.2 α-subunit abolishes action potential firing in GCs.

To further corroborate shRNA-mediated Na_V_1.2 knockdown, we examined the morphology of transduced GCs and quantified Na_V_1.2 expression. The general morphological features of GCs were unchanged upon shRNA expression (Fig 3D), and we observed a reduction of the Na_V_1.2 staining (Fig 3E) upon viral transduction of the GCL with rAAVs carrying shRNA#14, in comparison to control infection (mGFP) and shRNAmm14 (Fig 3F). For a quantitative comparison, we calculated fluorescence ratios relative to the mean fluorescence of the control (control: 1.00 ± 0.019, shRNAmm: 1.01 ± 0.02, shRNA#14: 0.44 ± 0.02, ANOVA, *F* = 265.2, *p* < 0.001, see Materials and methods). While we observed no intensity ratio differences between the control and the shRNAmm (*t* = 0.42, *p* > 0.05), the intensity ratio was strongly reduced when comparing shRNA#14 expression with control (*t* = 19.73, *p* < 0.001) and shRNAmm (*t* = 20.16; *p* < 0.001). In summary, we demonstrated the effectiveness of shRNA#14 in reducing Na_V_1.2 subtype expression in GCs, leading to a strong reduction of Na^+^ currents and abrogating action potential firing in GCs.

Before analyzing the physiological and behavioral consequences of Na_V_1.2 knockdown in GCs, we investigated the spread of rAAV1/2 infection for widespread expression of shRNA#14 within the OB (S6A Fig). We observed that virtually all green fluorescent protein (GFP)-positive cells were confined to the GCL, indicating that the stereotaxic delivery of rAAV2/1 particles into the GCL spares the MCL, EPL, and glomerular layer (GL), as previously demonstrated [16]. Furthermore, rAAV transduction did not spread beyond the OB, thereby constituting a highly local genetic perturbation, a prerequisite to causally link molecular function to odor discrimination behavior (S6B Fig) [16],[38]. While this approach is not inherently GC specific, more than 99% of the cells expressing GFP were GCs as judged by the diameter of the soma and location of the dendrite (S6C–S6E Fig) [39,40]. Expression of the shRNA#14 (kd) and the shRNAmm control were tracked by coexpression of eGFP and compared to eGFP expression alone (control). Additionally, to quantify the number of infected cells in the GCL, the neuronal marker NeuN was used to identify the nuclei of neurons populating the OB, while 4,6-diamidino-2-phenylindole (DAPI) was used to label nuclei of all cell types in the OB sections (Fig 3G; S6F Fig). The number of DAPI-positive nuclei did not vary among the conditions tested (control: 529.7 ± 22.07, *n* = 4; mm: 486.5 ± 34.40, *n* = 6; kd: 520.0 ± 30.82, *n* = 6; ANOVA, *p* = 0.58), indicating that neither cell death nor glial proliferation occurred (S6G Fig). This is consistent with the unaltered ratio of NeuN/DAPI cells in the GCL (S6H Fig; control: 73.92% ± 2.25%; mm: 80.43% ± 2.20%; kd: 76.16% ± 3.84%; ANOVA, *F* = 1.33, *p* = 0.31). To assess the number of neurons infected in the GCL, we counted the nuclei that were positive for both NeuN and eGFP (Fig 3H). The fraction of infected neurons was not significantly different (control: 47.57% ± 0.93%; mm: 54.92% ± 1.73%; kd: 55.45% ± 2.35%; ANOVA, *p* = 0.12), suggesting that each viral preparation transduced approximately 50% of the GCs (Fig 3H). While the GCL is dominated by GCs, other types of neurons are present as well, although at much lower numbers [40,41]. Because viral infection might also transduce a small number of these neurons (S6D and S6E Fig), we refer to mice infected with rAAVs carrying the shRNA#14 as Na_V_1.2^ΔGCL^ mice and mice infected with rAAVs carrying the shRNAmm as mm^ΔGCL^. Herewith, we show that our acute genetic perturbation approach is effective in selectively infecting the GCL, leaving other OB cell layers unaffected.

### Dendrodendritic inhibition of MCs requires VGSC activation in GCs

**Fig 4:**
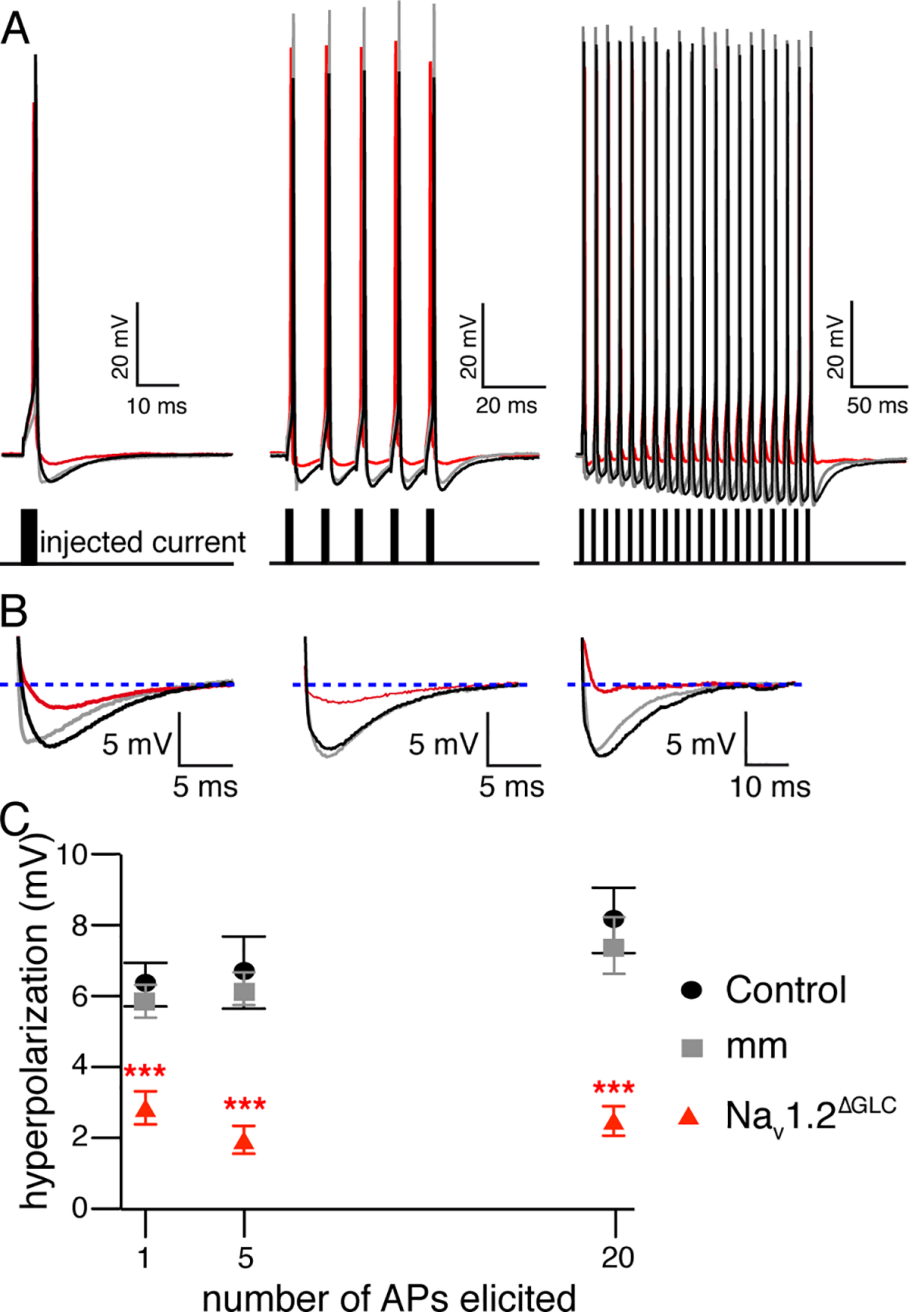
VGSCs are essential for GABA release from GCs and inhibition of MCs. (A) Representative traces of a single AP and trains of 5 and 20 APs at 100 Hz elicited through somatic current injections in MCs. Control (black), mismatch (gray), and knockdown (red). (B) Hyperpolarization elicited by the three stimulation protocols in each condition (color code as in A). The horizontal stippled line (blue) denotes the resting membrane potential. (C) Quantification of the hyperpolarization amplitude. Overall, a significant difference of the hyperpolarization amplitude was observed (statistics described in Results text). Data used in the generation of this figure can be found in S1 Data. AP, action potential; GABA, gamma-aminobutyric acid; GC, granule cell; MC, mitral cell; VGSC, voltage-gated sodium channel.

To test if a lack of action potential firing in GCs would affect dendrodendritic inhibition of MCs by GCs, we performed whole-cell current clamp recordings from MCs in acute OB slices at near physiological temperature (34 ± 1 °C). Somatic current injections in MCs reliably generated action potentials (Table 1, Fig 4A and 4B). In the control and shRNA conditions, similar amounts of current were required to trigger action potentials with indistinguishable properties (Table 1, ANOVA, *F* = 0.80, *p* = 0.45). The action potential was followed by a rapid and pronounced hyperpolarization that increased with the number and frequency of action potentials elicited (Fig 4A and 4B). To further define the nature of this hyperpolarization, we blocked dendrodendritic transmission with ionotropic glutamate receptor (6-cyano-7- nitroquinoxaline-2,3-dione [CNQX] and 2-amino-5-phosphonopentanoic acid [APV]) or GABA_A_ receptor (gabazine) antagonists (S7 Fig). In both cases, a remaining hyperpolarization of 8%–11% was observed that could be attributed to MC-intrinsic conductances such as the afterhyperpolarization (S8 Fig). Therefore, we conclude that our recording conditions give rise to a fast hyperpolarization that predominantly reflects the synchronous recurrent inhibitory postsynaptic potential (rIPSP).

**Table 1:** Electrophysiological properties of MCs and amplitudes of injected currents.

	AP peak (mV)	Resting potential (mV)	R_seal_ (MΩ)	Injected current 1 AP (pA)	Injected current 5 AP (pA)	Injected current 20 AP (pA)	*n*
**control**	36.86 ± 1.90	−53.96 ± 0.63	56.77 ± 4.73	490.0 ± 60.46	620.0 ± 48.99	790.0 ± 48.19	10
**mm^ΔGCL^**	35.18 ± 2.16	−53.95 ± 0.60	56.61 ± 5.42	433.3 ± 64.35	616.7 ± 73.68	675.0 ± 65.80	12
**Na_V_1.2^ΔGCL^**	35.51 ± 2.61	−53.50 ± 0.64	50.88 ± 5.21	484.6 ± 45.07	584.6 ± 62.89	669.2 ± 58.16	13
***F* value**	0.14	0.47	0.47	0.31	0.09	1.23	
***p*-value**	0.87	0.71	0.71	0.74	0.91	0.31	

Abbreviation: AP, action potential; MC, mitral cell.

In control (*n* = 10) and mm^ΔGCL^ cells (*n* = 12), action potential firing produced hyperpolarizations (Fig 4B and 4C) with indistinguishable amplitudes (control: 6.35 ± 0.62 mV; mm^ΔGCL^: 5.816 ± 0.47 mV; *t* = 0.71, *p* > 0.05). However, in Na_V_1.2^ΔGCL^ cells, the hyperpolarization amplitudes were strongly decreased (2.87 ± 0.46 mV [*n* = 13]; control versus Na_V_1.2^ΔGCL^ *t* = 4.73, mm^ΔGCL^ versus Na_V_1.2^ΔGCL^ *t* = 4.21, *p* < 0.001 in both conditions). Moreover, we observed the same effect for trains of 5 and 20 action potentials (Fig 4C; 5 action potentials—control: 6.68 ± 1.01 mV, mm^ΔGCL^: 6.18 ± 0.47 mV, Na_V_1.2^ΔGCL^: 1.97 ± 0.39 mV; control versus mm^ΔGCL^ *t* = 0.56 *p* > 0.05, control versus Na_V_1.2^ΔGCL^ *t* = 5.28 *p* < 0.001, mm^ΔGCL^ versus Na_V_1.2^ΔGCL^ *t* = 4.95 *p* < 0.001; 20 action potentials—control: 8.15 ± 0.92 mV, mm^ΔGCL^: 7.41 ± 0.81 mV, Na_V_1.2^ΔGCL^: 2.50 ± 0.41 mV; control versus mm^ΔGCL^ *t* = 0.74 *p* > 0.05, control versus Na_V_1.2^ΔGCL^ *t* = 5.53 *p* < 0.001, mm^ΔGCL^ versus Na_V_1.2^ΔGCL^ *t* = 5.04 *p* < 0.001). Hence, given that 90% of the measured hyperpolarization is contributed by the rIPSP (see above), knockdown of Na_V_1.2 in GCs highly significantly reduces dendrodendritic inhibition. While the action of ionotropic glutamate receptor blockers and GABA_A_ receptors appears stronger than the effect of Na_V_1.2 knockdown (S8 Fig), this is expected because Na_V_1.2 is only knocked down in approximately 55% of the GCs (see Fig 3H and Discussion).

To simulate odor-induced spike trains [4,43], we stimulated MCs for different durations, with trains of action potentials at 100 Hz. As described above, in control cells, the hyperpolarization amplitude increased with the number of action potentials triggered in the MC (Fig 4A and 4C), suggesting that brief bursts of action potentials facilitated GABA release from GCs. Upon Na_V_1.2 α-subunit knockdown in the GCL, the frequency-dependent facilitation of the hyperpolarization amplitude was abolished (Fig 4C). Furthermore, action potentials in MCs remained unaffected (Table 1), supporting the notion that rAAV infection is restricted to the GCL. In conclusion, these results demonstrate that Na_V_1.2 VGSC α-subunits are required for synchronous release of GABA to produce dendrodendritic inhibition of MCs.

### Reduced inhibition of MCs results in a stimulus-dependent slowing of odor discrimination time

**Fig 5:**
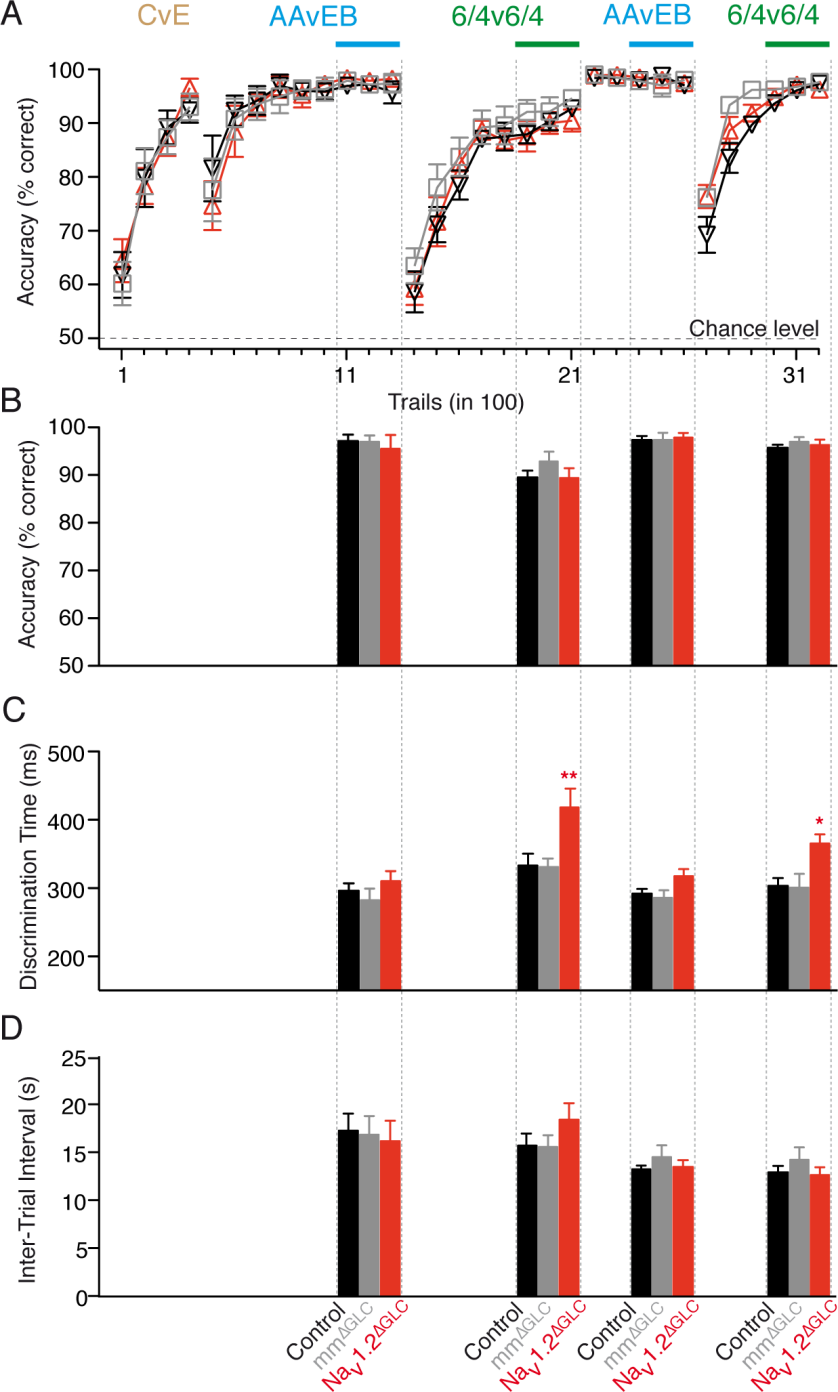
Disinhibition of mitral cells slows odor discrimination of highly similar odors. (A) Learning curves (mean ± SEM). Control (black, *n* = 8), mm^ΔGCL^ (gray, *n* = 8), and Na_V_1.2^ΔGCL^ (red, *n* = 8). Odor pairs: CvE, AAvEB. No differences in learning were observed. (B) Odor discrimination accuracy (mean ± SEM) was measured after animals learned to discriminate the test odors (as indicated in A: blue bars [AAvEB], green bars [6/4v6/4]). No significant differences were found between the groups (first round—AAvEB *F* = 0.33, *p* = 0.72; 6v4/4v6 *F* = 1.12, *p* = 0.34; second round—AAvEB *F* = 0.05, *p* = 0.95; 6v4/4v6 *F* = 0.41, *p* = 0.67). (C) Discrimination time (mean ± SEM) determined after mice achieved highly accurate discrimination (during the trials marked with horizontal bars in A). No significant differences were observed for the discrimination time of monomolecular odors. Mixture discrimination time was significantly different in knockdown mice in both repetitions of the test (ANOVA, *F* = 6.23, *p* = 0.008, and *F* = 5.58, *p* = 0.01, respectively). In the first round, Na_V_1.2^ΔGCL^ mice (418.2 ± 27.65 ms, *n* = 8) were slower in discriminating highly similar odors than control (333.4 ± 16.86 ms, *n* = 8; *t* = 3.12, *p* < 0.05) and mm^ΔGCL^ (330.3 ± 12.16 ms, *n* = 8; *t* = 3.00, *p* < 0.05) mice, was well as in the second round (Na_V_1.2^ΔGCL^: 364.5 ± 13.29 ms, *n* = 8; control: 301.9 ± 11.33 ms, *n* = 8, *t* = 2.95, *p* < 0.05; mm^ΔGCL^: 299.4 ± 20.64 ms, *n* = 8, *t* = 2.84, *p* < 0.05). (D) Intertrial interval (mean ± SEM) as an indicator of motivation. None of the groups showed a significant difference as assessed by ANOVA (first round AAvEB *F* = 0.12 *p* = 0.89, second round AAvEB *F* = 0.62 *p* = 0.55; first round 6/4v6/4 *F* = 1.23 *p* = 0.31, second round 6/4v6/4 *F* = 0.77 *p* = 0.48). *t*-values were derived from the Bonferroni multiple comparisons test. Data used in the generation of this figure can be found in S1 Data. AAvEB, amyl acetate versus ethyl butyrate; CvE, cineol versus eugenol.

The go/no-go operant conditioning paradigm [16,44] was used to address the role of GC Na_V_1.2 subunits in odor discrimination behavior (Fig 5). Three groups of mice—wild-type controls, mm^ΔGCL^ controls, and Na_V_1.2^ΔGCL^ knockdown mice—were pretrained to discriminate the odorants cineol and eugenol (cineol versus eugenol [CvE]) for task habituation. Subsequently, the test odorants amyl acetate and ethyl butyrate (amyl acetate versus ethyl butyrate [AAvEB]) and their binary 6:4 mixtures (6v4/4v6) were tested. The three groups of mice learned equally well to discriminate the tested odorants with accuracies exceeding 90% (Fig 5A and 5B). For the simple odors, mice had similar discrimination times in both rounds of testing (Fig 5C; first round AAvEB—control: 295.2 ± 10.81 ms, mm^ΔGCL^: 281.50 ± 16.78 ms, Na_V_1.2^ΔGCL^: 310.60 ± 14.55 ms, ANOVA, *F* = 1.04, *p* = 0.37; second round AAvEB—control: 291.7 ± 7.032 ms, mm^ΔGCL^: 284.5 ± 10.07 ms, Na_V_1.2^ΔGCL^: 316.10 ± 10.07 ms, ANOVA, *F* = 3.11, *p* = 0.07). However, Na_V_1.2^ΔGCL^ mice showed a significant increase of the discrimination time for highly similar odorants (Fig 5C), in comparison to control and mm^ΔGCL^ mice, in both rounds of testing (first round 6/4v6/4—control: 333.40 ± 16.86 ms, mm^ΔGCL^: 330 ± 12.16 ms, Na_V_1.2^ΔGCL^: 418.20 ± 27.65 ms, ANOVA, *F* = 6.23, *p* = 0.008; control versus mm^ΔGCL^ *t* = 0.11 *p* > 0.05; control versus Na_V_1.2^ΔGCL^ *t* = 3.00 *p* < 0.05; mm^ΔGCL^ versus Na_V_1.2^ΔGCL^ *t* = 3.11 *p* < 0.005; second round 6/4v6/4—control: 301.9 ± 11.33 ms, mm^ΔGCL^: 299.40 ± 20.64 ms, Na_V_1.2^ΔGCL^: 364.50 ± 20.64 ms, ANOVA, *F* = 5.58, *p* = 0.01; control versus mm^ΔGCL^ *t* = 0.11 *p* > 0.05; control versus Na_V_1.2^ΔGCL^ *t* = 2.84 *p* < 0.05; mm^ΔGCL^ versus Na_V_1.2^ΔGCL^ *t* = 2.95 *p* < 0.05). The intertrial interval, taken as a motivational indicator, was unchanged, suggesting constant motivational conditions (Fig 5D). Our behavioral data demonstrate that lack of MC inhibition caused by Na_V_1.2 knockdown in GCs leads to a stimulus-dependent prolongation of odor discrimination time, but it does not change discrimination accuracy or learning.

## Discussion

In this study, we show that GCs express clusters of Na_V_1.2 subunits on their dendritic surface including spine heads. These VGSCs are required for synchronous GABA release, MC inhibition, and rapid odor discrimination. On a more general perspective, our results suggest that dendritic neurotransmitter release operates mechanisms of fast and synchronous release known for axon terminals.

### Dendritic VGSCs

Our expression analysis shows that GCs exclusively express the sodium channel α-subunit Na_V_1.2, a subunit predominantly expressed in axons [32]. At first glance, it may sound surprising that an axonless neuron expresses an axonal VGSC subunit in its dendrites. However, given that GCs use dendritic spines both to receive synaptic input and to generate output, the presence of the Na_V_1.2 subunit may imply that neurotransmitter release from spines utilizes the same mechanisms established in axon terminals. Another VGSC subunit, Na_V_1.6, has been found in proximal and distal dendrites of hippocampal CA1 neurons [31] supporting a role in mediating backpropagating action potentials [45]. Lorincz and colleagues found Na_V_1.2 only within axons and presynaptic terminals but not in the dendritic domain. Hence, while dendritic expression of certain VGSC subunits appears to be a general phenomenon, we postulate that Na_V_1.2 occurs only in dendrites capable of neurotransmitter release.

### Functional predictions derived from the clustered expression pattern of Na_V_1.2 in GCs

Na_V_1.2 is expressed in small clusters over the entire extent of a GC, including the heads of GC spines (e.g., Fig 2E). Cluster formation of Na_V_1.2 may be required to achieve a sufficiently large local current density for sodium channel activation and action potential initiation, similar to the situation found at nodes of Ranvier or the axon initial segment. VGSC clusters in the dendritic shaft may propagate the action potential along the dendritic tree of the GC, thereby generating a global action potential. Clusters situated in the GC spines may mediate a locally restricted action potential, as discussed in a separate section below.

### Mechanisms of GABA release from GC spines

**Fig 6:**
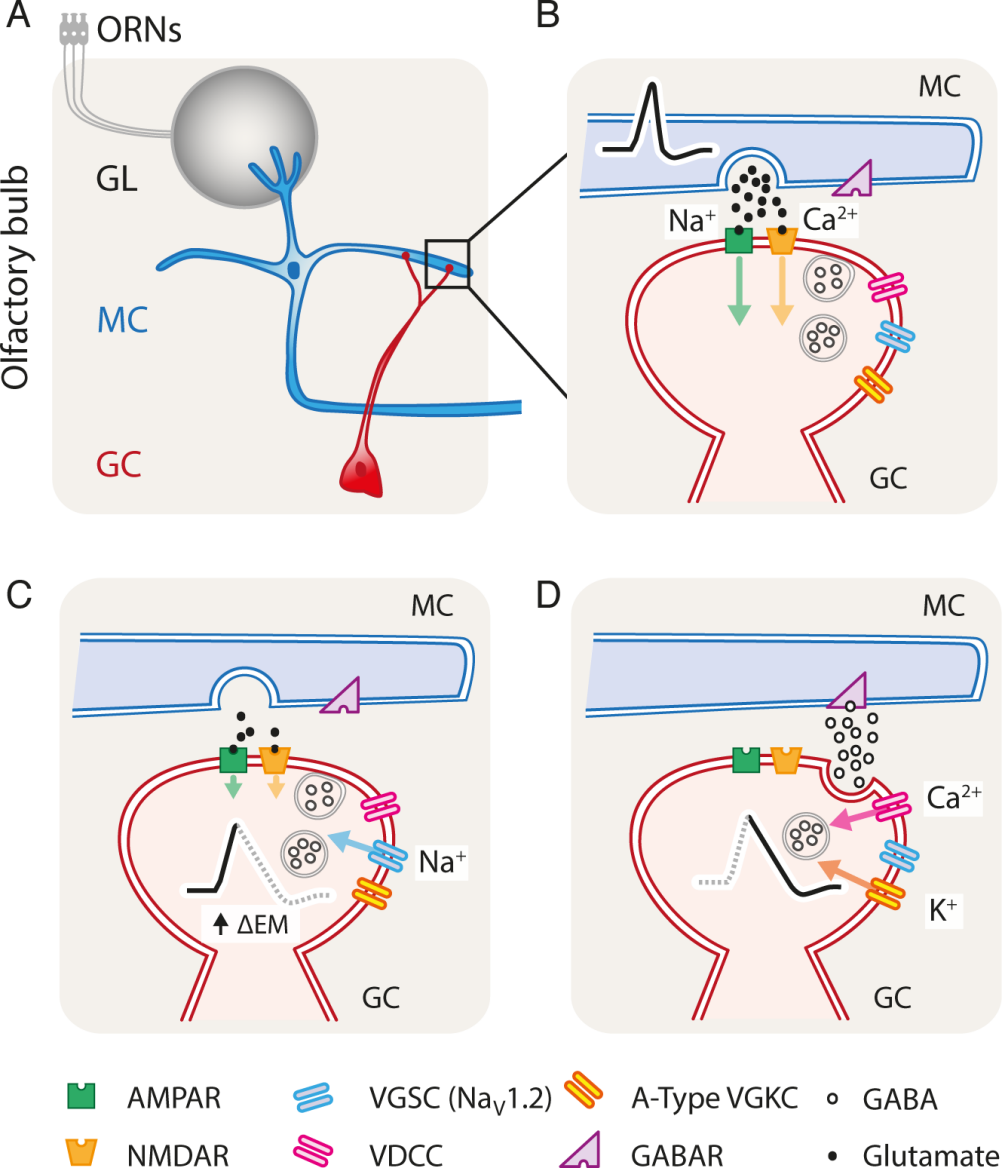
Model of dendrodendritic inhibition. (A) MCs receive inputs from the ORNs, activating the recurrent inhibitory network. (B) Action potential propagation in the MC lateral dendrite leads to glutamate release from MCs and consequent activation of AMPAR and NMDAR in GC spines, leading to sodium and calcium influx, respectively. (C) The depolarization of the GC spine activates the VGSC Na_V_1.2 subtype, which leads to an all-or-none event, as known for release in axon terminals. (D) Activation of the VGSCs activates VDCCs that in turn generate a Ca^2+^ nanodomain during the repolarization phase of the action potential, when the driving force for Ca^2+^ is particularly large. Finally, the nanodomain of Ca^2+^ (not shown for simplicity) triggers GABA release in a fast and synchronous fashion. A-type VDKCs produce a prolonged potassium influx that contributes to prolonged depolarization of the CG spine. AMPAR, α-amino-3-hydroxy-5-methyl-4-isoxazolepropionic acid receptor; GABAR, gamma-aminobutyric acid receptor; GC, granule cell; GL, glomerular layer; MC, mitral cell; NMDAR, N-methyl-D-aspartate receptor; ORN, olfactory receptor neuron; VDCC, voltage-dependent calcium channel; VDKC, voltage-dependent potassium channel; VGKC, voltage-gated potassium channel; VGSC, voltage-gated sodium channel.

We suggest that under physiological conditions, the glutamatergic EPSP (Fig 6A and 6B) leads to a local depolarization that reaches the threshold of Na_V_1.2 activation (Fig 6C), which then drives the initiation of an action potential in the spine. Subsequently, activated VDCCs, presumed to be localized in the GABAergic active zone, produce a nanodomain with high Ca^2+^ concentration during the repolarization phase of the action potential (Fig 6D), when the driving force for Ca^2+^ is high, similar to the mechanisms known to exist in axon terminals [25,26,46]. In GC spines, this mechanism allows for fast and synchronous release of GABA and temporally well-resolved inhibition of MCs (Fig 4).

Knockdown of Na_V_1.2 in GCs activity-dependently reduced the hyperpolarization amplitude in MCs by 60%–75% (Fig 4B, S8 Fig). Considering that 10% of the control hyperpolarization is mediated by the afterhyperpolarization or other intrinsic MC conductances such as I_h_, an rIPSP component of approximately 15%–30% remained (see S8 Fig). However, if GABA release depends on a local all-or-none spine action potential, expression of the shRNA should result in a complete block of the rIPSP. This apparent discrepancy may be due to the incomplete transduction of GCs connected to the stimulated MC with rAAVs expressing shRNA and the incomplete knockdown of Na_V_1.2 (Fig 3). Thus, a mixed population of unperturbed GCs and GCs with Na_V_1.2 knockdown defines the strength of recurrent inhibition found in MC recordings presented here. Alternatively, after Na_V_1.2 knockdown, T-type Ca^2+^ channels may contribute to GABA release from GC spines and thereby generate some of the remaining MC hyperpolarization. The contribution of VGSC α-subunits other than Na_V_1.2 appears unlikely, given that none of these could be detected in GCs by immunohistochemistry (S2 Fig). Hence, the strong reduction of the rIPSP amplitude indicates that an all-or-none action potential is required for fast and synchronous GABA release from GC spines.

While we demonstrated a clustered distribution of Na_V_1.2 within GCs, with higher densities in the spine heads and dendrites compared to the soma, the Na_V_1.2 knockdown is likely to affect clusters in all locations of the GC. Hence, despite the highest density of clusters occurring in the spine heads, it remains difficult to know at which site action potentials get initiated: spine head or dendrite. Assuming the former raises the question whether this action potential remains a local event or propagates into the GC dendrite, thereby causing a global GC action potential. Recent work suggested that the high impedance of GC spine necks may insulate GC spines to function as “mini-neurons” or single processing units [47]. Such isolated autonomous compartments would mainly support local recurrent MC inhibition and may employ local plasticity mechanisms but would not allow dendritic integration of multiple glutamatergic inputs from different MCs. Yet the spine neck has little effect on EPSP propagation in the forward direction [48]. Such an impedance mismatch circuit allows coincidence detection within the dendrite and, in combination with dendritic inhibition, controls action potential generation in the dendritic tree. Hence, the design of the GC dendrite provides a solution to both ensure efficient local neurotransmitter release, plasticity, and dendritic computations of multiple synchronously active MC inputs.

### Role of NMDARs at the dendrodendritic synapse?

This Na_V_1.2-dependent model of reciprocal synaptic communication seemingly contradicts previous work claiming that dendrodendritic GABA release is governed by NMDAR-mediated currents that activate P/Q-type VDCCs [17,28] or that Ca^2+^ entry by NMDARs suffices to drive GABA release [18,19], such that VGSCs are not required for GABA release at the dendrodendritic synapse [17]. These studies used Mg^2+^-free solutions containing TTX combined with very long MC current injections. Such unphysiological conditions will yield an unnaturally strong release of glutamate from MC dendrites, followed by a sustained Ca^2+^ inflow through NMDARs into the GC spine heads that will suffice to trigger slow, asynchronous GABA release. In contrast, synchronous GABA release physiologically triggered by MC action potentials occurs on the time scale of a few milliseconds (see Fig 4B). Thus, a comparison of the studies mentioned above with our results needs to consider that two different modes of transmitter release are affected—asynchronous and synchronous release, respectively—each operating on different release sensors [49].

Electron microscopy has revealed a segregated organization of the postsynaptic density and the active zone harboring GABA-filled synaptic vesicles [11,13], suggesting that NMDAR-mediated Ca^2+^ entry is not spatially coupled to the release machinery. However, maximal release rates only occur at high local Ca^2+^ concentrations [50,51] requiring a close spatial relationship (a few nm) between the VDCCs and the release machinery [26]. Thus, synaptic NMDAR activation will not suffice to generate a Ca^2+^ transient sufficient to release GABA from GC spines, but the unphysiological conditions described above may result in a Ca^2+^ domain reaching from the glutamatergic postsynaptic density to the GABAergic active zone, where it could trigger the release of GABA. Consistent with nanodomains mediating GABA release, EGTA perfused into GCs did not affect dendrodendritic inhibition [20].

An MC action potential triggers recurrent inhibition with a delay of a few milliseconds (Fig 4). NMDARs activate rather slowly, half-maximal currents are reached after approximately 3 ms, and the peak current is reached after approximately 10 ms. Therefore, NMDAR-mediated Ca^2+^ [52] inflow into the GC will only occur after the peak of the MC recurrent hyperpolarization is already declining, consistent with our notion that synchronous GABA release from GCs cannot be mediated by NMDARs under physiological conditions.

What then are NMDARs needed for at the dendrodendritic synapse? Our previous work [16] has shown that deletion of NMDARs in GCs slows the EPSP time course, reduces MC inhibition, and slows odor discrimination time, albeit to a much smaller extent than reported here for deletion of Na_V_1.2. These results suggest that NMDARs are not essential for synchronous GABA release from GC spines under physiological conditions. Alternatively, NMDARs may enhance the local depolarization after AMPAR-mediated relief of Mg^2+^ block and may contribute a Ca^2+^ signal that could modulate the release machinery via high-affinity Ca^2+^ sensors or exert actions via other Ca^2+^-dependent pathways. This Ca^2+^ signal could also be detected by high-affinity release sensors and directly translated into long-lasting asynchronous release [49], consistent with the slow time course of the IPSC reported by previous studies [18,19]. Due to the long-lasting activation (hundreds of ms) of NMDARs caused by a single synaptic glutamate transient [53], repetitive glutamatergic input may recruit these mechanisms even more strongly and may transiently increase GABA release (see Fig 4A). Alternatively, NMDARs may establish synaptic plasticity mechanisms and contribute to local signals that do not reach VGSCs’ activation threshold.

### GC spiking and modes of MC inhibition

GCs have been shown to fire action potentials in response to odorants [2,4,54,55]. Moreover, awake behaving animals show much stronger GC activity with low temporal structure compared to anesthetized animals [55–57], implying that GCs modulate MC activity through extended lateral interactions independent of the respiratory cycle [55]. These observations contradict the claim of the GC spine being a “mini-neuron” [47], for which consequently the GC would appear rather silent, with dendritic spines working independently. We propose that EPSPs generated in several spines within close spatial proximity may depolarize the dendrites to firing threshold, causing the GC to fire a global action potential. In this context, T-type Ca^2+^ channels may play an important role in amplifying small glutamate receptor–mediated depolarizations [21] in gemmules and thereby generate a Ca^2+^ signal in the neighboring compartments of the dendritic tree. Our model of GC function allows establishing rapid and synchronous local recurrent inhibition [17,58] in parallel to global dendritic computations, resulting in lateral inhibition [18,21,22]. Yet these events cannot occur segregated or in a gradual fashion, as previously proposed [17,22,58,59], because once the depolarization reaches action potential threshold at the dendrite, a global action potential will be elicited. GC inhibition can shunt action potential propagation and consequently isolate dendritic compartments to compute stimuli from different sources. Indeed, GC dendrites can respond differently to odors than the soma [57] because of GC inhibitory mechanisms that might play a pivotal role to compartmentalize GC responses to a given stimulus. At the spine level, A-type voltage-gated potassium channels (VGKCs [20]) will dampen the developing interspike depolarization to temporally space successive action potentials more widely [60]. The A-type current may lead to the low spiking rate observed in GCs [55,61,62]. Altogether, we suggest that GCs use VGSCs to drive GABA release in a fast and synchronous manner at the GC spine and that generation of dendritic spikes at multiple dendritic domains allows for coincidence detection and dendritic integration.

### GCs’ specificity of viral transduction

The viral approach used in this study is not inherently selective for GCs. The parameters of stereotaxic injection can effectively limit viral spread and transduction to the GCL of the OB [16,38]. However, in addition to GCs, the GCL also contains deep short axon (dSA) cells, a heterogeneous population of cells classified based on their soma size, location, and morphology (reviewed in [39]). More than 99% of the cells analyzed in our study had dendrites of typical morphology reaching into the EPL and soma sizes of less than 10 μm (S6 Fig). On the functional level, knockdown of Na channels in dSA cells would cause a loss of dSA activity and would, based on their inhibitory nature and their synaptic connections with GCs [39,41], yield GCs more excitable. This would in turn increase MC inhibition (see [38]). However, we found the opposite, indicating that dSA cells were not perturbed in a physiologically relevant manner.

### Rapid odor discrimination requires VGSC expression in GCs

GCL-specific deletion of Na_V_1.2 channels resulted in a stimulus-dependent slowing of the time needed for highly accurate odor discrimination, not interfering with odor discrimination learning and accuracy (Fig 5). These results recapitulate our previous observations after GCL-specific deletion of the NMDA-type glutamate receptor subunit 1 (GluN1) [16]. Nevertheless, Na_V_1.2 deletion had a much stronger effect on the rIPSP amplitude, indicating that AMPARs remaining in the absence of NMDARs are sufficient to maintain dendrodendritic inhibition at a level stronger than after Na_V_1.2 knockdown. Interestingly, both GluN1 deletion and Na_V_1.2 knockdown resulted in a stimulus-dependent phenotype: only discrimination of highly similar binary mixtures, but not of dissimilar stimuli, was affected. In conclusion, under physiological conditions, Na_v_1.2 activation is crucial for inhibitory interactions of GCs and MCs, underlying an important molecular mechanism for the OB to enhance discrimination of highly similar activity patterns.

What is the mechanistic link between altered MC inhibition and odor discrimination behavior? This and our previous work [16,38] suggest a correlation between the strength of dendrodendritic inhibition and discrimination time: stronger inhibition will accelerate odor discrimination and vice versa. On the cellular level, the simplest mechanism causing a shift in discrimination time could be the time required to build up a hypothetical level of recurrent MC inhibition sufficient to discriminate similar stimuli [16]. However, understanding how network connectivity, neuronal ensemble formation, decision-making, and coupling of olfactory and motor areas give rise to odor discrimination behavior remains a challenge to be addressed.

## Materials and methods

### Ethics statement

Mice used in this study were handled in agreement with the European FELASA guidelines, and all procedures were approved by the national authorities, Regierungspraesidium Karlsruhe, Germany, under the approved protocol number 35–9185.81/G-100/09. All animal care procedures were conducted in agreement with the European Directive 2010/63, at the Heidelberg University, under the supervision of the Interfakultaere Biomedizinische Forschungseinrichtung.

### Mice

In all experiments, mice were housed in standard cages, in a constant day–night cycle (12 hours–12 hours) and in a temperature- (22 ± 2 °C) and humidity- (60% ± 4%) controlled environment. Individuals used for behavior were kept in an inverted light cycle, and the experiments were performed in the night period. All mice (strain C57Bl6) were purchased from Charles River.

### RT-PCR and western blotting

To assess VGSC expression in the main OB, RNA was extracted from whole brain, whole OB, heart, and muscle using TRIzol (Ambion, cat. # 15596–018). For each extract, a cDNA library was created by inverse transcription using SuperScript II kit (Invitrogen, cat.# 18064–014). PCRs were made using primers previously published [63,64] for the different subunits. Primer specificity was tested using whole brain, heart, and muscle cDNA. All known mRNAs could be detected by rT-PCR.

Due to the high molecular weight of the VGSCs subunits, western blots were done following the protocol of Fairbainks [65,66]. Antibodies against Na_V_1.1 (AB_2238842), Na_V_1.2 (AB_2184197), Na_V_1.6 (AB_2184197), and Na_V_1.7 (AB_2184355) were purchased from Neuromab (Antibodies, United States of America). The antibody against the Na_V_1.3 (pab0279-P) subunit was purchased from Covalab (France). These antibodies were also used in the immunohistochemistry procedures.

### Design of shRNAs

The design of shRNA molecules was performed using the InvivoGen algorithm (www.sirnawizard.com). To restrict the number of candidate molecules, the following constrains were used: (1) sequences containing TTATT were discarded (known to induce immune response), (2) sense and antisense oligos should contain 21 nts length each, (3) C-G amount <70%. The sequence of the shRNA loop was TAATATTAT. Specificity was controlled by determining the E value with BLAST (blast.ncbi.nlm.nih.gov). Four molecules (sense sequences: shRNA#5: GAA AGC AAT CTC TCG GTT CAG; shRNA#14: GTT GGA AGA CCC TAC ATC AAG; shRNA#22: GAT GGA AAC GGG ACG ACC AGT; shRNA#44: GTG GAC CTC CCG ATT GTG ACC) were selected and tested for their efficacy in reducing Na^+^ currents in GCs (S4 Fig). For the most effective shRNA, a shRNAmm was produced by changing each third base of the original oligo (mmhRNA#14: GTA GGG AGT CCG TAG ATG AAC). To avoid possible interferences with other cellular mRNAs, the specificity of the mismatch molecule was predicted using BLAST.

### Viral gene transfer

Mice, 21 days old, were stereotaxically injected using a stereotax (myNeurolab, USA). The rAVV chimeric vectors (1:1 ratio of AAV1 and AAV2 capsid proteins) carrying rAAV-specific expression cassettes (pAM plasmid) were injected in the GCL of the OB, as previously described [16]. For 3D-immunohistochemistry, mGFP expression under the chicken beta actin (CAG) promoter was used. For in vitro whole-cell electrophysiology and behavioral experiments, control plasmids expressed eGFP under the CAG promoter. Expression of shRNAs was driven by the U6 promoter with bicistronic expression of eGFP under the control of the CAG promoter.

### Three-dimensional immunohistochemistry

This approach combines viral-mediated labeling of cells with immunohistochemistry [33]. Fixed tissue sections containing neurons labeled with mGFP for precise detection of even thin processes (typically not visible with soluble cytoplasmic eGFP expression) were treated with the antibody of interest. Dual-color confocal image stacks were acquired in the serial scanning mode. Using ImageJ or AMIRA software, the 3D morphology template of the cell, as delineated by mGFP, was used to excise the immunohistochemistry signals residing within the cell of interest. To reconstruct full GC cell bodies or EPL dendrites, 50–100 consecutive confocal image frames were used. The result was rendered in 3D and shows the distribution of the immunosignal within the labeled cell.

Practically, 21-day-old wild-type mice were stereotaxically injected with rAAV-mGFP, and after 3–4 weeks of expression, mice were transcardially perfused using 4% paraformaldehyde (PFA). The brains were removed, and free-floating 50-μm-thick slices of the OB were prepared using a vibratome. Afterwards, the slices were incubated for 45 minutes at room temperature in vehicle buffer (10% normal goat serum, 1% bovine serum albumin fraction V, 0.3% Triton X-100, in 1x PBS, pH = 7.4) with 0.1% cold fish gelatin (blocking solution). The antibody against the Na_V_1.2 subunit was carried in vehicle (1:1,000 dilution) and incubated overnight at 4 °C. After the third wash in vehicle (10 minutes each wash step), the alexa dye conjugated secondary antibody (1:1,000 in vehicle; life technologies cat.#: A-21244) was incubated for 90 minutes at room temperature. The slices were washed three times in 1x PBS, pH = 7.4, and mounted on coverslips using Moviol. All incubation steps were performed under gentle agitation using a horizontal rocking shaker (neoLab, Germany).

Images were acquired in a confocal microscope (Leica SP5, Leica, Germany) using a 63× glycerol immersion objective (NA = 1.3).

#### Quantification of Na_V_1.2-expression in the OB

Immunohistochemistry was performed as described in the previous section. All sections were stained in parallel to ascertain identical conditions for quantitative comparison. Single-frame confocal images (233.94 μm × 233.94 μm with a pixel size of 0.228 μm) were acquired and analyzed without additional image processing. The average gray value was quantified in control, shRNAmm, and shRNA-treated mice (in each condition, 12 images taken from 2 mice), and ratios were calculated for each condition, using the mean gray value of the control.

#### Quantification of control (eGFP), shRNA#14 or shRNAmm expression efficiency

To access the total number of nuclei (DAPI, Sigma-Aldrich cat. # D9542-1MG), total number of neural cells (NeuN, Millipore, cat. # MAB377), and total number of eGFP-positive cells, 4% PFA-fixed 50-mm-thick horizontal OB slices were used that were obtained from mice stereotaxically injected with rAAV-eGFP, rAAV-shRNA#14 or rAVV-shRNAmm used in behavior (*n* = 16). Stacks of images (200 × 200 mm in the x-y plane and 0.15 mm in z plane) of randomly selected regions in the GCL were acquired in a confocal microscope (Leica SP5). From each mouse, 3–5 slices were used, and in each slice, 8–10 regions were imaged. To count nuclei, maximal intensity projections of stacks of 10 images were used, using the ImageJ cell counter built-in function.

### Electrophysiology

OB horizontal 300-μm-thick slices were prepared from mice, 6–10 weeks old, using a vibratome (Leica) while submersed in ice-cold oxygenated slicing solution (in mM): 125 NaCl, 2.5 KCl, 25 NaHCO_3_, 1.25 NaH_2_PO_4_, 3 myo-Inositol, 2 Na-pyruvate, 0.4 ascorbic acid, 0.1 CaCl_2_, 3 MgCl_2_, 25 glucose. Slices were transferred and incubated for about 30 minutes in a 37 °C warm oxygenated bath solution (in mM): 125 NaCl, 2.5 KCl, 25 NaHCO_3_, 1.25 NaH_2_PO_4_, 2 CaCl_2_, 1 MgCl_2_, 25 glucose. This bath solution was continuously aerated with carbogen and used for all recordings at a temperature of 33–35 °C.

Whole-cell recordings were established using an EPC9 amplifier (HEKA, Lambrecht, Germany). To evaluate Na^+^ currents in GCs, voltage-clamp recordings were performed using pipettes with resistances ranging 3.5–4.5 MΩ, filled with (in mM) 130 CsCl, 4 TEA-Cl, 10 Na_2_-phosphocreatine, 10 HEPES, 5 EGTA, 4 Mg-ATP, 0.3 Na-GTP, 2 Ascorbate, pH = 7.2 with CsOH. TEA (10 mM) was added to the bath solution. Current-clamp recordings were established from MCs to access recurrent inhibition. To induce a single action potential, a 3-ms current pulse was used. To induce 100-Hz stimulation, 3-ms current pulses interleaved with 8 ms of no current injection were used. Pipettes had resistances of 3–4 MΩ, filled with 135 K-gluconate, 10 HEPES, 10 Na_2_-phosphocreatine, 4 MgATP, 4 KCl, 0.3 Na-GTP, pH = 7.2 adjusted with KOH. All recordings were performed using a micro-salt bridge in the electrode attached to the pipette holder [67]. To access cell morphology, the dye alexa 594 hydrazide (10 μM; Molecular Probes, cat.# A10438) was added routinely in the intracellular solutions in all experiments. For action potential recordings in GCs, the same solutions were used. Current injections of 1-ms duration and 100-ms duration were used to analyze action potential firing in GCs.

### Behavior

For the behavioral experiments, animals were kept separated in macrolon type II cages in a temperature- and humidity-controlled environment operated under a reverse light cycle (12 hours–12 hours). Experiments were conducted in a dark room during the night period directly adjacent to the animal room. The behavioral protocol was described previously [16,44]. The behavioral pretraining started 4–6 weeks after the surgery and 2–3 days before the animals were kept under water restriction (by periods no longer than 12 hours). The weight of the animals was strictly monitored and kept >85% of the initial body weight. The pretraining took 3–5 days, and the behavioral training took usually no longer than 8 weeks. The control of the eight-channel semiautomated olfactometers [68] (Knosys, Washington) and data acquisition were carried out with custom programmed software (S1 File) written in Igor Pro 6 (Wavemetrics). Odor presentation tasks began after all animals finished the pretraining successfully. All odors were diluted to 1% in mineral oil and further air-diluted at a 1:20 ratio in the olfactometers. Odors were presented in a fully randomized way, with no more than 5 consecutive trials having the same stimulus. Bias toward any of the odors presented was avoided by counterbalancing between animals. The odor pair CvE was used for task habituation; usually, 400 trials sufficed to achieve an accuracy >80%. Test odors (AAvEB and 6v4/4v6) were used to determine the reaction times.

### Data analysis

To study the localization and distribution of the Na_V_1.2 subunit in GCs, the ImageJ software was used for data visualization. Electrophysiology and behavior data analyses (S1 File) were performed using custom software written in Igor Pro6 (Wavemetrics). All data are presented as mean ± SEM. ANOVA refers to one-way ANOVA, and *t*-values were derived from the Bonferroni multiple comparison test, except when otherwise denoted. Statistical analyses were performed in Prism 5 (GraphPad Software).

## Supporting information

S1 Fig Epitope specificity of the antibodies against the VGSCs subtypes Na_v_1.1 and Na_v_1.2. (A) The epitope sequences that the antibodies against the VGSCs subtypes Na_v_1.2 (#1) and Na_v_1.1 (#2) recognize were cloned in a pAM vector backbone. Expression was driven under the CMV. The Na_v_1.2 epitope consisted of the amino acids 1882–2005 of the Na_v_1.2 protein sequence, while the Na_v_1.2 epitope comprehended the amino acids 1929–2009 of the Na_v_1.1 protein sequence. To control for transfection efficiency, a plasmid carrying eGFP (#3) was used. (B) HEK293 cells were successfully transfected using pAM-eGFP, predicting good transfection of pAM #1 and pAM #2. (C and D) Left panels show that the antibody against Na_v_1.1 is specific in recognizing its epitope, and right panels show that the antibody against Na_v_1.2 is also specific in recognizing its epitope. For the western blot, (C) HEK293 cells extracts were made using RIPA lysis buffer, from cells transfected with pAM #1 (lane 1), pAM #2 (lane 2), and pAM #3 (lane 3) and from nontransfected (lane 4). The approximately 50-kDa band for the epitope recognized by the antibody against the Na_v_1.1 subtype and the approximately 35-kDa band for the epitope recognized by the antibody against the Na_v_1.2 subtype are in agreement with the expected size. Smaller bands often occur in overexpression systems. (D) Immunocytochemistry of HEK293 cells fixed with 4% PFA confirm the antibody specificity. CMV, cytomegalovirus promoter; eGFP, enhanced green fluorescent protein; HEK293, human embryonic kidney 293; PFA, paraformaldehyde; RIPA, radioimmunoprecipitation assay; VGSC, voltage-gated sodium channel.
(TIF)

S2 Fig Nav1.1, Nav1.3, and Nav1.6 are not expressed in GCs. Stereotaxic injection of rAAV-mGFP in the GCL was used to label GCs. Immunohistochemistry was performed in horizontal OB slices, and stacks of image frames were acquired by confocal microscopy. 3D reconstructions were made in ImageJ using the GFP signal of 100–200 consecutive image frames. The antibody signal was excised through frame-by-frame multiplication with the GFP signal template. GCs show no expression of (A) Na_v_1.1, (B) Na_v_1.3, and (C) Na_v_1.6 in the cell body, dendritic stem (upper panels in A, B and C), dendritic shafts, and gemmules (lower panels in A, B and C). In the GC somas, we have observed unspecific immunosignals (white arrows) overlapping with the mGFP signal. GC, granule cell; GCL, granule cell layer; GFP, green fluorescent protein; mGFP, membrane-bound GFP; OB, olfactory bulb; rAAV, recombinant adeno-associated virus.
(TIF)

S3 Fig GCs’ Na^+^-currents are strongly reduced by phrixotoxin-3, a specific inhibitor of Na_V_1.2 channels. (A) Whole-cell voltage-clamp recordings were established from GCs. Series of voltage square pulses from −40 mV to +10 mV, increasing 10 mV per step, with 5-ms duration were used to record Na^+^ currents in bath solution supplemented with 10 mM TEA at 34 ± 1 °C. (B) Bath application of 1 nM phrixotoxin-3 (red) strongly reduced the Na^+^ current in GCs at −30 mV, while application of 1 μM TTX (blue) abolished Na^+^ currents. The small increase of the current approximately 2.5 ms after onset of the square pulse was found in most recordings done in the presence of phrixotoxin-3. While the mechanism underlying this effect is unclear, it does not affect our conclusion that phrixotoxin-3 strongly blocks Na^+^ currents in GCs. (C) Quantification of peak amplitudes recorded from GCs at different membrane potentials (*n* = 4; ANOVA, *F* = 112.50, *p* < 0.001; Bonferroni multiple comparison test, **p ≤ 0.01, ***p ≤ 0.001). (D) Whole-cell voltage-clamp recordings from MCs performed as described in A. (E) Bath application of 1 nM phrixotoxin-3 (red) affects Na^+^ currents only weakly, while 1 μM TTX (blue) completely abolished Na^+^ currents at −30 mV in MCs. (F) Quantification of peak amplitudes recorded from MCs at different membrane potentials *n* = 4; ANOVA, *F* = 45.71, *p* < 0.001; Bonferroni multiple comparison test, **p ≤ 0.01, ***p ≤ 0.001). Data used in the generation of this figure can be found in S1 Data. GC, granule cell; MC, mitral cell; TEA, tetraethylammonium; TTX, tetrodotoxin.
(TIF)

S4 Fig Knockdown of Na_v_1.2 strongly reduces Na^+^-currents in GCs. (A) shRNAs were designed using the InvivoGen Wizard (www.sirnawizard.com). Four suitable target sequences were identified on the SCN2A mRNA. rAAV1/2 vectors mediating shRNA expression driven by the U6 promotor and GFP expression from the CBA promoter. rAAV was injected into the OB (see Materials and methods). (B-E) Voltage-clamp recordings were established from transduced and control GCs in 300-μm-thick OB slices at 34 ± 1 °C. Series of voltage square pulses ranging from −70 mV to +10 mV per step, with 5-ms duration, were applied to assess the amplitude of Na^+^ currents in each pulse tested. Four shRNA molecules were tested (B-E), and each affected the Na^+^ current differently. (B) The shRNA#5 targeted nucleotides 291–312 and reduced the Na^+^ current by approximately 60% compared to control. (C) The shRNA#14 targeted nucleotides 2085–2106 and reduced the Na^+^ current by approximately 90% relative to control. (D) The shRNA#22 targeted nucleotides 3211–3232 and reduced the Na^+^ current by approximately 75% relative to control. (E) The shRNA#44 targeted nucleotides 5180–5201 and reduced the Na^+^ current by approximately 45% compared to control. Data used in the generation of this figure can be found in S1 Data. CBA, chicken beta actin; GC, granule cell; GFP, green fluorescent protein; OB, olfactory bulb; rAAV, recombinant adeno-associated virus; shRNA, short hairpin RNA.
(TIF)

S5 Fig In silico simulation of the specificity of the shRNA#14 to target specifically the SCN2a gene in mice. (A) Amino acid sequence similarity among the Na_v_1.x subtypes. Sequences were aligned in the CLC sequence viewer software, using the UPGMA algorithm. The color code depicts the most prominent expression location as indicated in the color legend. The resistance of each subtype to TTX is indicated gray. (B) Phylogenetic relationship of the mouse VGSCs α-subunits. The published nucleotide sequences in PubMed Central were aligned using the neighbor-joining algorithm, with a gap open cost of 7, gap extension cost of 3, and gap end was free. Bootstrapping analysis was performed, and the values shown in the ramification branches of the tree represent the number of replications. The tree was rooted using a VGSC expressed in *Drosophila melanogaster* (not shown). The scale bar represents 100 nucleotide substitutions. The tree was generated using the CLC sequence viewer software. (C) Coding sequence similarities among the most similar Na_V_1.x brain subtypes with the gray line delineating the target region of shRNA#14. The sequence similarity is 62% among Na_V_1.1 and Na_V_ 1.3 and 76% between Na_V_1.1 and Na_V_ 1.2. (D) The shRNA#14 is highly specific for SCN2a. BLAST of the 21-nucleotide sense sequence of shRNA#14 against the sequences of the VGSCs brain subunits demonstrates that this molecule has very low probabilities to knock down SCN1a, SCN3a, and SCN8a (E value > 0.1) at any location within the coding region of the VGSCs subunits (left side of the panels). The shRNA#14 shows a region in the SCN2a gene with 100% similarity (red arrow), so that the E value is very low for this region (2e^-8^), indicating high probability of knockdown of the Na_v_1.2 subunit. shRNA, short hairpin RNA; TTX, tetrodotoxin; UPGMA, unweighted pair group method with arithmetic mean; VGSC, voltage-gated sodium channel.
(TIF)

S6 Fig GCL-restricted expression in a large fraction of GCs reveals GC specificity of acute targeted genetic perturbations within the OB. (A) rAAV1/2 particles were delivered into the GCL of the OB, and spread of infection was assessed by imaging eGFP, rAAV-mm#14//eGFP (mm), and rAAV-shRNA#14//eGFP (kd). All images were acquired with a wide-field fluorescence microscope. DAPI staining identifies OB layers (top-down: GCL, MCL, EPL, GL). (B) Low-resolution wide-field epifluorescence image of a typical injection directed to the GCL of the OB with GFP expression in the GC domain of the OB (somas located in the GCL, dendrites that transverse the GCL toward the EPL and dendritic arborizations very prominent in the EPL). No fluorescence is observed in cortical and subcortical regions. (C) Maximal projection of a confocal image stack showing GFP expression in the GCL (10–15 consecutive stacks; *n* = 3 preparations from one mouse each); 3 fields of 200 μm × 800 μm per preparation were chosen randomly to determine the dimensions of GFP-expressing cells. (D) Histogram showing the distribution of the soma diameters of GFP-expressing cells in the GCL. A total of 510 cells were analyzed. According to Nagayama and colleagues [39], GCs are the only cells that extend their dendrites into the EPL, as was the case for all GCs analyzed. Furthermore, GCs have much smaller somas (6–8 μm) than dSA cells (10–20 μm). (E) Cumulative distribution of the data shown in C; 99% of the infected cells have a soma diameter of less than 10 μm and hence can be classified as GCs. (F) Representative confocal image stacks (MIP of 10 image frames) of OB horizontal sections of mice stereotaxically injected with rAAV-eGFP (“control,” *n* = 4 mice), rAAV-mmshRNA#14 (“mm,” *n* = 6 mice), or rAAV-shRNA#14 (“kd,” *n* = 6 mice). DAPI-stained nuclei (blue), anti-NeuN antibody labels neuronal nuclei (magenta), and GFP reveals transduced GCs (green). (G-H) Nuclei were counted in OB horizontal sections for each labeling condition. From each OB analyzed, 5 stacks of 10 image frames each were taken from random regions of the GCL. (C) Total number of nuclei: control: 529.7 ± 22.07, mm: 486.5 ± 34.40, kd: 520.0 ± 30.82; ANOVA, *F* = 0.59, *p* = 0.58. (D) Proportion of neurons: control: 73.92% ± 2.25%, mm: 80.43% ± 2.20%, kd: 76.16% ± 3.84%; ANOVA, *F* = 1.33, *p* = 0.31. Data used in the generation of this figure can be found in S1 Data. DAPI, 4,6-diamidino-2-phenylindole; dSA, deep short axon; eGFP, enhanced GFP; EPL, external plexiform layer; GC, granule cell; GCL, granule cell layer; GFP, green fluorescent protein; GL, glomerular layer; MCL, mitral cell layer; MIP, maximum intensity projection; OB, olfactory layer; rAAV, recombinant adeno-associated virus; shRNA, short hairpin RNA.
(TIF)

S7 Fig MC inhibition depends on the activation of AMPA, NMDA, and GABA_A_ receptors. (A) Single action potentials (left) or 20 consecutive action potentials at 100 Hz (right) were evoked in MCs (*n* = 3) through somatic current injections. Recordings were made at 34 ± 1 °C. Black traces represent control recordings in bath solution; green traces represent recordings in bath solution supplemented with 10 μM CNQX and 50 μM APV. The hyperpolarization was nearly abolished upon a 25-minute bath application of the drugs (single action potential: 0.88 ± 0.25 mV; 100 Hz: 1.43 ± 0. 13 mV) in comparison to the control situation (single action potential: 8.79 ± 0.52 mV; 100 Hz: 12.50 ± 0.87 mV; Student *t* test, *p* = 0.009 and *p* = 0.008, respectively). (B) As in (A) but using 20 μM Gabazine (blue). The rIPSP amplitude was significantly reduced upon a 25-minute bath application of Gabazine (single action potential: 0.83 ± 0.11 mV; 100 Hz: 0.88 ± 0.20 mV) in comparison to control conditions (single action potential: 8.00 ± 0.33 mV; 100 Hz: 11.65 ± 1.47 mV; Student *t* test, *p* = 0.002 in both situations). Data used in the generation of this figure can be found in S1 Data. AMPA, α-amino-3-hydroxy-5-methyl-4-isoxazolepropionic acid; APV, 2-amino-5-phosphonopentanoic acid; CNQX, 6-cyano-7- nitroquinoxaline-2,3-dione; GABA, gamma-aminobutyric acid; MC, mitral cell; NMDA, N-methyl-D-aspartate; rIPSP, recurrent inhibitory postsynaptic potential.
(TIF)

S8 Fig Relative contribution of synaptic and nonsynaptic mechanisms to MC hyperpolarization. Quantitative comparison of Na_V_1.2 knockdown and pharmacological blockade of dendrodendritic communication based on the data shown in Fig 5 and S7 Fig. Glutamate receptor and GABA_A_ receptor blockade reduced the hyperpolarization by 90%. Assuming a close to complete block of all glutamate receptors and GABA_A_ receptors of the dendrodendritic synapse, the remaining 10% of the hyperpolarization could be attributed to a nonsynaptic mechanism such as the AHP [58]. Na_V_1.2 knockdown reduced the hyperpolarization amplitude by 60%–75%. The less pronounced block caused by the genetic manipulation can be explained by the incomplete transduction of the GC population (Fig 4). MCs receive gemmules of GCs from a mixed population: GCs with a reduced number of Na_V_1.2 and unperturbed GCs with a normal complement of Na_V_1.2 channels. As we have demonstrated above (Fig 4, S6 Fig), our genetic perturbation does not affect MCs and hence cannot be attributed to any MC-intrinsic mechanisms. Furthermore, phrixotoxin-3 did not have an effect on voltage-gated conductances in MCs (S3D–S3F Fig), indicating that even Na_V_1.2 knockdown in MCs would not affect action potential firing. Taken together, the hyperpolarization determined after Na_V_1.2 knockdown includes nonsynaptic components such as the AHP or I_H_ but mostly reflects the rIPSP. Subtracting these approximately 10% nonsynaptic contributions from control and knockdown conditions would yield a reduction of the rIPSP by approximately 70%–80%. (A) Single MC action potential. ANOVA, *F* = 17.25, *p* < 0.0001. *** denotes highly significant difference relative to control and mm. No significant difference was found when comparing kd, APV+CNQX, and GABAzine conditions or when comparing control and mm. (B) Twenty consecutive action potentials at 100 Hz elicited in MCs. ANOVA, *F* = 18.02, *p* < 0.0001. *** denotes highly significant difference relative to control and mm. No significant difference was found when comparing kd, APV+CNQX, and GABAzine conditions or when comparing control and mm. Data used in the generation of this figure can be found in S1 Data. APV+CNQX, 2-amino-5-phosphonopentanoic acid and 6-cyano-7- nitroquinoxaline-2,3-dione; AHP, afterhyperpolarization; GABA, gamma-aminobutyric acid; GC, granule cell; MC, mitral cell; rIPSP, recurrent inhibitory postsynaptic potential.
(TIF)

S1 Data Summary of the data. Excel spreadsheet containing, in separate sheets, the underlying numerical data for Figs panels 1D, 1E, 2H, 3B, 3F, 3H, 4C, 5A, 5B, 5C, 5D, S3C, S3F, S4B, S4C, S4D, S4E, S6D, S6E, S6G, S6H, S7A, S7B, S8A and S8B.
(XLSX)

S1 File Compilation of the custom-made software used for data processing and analysis. This compilation includes code for electrophysiology, behavior data acquisition, and behavior data analysis in separate chapters. All supporting software was written in Igor Pro6 (Wavemetrics).
(PDF)

## Abbreviations

6v4/4v6: binary 6:4 mixtures of amyl acetate and ethyl butyrate
AAV: adeno-associated virus
AAvEB: amyl acetate versus ethyl butyrate
AMPAR: α-amino-3-hydroxy-5-methyl-4-isoxazolepropionic acid receptor
APV: 2-amino-5-phosphonopentanoic acid
CNQX: 6-cyano-7- nitroquinoxaline-2,3-dione
CvE: cineol versus eugenol
DAPI: 4,6-diamidino-2-phenylindole
DRG: dorsal root ganglia
dSA: deep short axon
eGFP: enhanced green fluorescent protein
EPL: external plexiform layer
EPSP: excitatory post-synaptic potential
GABA: gamma-aminobutyric acid
GABAR: GABA receptor
GC: granule cell
GCL: granule cell layer
GFP: green fluorescent protein
GL: glomerular layer
HEK293: human embryonic kidney 293
GluN1: NMDA-type glutamate receptor subunit 1
MC: mitral cell
MCL: mitral cell layer
mGFP: membrane-bound green fluorescent protein
MIP: maximum intensity projection
NMDA: N-methyl-D-aspartate
NMDAR: NMDA receptor
OB: olfactory bulb
ONL: olfactory nerve layer
ORN: olfactory receptor neuron
PFA: paraformaldehyde
rAAV: recombinant adeno-associated virus
ROI: region of interest
rT-PCR: reverse transcriptase PCR
rIPSP: recurrent inhibitory postsynaptic potential
shRNA: short hairpin RNA
shRNAmm: mismatch shRNA
TRPC: transient receptor potential-canonical
TTX: tetrodotoxin
VDCC: voltage-dependent calcium channel
VDKC: voltage-dependent potassium channel
VGSC: voltage-gated sodium channel
VGKC: voltage-gated potassium channel
