# Supplementary figures and images for: Axonal sodium channel NaV1.2 drives granule cell dendritic GABA release and rapid odor discrimination
Source: PLoS Biol. 2018 Aug 20;16(8):e2003816. doi: 10.1371/journal.pbio.2003816 (PMC6117082; doi:10.1371/journal.pbio.2003816)

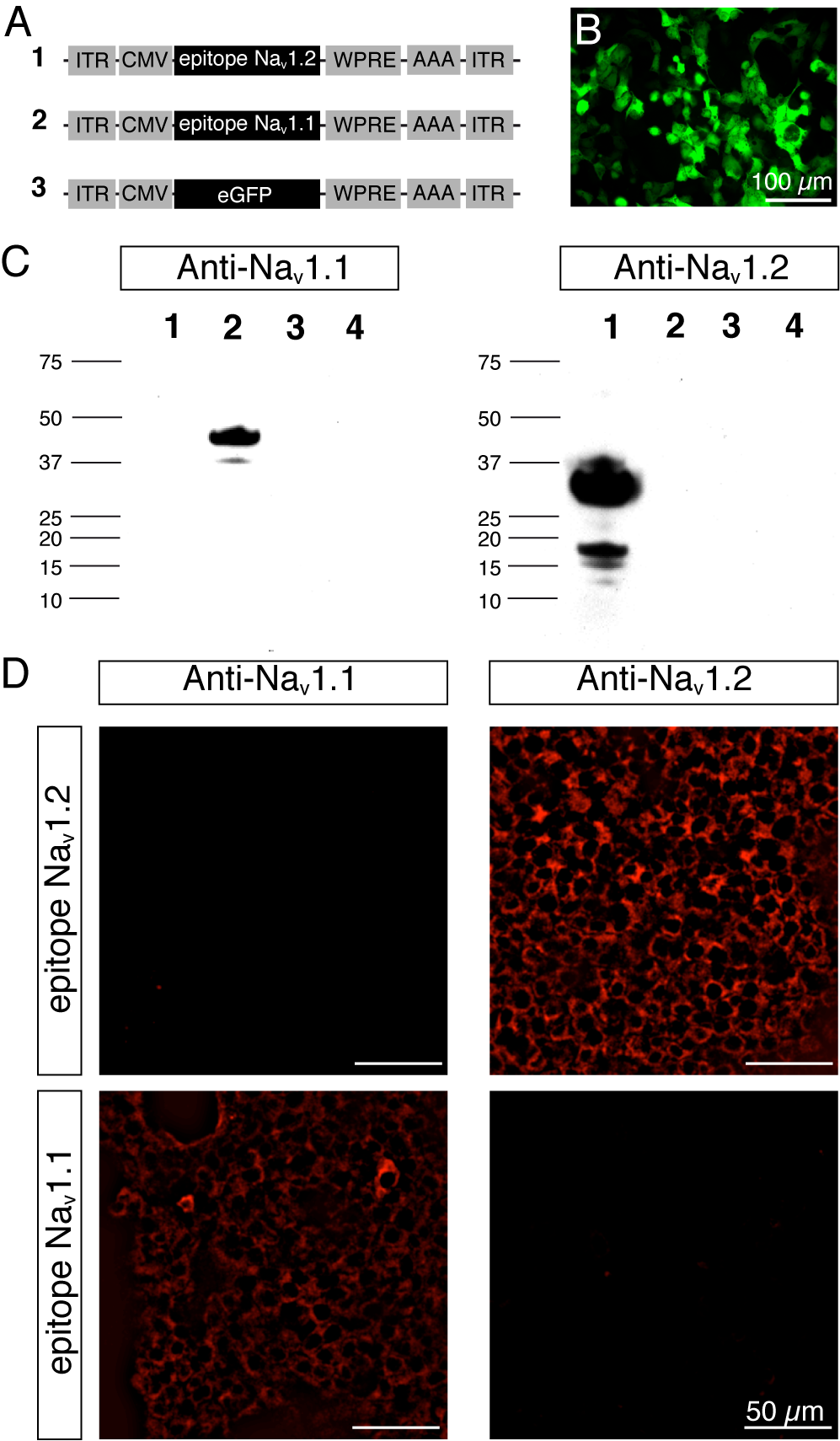

Supplement: S1 Fig — (A) The epitope sequences that the antibodies against the VGSCs subtypes Nav1.2 (#1) and Nav1.1 (#2) recognize were cloned in a pAM vector backbone. Expression was driven under the CMV. The Nav1.2 epitope consisted of the amino acids 1882–2005 of the Nav1.2 protein sequence, while the Nav1.2 epitope comprehended the amino acids 1929–2009 of the Nav1.1 protein sequence. To control for transfection efficiency, a plasmid carrying eGFP (#3) was used. (B) HEK293 cells were successfully transfected using pAM-eGFP, predicting good transfection of pAM #1 and pAM #2. (C and D) Left panels show that the antibody against Nav1.1 is specific in recognizing its epitope, and right panels show that the antibody against Nav1.2 is also specific in recognizing its epitope. For the western blot, (C) HEK293 cells extracts were made using RIPA lysis buffer, from cells transfected with pAM #1 (lane 1), pAM #2 (lane 2), and pAM #3 (lane 3) and from nontransfected (lane 4). The approximately 50-kDa band for the epitope recognized by the antibody against the Nav1.1 subtype and the approximately 35-kDa band for the epitope recognized by the antibody against the Nav1.2 subtype are in agreement with the expected size. Smaller bands often occur in overexpression systems. (D) Immunocytochemistry of HEK293 cells fixed with 4% PFA confirm the antibody specificity. CMV, cytomegalovirus promoter; eGFP, enhanced green fluorescent protein; HEK293, human embryonic kidney 293; PFA, paraformaldehyde; RIPA, radioimmunoprecipitation assay; VGSC, voltage-gated sodium channel. (TIF) [file pbio.2003816.s001.tif]

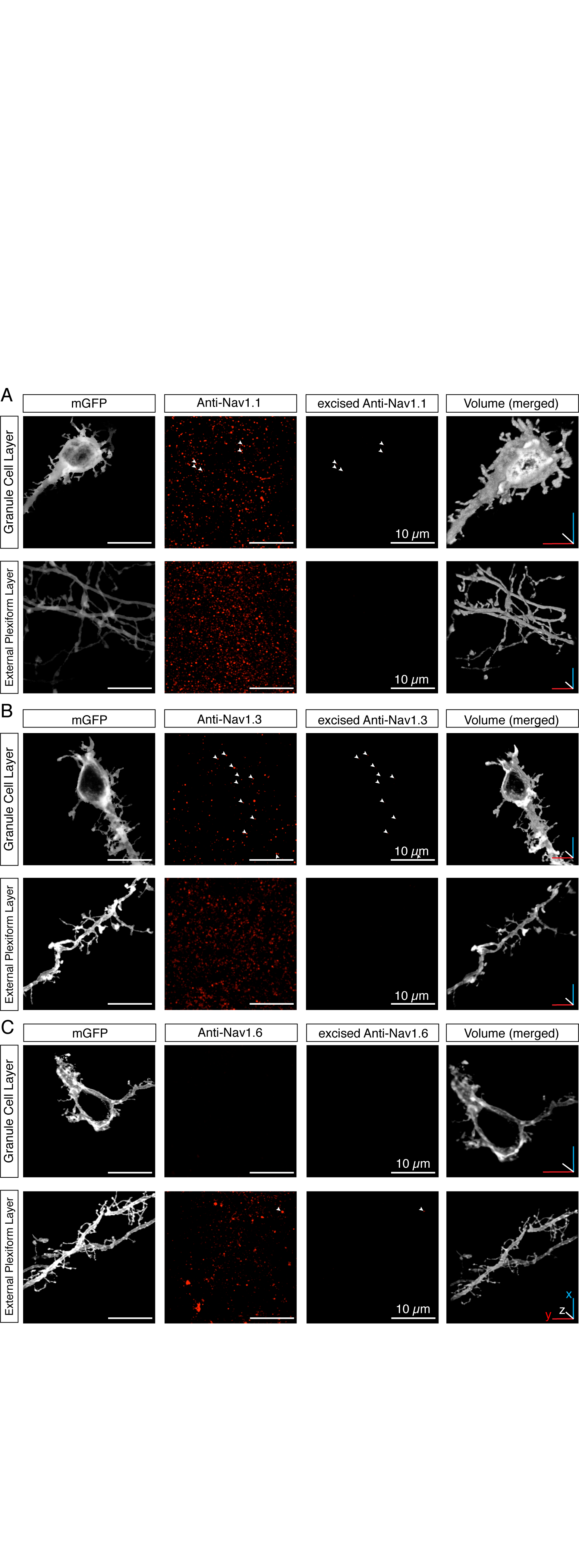

Supplement: S2 Fig — Stereotaxic injection of rAAV-mGFP in the GCL was used to label GCs. Immunohistochemistry was performed in horizontal OB slices, and stacks of image frames were acquired by confocal microscopy. 3D reconstructions were made in ImageJ using the GFP signal of 100–200 consecutive image frames. The antibody signal was excised through frame-by-frame multiplication with the GFP signal template. GCs show no expression of (A) Nav1.1, (B) Nav1.3, and (C) Nav1.6 in the cell body, dendritic stem (upper panels in A, B and C), dendritic shafts, and gemmules (lower panels in A, B and C). In the GC somas, we have observed unspecific immunosignals (white arrows) overlapping with the mGFP signal. GC, granule cell; GCL, granule cell layer; GFP, green fluorescent protein; mGFP, membrane-bound GFP; OB, olfactory bulb; rAAV, recombinant adeno-associated virus. (TIF) [file pbio.2003816.s002.tif]

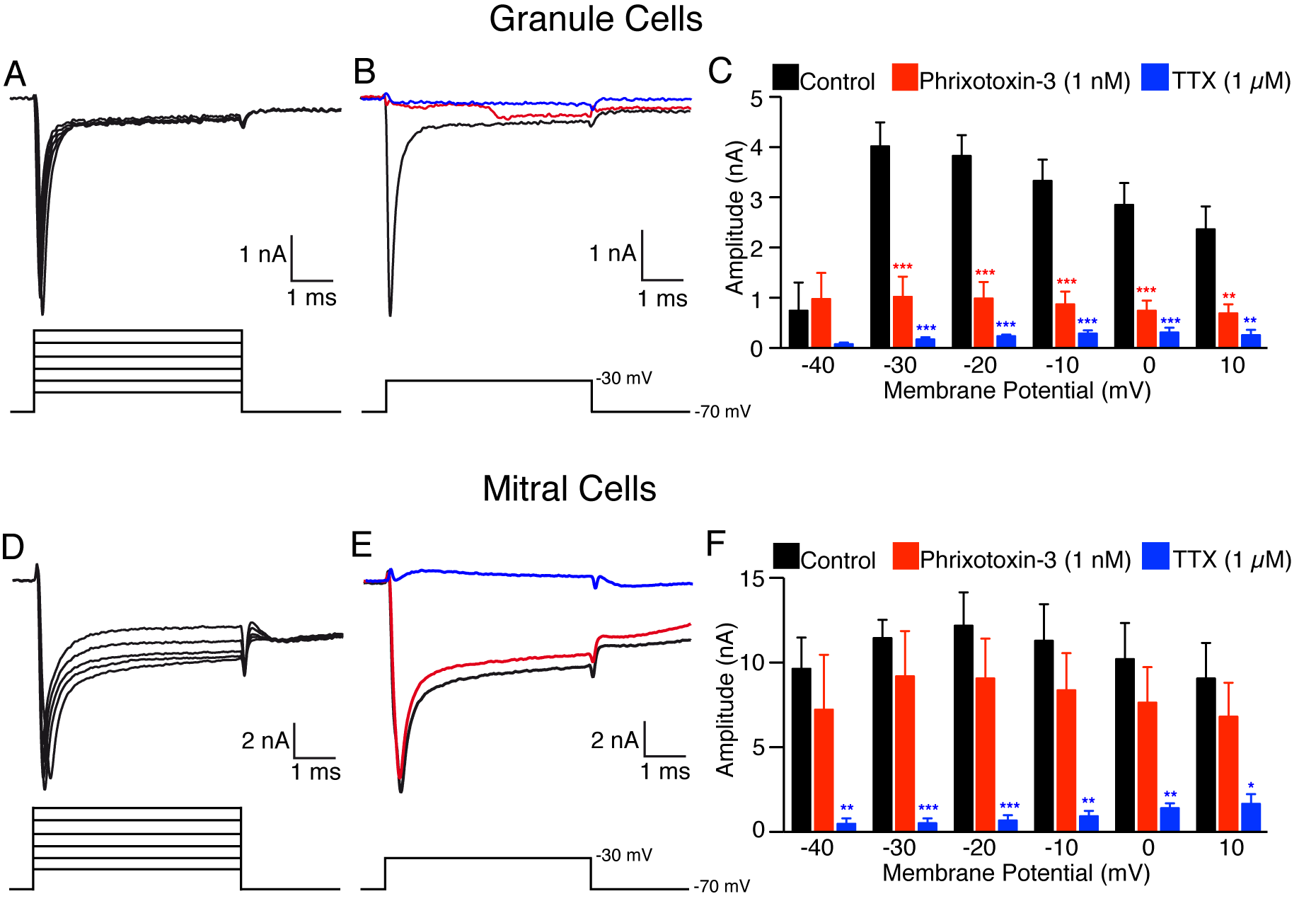

Supplement: S3 Fig — (A) Whole-cell voltage-clamp recordings were established from GCs. Series of voltage square pulses from −40 mV to +10 mV, increasing 10 mV per step, with 5-ms duration were used to record Na+ currents in bath solution supplemented with 10 mM TEA at 34 ± 1 °C. (B) Bath application of 1 nM phrixotoxin-3 (red) strongly reduced the Na+ current in GCs at −30 mV, while application of 1 μM TTX (blue) abolished Na+ currents. The small increase of the current approximately 2.5 ms after onset of the square pulse was found in most recordings done in the presence of phrixotoxin-3. While the mechanism underlying this effect is unclear, it does not affect our conclusion that phrixotoxin-3 strongly blocks Na+ currents in GCs. (C) Quantification of peak amplitudes recorded from GCs at different membrane potentials (n = 4; ANOVA, F = 112.50, p < 0.001; Bonferroni multiple comparison test, **p ≤ 0.01, ***p ≤ 0.001). (D) Whole-cell voltage-clamp recordings from MCs performed as described in A. (E) Bath application of 1 nM phrixotoxin-3 (red) affects Na+ currents only weakly, while 1 μM TTX (blue) completely abolished Na+ currents at −30 mV in MCs. (F) Quantification of peak amplitudes recorded from MCs at different membrane potentials n = 4; ANOVA, F = 45.71, p < 0.001; Bonferroni multiple comparison test, **p ≤ 0.01, ***p ≤ 0.001). Data used in the generation of this figure can be found in S1 Data. GC, granule cell; MC, mitral cell; TEA, tetraethylammonium; TTX, tetrodotoxin. (TIF) [file pbio.2003816.s003.tif]

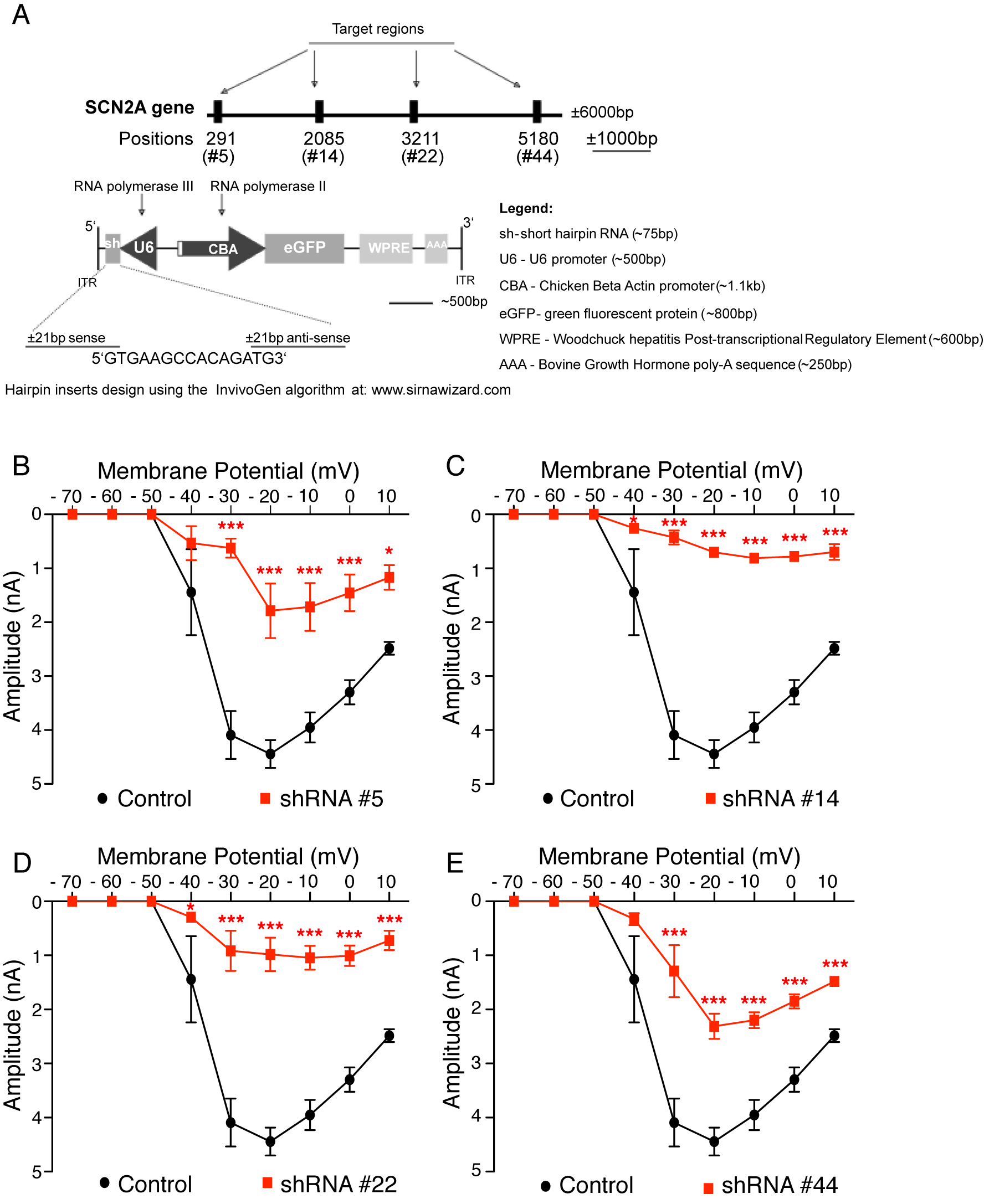

Supplement: S4 Fig — (A) shRNAs were designed using the InvivoGen Wizard (www.sirnawizard.com). Four suitable target sequences were identified on the SCN2A mRNA. rAAV1/2 vectors mediating shRNA expression driven by the U6 promotor and GFP expression from the CBA promoter. rAAV was injected into the OB (see Materials and methods). (B-E) Voltage-clamp recordings were established from transduced and control GCs in 300-μm-thick OB slices at 34 ± 1 °C. Series of voltage square pulses ranging from −70 mV to +10 mV per step, with 5-ms duration, were applied to assess the amplitude of Na+ currents in each pulse tested. Four shRNA molecules were tested (B-E), and each affected the Na+ current differently. (B) The shRNA#5 targeted nucleotides 291–312 and reduced the Na+ current by approximately 60% compared to control. (C) The shRNA#14 targeted nucleotides 2085–2106 and reduced the Na+ current by approximately 90% relative to control. (D) The shRNA#22 targeted nucleotides 3211–3232 and reduced the Na+ current by approximately 75% relative to control. (E) The shRNA#44 targeted nucleotides 5180–5201 and reduced the Na+ current by approximately 45% compared to control. Data used in the generation of this figure can be found in S1 Data. CBA, chicken beta actin; GC, granule cell; GFP, green fluorescent protein; OB, olfactory bulb; rAAV, recombinant adeno-associated virus; shRNA, short hairpin RNA. (TIF) [file pbio.2003816.s004.tif]

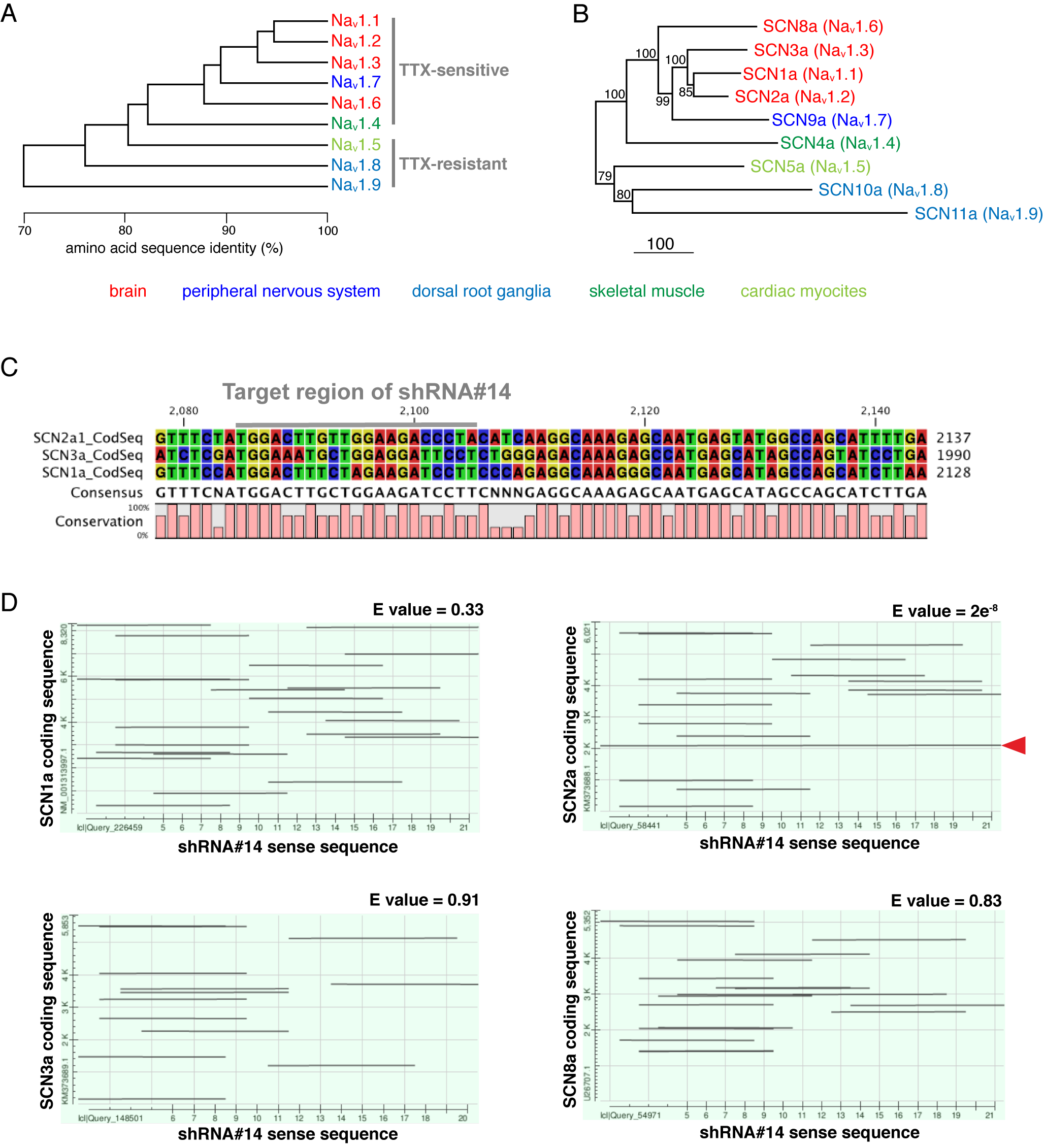

Supplement: S5 Fig — (A) Amino acid sequence similarity among the Nav1.x subtypes. Sequences were aligned in the CLC sequence viewer software, using the UPGMA algorithm. The color code depicts the most prominent expression location as indicated in the color legend. The resistance of each subtype to TTX is indicated gray. (B) Phylogenetic relationship of the mouse VGSCs α-subunits. The published nucleotide sequences in PubMed Central were aligned using the neighbor-joining algorithm, with a gap open cost of 7, gap extension cost of 3, and gap end was free. Bootstrapping analysis was performed, and the values shown in the ramification branches of the tree represent the number of replications. The tree was rooted using a VGSC expressed in Drosophila melanogaster (not shown). The scale bar represents 100 nucleotide substitutions. The tree was generated using the CLC sequence viewer software. (C) Coding sequence similarities among the most similar NaV1.x brain subtypes with the gray line delineating the target region of shRNA#14. The sequence similarity is 62% among NaV1.1 and NaV 1.3 and 76% between NaV1.1 and NaV 1.2. (D) The shRNA#14 is highly specific for SCN2a. BLAST of the 21-nucleotide sense sequence of shRNA#14 against the sequences of the VGSCs brain subunits demonstrates that this molecule has very low probabilities to knock down SCN1a, SCN3a, and SCN8a (E value > 0.1) at any location within the coding region of the VGSCs subunits (left side of the panels). The shRNA#14 shows a region in the SCN2a gene with 100% similarity (red arrow), so that the E value is very low for this region (2e-8), indicating high probability of knockdown of the Nav1.2 subunit. shRNA, short hairpin RNA; TTX, tetrodotoxin; UPGMA, unweighted pair group method with arithmetic mean; VGSC, voltage-gated sodium channel. (TIF) [file pbio.2003816.s005.tif]

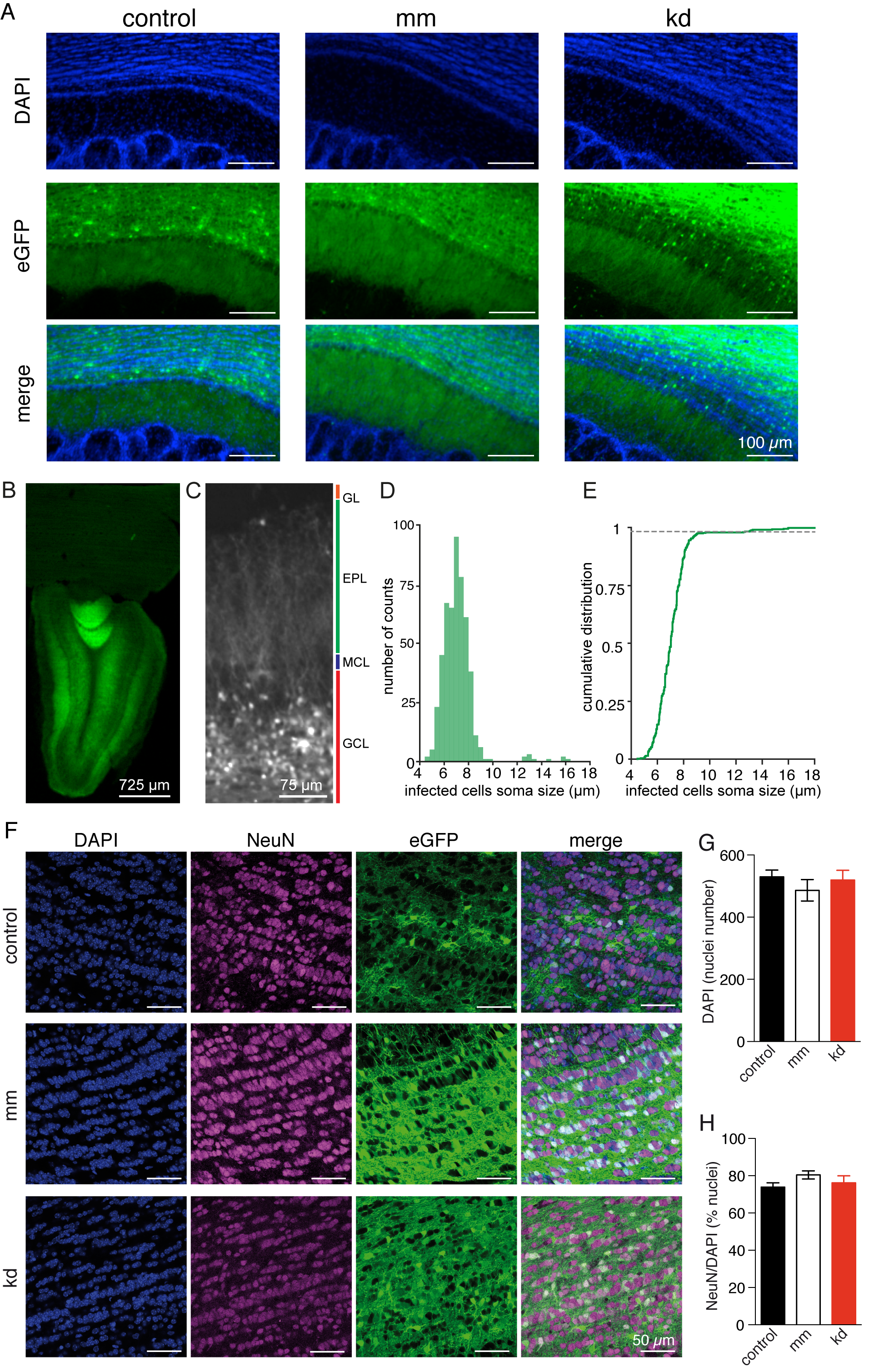

Supplement: S6 Fig — (A) rAAV1/2 particles were delivered into the GCL of the OB, and spread of infection was assessed by imaging eGFP, rAAV-mm#14//eGFP (mm), and rAAV-shRNA#14//eGFP (kd). All images were acquired with a wide-field fluorescence microscope. DAPI staining identifies OB layers (top-down: GCL, MCL, EPL, GL). (B) Low-resolution wide-field epifluorescence image of a typical injection directed to the GCL of the OB with GFP expression in the GC domain of the OB (somas located in the GCL, dendrites that transverse the GCL toward the EPL and dendritic arborizations very prominent in the EPL). No fluorescence is observed in cortical and subcortical regions. (C) Maximal projection of a confocal image stack showing GFP expression in the GCL (10–15 consecutive stacks; n = 3 preparations from one mouse each); 3 fields of 200 μm × 800 μm per preparation were chosen randomly to determine the dimensions of GFP-expressing cells. (D) Histogram showing the distribution of the soma diameters of GFP-expressing cells in the GCL. A total of 510 cells were analyzed. According to Nagayama and colleagues [39], GCs are the only cells that extend their dendrites into the EPL, as was the case for all GCs analyzed. Furthermore, GCs have much smaller somas (6–8 μm) than dSA cells (10–20 μm). (E) Cumulative distribution of the data shown in C; 99% of the infected cells have a soma diameter of less than 10 μm and hence can be classified as GCs. (F) Representative confocal image stacks (MIP of 10 image frames) of OB horizontal sections of mice stereotaxically injected with rAAV-eGFP (“control,” n = 4 mice), rAAV-mmshRNA#14 (“mm,” n = 6 mice), or rAAV-shRNA#14 (“kd,” n = 6 mice). DAPI-stained nuclei (blue), anti-NeuN antibody labels neuronal nuclei (magenta), and GFP reveals transduced GCs (green). (G-H) Nuclei were counted in OB horizontal sections for each labeling condition. From each OB analyzed, 5 stacks of 10 image frames each were taken from random regions of the GCL. (C) Total number of nuclei: con [file pbio.2003816.s006.tif]

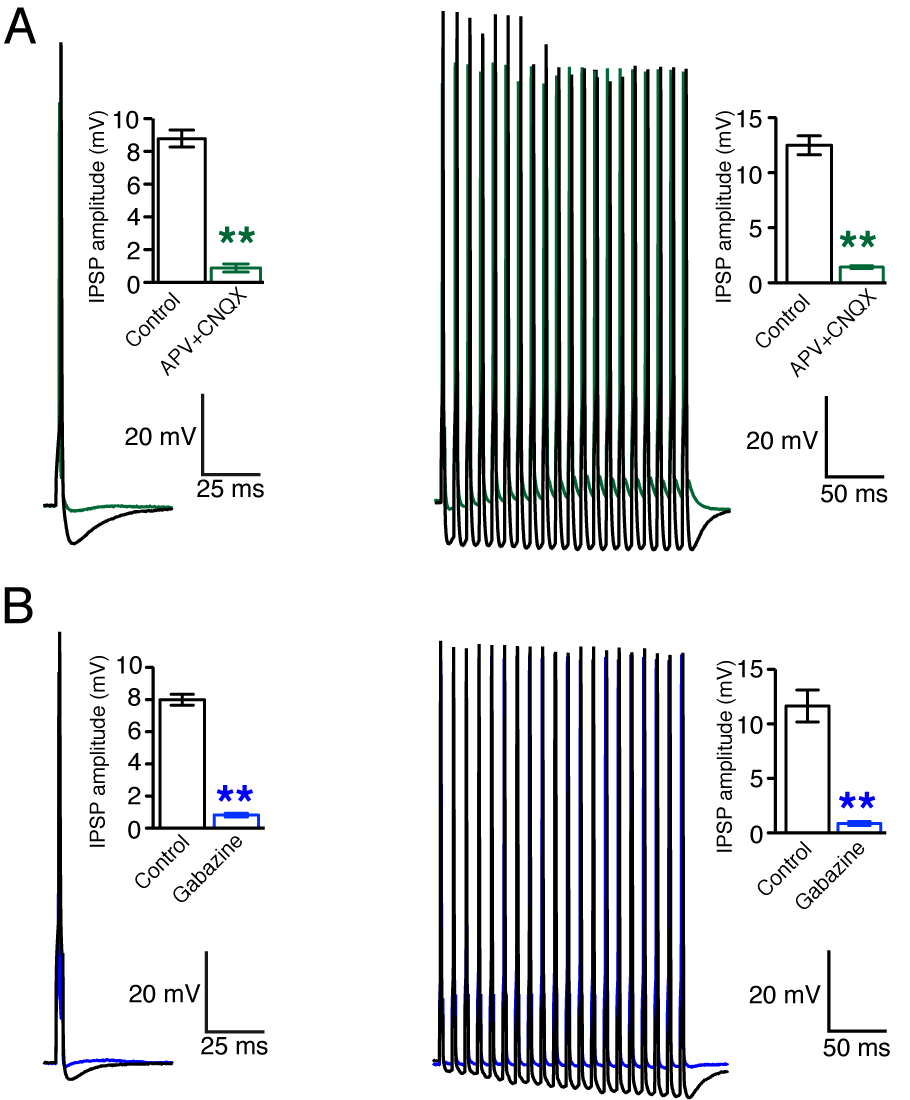

Supplement: S7 Fig — (A) Single action potentials (left) or 20 consecutive action potentials at 100 Hz (right) were evoked in MCs (n = 3) through somatic current injections. Recordings were made at 34 ± 1 °C. Black traces represent control recordings in bath solution; green traces represent recordings in bath solution supplemented with 10 μM CNQX and 50 μM APV. The hyperpolarization was nearly abolished upon a 25-minute bath application of the drugs (single action potential: 0.88 ± 0.25 mV; 100 Hz: 1.43 ± 0. 13 mV) in comparison to the control situation (single action potential: 8.79 ± 0.52 mV; 100 Hz: 12.50 ± 0.87 mV; Student t test, p = 0.009 and p = 0.008, respectively). (B) As in (A) but using 20 μM Gabazine (blue). The rIPSP amplitude was significantly reduced upon a 25-minute bath application of Gabazine (single action potential: 0.83 ± 0.11 mV; 100 Hz: 0.88 ± 0.20 mV) in comparison to control conditions (single action potential: 8.00 ± 0.33 mV; 100 Hz: 11.65 ± 1.47 mV; Student t test, p = 0.002 in both situations). Data used in the generation of this figure can be found in S1 Data. AMPA, α-amino-3-hydroxy-5-methyl-4-isoxazolepropionic acid; APV, 2-amino-5-phosphonopentanoic acid; CNQX, 6-cyano-7- nitroquinoxaline-2,3-dione; GABA, gamma-aminobutyric acid; MC, mitral cell; NMDA, N-methyl-D-aspartate; rIPSP, recurrent inhibitory postsynaptic potential. (TIF) [file pbio.2003816.s007.tif]

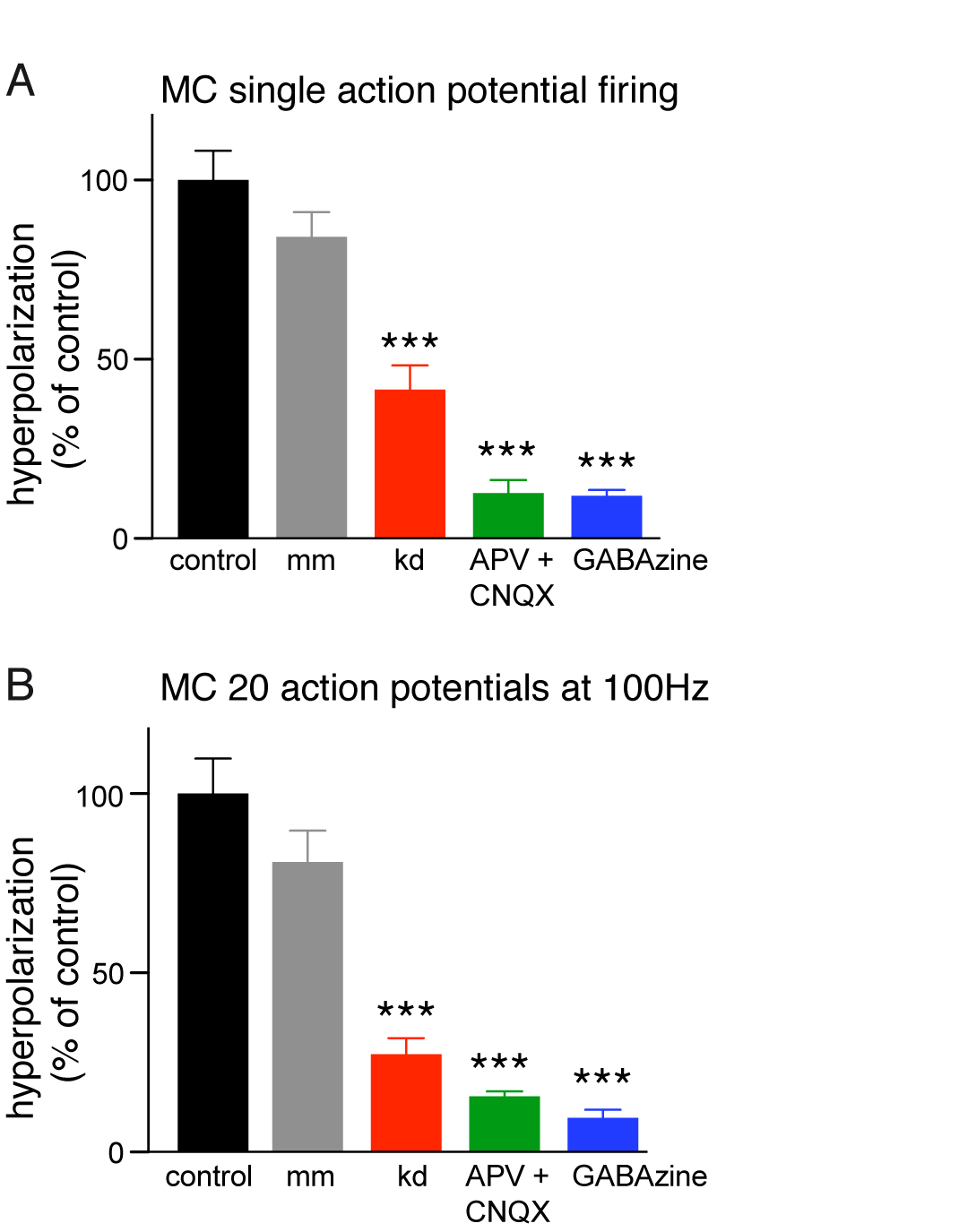

Supplement: S8 Fig — Quantitative comparison of NaV1.2 knockdown and pharmacological blockade of dendrodendritic communication based on the data shown in Fig 5 and S7 Fig. Glutamate receptor and GABAA receptor blockade reduced the hyperpolarization by 90%. Assuming a close to complete block of all glutamate receptors and GABAA receptors of the dendrodendritic synapse, the remaining 10% of the hyperpolarization could be attributed to a nonsynaptic mechanism such as the AHP [58]. NaV1.2 knockdown reduced the hyperpolarization amplitude by 60%–75%. The less pronounced block caused by the genetic manipulation can be explained by the incomplete transduction of the GC population (Fig 4). MCs receive gemmules of GCs from a mixed population: GCs with a reduced number of NaV1.2 and unperturbed GCs with a normal complement of NaV1.2 channels. As we have demonstrated above (Fig 4, S6 Fig), our genetic perturbation does not affect MCs and hence cannot be attributed to any MC-intrinsic mechanisms. Furthermore, phrixotoxin-3 did not have an effect on voltage-gated conductances in MCs (S3D–S3F Fig), indicating that even NaV1.2 knockdown in MCs would not affect action potential firing. Taken together, the hyperpolarization determined after NaV1.2 knockdown includes nonsynaptic components such as the AHP or IH but mostly reflects the rIPSP. Subtracting these approximately 10% nonsynaptic contributions from control and knockdown conditions would yield a reduction of the rIPSP by approximately 70%–80%. (A) Single MC action potential. ANOVA, F = 17.25, p < 0.0001. *** denotes highly significant difference relative to control and mm. No significant difference was found when comparing kd, APV+CNQX, and GABAzine conditions or when comparing control and mm. (B) Twenty consecutive action potentials at 100 Hz elicited in MCs. ANOVA, F = 18.02, p < 0.0001. *** denotes highly significant difference relative to control and mm. No significant difference was found when comparing kd, APV+CNQX, and GABAzine conditions [file pbio.2003816.s008.tif]
