# Supplementary material for: Axonal sodium channel NaV1.2 drives granule cell dendritic GABA release and rapid odor discrimination
Source: PLoS Biol. 2018 Aug 20;16(8):e2003816. doi: 10.1371/journal.pbio.2003816 (PMC6117082; doi:10.1371/journal.pbio.2003816)
Supplement: S1 File — This compilation includes code for electrophysiology, behavior data acquisition, and behavior data analysis in separate chapters. All supporting software was written in Igor Pro6 (Wavemetrics). (PDF) [file pbio.2003816.s010.pdf]

This file contains all custom-made software written in Igor Pro (Wavemetrics) to:

1 – Analysis of whole-cell voltage clamp experiments in GCs and MCs (pages 1 to 4)
2 – Analysis of somatic current injections in MCs (pages 5 to 19)
3 – Analysis of behavior data (pages 20 to 139)
4 – Behavior experiment: training template (pages 140-166)
5 – Behavior experiment: pre-training template (pages 167 - 192)

1 – Analysis of whole-cell voltage clamp experiments in GCs and MCs

```
#pragma rtGlobals=1		// Use modern global access method.
#include  <Append Calibrator>


macro Beggin_Analysis (How_many_waves)
variable How_many_waves

string wana, ws
variable depolarization, current_baseline, peak, x_value, peakpn, amplitude i, j,
amplitude_result
make /O/n=9 Save_results_1
i=0
depolarization=-70

silent 1

	do
		ws = wavelist("*",";","")
		wana =		StringFromList(i,ws)
		duplicate /O $wana, Trace
		Print "..............................................................................................."
		Print "Analysis for Sodium Current Amplitude at "+num2str(depolarization)+"
mV"
		Print "Wave analysed",wana
Wavestats /Q/M=1/R=[0,1000] Trace
current_baseline=v_avg
Print "The baseline is: "+num2str(current_baseline*1e12)+" pA"

//peak detection
Wavestats /Q /M=1 /R=[1000,1500] Trace
peak=V_min
x_value=v_minloc
Findvalue /T=1e-12 /v=(peak) Trace
peakpn=v_value
//Cursor /A=1 /P /C=(3,52428,1)/S=0 A, Trace, peakpn
Print "Peak was detected at: "+num2str(x_value*1000)+" ms, at point: "+num2str(peakpn)
```

```
//current amplitude
amplitude=peak-current_baseline
amplitude_result=-amplitude*1e9
save_results_1[i]=amplitude_result
Print "Amplitude is: "+num2str(amplitude*1e9)+" nA"
                i+=1
                depolarization+=10
                sleep 00:00:01
        while ( i<How_many_waves)
        edit save_results_1
endmacro

Macro Current_Analysis()

silent 1

//baseline definition
Wavestats /Q/M=1/R=[0,1000] Trace
variable current_baseline=v_avg
Print "The baseline is: "+num2str(current_baseline*1e12)+" pA"
Display trace

//peak detection
Wavestats /Q /M=1 Trace
variable peak=V_min
variable x_value=v_minloc
Findvalue /T=1e-12 /v=(peak) Trace
variable peakpn=v_value
Cursor /A=1 /P /C=(3,52428,1)/S=0 A, Trace, peakpn
Print "Peak was detected at: "+num2str(x_value*1000)+" ms, at point: "+num2str(peakpn)

//current amplitude
variable amplitude=peak-current_baseline
Print "Amplitude is: "+num2str(amplitude*1e9)+" nA"

Endmacro


Macro Analysis_with_Artifact_Removal(How_many_waves)
variable how_many_waves
//artifact removal - P/N subtraction
        //using only one current response to a 10 mV voltage pulse - second pulse (from -
70mV or hold to -60 mV )
        // pulse is then summated to the same amplitude of the pulse current in analysis
        // at the end, the summated pulse is subtracted from the current response to be
analysed

variable i=0, correction
variable multiplicate=1, depolarization=-70
variable current_baseline, peak, x_value, peakpn, amplitude, amplitude_result
```

```
string ws, wl, pulse, wana
string w_event
silent 1
make /O/n=9 Save_results

Print "..............................................................................."
ws=wavelist("*",";","")
wana=stringfromlist(i,ws)
Print "Wave analysed", wana
duplicate/O $wana, Trace
Print "Analysis for Sodium Current Amplitude at "+num2str(depolarization)+" mV"
wl=wavelist("*",";","")
pulse= StringFromList(0,wl)
duplicate /O $pulse, PN_Sub
PN_sub=PN_sub*-multiplicate

duplicate /O Trace, Result
Result[0,2999]=Result[p]+pn_sub[p]
correction=Result[0]-trace[0]
Result[0,2499]=Result[p]-correction

//baseline definition
Wavestats /Q/M=1/R=[0,1000] Result
current_baseline=v_avg
Print "The baseline is: "+num2str(current_baseline*1e12)+" pA"


//peak detection
Wavestats /Q /M=1 Result
peak=V_min
x_value=v_minloc
Findvalue /T=1e-12 /v=(peak) Result
peakpn=v_value
//dowindow result
//Cursor /A=1 /P /C=(3,52428,1)/S=0 A, Result, peakpn
Print "Peak was detected at: "+num2str(x_value*1000)+" ms, at point: "+num2str(peakpn)

//current amplitude
amplitude=peak-current_baseline
amplitude_result=-amplitude*1e9
Print "Amplitude is: "+num2str(amplitude*1e9)+" nA"
sleep 00:00:01
save_results[i]=amplitude_result
multiplicate+=1
depolarization+=10
i+=1


do
Print "..............................................................................."
```

```
ws=wavelist("*",";","")
wana=stringfromlist(i,ws)
Print "Wave analysed", wana
duplicate/O $wana, Trace
Print "Analysis for Sodium Current Amplitude at "+num2str(depolarization)+" mV"
wl=wavelist("*",";","")
pulse= StringFromList(1,wl)
duplicate /O $pulse, PN_Sub
PN_sub[0,2499]=PN_sub[p]*-1*multiplicate
duplicate /O Trace, Result
Result[0,2499]=Result[p]+pn_sub[p]
correction=Result[0]-trace[0]
Result[0,2499]=Result[p]-correction

w_event="trace"+num2str(i)
make /O/n=2499 $w_event
$W_event=result


//baseline definition
Wavestats /Q/M=1/R=[0,1000] Result
current_baseline=v_avg
Print "The baseline is: "+num2str(current_baseline*1e12)+" pA"


//peak detection
Wavestats /Q /M=1 Result
peak=V_min
x_value=v_minloc
Findvalue /T=1e-12 /v=(peak) Result
peakpn=v_value
//dowindow result
//Cursor /A=1 /P /C=(3,52428,1)/S=0 A, Result, peakpn
Print "Peak was detected at: "+num2str(x_value*1000)+" ms, at point: "+num2str(peakpn)

//current amplitude
amplitude=peak-current_baseline
amplitude_result=-amplitude*1e9
Print "Amplitude is: "+num2str(amplitude*1e9)+" nA"
sleep 00:00:01
save_results[i]=amplitude_result
multiplicate+=1
depolarization+=10
i+=1
while (i<how_many_waves)
edit save_results

EndMacro
```

2 – Analysis of somatic current injections in MCs

```
#pragma rtGlobals=1                    // Use modern global access method.

Macro Initialize()

string ws, wanna, wannan
variable /g startx, endx
variable i

make /O /N=1 Baseline, IPSPpeak, IPSPamplitude, IPSPtau, FitAsymptote
make /O/T /N=20 APnumber
make /O/N=20 amplitudes

ws=wavelist("*",";","")
wanna=stringfromlist(0,ws)

wannan=wanna+num2istr(i)

duplicate /O $wanna, Trace
display Trace

Endmacro


Macro IPSP_analysis_1AP()

Initialize()

Print "..........................................................................................."
Print "IPSP analysis for 1 AP"

Duplicate /O/R=[4500,6000] Trace, IPSP
display IPSP; delayupdate
modifygraph  rgb=(0,0,0)

silent 1

//baselline definition
wavestats /Q/M=1/R=[0,1000] Trace
variable IPSP_baseline=v_avg
print "The resting membrane potential (baseline) is: "+num2str(ipsp_baseline*1000)+" mV"
Baseline[]=ipsp_baseline*1000

//peak detection
wavestats /Q /M=1 IPSP
variable peak=V_min
variable x_value=v_minloc
findvalue /v=(peak) IPSP
```

```
variable peakpn=v_value
cursor /A=1 /P /C=(3,52428,1)/S=0 A, IPSP, peakpn
print "Peak was detected at: "+num2str(x_value*1000)+" ms, at point: "+num2str(peakpn)
print "Membrane potential at peak is:"+num2str(peak*1000)+" mV"
IPSPpeak[]=peak*1000

//ipsp amplitude
variable ipsp_amplitude=peak-ipsp_baseline
print "IPSP amplitude is: "+num2str(ipsp_amplitude*1000)+" mV"
IPSPamplitude=ipsp_amplitude*1000

//decay time fit from 90% to 10%peak
variable ninety=ipsp_baseline+ipsp_amplitude*0.9
variable endfit=ipsp_baseline+ipsp_amplitude*0.1
findlevel /Q /R=[peakpn] IPSP, endfit
variable endfity=v_levelx
findlevel /Q/R=[peakpn] IPSP, ninety
variable startfity=V_levelX
curvefit /Q  exp_XOffset IPSP(startfity, endfity) /A/D
variable ipsp_Tau=w_coef(2)
variable asympt=W_coef(0)
print "Tau is: "+num2str(ipsp_tau*1000)+" ms"
print "The fitted trace asymptotes to: "+num2str(asympt*1000)+" mV"
IPSPtau=ipsp_tau*1000
FitAsymptote=asympt*1000

Endmacro


Macro IPSP_analysis_5APs()

Initialize()

Print "...................................................................................."
Print "IPSP analysis for 5 APs"

duplicate /O/R=[5450,10000] Trace, IPSP
display IPSP; delayupdate
modifygraph  rgb=(0,0,0)

//baseline definition
wavestats /Q/M=1/R=[2000,3000] Trace
variable ipsp_baseline=v_avg
print "The resting membrane potential (baseline) is: "+num2str(ipsp_baseline*1000)+" mV"
Baseline[]=ipsp_baseline*1000

//peak detection
wavestats /Q /M=1 IPSP
variable peak=V_min
variable x_value=v_minloc
```

```
findvalue /v=(peak) IPSP
variable peakpn=v_value
cursor /A=1 /P /C=(3,52428,1)/S=0 A, IPSP, peakpn
print "Peak ist at: "+num2str(peak*1000)+" mV"
print "Peak was detected at: "+num2str(x_value*1000)+" ms, at point: "+num2str(peakpn)
print "Membrane potential at peak is:"+num2str(peak*1000)+" mV"
IPSPpeak[]=peak*1000

//ipsc amplitude
variable ipsp_amplitude=peak-ipsp_baseline
print "IPSP amplitude is: "+num2str(ipsp_amplitude*1000)+" mV"
IPSPamplitude=ipsp_amplitude*1000

//decay time fit from 90%peak to 10%peak
variable ninety=ipsp_baseline+ipsp_amplitude*0.9
variable endfit=ipsp_baseline+ipsp_amplitude*0.1
findlevel /Q/R=[peakpn] IPSP, endfit
variable endfity=V_levelX
findlevel /Q/R=[peakpn] IPSP, ninety
variable startfity=V_levelX
curvefit  /Q  exp_XOffset IPSP(startfity, endfity) /A/D
variable ipsp_Tau=w_coef(2)
variable asympt=W_coef(0)
print "Tau is: "+num2str(ipsp_tau*1000)+" ms"
print "The fitted trace asymptotes to: "+num2str(asympt*1000)+" mV"
IPSPtau=ipsp_tau*1000
FitAsymptote=asympt*1000

Endmacro


Macro IPSP_analysis_20APs()

Initialize()

Print "..........................................................................................."
Print "IPSP analysis for 20 APs"

//wave duplication from end of stimulus to end of trace
duplicate /O/R=[7100,15000]Trace, IPSP
display IPSP; delayupdate
modifygraph  rgb=(0,0,0)

//baseline definition
wavestats /Q/M=1/R=[2000,3000] Trace
variable ipsp_baseline=v_avg
print "The resting membrane potential (baseline) is: "+num2str(ipsp_baseline*1000)+" mV"
Baseline[]=ipsp_baseline*1000

//peak detection
```

```
wavestats /Q /M=1 IPSP
variable peak=V_min
variable x_value=v_minloc
findvalue /v=(peak) IPSP
variable peakpn=v_value
cursor /A=1 /P /C=(3,52428,1)/S=0 A, IPSP, peakpn
print "Peak ist at: "+num2str(peak*1000)+" mV"
print "Peak was detected at: "+num2str(x_value*1000)+" ms, at point: "+num2str(peakpn)
print "Membrane potential at peak is:"+num2str(peak*1000)+" mV"
IPSPpeak[]=peak*1000

//ipsc amplitude
variable ipsp_amplitude=peak-ipsp_baseline
print "IPSP amplitude is: "+num2str(ipsp_amplitude*1000)+" mV"
IPSPamplitude=ipsp_amplitude*1000

//decay time fit from 90%peak to 10%peak
variable ninety=ipsp_baseline+ipsp_amplitude*0.9
variable endfit=ipsp_baseline+ipsp_amplitude*0.1
findlevel /Q/R=[peakpn] IPSP, endfit
variable endfity=V_levelX
findlevel /Q/R=[peakpn] IPSP, ninety
variable startfity=V_levelX
curvefit  /Q  exp_Xoffset IPSP(startfity, endfity) /A/D
variable ipsp_Tau=w_coef(2)
variable asympt=W_coef(0)
print "Tau is: "+num2str(ipsp_tau*1000)+" ms"
print "The fitted trace asymptotes to: "+num2str(asympt*1000)+" mV"
IPSPtau=ipsp_tau*1000
FitAsymptote=asympt*1000

EndMacro

Macro Inter_AP_IPSP()

Initialize()

Print "..............................................................................."
Print "IPSP analysis for 20 APs"

// wave duplications to find peaks in between APs
Duplicate /O/R=[5030, 5120] Trace, IPSP_1
Duplicate /O/R=[5140, 5230] Trace, IPSP_2
Duplicate /O/R=[5250, 5340] Trace, IPSP_3
Duplicate /O/R=[5360, 5450] Trace, IPSP_4
Duplicate /O/R=[5470, 5560] Trace, IPSP_5
Duplicate /O/R=[5580, 5670] Trace, IPSP_6
Duplicate /O/R=[5690, 5780] Trace, IPSP_7
Duplicate /O/R=[5800, 5890] Trace, IPSP_8
Duplicate /O/R=[5910, 6000] Trace, IPSP_9
```

```
Duplicate /O/R=[6020, 6110] Trace, IPSP_10
Duplicate /O/R=[6130, 6220] Trace, IPSP_11
Duplicate /O/R=[6240, 6330] Trace, IPSP_12
Duplicate /O/R=[6350, 6440] Trace, IPSP_13
Duplicate /O/R=[6460, 6550] Trace, IPSP_14
Duplicate /O/R=[6570, 6660] Trace, IPSP_15
Duplicate /O/R=[6680, 6770] Trace, IPSP_16
Duplicate /O/R=[6790, 6880] Trace, IPSP_17
Duplicate /O/R=[6900, 6990] Trace, IPSP_18
Duplicate /O/R=[7010, 7100] Trace, IPSP_19
Duplicate /O/R=[7120, 7210] Trace, IPSP_20

display IPSP_1
appendtograph
IPSP_2,IPSP_3,IPSP_4,IPSP_5,IPSP_6,IPSP_7,IPSP_8,IPSP_9,IPSP_10
appendtograph
IPSP_11,IPSP_12,IPSP_13,IPSP_14,IPSP_15,IPSP_16,IPSP_17,IPSP_18,IPSP_19,
IPSP_20



//baseline definition
wavestats /Q/M=1/R=[2000,3000] Trace
variable ipsp_baseline=v_avg
print "The resting membrane potential (baseline) is: "+num2str(ipsp_baseline*1000)+" mV"
Baseline[]=ipsp_baseline*1000

//peak detection_IPSP_1
wavestats /Q /M=1 IPSP_1
variable peak_1=V_min
variable x_value_1=v_minloc
findvalue /v=(peak_1) IPSP_1
variable peakpn_1=v_value
cursor /A=1 /P /C=(3,52428,1)/S=0 A, IPSP_1, peakpn_1
print "Peak ist at: "+num2str(peak_1*1000)+" mV"
print "Peak was detected at: "+num2str(x_value_1*1000)+" ms, at point:
"+num2str(peakpn_1)
print "Membrane potential at peak is:"+num2str(peak_1*1000)+" mV"
// amplitude IPSP_1
variable ipsp_amplitude_1=peak_1-ipsp_baseline
print "IPSP amplitude is: "+num2str(ipsp_amplitude_1*1000)+" mV"
variable IPSPamplitude_1=ipsp_amplitude_1*1000
APnumber[0]="IPSP_1"
amplitudes[0]=IPSPamplitude_1
silent 1

Print "..................................................................................."
//peak detection_IPSP_2
wavestats /Q /M=1 IPSP_2
variable peak_2=V_min
```

```
variable x_value_2=v_minloc
findvalue /v=(peak_2) IPSP_2
variable peakpn_2=v_value
cursor /A=1 /P /C=(3,52428,1)/S=0 A, IPSP_2, peakpn_2
print "Peak ist at: "+num2str(peak_2*1000)+" mV"
print "Peak was detected at: "+num2str(x_value_2*1000)+" ms, at point:
"+num2str(peakpn_2)
print "Membrane potential at peak is:"+num2str(peak_2*1000)+" mV"
// amplitude IPSP_2
variable ipsp_amplitude_2=peak_2-ipsp_baseline
print "IPSP amplitude is: "+num2str(ipsp_amplitude_2*1000)+" mV"
variable IPSPamplitude_2=ipsp_amplitude_2*1000
APnumber[1]="IPSP_2"
amplitudes[1]=IPSPamplitude_2
silent 1

Print "..........................................................................................."
//peak detection_IPSP_3
wavestats /Q /M=1 IPSP_3
variable peak_3=V_min
variable x_value_3=v_minloc
findvalue /v=(peak_3) IPSP_3
variable peakpn_3=v_value
cursor /A=1 /P /C=(3,52428,1)/S=0 A, IPSP_3, peakpn_3
print "Peak ist at: "+num2str(peak_3*1000)+" mV"
print "Peak was detected at: "+num2str(x_value_3*1000)+" ms, at point:
"+num2str(peakpn_3)
print "Membrane potential at peak is:"+num2str(peak_3*1000)+" mV"
// amplitude IPSP_3
variable ipsp_amplitude_3=peak_3-ipsp_baseline
print "IPSP amplitude is: "+num2str(ipsp_amplitude_3*1000)+" mV"
variable IPSPamplitude_3=ipsp_amplitude_3*1000
APnumber[2]="IPSP_3"
amplitudes[2]=IPSPamplitude_3
silent 1

Print "..........................................................................................."
//peak detection_IPSP_4
wavestats /Q /M=1 IPSP_4
variable peak_4=V_min
variable x_value_4=v_minloc
findvalue /v=(peak_4) IPSP_4
variable peakpn_4=v_value
cursor /A=1 /P /C=(3,52428,1)/S=0 A, IPSP_4, peakpn_4
print "Peak ist at: "+num2str(peak_4*1000)+" mV"
print "Peak was detected at: "+num2str(x_value_4*1000)+" ms, at point:
"+num2str(peakpn_4)
print "Membrane potential at peak is:"+num2str(peak_4*1000)+" mV"
// amplitude IPSP_4
variable ipsp_amplitude_4=peak_4-ipsp_baseline
```

```
print "IPSP amplitude is: "+num2str(ipsp_amplitude_4*1000)+" mV"
variable IPSPamplitude_4=ipsp_amplitude_4*1000
APnumber[3]="IPSP_3"
amplitudes[3]=IPSPamplitude_4
silent 1

Print "..............................................................................."
//peak detection_IPSP_5
wavestats /Q /M=1 IPSP_5
variable peak_5=V_min
variable x_value_5=v_minloc
findvalue /v=(peak_5) IPSP_5
variable peakpn_5=v_value
cursor /A=1 /P /C=(3,52428,1)/S=0 A, IPSP_5, peakpn_5
print "Peak ist at: "+num2str(peak_5*1000)+" mV"
print "Peak was detected at: "+num2str(x_value_5*1000)+" ms, at point:
"+num2str(peakpn_5)
print "Membrane potential at peak is:"+num2str(peak_5*1000)+" mV"
// amplitude IPSP_5
variable ipsp_amplitude_5=peak_5-ipsp_baseline
print "IPSP amplitude is: "+num2str(ipsp_amplitude_5*1000)+" mV"
variable IPSPamplitude_5=ipsp_amplitude_5*1000
APnumber[4]="IPSP_5"
amplitudes[4]=IPSPamplitude_5
silent 1

Print "..............................................................................."
//peak detection_IPSP_6
wavestats /Q /M=1 IPSP_6
variable peak_6=V_min
variable x_value_6=v_minloc
findvalue /v=(peak_6) IPSP_6
variable peakpn_6=v_value
cursor /A=1 /P /C=(3,52428,1)/S=0 A, IPSP_6, peakpn_6
print "Peak ist at: "+num2str(peak_6*1000)+" mV"
print "Peak was detected at: "+num2str(x_value_6*1000)+" ms, at point:
"+num2str(peakpn_6)
print "Membrane potential at peak is:"+num2str(peak_6*1000)+" mV"
// amplitude IPSP_6
variable ipsp_amplitude_6=peak_6-ipsp_baseline
print "IPSP amplitude is: "+num2str(ipsp_amplitude_6*1000)+" mV"
variable IPSPamplitude_6=ipsp_amplitude_6*1000
APnumber[5]="IPSP_6"
amplitudes[5]=IPSPamplitude_6
silent 1

Print "..............................................................................."
//peak detection_IPSP_7
wavestats /Q /M=1 IPSP_7
variable peak_7=V_min
```

```
variable x_value_7=v_minloc
findvalue /v=(peak_7) IPSP_7
variable peakpn_7=v_value
cursor /A=1 /P /C=(3,52428,1)/S=0 A, IPSP_7, peakpn_7
print "Peak ist at: "+num2str(peak_7*1000)+" mV"
print "Peak was detected at: "+num2str(x_value_7*1000)+" ms, at point:
"+num2str(peakpn_7)
print "Membrane potential at peak is:"+num2str(peak_7*1000)+" mV"
// amplitude IPSP_7
variable ipsp_amplitude_7=peak_7-ipsp_baseline
print "IPSP amplitude is: "+num2str(ipsp_amplitude_7*1000)+" mV"
variable IPSPamplitude_7=ipsp_amplitude_7*1000
APnumber[6]="IPSP_7"
amplitudes[6]=IPSPamplitude_7
silent 1

Print "..........................................................................................."
//peak detection_IPSP_8
wavestats /Q /M=1 IPSP_8
variable peak_8=V_min
variable x_value_8=v_minloc
findvalue /v=(peak_8) IPSP_8
variable peakpn_8=v_value
cursor /A=1 /P /C=(3,52428,1)/S=0 A, IPSP_8, peakpn_8
print "Peak ist at: "+num2str(peak_8*1000)+" mV"
print "Peak was detected at: "+num2str(x_value_8*1000)+" ms, at point:
"+num2str(peakpn_8)
print "Membrane potential at peak is:"+num2str(peak_8*1000)+" mV"
// amplitude IPSP_8
variable ipsp_amplitude_8=peak_8-ipsp_baseline
print "IPSP amplitude is: "+num2str(ipsp_amplitude_8*1000)+" mV"
variable IPSPamplitude_8=ipsp_amplitude_8*1000
APnumber[7]="IPSP_8"
amplitudes[7]=IPSPamplitude_8
silent 1

Print "..........................................................................................."
//peak detection_IPSP_9
wavestats /Q /M=1 IPSP_9
variable peak_9=V_min
variable x_value_9=v_minloc
findvalue /v=(peak_9) IPSP_9
variable peakpn_9=v_value
cursor /A=1 /P /C=(3,52428,1)/S=0 A, IPSP_9, peakpn_9
print "Peak ist at: "+num2str(peak_9*1000)+" mV"
print "Peak was detected at: "+num2str(x_value_9*1000)+" ms, at point:
"+num2str(peakpn_9)
print "Membrane potential at peak is:"+num2str(peak_9*1000)+" mV"
// amplitude IPSP_9
variable ipsp_amplitude_9=peak_9-ipsp_baseline
```

```
print "IPSP amplitude is: "+num2str(ipsp_amplitude_9*1000)+" mV"
variable IPSPamplitude_9=ipsp_amplitude_9*1000
APnumber[8]="IPSP_9"
amplitudes[8]=IPSPamplitude_9
silent 1

Print "..........................................................................................."
//peak detection_IPSP_10
wavestats /Q /M=1 IPSP_10
variable peak_10=V_min
variable x_value_10=v_minloc
findvalue /v=(peak_10) IPSP_10
variable peakpn_10=v_value
cursor /A=1 /P /C=(3,52428,1)/S=0 A, IPSP_10, peakpn_10
print "Peak ist at: "+num2str(peak_10*1000)+" mV"
print "Peak was detected at: "+num2str(x_value_10*1000)+" ms, at point:
"+num2str(peakpn_10)
print "Membrane potential at peak is:"+num2str(peak_10*1000)+" mV"
// amplitude IPSP_10
variable ipsp_amplitude_10=peak_10-ipsp_baseline
print "IPSP amplitude is: "+num2str(ipsp_amplitude_10*1000)+" mV"
variable IPSPamplitude_10=ipsp_amplitude_10*1000
APnumber[9]="IPSP_10"
amplitudes[9]=IPSPamplitude_10
silent 1

Print "..........................................................................................."
//peak detection_IPSP_11
wavestats /Q /M=1 IPSP_11
variable peak_11=V_min
variable x_value_11=v_minloc
findvalue /v=(peak_11) IPSP_11
variable peakpn_11=v_value
cursor /A=1 /P /C=(3,52428,1)/S=0 A, IPSP_11, peakpn_11
print "Peak ist at: "+num2str(peak_11*1000)+" mV"
print "Peak was detected at: "+num2str(x_value_11*1000)+" ms, at point:
"+num2str(peakpn_11)
print "Membrane potential at peak is:"+num2str(peak_11*1000)+" mV"
// amplitude IPSP_11
variable ipsp_amplitude_11=peak_11-ipsp_baseline
print "IPSP amplitude is: "+num2str(ipsp_amplitude_11*1000)+" mV"
variable IPSPamplitude_11=ipsp_amplitude_11*1000
APnumber[10]="IPSP_11"
amplitudes[10]=IPSPamplitude_11
silent 1

Print "..........................................................................................."
//peak detection_IPSP_12
wavestats /Q /M=1 IPSP_12
variable peak_12=V_min
```

```
variable x_value_12=v_minloc
findvalue /v=(peak_12) IPSP_12
variable peakpn_12=v_value
cursor /A=1 /P /C=(3,52428,1)/S=0 A, IPSP_12, peakpn_12
print "Peak ist at: "+num2str(peak_12*1000)+" mV"
print "Peak was detected at: "+num2str(x_value_12*1000)+" ms, at point:
"+num2str(peakpn_12)
print "Membrane potential at peak is:"+num2str(peak_12*1000)+" mV"
// amplitude IPSP_12
variable ipsp_amplitude_12=peak_12-ipsp_baseline
print "IPSP amplitude is: "+num2str(ipsp_amplitude_12*1000)+" mV"
variable IPSPamplitude_12=ipsp_amplitude_12*1000
APnumber[11]="IPSP_12"
amplitudes[11]=IPSPamplitude_12
silent 1

Print "..........................................................................................."
//peak detection_IPSP_13
wavestats /Q /M=1 IPSP_13
variable peak_13=V_min
variable x_value_13=v_minloc
findvalue /v=(peak_13) IPSP_13
variable peakpn_13=v_value
cursor /A=1 /P /C=(3,52428,1)/S=0 A, IPSP_13, peakpn_13
print "Peak ist at: "+num2str(peak_13*1000)+" mV"
print "Peak was detected at: "+num2str(x_value_13*1000)+" ms, at point:
"+num2str(peakpn_13)
print "Membrane potential at peak is:"+num2str(peak_13*1000)+" mV"
silent 1
// amplitude IPSP_13
variable ipsp_amplitude_13=peak_13-ipsp_baseline
print "IPSP amplitude is: "+num2str(ipsp_amplitude_13*1000)+" mV"
variable IPSPamplitude_13=ipsp_amplitude_13*1000
APnumber[12]="IPSP_13"
amplitudes[12]=IPSPamplitude_13
silent 1

Print "..........................................................................................."
//peak detection_IPSP_14
wavestats /Q /M=1 IPSP_14
variable peak_14=V_min
variable x_value_14=v_minloc
findvalue /v=(peak_14) IPSP_14
variable peakpn_14=v_value
cursor /A=1 /P /C=(3,52428,1)/S=0 A, IPSP_14, peakpn_14
print "Peak ist at: "+num2str(peak_14*1000)+" mV"
print "Peak was detected at: "+num2str(x_value_14*1000)+" ms, at point:
"+num2str(peakpn_14)
print "Membrane potential at peak is:"+num2str(peak_14*1000)+" mV"
// amplitude IPSP_14
```

```
variable ipsp_amplitude_14=peak_14-ipsp_baseline
print "IPSP amplitude is: "+num2str(ipsp_amplitude_14*1000)+" mV"
variable IPSPamplitude_14=ipsp_amplitude_14*1000
APnumber[13]="IPSP_14"
amplitudes[13]=IPSPamplitude_14
silent 1

Print "..........................................................................................."
//peak detection_IPSP_15
wavestats /Q /M=1 IPSP_15
variable peak_15=V_min
variable x_value_15=v_minloc
findvalue /v=(peak_15) IPSP_15
variable peakpn_15=v_value
cursor /A=1 /P /C=(3,52428,1)/S=0 A, IPSP_15, peakpn_15
print "Peak ist at: "+num2str(peak_15*1000)+" mV"
print "Peak was detected at: "+num2str(x_value_15*1000)+" ms, at point:
"+num2str(peakpn_15)
print "Membrane potential at peak is:"+num2str(peak_15*1000)+" mV"
// amplitude IPSP_15
variable ipsp_amplitude_15=peak_15-ipsp_baseline
print "IPSP amplitude is: "+num2str(ipsp_amplitude_15*1000)+" mV"
variable IPSPamplitude_15=ipsp_amplitude_15*1000
APnumber[14]="IPSP_15"
amplitudes[14]=IPSPamplitude_15
silent 1

Print "..........................................................................................."
//peak detection_IPSP_16
wavestats /Q /M=1 IPSP_16
variable peak_16=V_min
variable x_value_16=v_minloc
findvalue /v=(peak_16) IPSP_16
variable peakpn_16=v_value
cursor /A=1 /P /C=(3,52428,1)/S=0 A, IPSP_16, peakpn_16
print "Peak ist at: "+num2str(peak_16*1000)+" mV"
print "Peak was detected at: "+num2str(x_value_16*1000)+" ms, at point:
"+num2str(peakpn_16)
print "Membrane potential at peak is:"+num2str(peak_16*1000)+" mV"
// amplitude IPSP_16
variable ipsp_amplitude_16=peak_16-ipsp_baseline
print "IPSP amplitude is: "+num2str(ipsp_amplitude_16*1000)+" mV"
variable IPSPamplitude_16=ipsp_amplitude_16*1000
APnumber[15]="IPSP_16"
amplitudes[15]=IPSPamplitude_16
silent 1

Print "..........................................................................................."
//peak detection_IPSP_17
wavestats /Q /M=1 IPSP_17
```

```
variable peak_17=V_min
variable x_value_17=v_minloc
findvalue /v=(peak_17) IPSP_17
variable peakpn_17=v_value
cursor /A=1 /P /C=(3,52428,1)/S=0 A, IPSP_17, peakpn_17
print "Peak ist at: "+num2str(peak_17*1000)+" mV"
print "Peak was detected at: "+num2str(x_value_17*1000)+" ms, at point: 
"+num2str(peakpn_17)
print "Membrane potential at peak is:"+num2str(peak_17*1000)+" mV"
// amplitude IPSP_17
variable ipsp_amplitude_17=peak_17-ipsp_baseline
print "IPSP amplitude is: "+num2str(ipsp_amplitude_17*1000)+" mV"
variable IPSPamplitude_17=ipsp_amplitude_17*1000
APnumber[16]="IPSP_17"
amplitudes[16]=IPSPamplitude_17
silent 1

Print "............................................................................................"
//peak detection_IPSP_18
wavestats /Q /M=1 IPSP_18
variable peak_18=V_min
variable x_value_18=v_minloc
findvalue /v=(peak_18) IPSP_18
variable peakpn_18=v_value
cursor /A=1 /P /C=(3,52428,1)/S=0 A, IPSP_18, peakpn_18
print "Peak ist at: "+num2str(peak_18*1000)+" mV"
print "Peak was detected at: "+num2str(x_value_18*1000)+" ms, at point: 
"+num2str(peakpn_18)
print "Membrane potential at peak is:"+num2str(peak_18*1000)+" mV"
// amplitude IPSP_18
variable ipsp_amplitude_18=peak_18-ipsp_baseline
print "IPSP amplitude is: "+num2str(ipsp_amplitude_18*1000)+" mV"
variable IPSPamplitude_18=ipsp_amplitude_18*1000
APnumber[17]="IPSP_18"
amplitudes[17]=IPSPamplitude_18
silent 1

Print "............................................................................................"
//peak detection_IPSP_19
wavestats /Q /M=1 IPSP_19
variable peak_19=V_min
variable x_value_19=v_minloc
findvalue /v=(peak_19) IPSP_19
variable peakpn_19=v_value
cursor /A=1 /P /C=(3,52428,1)/S=0 A, IPSP_19, peakpn_19
print "Peak ist at: "+num2str(peak_19*1000)+" mV"
print "Peak was detected at: "+num2str(x_value_19*1000)+" ms, at point: 
"+num2str(peakpn_19)
print "Membrane potential at peak is:"+num2str(peak_19*1000)+" mV"
// amplitude IPSP_19
```

```
variable ipsp_amplitude_19=peak_19-ipsp_baseline
print "IPSP amplitude is: "+num2str(ipsp_amplitude_19*1000)+" mV"
variable IPSPamplitude_19=ipsp_amplitude_19*1000
APnumber[18]="IPSP_19"
amplitudes[18]=IPSPamplitude_19
silent 1

Print "...................................................................................."
//peak detection_IPSP_20
wavestats /Q /M=1 IPSP_20
variable peak_20=V_min
variable x_value_20=v_minloc
findvalue /v=(peak_20) IPSP_20
variable peakpn_20=v_value
cursor /A=1 /P /C=(3,52428,1)/S=0 A, IPSP_20, peakpn_20
print "Peak ist at: "+num2str(peak_20*1000)+" mV"
print "Peak was detected at: "+num2str(x_value_20*1000)+" ms, at point:
"+num2str(peakpn_20)
print "Membrane potential at peak is:"+num2str(peak_20*1000)+" mV"
// amplitude IPSP_20
variable ipsp_amplitude_20=peak_20-ipsp_baseline
print "IPSP amplitude is: "+num2str(ipsp_amplitude_20*1000)+" mV"
variable IPSPamplitude_20=ipsp_amplitude_20*1000
APnumber[19]="IPSP_20"
amplitudes[19]=IPSPamplitude_20
silent 1
Print "...................................................................................."

Results_InterAPs_IPSP()

Endmacro


//=====================Results Tables ==============================


Window Results() : Table
	PauseUpdate; Silent 1		// building window...
	Edit/W=(305,535,796,658)
Baseline,IPSPpeak,IPSPamplitude,IPSPtau,FitAsymptote
	ModifyTable format(Point)=1
EndMacro

Window Results_InterAPs_IPSP() : Table
	PauseUpdate; Silent 1		// building window...
	Edit/W=(305,535,796,658) APnumber, Amplitudes
	ModifyTable format(Point)=1
EndMacro

//=====================Panel Macros
```

```
Window Analysis() : Panel
	PauseUpdate; Silent 1		// building window...
	NewPanel /W=(87,569,376,692)
	Button button0,pos={19,29},size={70,35},proc=ButtonProc,title="1 AP"
	Button button1,pos={106,28},size={70,36},proc=ButtonProc_1,title="5 APs"
	Button button2,pos={194,30},size={70,35},proc=ButtonProc_2,title="20 APs"
	Button button3,pos={86,82},size={103,22},proc=ButtonProc_3,title="Results Table"
EndMacro

Function ButtonProc(ba) : ButtonControl
	STRUCT WMButtonAction &ba



	switch( ba.eventCode )
		case 2: // mouse up
			execute "IPSP_analysis_1AP()"// click code here
			break
	endswitch

	return 0
End

Function ButtonProc_1(ba) : ButtonControl
	STRUCT WMButtonAction &ba


	switch( ba.eventCode )
		case 2: // mouse up
			execute "IPSP_Analysis_5APs()"// click code here
			break
	endswitch

	return 0
End

Function ButtonProc_2(ba) : ButtonControl
	STRUCT WMButtonAction &ba



	switch( ba.eventCode )
		case 2: // mouse up
			execute "IPSP_Analysis_20APs()" // click code here
			break
	endswitch

	return 0
End
```

```
Function ButtonProc_3(ba) : ButtonControl
	STRUCT WMButtonAction &ba

	switch( ba.eventCode )
		case 2: // mouse up
			execute "Results()"// click code here
			break
	endswitch

	return 0
End


Function ButtonProc_4(ba) : ButtonControl
	STRUCT WMButtonAction &ba

	switch( ba.eventCode )
		case 2: // mouse up
			execute "Inter_AP_IPSP()"  // click code here
			break
	endswitch

	return 0
End
```

3 – Analysis of the behavior data

```
#pragma rtGlobals=1     // Use modern global access method.
#include <Percentile and Box Plot>
#include <median>
Menu "Olfactometer Analysis"
   "Start Main Panel", MainControl()
END


function GlobalConstants()
   variable /g MAXVALVNUMBER=10
   variable /g MAXNUMBEROFGROUPS=3
   variable /g MAXDISPLAYEDPERTASK=50
   variable /g MAXNUMBEROFTICKS=20 // needs to be < 30
   variable /g MAXLISTPOINTS=100
   variable /g ANIMALCHAR=1
   string /g SPECIFICWAVEENDING="ibw"
   make /O /N=(MAXNUMBEROFGROUPS)
GROUPCOLORRED,GROUPCOLORGREEN,GROUPCOLORBLUE
   GROUPCOLORRED[0]=65535; GROUPCOLORGREEN[0]=65535;
GROUPCOLORBLUE[0]=65535;
   GROUPCOLORRED[1]=0; GROUPCOLORGREEN[1]=      0 ;
GROUPCOLORBLUE[1]=       0;
   GROUPCOLORRED[2]=  65535; GROUPCOLORGREEN[2]=      0 ;
GROUPCOLORBLUE[2]=       0;



end

Window MainControl() : Panel
   DoWindow /K MainControl
   GlobalConstants()
   variable /g Trailing0An, Trailing0Task
   PauseUpdate; Silent 1       // building window...
   NewPanel  /K=2 /W=(144.75,70.25,795.75,404) as "Main Analysis Window"
   SetDrawLayer UserBack
   DrawText 40,190,"Save Experiment under New Name. Press \"Init\" to Initialize."
   DrawText 40,210,"Enter name of the Analysis and a short Base name that will be added
to all variables."
   DrawText 40,230,"\"Start new Analysis\" will create a notebook window (name=base
Name, Title = Name Of Analysis)"
   DrawText 40,250,"Finally press \"Names of Individual Experiments\" to locate the data"
   DrawText 40,270,"Proceed with either \"individual analysis\" (Mainly for displaying
purpose) "
   DrawText 40,290,"or \"Multiple Animal Analysis\" (For proper analysis) and follow
instructions"
   SetVariable AnalysisName,pos={152,25},size={287,16},title="Name Of Analysis"
   SetVariable AnalysisName,limits={-Inf,Inf,1},value= AnalysisName
```

```
    SetVariable BaseName,pos={152,43},size={287,16},title="Base Name "
    SetVariable BaseName,limits={-Inf,Inf,1},value= baseName
//    Button InitAll,pos={37,26},size={86,32},proc=ButtonProc_InitAll,title="Init all"
    Button
StartAnalysis,pos={37,66},size={182,31},proc=ButtonProc_StartAnalysis,title="Start New
Analysis"
    Button
NamesExp,pos={235,66},size={182,31},proc=ButtonProc_NamesExp,title="Names Of
Individual Experiments ..."
    Button
IndividualAnimalAnalysis,pos={37,120},size={182,31},proc=ButtonProc_IndAnimalAnalysis
,title="Individual Animal Analysis ..."
    Button
MultipleAnimalAnalysis_1,pos={235,120},size={182,31},proc=ButtonProc_MultAnimalAnal
ysis,title="Multiple Animal Analysis ..."
EndMacro

Window NamesOfExpWindow() : Panel
    DoWindow /K NamesOfExpWindow
    PauseUpdate; Silent 1       // building window...
    NewPanel  /K=1 /W=(261.75,181.25,446.25,341) as "Names of Individual Experiments"
    Button
EnterManually,pos={37,47},size={114,27},proc=ButtonProc_EnterManually,title="Enter
Manually"
    Button
OnlyDatesManually,pos={38,82},size={114,27},proc=ButtonProc_OnlyDatesManually,title=
"Only Dates Manually"
    Button
NamesAllAutomatic,pos={38,118},size={114,27},proc=ButtonProc_AllAutomatic,title="All
Automatic"
    SetVariable NumberOfTasks,pos={7,5},size={170,16},title="Total Number Of Tasks"
    SetVariable NumberOfTasks,limits={-Inf,Inf,1},value= NumberOfTasks
    SetVariable NumberOfAnimals,pos={7,24},size={170,16},title="Total Number Of
Animals"
    SetVariable NumberOfAnimals,limits={-Inf,Inf,1},value= NumberOfAnimals
EndMacro


Window AllAutomaticNameWindow() : Panel
    DoWindow /K AllAutomaticNameWindow
    PauseUpdate; Silent 1       // building window...
    NewPanel  /K=1 /W=(401.25,166.25,719.25,374.75) as "Construct Experiment Names
...."
    SetDrawLayer UserBack
    SetDrawEnv linethick= 5
    DrawLine 10,118,299,118
    DrawText 54,152,"RESULTING NAME (ANIMAL 2 TASK 1)"
    SetVariable
setvar0,pos={50,10},size={172,16},proc=Update_ExampleName,title="Animal name 1st
(1=True)"
```

```
	SetVariable setvar0,limits={0,1,1},value= AnimalAt1stPos
	SetVariable setvar0_1,pos={50,35},size={172,16},proc=Update_ExampleName,title="Base 1"
	SetVariable setvar0_1,limits={0,1,1},value= baseForExperimentNames1
	SetVariable setvar0_2,pos={13,157},size={280,16},title=" "
	SetVariable setvar0_2,limits={0,1,1},value= exampleName
	SetVariable setvar0_3,pos={50,60},size={172,16},proc=Update_ExampleName,title="Base 2"
	SetVariable setvar0_3,limits={0,1,1},value= baseForExperimentNames2
	Button button0,pos={91,180},size={114,20},proc=ButtonProc_GenerateAllAutomatic,title="Generate and View ..."
	SetVariable setvar0_4,pos={50,85},size={172,16},proc=Update_ExampleName,title="Base 3"
	SetVariable setvar0_4,limits={0,1,1},value= baseForExperimentNames3
EndMacro

Window DatesManuallyWindow() : Panel
	DoWindow /K DatesManuallyWindow
	PauseUpdate; Silent 1		// building window...
	NewPanel  /K=1 /W=(401.25,166.25,750,374.75) as "Construct Experiment Names ...."
	SetDrawLayer UserBack
	SetDrawEnv linethick= 5
	DrawLine 10,118,299,118
	DrawText 54,152,"RESULTING NAME (ANIMAL 2 TASK 1)"
	SetVariable setvar0,pos={50,10},size={172,16},proc=Update_ExampleName,title="Animal name 1st (1=True)"
	SetVariable setvar0,limits={0,1,1},value= AnimalAt1stPos
	SetVariable setvar0_1,pos={50,35},size={172,16},proc=Update_ExampleName,title="Base 1"
	SetVariable setvar0_1,limits={0,1,1},value= baseForExperimentNames1
	SetVariable setvar0_5,pos={230,45},size={120,16},title="Trailing 0 Animals:"
	SetVariable setvar0_5,limits={0,1,1},value= Trailing0An
	SetVariable setvar0_6,pos={230,70},size={120,16},title="Trailing 0 Tasks:"
	SetVariable setvar0_6,limits={0,1,1},value= Trailing0Task
	SetVariable setvar0_2,pos={13,157},size={280,16},title=" "
	SetVariable setvar0_2,limits={0,1,1},value= exampleName
	SetVariable setvar0_3,pos={50,60},size={172,16},proc=Update_ExampleName,title="Base 2"
	SetVariable setvar0_3,limits={0,1,1},value= baseForExperimentNames2
	Button button0,pos={91,180},size={114,20},proc=ButtonProc_GenHalfAutomatic,title="Generate and View ..."
	SetVariable setvar0_4,pos={50,85},size={172,16},proc=Update_ExampleName,title="Base 3"
	SetVariable setvar0_4,limits={0,1,1},value= baseForExperimentNames3
EndMacro
```

```
Function ButtonProc_IndAnimalAnalysis(ctrlName) : ButtonControl
   String ctrlName
   execute("IndividualAnimalAnalysis()")
End

Function ButtonProc_MultAnimalAnalysis(ctrlName) : ButtonControl
   String ctrlName
   variable /g NumberOfAnimals,NumberOfGroups
   variable /g CountOnlyCorrect
   make /O /N=(NumberOfAnimals+1) GroupOfAnimal // +1 due to counting starting
usually at 1
   variable i=0
   do
      variable /g $("tmpA_"+num2str(i))
      i+=1
    while(i<NumberOfAnimals+1)
    execute("MultipleAnimalAnalysis()")
End

Function ButtonProc_EnterManually(ctrlName) : ButtonControl
   String ctrlName
   DoWindow /K NamesOfExpWindow
   ManualEntryNamesOfExp()
End

Function ButtonProc_OnlyDatesManually(ctrlName) : ButtonControl
   String ctrlName
   DoWindow /K NamesOfExpWindow
   variable/g NumberOfTasks, NumberOfAnimals
   make /N=(NumberOfTasks) /O /T DatePrefix, DateSuffix
   make /N=(NumberOfTasks) /O Tasks
   make /N=(NumberOfAnimals) /O /T AnimalPreFix
   make /N=(NumberOfAnimals) /O Animals
   Animals=x+1
   Tasks=x+1
   execute("TableForManualDates()")
   execute("TableForManualAnimals()")
   execute("DatesManuallyWindow()")
End

Window TableForManualDates() : Table
        DoWIndow /K TableForManualDates
        PauseUpdate; Silent 1                 // building window...
        Edit/W=(543,39.5,760.5,472.25)/K=1 Tasks,DatePrefix,DateSuffix as "Enter Pre-
and Suffix for each Task"
        ModifyTable width(Point)=0,style(Tasks)=1,width(Tasks)=41
EndMacro

Window TableForManualAnimals() : Table
        DoWIndow /K TableForManualAnimals
```

```
	PauseUpdate; Silent 1		// building window...
	Edit/W=(343,39.5,535,472.25)/K=1 Animals,AnimalPrefix as "Enter Prefix for each
Animal"
	ModifyTable width(Point)=0,style(Animals)=1,width(Animals)=41
EndMacro

Function ButtonProc_AllAutomatic(ctrlName) : ButtonControl
   String ctrlName
   string /g
baseForExperimentNames1,baseForExperimentNames2,baseForExperimentNames3,
exampleName
   variable /g AnimalAt1stPos
   Update_ExampleName("",1,"","")
   execute("AllAutomaticNameWindow()")
   DoWindow /K NamesOfExpWindow
End

Function ButtonProc_StartAnalysis(ctrlName) : ButtonControl
   String ctrlName
   StartAnalysis()
End

Function ButtonProc_NamesExp(ctrlName) : ButtonControl
   String ctrlName
   NamesOfExp()
End

Function ButtonProc_InitAll(ctrlName) : ButtonControl
   String ctrlName
   string /g AnalysisName="Temp Analysis", baseName="AnlTmp"
End

function NamesOfExp()
	// pops up Dialogue how task name sshould be specified
	string /g baseName, IndividualTaskName = baseName+"_IndividualTaskName" //
2D wave that contains name of task for each animal and day
	variable /g NumberOfTasks, NumberOfAnimals
	execute("NamesOfExpWindow()")
end

function ManualEntryNamesOfExp()
	string /g baseName, IndividualTaskName, IndividualTaskTableName =
baseName+"_IndividualTaskTablename" // how the popup table is named
	variable /g NumberOfTasks, NumberOfAnimals
	make /O /T /N=(NumberOfAnimals, NumberOfTasks) $IndividualTaskName
	NewPath /O /M="Select Folder, whose subdirectories contain the data" DataPath
	edit /K=1 $IndividualTaskName as "Columns=Tasks, Rows=Animals"
	DoALert 0, "Check / Fill Out Table, then close it and return to Main Analysis
WIndow"
end
```

```
Function Update_ExampleName(ctrlName,varNum,varStr,varName) : SetVariableControl
	String ctrlName
	Variable varNum
	String varStr
	String varName
	variable /g AnimalAt1stPos
	string /g
baseForExperimentNames1,baseForExperimentNames2,baseForExperimentNames3,
exampleName
	string local1, local2
	if(animalAt1stPos)
		local1="2"
		local2="1"
	else
		local1="1"
		local2="2"
	endif
	exampleName=baseForExperimentNames1+local1+baseForExperimentNames2+lo
cal2+baseForExperimentNames3
end

Function ButtonProc_GenerateAllAutomatic(ctrlName) : ButtonControl
	String ctrlName
	string local1,local2
	string /g baseName, IndividualTaskName, IndividualTaskTableName =
baseName+"_IndividualTaskTablename" // how the popup table is named
	string /g
baseForExperimentNames1,baseForExperimentNames2,baseForExperimentNames3
	variable /g NumberOfTasks, NumberOfAnimals, animalAt1stPos, ANIMALCHAR


	DoWindow /K AllAutomaticNameWindow
	make /O /T /N=(NumberOfAnimals, NumberOfTasks) $IndividualTaskName
	duplicate /O /T $IndividualTaskName, waveNamesLOC

	variable cTr=0, cAn=0
	do
		cAn=0
		do
			if(animalAt1stPos)
				local1=num2str(cAn+1)
				if(AnimalChar)
					local1=num2char(cAn+97)
				endif
				local2=num2str(cTr+1)
			else
				local1=num2str(cTr+1)
				local2=num2str(cAn+1)
				if(AnimalChar)
```

```
					local2=num2char(cAn+97)
				endif

			endif

	waveNamesLOC[cAn][cTr]=baseForExperimentNames1+local1+baseForExperimen
tNames2+local2+baseForExperimentNames3
			cAn+=1
		while(cAn<NumberOfAnimals)
		cTr+=1
	while(cTr<NumberOfTasks)
	duplicate /O waveNamesLOC, $IndividualTaskName
	ManualEntryNamesOfExp()
End

Function ButtonProc_GenHalfAutomatic(ctrlName) : ButtonControl
	String ctrlName
	string local1,local2
	string /g baseName, IndividualTaskName, IndividualTaskTableName =
baseName+"_IndividualTaskTablename" // how the popup table is named
	string /g
baseForExperimentNames1,baseForExperimentNames2,baseForExperimentNames3
	variable /g NumberOfTasks, NumberOfAnimals, animalAt1stPos, Trailing0An,
Trailing0Task
	make /O /N=(NumberOfTasks) /T DateSuffix, DatePrefix
	make /O /N=(NumberOfAnimals) /T AnimalPrefix
//	DoWindow /K AllAutomaticNameWindow
	make /O /T /N=(NumberOfAnimals, NumberOfTasks) $IndividualTaskName
	duplicate /O /T $IndividualTaskName, waveNamesLOC

	variable cTr=0, cAn=0
	do
		cAn=0
		do
			if(animalAt1stPos)
				local1=num2str(cAn+1)
				if(Trailing0An*(cAn<9))
					local1="0"+local1
				endif
				local2=num2str(cTr+1)
				if(Trailing0Task*(cTr<9))
					local2="0"+local2
				endif
			else
				local1=num2str(cTr+1)
				if(Trailing0Task*(cTr<9))
					local1="0"+local1
				endif
				local2=num2str(cAn+1)
				if(Trailing0An*(cAn<9))
```

```
					local2="0"+local2
				endif
			endif

		waveNamesLOC[cAn][cTr]=AnimalPrefix[cAn]+DatePrefix[cTr]+baseForExperiment
Names1+local1+baseForExperimentNames2+local2+baseForExperimentNames3+DateSu
ffix[cTr]
			cAn+=1
		while(cAn<NumberOfAnimals)
		cTr+=1
	while(cTr<NumberOfTasks)
	duplicate /O waveNamesLOC, $IndividualTaskName
	ManualEntryNamesOfExp()
End



FUNCTION TestItF()
variable a,b
sscanf "123:22", "%f:%f", a, b
print a,b
end


MACRO DOIt()

variable i=1
do
	DispFirstdetect=i
	ButtonProc_IndDisplayAndLayout("")
	keepName="Det"+num2str(i)
	ButtonProc_IndKeepAndLayout("")
	i+=1
while(i<6)
endmacro
Window Layout0() : Layout
	PauseUpdate; Silent 1		// building window...
	Layout/C=1/W=(5.25,42.5,506.25,474.5) as " Layout"
EndMacro

Window Table12() : Table
	PauseUpdate; Silent 1		// building window...
	Edit/W=(27,67.25,265.5,335) tmpP
	ModifyTable format(Point)=1
EndMacro

Window Table17() : Table
	PauseUpdate; Silent 1		// building window...
	Edit/W=(39,154.25,768,428.75) ForANOVA_FitSlope
	ModifyTable format(Point)=1,width(ForANOVA_FitSlope)=56
```

```
EndMacro

Window Table14() : Table
	PauseUpdate; Silent 1		// building window...
	Edit/W=(10.5,266.75,804.75,444.5) ForANOVA_PercLick
	ModifyTable format(Point)=1,width(ForANOVA_PercLick)=62
	MoveWindow 0, 0, 0, 0		// Minimize the window.
EndMacro

Window Table1() : Table
	PauseUpdate; Silent 1		// building window...
	Edit/W=(4.5,42.5,525.75,234.5) tmpM,tmpP
	ModifyTable format(Point)=1
	MoveWindow 0, 0, 0, 0		// Minimize the window.
EndMacro

Window Table6() : Table
	PauseUpdate; Silent 1		// building window...
	Edit/W=(18,42.5,654.75,258.5) tmp2_LickPWave
	ModifyTable format(Point)=1
	MoveWindow 0, 0, 0, 0		// Minimize the window.
EndMacro

Window Table16() : Table
	PauseUpdate; Silent 1		// building window...
	Edit/W=(37.5,156.5,739.5,383) ForANOVA_FitMax
	ModifyTable format(Point)=1,width(ForANOVA_FitMax)=68
	MoveWindow 0, 0, 0, 0		// Minimize the window.
EndMacro

Window Table4() : Table
	PauseUpdate; Silent 1		// building window...
	Edit/W=(4.5,42.5,510,201.5) SM,SP
	ModifyTable format(Point)=1
	MoveWindow 0, 0, 0, 0		// Minimize the window.
EndMacro

Window Table13() : Table
	PauseUpdate; Silent 1		// building window...
	Edit/W=(-81,242,732.75,455.75) ForANOVA_ITI
	ModifyTable width=65,format(Point)=1
	MoveWindow 0, 0, 0, 0		// Minimize the window.
EndMacro

Window Table10() : Table
	PauseUpdate; Silent 1		// building window...
	Edit/W=(25.5,235.25,554.25,459.5) tmpP
	ModifyTable format(Point)=1
	MoveWindow 0, 0, 0, 0		// Minimize the window.
EndMacro
```

```
Window Table3() : Table
	PauseUpdate; Silent 1		// building window...
	Edit/W=(4.5,42.5,510,201.5) lickP1
	ModifyTable format(Point)=1
	MoveWindow 0, 0, 0, 0		// Minimize the window.
EndMacro

Window Table15() : Table
	PauseUpdate; Silent 1		// building window...
	Edit/W=(5.25,42.5,768.75,220.25) ForANOVA_PercCorr
	ModifyTable format(Point)=1,width(ForANOVA_PercCorr)=60
	MoveWindow 0, 0, 0, 0		// Minimize the window.
EndMacro

Window Table7() : Table
	PauseUpdate; Silent 1		// building window...
	Edit/W=(6.75,204.5,610.5,536) tmp_LickPWave
	ModifyTable format(Point)=1
EndMacro

Window Table0() : Table
	PauseUpdate; Silent 1		// building window...
	Edit/W=(4.5,42.5,582.75,439.25) tmpM,tmpP
	ModifyTable format(Point)=1
	MoveWindow 0, 0, 0, 0		// Minimize the window.
EndMacro

Window Table11() : Table
	PauseUpdate; Silent 1		// building window...
	Edit/W=(13.5,255.5,835.5,557.75) tmp2_LickMWave
	ModifyTable format(Point)=1
	MoveWindow 0, 0, 0, 0		// Minimize the window.
EndMacro

Window Table5() : Table
	PauseUpdate; Silent 1		// building window...
	Edit/W=(31.5,248,554.25,604.25) tmp_LickPWave
	ModifyTable format(Point)=1
	MoveWindow 0, 0, 0, 0		// Minimize the window.
EndMacro

Window Table8() : Table
	PauseUpdate; Silent 1		// building window...
	Edit/W=(4.5,42.5,609.75,407) tmpM,tmpP
	ModifyTable format(Point)=1
	MoveWindow 0, 0, 0, 0		// Minimize the window.
EndMacro

Window Table2() : Table
```

```
	PauseUpdate; Silent 1		// building window...
	Edit/W=(465,42.5,877.5,280.25) SMinusValves
	ModifyTable format(Point)=1
	MoveWindow 0, 0, 0, 0		// Minimize the window.
EndMacro

Window Table9() : Table
	PauseUpdate; Silent 1		// building window...
	Edit/W=(96,177.5,693,542.75) tmpM,tmpP
	ModifyTable format(Point)=1
	MoveWindow 0, 0, 0, 0		// Minimize the window.
EndMacro

Window Table18() : Table
	PauseUpdate; Silent 1		// building window...
	Edit/W=(218.25,549.5,861.75,799.25) ForANOVA_FitHalf
	ModifyTable format(Point)=1,width(ForANOVA_FitHalf)=59
	MoveWindow 0, 0, 0, 0		// Minimize the window.
EndMacro

Window Graph0() : Graph
	PauseUpdate; Silent 1		// building window...
	Display /W=(5.25,41.75,399.75,250.25) tmp_ttestValues
	ModifyGraph log(left)=1
	Cursor/P A tmp_ttestValues 43
	ShowInfo
EndMacro


#pragma rtGlobals=1	// Use modern global access method.
// rather hard-wired Criterion definition as in the experiment
// change Function IsCriterion() for other Criterion definitions
// assumption: number of Trials for Odorvalve same as for LickPattern
Function GetLickPattern()
   string /g IndividualTaskName, SPECIFICWAVEENDING
   variable /g CurrentTask, WhichAnimal, LengthOfTimeSampling, NumberOfTasks,
MAXVALVNUMBER
   variable /g startTrials, endTrials, CountOnlyCorrect
   duplicate /o /T $IndividualTaskName, tmpWave1
   variable i, tmpLogic,j, numPTrials=0, numMTrials=0, ToBeCountedAsCorrect
   string PathSpecifier
   make /N=(NumberOfTasks) /O NumSPlusValves, NumSMinusValves
   make /O /N=(LengthOfTimeSampling) tmp_LickPWave=0,tmp_LickMWave=0
   make /O /N=(LengthOfTimeSampling) LickPatternTmp
   make /O OdorValve, SamplePattern
   make /N=(NumSPlusValves[CurrentTask-1]) /O SPlusValvesTmp
   make /N=(NumSMinusValves[CurrentTask-1]) /O SMinusValvesTmp
   make /N=(NumberOfTasks,MAXVALVNUMBER) /O SPlusValves
   make /N=(NumberOfTasks,MAXVALVNUMBER) /O SMinusValves
```

```
    SPlusValvesTmp=SPlusValves[CurrentTask-1][p]
    SMinusValvesTmp=SMinusValves[CurrentTask-1][p]

    PathSpecifier=tmpWave1[WhichAnimal-1][CurrentTask-1]
    killwaves OdorValve, SamplePattern, lickpattern
    LoadWave/H/O/P=DataPath/K=0 ":"+PathSpecifier+"
Folder:OdorValve."+SPECIFICWAVEENDING
    LoadWave/H/O/P=DataPath/K=0 ":"+PathSpecifier+"
Folder:lickPattern."+SPECIFICWAVEENDING; wave lickpattern=lickpattern
    i=startTrials
    do
        j=0;tmpLogic=0      // next couple of lines assess whether trial i was any of the s+
trials
        do
          tmpLogic+=(OdorValve[i]==SPlusValvesTmp[j])
          j+=1
        while(j<NumSPlusValves[CurrentTask-1])
        if(tmpLogic)
          tmp_LickPWave+=LickPattern[i][p]
          numPTrials+=1
        endif
        j=0;tmpLogic=0      // next couple of lines assess whether tril i was any of the s- trials
        do
          tmpLogic+=(OdorValve[i]==SMinusValvesTmp[j])
          j+=1
        while(j<NumSMinusValves[CurrentTask-1])
        if(tmpLogic)
          tmp_LickMWave+=LickPattern[i][p]
          numMTrials+=1
        endif
        i+=1
    while(i<EndTrials)
  print "Trials : ", numpTrials, numMTrials
    tmp_LickPWave/=NumPTrials
    tmp_LickMWave/=NumMTrials
END

Function CalculateIsCorrect()
    variable /g LengthOfTImeSampling, CurrentTask, MAXVALVNUMBER
    variable /g
CurrentTask,CurrentTaskNumberOfTrials,NumberOfTasks,startTrials,endTrials
    make /O /N=(LengthOfTimeSampling) LickPatternTmp
    make /N=(NumberOfTasks,MAXVALVNUMBER) /O SPlusValves
    make /N=(NumberOfTasks,MAXVALVNUMBER) /O SMinusValves
    make /N=(CurrentTaskNumberOfTrials) /O OdorValve
    make /O /N=(CurrentTaskNumberOfTrials,LengthOfTimeSampling) LickPattern
    make /N=(NumberOfTasks) /O NumSPlusValves, NumSMinusValves

    make /N=(NumSPlusValves[CurrentTask-1]) /O SPlusValvesTmp
    make /N=(NumSMinusValves[CurrentTask-1]) /O SMinusValvesTmp
```

```
   variable i,j,tmpLogic,IsAccepted
   variable NumPTrials=0,NumMTrials=0, CorrPlus=0, UnCorrPLus=0, CorrMinus=0,
UnCorrMinus=0, returnVal

   SPlusValvesTmp=SPlusValves[CurrentTask-1][p]
   SMinusValvesTmp=SMinusValves[CurrentTask-1][p]

   i=startTrials
   do
      LickPatternTmp=LickPattern[i][p]
      IsAccepted=IsCriterion()
      j=0;tmpLogic=0      // next couple of lines assess whether trial i was any of the s+
trials
      do
        tmpLogic+=(OdorValve[i]==SPlusValvesTmp[j])
        j+=1
      while(j<NumSPlusValves[CurrentTask-1])
      if(tmpLogic)
         if(IsAccepted)
            CorrPlus+=1
         else
            UnCorrPlus+=1
         endif
         numPTrials+=1
      endif
      j=0;tmpLogic=0      // next couple of lines assess whether tril i was any of the s+ trials
      do
        tmpLogic+=(OdorValve[i]==SMinusValvesTmp[j])
        j+=1
      while(j<NumSMinusValves[CurrentTask-1])
      if(tmpLogic)
         if(IsAccepted)
            UnCorrMinus+=1
         else
            CorrMinus+=1
         endif
         numMTrials+=1
      endif
      i+=1
   while(i<endTrials)
   returnVal=(CorrPlus/max(NumPTrials,1)+CorrMinus/max(NumMTrials,1))/2
   if(NumMTrials<1)
        returnVal=CorrPlus/max(NumPTrials,1)
   endif
   if(NumPTrials<1)
        returnVal=CorrMinus/max(NumMTrials,1)
   endif

   return(returnVal)
END
```

```
Function CalculateIsLicked()
   variable /g LengthOfTImeSampling, CurrentTask, MAXVALVNUMBER
   variable /g
CurrentTask,CurrentTaskNumberOfTrials,NumberOfTasks,startTrials,endTrials
   make /O /N=(LengthOfTimeSampling) LickPatternTmp
   make /N=(NumberOfTasks,MAXVALVNUMBER) /O SPlusValves
   make /N=(NumberOfTasks,MAXVALVNUMBER) /O SMinusValves
   make /N=(CurrentTaskNumberOfTrials) /O OdorValve
   make /O /N=(CurrentTaskNumberOfTrials,LengthOfTimeSampling) LickPattern
   make /N=(NumberOfTasks) /O NumSPlusValves, NumSMinusValves

   make /N=(NumSPlusValves[CurrentTask-1]) /O SPlusValvesTmp
   make /N=(NumSMinusValves[CurrentTask-1]) /O SMinusValvesTmp
   variable i,j,tmpLogic,IsAccepted, Licked=0, NotLicked=0, numTrials=0
   variable NumPTrials=0,NumMTrials=0, CorrPlus=0, UnCorrPLus=0, CorrMinus=0,
UnCorrMinus=0

   SPlusValvesTmp=SPlusValves[CurrentTask-1][p]
   SMinusValvesTmp=SMinusValves[CurrentTask-1][p]

   i=startTrials
   do
      LickPatternTmp=LickPattern[i][p]
      IsAccepted=IsCriterion()
      if(IsAccepted)
            Licked+=1
      else
            NotLicked+=1
      endif
      numTrials+=1
      i+=1
   while(i<endTrials)
   return(Licked/numTrials)
END

Function DisplayPercentageCorrect()
   variable /g WhichAnimal, CurrentTask,StartTask,EndTask,WhichAnimalStart,
WhichAnimalStop, NumberOfAnimals
   variable /g LengthOfTImeSampling,
TrialsAveraged,NumberOfTasks,startTrials,endTrials
   make /N=(NumberOfAnimals+1) /O GroupOfAnimal
   variable /g MAXNUMBEROFGROUPS
   make /N=(MAXNUMBEROFGROUPS) /O
GROUPCOLORRED,GROUPCOLORGREEN,GROUPCOLORBLUE
   string /g AnlTmp_NoteBook,RecentDisplayWindowsList

   make /O  /N=(NumberOfTasks) TrialsPerTask
   DoWindow /K PercentageCorrectTmp
```

```
   variable i,j,NumberOfDataPoints,WaveEntryIndex
   i=StartTask-1
   do
      numberOfDataPoints+=round(TrialsPerTask[i]/TrialsAveraged+0.49)     // if trials are
averaged in 100 a piece but only 250 trials -> nevertheless three entries
      i+=1
   while(i<EndTask-1+0.1)

   make /N=(numberOfDataPoints) /O PercentCorrect
   display /W=(3,240,220,320)/K=1 as "Percentage Correct"
   DoWindow/C PercentageCorrectTmp
   ModifyGraph width=150,height=30
   RecentDisplayWindowsList=RecentDisplayWindowsList+"PercentageCorrectTmp;"

   WhichAnimal = WhichAnimalStart
   do
        if(GroupOfAnimal[WhichAnimal]>0)
      CurrentTask=StartTask
      WaveEntryIndex=0
      do
         GetLickPattern()
         j=0
         do
            startTrials=j*TrialsAveraged+1
            endTrials=min(TrialsPerTask[CurrentTask-1],startTrials+TrialsAveraged)
            PercentCorrect[WaveEntryIndex]=CalculateIsCorrect()
           // Notebook AnlTmp_NoteBook text="start "+num2str(startTrials)+ "; end :
"+num2str(endTrials) + "; WaveEntryIndex : "+num2str(WaveEntryIndex)+"\r"
            j+=1
            WaveEntryIndex+=1
         while(j<round(TrialsPerTask[CurrentTask-1]/TrialsAveraged+0.49))
         CurrentTask+=1
      while(CurrentTask<EndTask+0.1)
      duplicate /O PercentCorrect, $("tmpPercentCorrect_"+num2str(WhichAnimal))
      appendtograph $("tmpPercentCorrect_"+num2str(WhichAnimal))
      ModifyGraph  /W=PercentageCorrectTmp
rgb($("tmpPercentCorrect_"+num2str(WhichAnimal)))=(GROUPCOLORRED[GroupOfAni
mal[WhichAnimal]],GROUPCOLORGREEN[GroupOfAnimal[WhichAnimal]],GROUPCOLO
RBLUE[GroupOfAnimal[WhichAnimal]])
        endif
     WhichAnimal+=1
   While(WhichAnimal<WhichANimalStop+0.2)
END


FUNCTION IsCriterion()
   variable /g LengthOfTImeSampling
   make /o /N=(LengthOfTimeSampling) LickPatternTmp

   variable lick1, lick2, lick3, lick4
```

```
   wavestats /Q /R=[25,49] lickPatternTmp;  lick1=(V_avg>0)
   wavestats /Q /R=[50,74] lickPatternTmp;  lick2=(V_avg>0)
   wavestats /Q /R=[75,99] lickPatternTmp;  lick3=(V_avg>0)
   wavestats /Q /R=[100,124] lickPatternTmp;  lick4=(V_avg>0)
//  Print (lick1>0?1:0),(lick2>0?1:0),(lick3>0?1:0),(lick4>0?1:0)
   return (lick1+lick2+lick3+lick4>2?1:0)

END


#pragma rtGlobals=1                    // Use modern global access method.
Function GetDifferentTValues(WhichTest)
variable WhichTest // 0 no test, 1 equal variance, 2 ttest equal var, e ttest unqual var, 4
MannWhitney U test, 5 simple sortTest
   string /g IndividualTaskName, SPECIFICWAVEENDING
   variable /g CurrentTask, WhichAnimal, LengthOfTimeSampling, NumberOfTasks,
MAXVALVNUMBER, FinalValveTime,TemporalResolution
   variable /g startTrials, endTrials, SignificanceThreshold, FirstDetect
   duplicate /o /T $IndividualTaskName, tmpWave1
   variable i, tmpLogic,j, ToBeCountedAsCorrect
   variable /g numPTrials=0, numMTrials=0, CountOnlyCorrect
   string PathSpecifier
   make /N=(NumberOfTasks) /O NumSPlusValves, NumSMinusValves
   make /O /N=(endTrials-startTrials, LengthOfTimeSampling)
tmp_SamplingPWave=0,tmp_SamplingMWave=0
   make /O OdorValve, SamplePattern
   make /N=(NumSPlusValves[CurrentTask-1]) /O SPlusValvesTmp
   make /N=(NumSMinusValves[CurrentTask-1]) /O SMinusValvesTmp
   make /N=(NumberOfTasks,MAXVALVNUMBER) /O SPlusValves
   make /N=(NumberOfTasks,MAXVALVNUMBER) /O SMinusValves
   make /O /N=(LengthOfTimeSampling)  tmp_ttestValues=0, tmp_equalVariance=0
   make /O /N=(LengthOfTimeSampling) LickPatternTmp
 //   WhichTest=(WhichTest>0?5:0)
   make /O tmpP, tmpM // redimensioned below

   SPlusValvesTmp=SPlusValves[CurrentTask-1][p]
   SMinusValvesTmp=SMinusValves[CurrentTask-1][p]

   PathSpecifier=tmpWave1[WhichAnimal-1][CurrentTask-1]
   killwaves OdorValve, SamplePattern
   LoadWave/H/O/P=DataPath/K=0 ":"+PathSpecifier+"
Folder:samplePattern."+SPECIFICWAVEENDING
   LoadWave/H/O/P=DataPath/K=0 ":"+PathSpecifier+"
Folder:OdorValve."+SPECIFICWAVEENDING
   LoadWave/H/O/P=DataPath/K=0 ":"+PathSpecifier+"
Folder:lickPattern."+SPECIFICWAVEENDING; wave lickpattern=lickpattern
   i=startTrials
   do
         ToBeCountedAsCorrect=0.5
         if(CountOnlyCorrect)
```

```
         LickPatternTmp=LickPattern[i][p]
                  ToBeCountedAsCorrect=IsCriterion()
           endif
       j=0;tmpLogic=0      // next couple of lines assess whether trial i was any of the s+
trials
       do
         tmpLogic+=(OdorValve[i]==SPlusValvesTmp[j])
         j+=1
       while(j<NumSPlusValves[CurrentTask-1])
       if(tmpLogic*ToBeCountedAsCorrect)
         tmp_SamplingPWave[numPTrials][]=SamplePattern[i][q]
         numPTrials+=1
       endif
       j=0;tmpLogic=0      // next couple of lines assess whether tril i was any of the s+ trials
       do
         tmpLogic+=(OdorValve[i]==SMinusValvesTmp[j])
         j+=1
       while(j<NumSMinusValves[CurrentTask-1])
       if(tmpLogic*(1-ToBeCountedAsCorrect))
         tmp_SamplingMWave[numMTrials][]=SamplePattern[i][q]
         numMTrials+=1
       endif
       i+=1
    while(i<EndTrials)
//    make /O /N=(NumPTrials, LengthOfTimeSampling) tmp_SamplingPWave
//    make /O /N=(NumMTrials, LengthOfTimeSampling) tmp_SamplingMWave

    make /O /N=(numMTrials) tmpM
    make /O /N=(numPTrials) tmpP

    i=0
    do
       tmpM=tmp_samplingMWave[p][i]
       tmpP=tmp_samplingPWave[p][i]
//         tmp_equalVariance[i] = imag(statFtest(tmpM,tmpP))
           switch(WhichTest)
             case 1:
           tmp_ttestvalues[i]   = imag(statFtest(tmpM,tmpP))     // equal Variance
           break
         case 2:
           tmp_ttestvalues[i] = imag(statttest(0,tmpM,tmpP))     // t-Test, equal variance
assumed
           break
         case 3:
           tmp_ttestvalues[i] = imag(statttest(1,tmpM,tmpP))     // t-Test, unequal variance
           break
         case 4:
           tmp_ttestvalues[i] = statUTest("tmpM","tmpP")         // Mann-Whitney U Test
           break
         case 5:
```

```
            tmp_ttestvalues[i] = statSortTest("tmpM","tmpP")       // Mann-Whitney U Test
            break
        endswitch
          i+=1
    while(i<LengthOfTImeSampling)
    SetScale/P x -FinalValveTime,TemporalResolution, tmp_ttestvalues
    SetScale/P x -FinalValveTime,TemporalResolution, tmp_equalVariance
    duplicate /O tmp_ttestvalues, tmp
    tmp=tmp_ttestvalues[LengthOfTimeSampling-p]
    findlevel /Q tmp, SignificanceThreshold
    FirstDetect = TemporalResolution*LengthOfTimeSampling-(V_levelx+FinalValveTime)-
FinalValveTime
    if (V_flag)
         FirstDetect = TemporalResolution*LengthOfTimeSampling-FinalValveTime
    endif
    print numPTrials, numMTrials

END


Function GetDifferentTValuesLick(WhichTest)
variable WhichTest // 0 no test, 1 equal variance, 2 ttest equal var, e ttest unqual var, 4
MannWhitney U test, 5 simple sortTest

 if(WhichTest>10)
        GetDifferentTValuesLickBinned(WhichTest-10)
else

    string /g IndividualTaskName, SPECIFICWAVEENDING
    variable /g CurrentTask, WhichAnimal, LengthOfTimeSampling, NumberOfTasks,
MAXVALVNUMBER, FinalValveTime,TemporalResolution
    variable /g startTrials, endTrials, SignificanceThreshold, FirstDetect
    duplicate /o /T $IndividualTaskName, tmpWave1
    variable i, tmpLogic,j, ToBeCountedAsCorrect
    variable /g numPTrials=0, numMTrials=0, CountOnlyCorrect
    string PathSpecifier
    make /N=(NumberOfTasks) /O NumSPlusValves, NumSMinusValves
    make /O /N=(endTrials-startTrials, LengthOfTimeSampling)
tmp_LickPWave=0,tmp_LickMWave=0
    make /O OdorValve, LickPattern
    make /N=(NumSPlusValves[CurrentTask-1]) /O SPlusValvesTmp
    make /N=(NumSMinusValves[CurrentTask-1]) /O SMinusValvesTmp
    make /N=(NumberOfTasks,MAXVALVNUMBER) /O SPlusValves
    make /N=(NumberOfTasks,MAXVALVNUMBER) /O SMinusValves
    make /O /N=(LengthOfTimeSampling)  tmp_ttestValues=0, tmp_equalVariance=0
    make /O /N=(LengthOfTimeSampling) LickPatternTmp
//    WhichTest=(WhichTest>0?5:0)
    make /O tmpP, tmpM // redimensioned below

    SPlusValvesTmp=SPlusValves[CurrentTask-1][p]
```

```
   SMinusValvesTmp=SMinusValves[CurrentTask-1][p]

   PathSpecifier=tmpWave1[WhichAnimal-1][CurrentTask-1]
   killwaves OdorValve, LickPattern
   LoadWave/H/O/P=DataPath/K=0 ":"+PathSpecifier+"
Folder:OdorValve."+SPECIFICWAVEENDING
   LoadWave/H/O/P=DataPath/K=0 ":"+PathSpecifier+"
Folder:lickPattern."+SPECIFICWAVEENDING; wave lickpattern=lickpattern
   i=startTrials
   do
      j=0;tmpLogic=0      // next couple of lines assess whether trial i was any of the s+
trials
      do
        tmpLogic+=(OdorValve[i]==SPlusValvesTmp[j])
        j+=1
      while(j<NumSPlusValves[CurrentTask-1])
      if(tmpLogic)
        tmp_LickPWave[numPTrials][]=LickPattern[i][q]
        numPTrials+=1
      endif
      j=0;tmpLogic=0      // next couple of lines assess whether tril i was any of the s+ trials
      do
        tmpLogic+=(OdorValve[i]==SMinusValvesTmp[j])
        j+=1
      while(j<NumSMinusValves[CurrentTask-1])
      if(tmpLogic*(1-ToBeCountedAsCorrect))
        tmp_LickMWave[numMTrials][]=LickPattern[i][q]
        numMTrials+=1
      endif
      i+=1
   while(i<EndTrials)
//   make /O /N=(NumPTrials, LengthOfTimeLick) tmp_LickPWave
//   make /O /N=(NumMTrials, LengthOfTimeLick) tmp_LickMWave

   make /O /N=(numMTrials) tmpM
   make /O /N=(numPTrials) tmpP

   i=0
   do
      tmpM=tmp_LickMWave[p][i]
      tmpP=tmp_LickPWave[p][i]
//         tmp_equalVariance[i] = imag(statFtest(tmpM,tmpP))
          switch(WhichTest)
            case 1:
          tmp_ttestvalues[i]   = imag(statFtest(tmpM,tmpP))     // equal Variance
          break
        case 2:
          tmp_ttestvalues[i] = imag(statttest(0,tmpM,tmpP))     // t-Test, equal variance
assumed
          break
```

```
        case 3:
          tmp_ttestvalues[i] = imag(statttest(1,tmpM,tmpP))     // t-Test, unequal variance
          break
        case 4:
          tmp_ttestvalues[i] = statUTest("tmpM","tmpP")         // Mann-Whitney U Test
          break
        case 5:
          tmp_ttestvalues[i] = statSortTestLick("tmpM","tmpP")// Mann-Whitney U Test
          break
      endswitch
        i+=1
   while(i<LengthOfTImeSampling)
   SetScale/P x -FinalValveTime,TemporalResolution, tmp_ttestvalues
   SetScale/P x -FinalValveTime,TemporalResolution, tmp_equalVariance
   duplicate /O tmp_ttestvalues, tmp
   tmp=tmp_ttestvalues[LengthOfTimeSampling-p]
   findlevel /Q tmp, SignificanceThreshold
   FirstDetect = TemporalResolution*LengthOfTimeSampling-(V_levelx+FinalValveTime)-
FinalValveTime
   if (V_flag)
        FirstDetect = TemporalResolution*LengthOfTimeSampling-FinalValveTime
   endif
   print numPTrials, numMTrials
endif // jumping to Binned .....
END


Function GetDifferentTValuesLickBinned(WhichTest)
variable WhichTest // 0 no test, 1 equal variance, 2 ttest equal var, e ttest unqual var, 4
MannWhitney U test, 5 simple sortTest
   string /g IndividualTaskName, SPECIFICWAVEENDING
   variable /g CurrentTask, WhichAnimal, LengthOfTimeSampling, NumberOfTasks,
MAXVALVNUMBER, FinalValveTime,TemporalResolution
   variable /g startTrials, endTrials, SignificanceThreshold, FirstDetect
   duplicate /o /T $IndividualTaskName, tmpWave1
   variable i, tmpLogic,j, ToBeCountedAsCorrect
   variable /g numPTrials=0, numMTrials=0, CountOnlyCorrect
   variable /g TrialsSum
   string PathSpecifier
   make /N=(NumberOfTasks) /O NumSPlusValves, NumSMinusValves
   make /O /N=(endTrials-startTrials, LengthOfTimeSampling)
tmp_LickPWave=0,tmp_LickMWave=0
   make /O OdorValve, LickPattern
   make /N=(NumSPlusValves[CurrentTask-1]) /O SPlusValvesTmp
   make /N=(NumSMinusValves[CurrentTask-1]) /O SMinusValvesTmp
   make /N=(NumberOfTasks,MAXVALVNUMBER) /O SPlusValves
   make /N=(NumberOfTasks,MAXVALVNUMBER) /O SMinusValves
   make /O /N=(LengthOfTimeSampling)  tmp_ttestValues=0, tmp_equalVariance=0
   make /O /N=(LengthOfTimeSampling) LickPatternTmp
//    WhichTest=(WhichTest>0?5:0)
```

```
    make /O tmpP, tmpM // redimensioned below

    SPlusValvesTmp=SPlusValves[CurrentTask-1][p]
    SMinusValvesTmp=SMinusValves[CurrentTask-1][p]

    PathSpecifier=tmpWave1[WhichAnimal-1][CurrentTask-1]
    killwaves OdorValve, LickPattern
    LoadWave/H/O/P=DataPath/K=0 ":"+PathSpecifier+"
Folder:OdorValve."+SPECIFICWAVEENDING
    LoadWave/H/O/P=DataPath/K=0 ":"+PathSpecifier+"
Folder:lickPattern."+SPECIFICWAVEENDING; wave lickpattern=lickpattern
    i=startTrials
    do
        j=0;tmpLogic=0      // next couple of lines assess whether trial i was any of the s+
trials
        do
          tmpLogic+=(OdorValve[i]==SPlusValvesTmp[j])
          j+=1
        while(j<NumSPlusValves[CurrentTask-1])
        if(tmpLogic)
          tmp_LickPWave[numPTrials][]=LickPattern[i][q]
          numPTrials+=1
        endif
        j=0;tmpLogic=0      // next couple of lines assess whether tril i was any of the s+ trials
        do
          tmpLogic+=(OdorValve[i]==SMinusValvesTmp[j])
          j+=1
        while(j<NumSMinusValves[CurrentTask-1])
        if(tmpLogic*(1-ToBeCountedAsCorrect))
          tmp_LickMWave[numMTrials][]=LickPattern[i][q]
          numMTrials+=1
        endif
        i+=1
    while(i<EndTrials)
//    make /O /N=(NumPTrials, LengthOfTimeLick) tmp_LickPWave
//    make /O /N=(NumMTrials, LengthOfTimeLick) tmp_LickMWave
     make /O /N=(numMTrials/TrialsSum+1, LengthOfTimeSampling) tmp2_LickMWave=0
     make /O /N=(numPTrials/TrialsSum+1, LengthOfTimeSampling) tmp2_LickPWave=0
    variable num2M=0, num2P=0
    i=0
    do
         j=0
         do
                 tmp2_LickMWave[num2M][]+=tmp_LickMWave[i][q]
                 i+=1
                 j+=1
         while(j<TrialsSum)
         num2M+=1
        i+=1
    while(i<NumMTrials-TrialsSum+1)
```

```
   tmp2_LickMWave/=TrialsSum

   i=0
   do
        j=0
        do
                tmp2_LickPWave[num2P][]+=tmp_LickPWave[i][q]
                i+=1
                j+=1
        while(j<TrialsSum)
        num2P+=1
      i+=1
   while(i<NumPTrials-TrialsSum+1)
   tmp2_LickPWave/=TrialsSum

//    make /O /N=(Num2M, LengthOfTimeSampling) tmp2_LickMWave
//    make /O /N=(Num2P, LengthOfTimeSampling) tmp2_LickPWave

   make /O /N=(num2M) tmpM
   make /O /N=(num2P) tmpP

   i=0
   do
      tmpM=tmp2_LickMWave[p][i]
      tmpP=tmp2_LickPWave[p][i]
//         tmp_equalVariance[i] = imag(statFtest(tmpM,tmpP))
          switch(WhichTest)
            case 1:
          tmp_ttestvalues[i]   = imag(statFtest(tmpM,tmpP))     // equal Variance
          break
        case 2:
          tmp_ttestvalues[i] = imag(statttest(0,tmpM,tmpP))     // t-Test, equal variance
assumed
          break
        case 3:
          tmp_ttestvalues[i] = imag(statttest(1,tmpM,tmpP))     // t-Test, unequal variance
          break
        case 4:
          tmp_ttestvalues[i] = statUTest("tmpM","tmpP")         // Mann-Whitney U Test
          break
        case 5:
          tmp_ttestvalues[i] = statSortTestBin("tmpM","tmpP") // Mann-Whitney U Test
          break
       endswitch
        i+=1
   while(i<LengthOfTImeSampling)
   SetScale/P x -FinalValveTime,TemporalResolution, tmp_ttestvalues
   SetScale/P x -FinalValveTime,TemporalResolution, tmp_equalVariance
   duplicate /O tmp_ttestvalues, tmp
   tmp=tmp_ttestvalues[LengthOfTimeSampling-p]
```

```
   findlevel /Q tmp, SignificanceThreshold
   FirstDetect = TemporalResolution*LengthOfTimeSampling-(V_levelx+FinalValveTime)-
FinalValveTime
   if (V_flag)
        FirstDetect = TemporalResolution*LengthOfTimeSampling-FinalValveTime
   endif
   print numPTrials, numMTrials
   print num2P, num2M

END

#pragma rtGlobals=1      // Use modern global access method.

Function GetSamplePattern()
   string /g IndividualTaskName, SPECIFICWAVEENDING
   variable /g CurrentTask, WhichAnimal, LengthOfTimeSampling, NumberOfTasks,
MAXVALVNUMBER
   variable /g startTrials, endTrials, CountOnlyCorrect
   duplicate /o /T $IndividualTaskName, tmpWave1
   variable i, tmpLogic,j, numPTrials=0, numMTrials=0, ToBeCountedAsCorrect
   string PathSpecifier
   make /N=(NumberOfTasks) /O NumSPlusValves, NumSMinusValves
   make /O /N=(LengthOfTimeSampling) tmp_SamplingPWave=0,tmp_SamplingMWave=0
   make /O /N=(LengthOfTimeSampling) LickPatternTmp
   make /O OdorValve, SamplePattern
   make /N=(NumSPlusValves[CurrentTask-1]) /O SPlusValvesTmp
   make /N=(NumSMinusValves[CurrentTask-1]) /O SMinusValvesTmp
   make /N=(NumberOfTasks,MAXVALVNUMBER) /O SPlusValves
   make /N=(NumberOfTasks,MAXVALVNUMBER) /O SMinusValves

   SPlusValvesTmp=SPlusValves[CurrentTask-1][p]
   SMinusValvesTmp=SMinusValves[CurrentTask-1][p]

   PathSpecifier=tmpWave1[WhichAnimal-1][CurrentTask-1]
   killwaves OdorValve, SamplePattern
   LoadWave/H/O/P=DataPath/K=0 ":"+PathSpecifier+"
Folder:samplePattern."+SPECIFICWAVEENDING
   LoadWave/H/O/P=DataPath/K=0 ":"+PathSpecifier+"
Folder:OdorValve."+SPECIFICWAVEENDING
   LoadWave/H/O/P=DataPath/K=0 ":"+PathSpecifier+"
Folder:lickPattern."+SPECIFICWAVEENDING; wave lickpattern=lickpattern
   i=startTrials
   do
       ToBeCountedAsCorrect=0.5
       if(CountOnlyCorrect)
     LickPatternTmp=LickPattern[i][p]
            ToBeCountedAsCorrect=IsCriterion()
       endif
    j=0;tmpLogic=0      // next couple of lines assess whether trial i was any of the s+
trials
```

```
        do
          tmpLogic+=(OdorValve[i]==SPlusValvesTmp[j])
          j+=1
        while(j<NumSPlusValves[CurrentTask-1])
        if(tmpLogic*ToBeCountedAsCorrect)
          tmp_SamplingPWave+=SamplePattern[i][p]
          numPTrials+=1
        endif
        j=0;tmpLogic=0      // next couple of lines assess whether tril i was any of the s- trials
        do
          tmpLogic+=(OdorValve[i]==SMinusValvesTmp[j])
          j+=1
        while(j<NumSMinusValves[CurrentTask-1])
        if(tmpLogic*(1-ToBeCountedAsCorrect))
          tmp_SamplingMWave+=SamplePattern[i][p]
          numMTrials+=1
        endif
        i+=1
    while(i<EndTrials)
  print "Trials : ", numpTrials, numMTrials
    tmp_SamplingPWave/=NumPTrials
    tmp_SamplingMWave/=NumMTrials
END
//      +       +       -       = M1                            thick
//      -       +       -       = M2                            middle
//      +       -       -       = M3                            thin
//      -       -       -       = M4 (does not exist)           dotted

//      +       +       +       = P1 (does not exist, cntrl)
//      -       +       +       = P2
//      +       -       +       = P3
//      -       -       +       = P4




Function GetSamplePatternSeparate()
    string /g IndividualTaskName, SPECIFICWAVEENDING
    variable /g CurrentTask, WhichAnimal, LengthOfTimeSampling, NumberOfTasks,
MAXVALVNUMBER
    variable /g startTrials, endTrials, CountOnlyCorrect
    duplicate /o /T $IndividualTaskName, tmpWave1
    variable i, tmpLogic,j, numP1Trials=0, numP2Trials=0, numP3Trials=0, numP4Trials=0,
numM1Trials=0,numM2Trials=0,numM3Trials=0,numM4Trials=0, IsPLusLast,IsMinusLast,
IsPLusSecondLast, IsMinusSecondLast
    string PathSpecifier
    make /N=(NumberOfTasks) /O NumSPlusValves, NumSMinusValves
    make /O /N=(LengthOfTimeSampling) tmp_SamplingP1Wave=0,
tmp_SamplingP2Wave=0, tmp_SamplingP3Wave=0,
```

```
tmp_SamplingP4Wave=0,tmp_SamplingM1Wave=0,tmp_SamplingM2Wave=0,tmp_Samp
lingM3Wave=0,tmp_SamplingM4Wave=0
   make /O /N=(LengthOfTimeSampling) LickPatternTmp
   make /O OdorValve, SamplePattern
   make /N=(NumSPlusValves[CurrentTask-1]) /O SPlusValvesTmp
   make /N=(NumSMinusValves[CurrentTask-1]) /O SMinusValvesTmp
   make /N=(NumberOfTasks,MAXVALVNUMBER) /O SPlusValves
   make /N=(NumberOfTasks,MAXVALVNUMBER) /O SMinusValves

   SPlusValvesTmp=SPlusValves[CurrentTask-1][p]
   SMinusValvesTmp=SMinusValves[CurrentTask-1][p]

   PathSpecifier=tmpWave1[WhichAnimal-1][CurrentTask-1]
   killwaves OdorValve, SamplePattern
   LoadWave/H/O/P=DataPath/K=0 ":"+PathSpecifier+"
Folder:samplePattern."+SPECIFICWAVEENDING
   LoadWave/H/O/P=DataPath/K=0 ":"+PathSpecifier+"
Folder:OdorValve."+SPECIFICWAVEENDING
   LoadWave/H/O/P=DataPath/K=0 ":"+PathSpecifier+"
Folder:lickPattern."+SPECIFICWAVEENDING; wave lickpattern=lickpattern
   i=startTrials+2
   do
      j=0;IsPLusLast=0      // next couple of lines assess whether tril i-1 was any of the s+
trials
      do
        IsPLusLast+=(OdorValve[i-1]==SPlusValvesTmp[j])
        j+=1
      while(j<NumSPlusValves[CurrentTask-1])
      j=0;IsMinusLast=0      // next couple of lines assess whether tril i-1 was any of the s-
trials
      do
        IsMinusLast+=(OdorValve[i-1]==SMinusValvesTmp[j])
        j+=1
      while(j<NumSMinusValves[CurrentTask-1])
      j=0;IsPLusSecondLast=0      // next couple of lines assess whether tril i-2 was any of
the s+ trials
      do
        IsPLusSecondLast+=(OdorValve[i-2]==SPlusValvesTmp[j])
        j+=1
      while(j<NumSPlusValves[CurrentTask-1])

      j=0;IsMinusSecondLast=0      // next couple of lines assess whether tril i-2 was any of
the s+ trials
      do
        IsMinusSecondLast+=(OdorValve[i-2]==SMinusValvesTmp[j])
        j+=1
      while(j<NumSMinusValves[CurrentTask-1])
```

```
	j=0;tmpLogic=0	// next couple of lines assess whether trial i was any of the s+
trials
	do
	  tmpLogic+=(OdorValve[i]==SPlusValvesTmp[j])
	  j+=1
	while(j<NumSPlusValves[CurrentTask-1])
	 if(tmpLogic)
	  if(IsPlusLast)
	    if(IsPLusSecondLast)
	   tmp_SamplingP1Wave+=SamplePattern[i][p]
	   numP1Trials+=1
	    elseif(IsMinusSecondLast)
	   tmp_SamplingP2Wave+=SamplePattern[i][p]
	   numP2Trials+=1
	    endif
	  elseif(IsMinusLast)  // last one was SMinus
	    if(IsPLusSecondLast)
	   tmp_SamplingP3Wave+=SamplePattern[i][p]
	   numP3Trials+=1
	    elseif(IsMinusSecondLast)
	   tmp_SamplingP4Wave+=SamplePattern[i][p]
	   numP4Trials+=1
	    endif
	  endif
	endif



	j=0;tmpLogic=0	// next couple of lines assess whether tril i was any of the s- trials
	do
	  tmpLogic+=(OdorValve[i]==SMinusValvesTmp[j])
	  j+=1
	while(j<NumSMinusValves[CurrentTask-1])
	 if(tmpLogic)
	  if(IsPlusLast)
	    if(IsPLusSecondLast)
	   tmp_SamplingM1Wave+=SamplePattern[i][p]
	   numM1Trials+=1
	    elseif(IsMinusSecondLast)
	   tmp_SamplingM2Wave+=SamplePattern[i][p]
	   numM2Trials+=1
	    endif
	  elseif(IsMinusLast)  // last one was SMinus
	    if(IsPLusSecondLast)
	   tmp_SamplingM3Wave+=SamplePattern[i][p]
	   numM3Trials+=1
	    elseif(IsMinusSecondLast)
	   tmp_SamplingM4Wave+=SamplePattern[i][p]
	   numM4Trials+=1
	    endif
```

```
         endif
       endif
       i+=1
   while(i<EndTrials)
  print "Trials : ", nump1Trials,nump2Trials,nump3Trials,nump4Trials,
numM1Trials,numM2Trials,numM3Trials,numM4Trials
   tmp_SamplingP1Wave/=NumP1Trials
   tmp_SamplingP2Wave/=NumP2Trials
   tmp_SamplingP3Wave/=NumP3Trials
   tmp_SamplingP4Wave/=NumP4Trials
   tmp_SamplingM1Wave/=NumM1Trials
   tmp_SamplingM2Wave/=NumM2Trials
   tmp_SamplingM3Wave/=NumM3Trials
   tmp_SamplingM4Wave/=NumM4Trials
END


Function ButtonProc_IndDisplayAndLayout(ctrlName) : ButtonControl
   String ctrlName
   string /g IndividualTaskName
   string /g RecentDisplayWindowsList=""
   variable /g StartTask, EndTask, CurrentTask, WhichAnimalStart, WhichAnimalStop,
WhichAnimal,MAXDISPLAYEDPERTASK
   variable /g LengthOfTimeSampling, NumberOfTasks, NumberOfAnimals,
TrialsAveraged,FinalValveTime,TemporalResolution
   variable /g startTrials=0, endTrials=199
   variable /g DispPercCorr, DispMax, DispAvg, DispFitMax, DispFitSlope, DispFitHalf,
DispSingle, DispPM, DispFirstDetect,DispSelectPPM
   variable /g DispLSingle, DispLPM, DispLFirstDetect
   variable /g FirstDetect
   make /N=(NumberOfTasks) /O TrialsPerTask
   make /O W_coef, W_Sigma
   make /O /N=(LengthOfTimeSampling)
tmp_SamplingPWave,tmp_SamplingMWave,tmp_LickPWave,tmp_LickMWave
   make /N=(NumberOfTasks) /O TrialsPerTask, FinalValveTimeArray

   make /N=(NumberOfAnimals+1) /O GroupOfAnimal
   variable /g MAXNUMBEROFGROUPS
   make /N=(MAXNUMBEROFGROUPS) /O
GROUPCOLORRED,GROUPCOLORGREEN,GROUPCOLORBLUE

   variable i,j,tmpLogic,numPTrials=0, numMTrials=0, numberOfDataPoints=0,k
   variable WaveEntryIndex     // running from 0 to NumberOfDataPoints-1
   // calculate number of data points in output waves (sampling rate (e.g. 100 trials)
summed up over tasks)
   i=StartTask-1
   do
      numberOfDataPoints+=round(TrialsPerTask[i]/TrialsAveraged+0.49)     // if trials are
averaged in 100 a piece but only 250 trials -> nevertheless three entries
      i+=1
```

```
    while(i<EndTask-1+0.1)
    print NumberOfDataPoints
//    i=0
//    do
//        DoWindow/K $("SamplingDiffExmpl"+num2str(i))
//        i+=1
//    while(i<NumberOfAnimals)
//    j=0
//    do
//        i=0
//        do
//            k=0
//            do
//                DoWindow/K $("SamplingExmpl"+Num2str(i)+"_"+num2str(j)+"_"+num2str(k))
//                    DoWindow/K $("SamplSepEx"+Num2str(i)+"_"+num2str(j)+"_"+num2str(k))
//                k+=1
//            while(k<MAXDISPLAYEDPERTASK)
//            i+=1
//        while(i<NumberOfTasks+1)
//        j+=1
//    while(j<NumberOfAnimals+1)
//
//    DoWindow /K SamplingDiffExmpl
//    DoWindow /K FittedMaximaTmp
//    DoWindow /K FittedSlopeTmp
//    DoWindow /K FittedHalfTmp
//    DoWindow /K AvgDiffTmp
//    DoWindow /K FirstDetectTmp
//

    if(DispPercCorr)
        DisplayPercentageCorrect()
    endif

    if(DispFitMax)
        DoWindow/K FittedMaximaTmp
        display /W=(3,40,220,120)/K=1 as "Fitted Maxima"
//      SetAxis left 0.5,1
        DoWindow/C FittedMaximaTmp
        ModifyGraph width=150,height=30
        RecentDisplayWindowsList=RecentDisplayWindowsList+"FittedMaximaTmp;"
    endif
    if(DispFitSlope)
        DoWindow/K FittedSlopeTmp
        display /W=(3,140,220,220)/K=1 as "Fitted Slope"
        DoWindow/C FittedSlopeTmp
        ModifyGraph width=150,height=30
        RecentDisplayWindowsList=RecentDisplayWindowsList+"FittedSlopeTmp;"
    endif
    if(DispFitHalf)
```

```
      DoWindow/K FittedHalfTmp
      display /W=(3,240,220,320)/K=1 as "Fitted HalfMaximum"
      DoWindow/C FittedHalfTmp
      ModifyGraph width=150,height=30
      RecentDisplayWindowsList=RecentDisplayWindowsList+"FittedHalfTmp;"
   endif
   if(DispAvg)
      DoWindow/K AvgDiffTmp
      display /W=(3,340,220,420)/K=1 as "Average Difference"
      DoWindow/C AvgDiffTmp
      ModifyGraph width=150,height=30
      RecentDisplayWindowsList=RecentDisplayWindowsList+"AvgDiffTmp;"
   endif
   if(DispFirstDetect)
      DoWindow/K FirstDetectTmp
      display /W=(3,340,220,420)/K=1 as "FirstDetect"
      DoWindow/C FirstDetectTmp
      ModifyGraph width=150,height=30
      RecentDisplayWindowsList=RecentDisplayWindowsList+"FirstDetectTmp;"
   endif
   make /N=(numberOfDataPoints) /O tmp_DispFirstDetectWave
   if(DispLFirstDetect)
      DoWindow/K LFirstDetectTmp
      display /W=(3,340,220,420)/K=1 as "LickFirstDetect"
      DoWindow/C LFirstDetectTmp
      ModifyGraph width=150,height=30
      RecentDisplayWindowsList=RecentDisplayWindowsList+"LFirstDetectTmp;"
   endif
   make /N=(numberOfDataPoints) /O tmp_DispLFirstDetectWave
   make /N=(numberOfDataPoints) /O /T WhichIsTheTask

   WhichAnimal = WhichAnimalStart
   do
    if(GroupOfAnimal[WhichANimal]>0)
      if(DispSingle)
         DoWindow/K $("SamplingDiffExmpl"+num2str(WhichAnimal))
         display/K=1  as "Individual Sampling Difference Example Animal
Nr."+num2str(WhichAnimal)
         DoWindow/C $("SamplingDiffExmpl"+num2str(WhichAnimal))
         ModifyGraph width=150,height=30

RecentDisplayWindowsList=RecentDisplayWindowsList+"SamplingDiffExmpl"+num2str(W
hichAnimal)+";"
      endif
      if(DispLSingle)
         DoWindow/K $("LickDiffExmpl"+num2str(WhichAnimal))
         display/K=1  as "Individual Lick Difference Animal Nr."+num2str(WhichAnimal)
         DoWindow/C $("LickDiffExmpl"+num2str(WhichAnimal))
         ModifyGraph width=150,height=30
```

```
RecentDisplayWindowsList=RecentDisplayWindowsList+"LickDiffExmpl"+num2str(WhichA
nimal)+";"
	endif

	CurrentTask=StartTask
	WaveEntryIndex=0

if(DispAvg+DispFitMax+DispFitSlope+DispSingle+DispFirstDetect+DispFitHalf+DispSingle
+DispPM+DispSelectPPM)
		do
		   j=0
		   do
		      FinalValveTime=FinalValveTImeArray[CurrentTask-1]
		      startTrials=j*TrialsAveraged+1
		      endTrials=min(TrialsPerTask[CurrentTask-1],startTrials+TrialsAveraged)
	   // Notebook AnlTmp_NoteBook text="start "+num2str(startTrials)+ ";end :
"+num2str(endTrials) + "; WaveEntryIndex :"+num2str(WaveEntryIndex)+"\r"
		      GetSamplePattern()
		      SetScale/P x -FinalValveTime,TemporalResolution,
tmp_SamplingMWave
		      SetScale/P x -FinalValveTime,TemporalResolution, tmp_SamplingPWave

		      duplicate /O tmp_SamplingPWave,
$("tmp_SamplingPWave"+num2str(CurrentTask)+"_"+num2str(WhichAnimal)+"_"+num2st
r(j))
		      duplicate /O tmp_SamplingMWave,
$("tmp_SamplingMWave"+num2str(CurrentTask)+"_"+num2str(WhichAnimal)+"_"+num2st
r(j))
		      if(DispPM)
		         DoWindow/K
$("SamplingExmpl"+Num2str(CurrentTask)+"_"+num2str(WhichAnimal)+"_"+num2str(j))
		         display /K=1
$("tmp_SamplingPWave"+num2str(CurrentTask)+"_"+num2str(WhichAnimal)+"_"+num2st
r(j)),
$("tmp_SamplingMWave"+num2str(CurrentTask)+"_"+num2str(WhichAnimal)+"_"+num2st
r(j)) as "Task "+num2str(CurrentTask)+" & Animal "+ num2str(WhichAnimal)+" Trials:
"+num2str(j)
		         label bottom "Time after odor onset (ms)"
		         label left "Sampling (in %)"
		         ModifyGraph width=150,height=30
		         DoWindow/C
$("SamplingExmpl"+Num2str(CurrentTask)+"_"+num2str(WhichAnimal)+"_"+num2str(j))
		         ModifyGraph
rgb($("tmp_SamplingPWave"+num2str(CurrentTask)+"_"+num2str(WhichAnimal))+"_"+nu
m2str(j))=(0,65280,0)

RecentDisplayWindowsList=RecentDisplayWindowsList+"SamplingExmpl"+Num2str(Curre
ntTask)+"_"+num2str(WhichAnimal)+"_"+num2str(j)+";"
		      endif
```

```
			duplicate /O tmp_SamplingPWave, tmp_SamplingDiffWave
			tmp_SamplingDiffWave=tmp_SamplingPWave-tmp_SamplingMWave
			duplicate /O tmp_samplingDiffWave,
$("tmp_SamplingDiff"+num2str(CurrentTask)+"_"+num2str(WhichAnimal)+"_"+num2str(j))
			if(DispSingle)
			   appendtograph /W=$("SamplingDiffExmpl"+num2str(WhichAnimal))
$("tmp_SamplingDiff"+num2str(CurrentTask)+"_"+num2str(WhichAnimal)+"_"+num2str(j))
			   label /W=$("SamplingDiffExmpl"+num2str(WhichAnimal))  bottom
"Time after odor onset (ms)"
			   label /W=$("SamplingDiffExmpl"+num2str(WhichAnimal))  left
"Sampling difference (in %Points)"
			   ModifyGraph  /W=$("SamplingDiffExmpl"+num2str(WhichAnimal))
rgb=(0,0,0)
			endif
			K0 = 0;
			CurveFit/H="1000" /Q Sigmoid tmp_SamplingDiffWave /D
			make /N=(numberOfDataPoints) /O
tmp_DispFitMaxWave,tmp_DispFitMaxWaveSD
			tmp_DispFitMaxWave[WaveEntryIndex]=W_coef[1]
			tmp_DispFitMaxWaveSD[WaveEntryIndex]=W_sigma[1]
			if(DispFitMax)
			   print "Max is ", W_coef[1]," +-", W_sigma[1]
			endif

			make /N=(numberOfDataPoints) /O
tmp_DispFitSlopeWave,tmp_DispFitSlopeWaveSD
			tmp_DispFitSlopeWave[WaveEntryIndex]=W_coef[3]
			tmp_DispFitSlopeWaveSD[WaveEntryIndex]=W_sigma[3]
			if(DispFitSlope)
			   print "Slope is ", W_coef[3]," +-", W_sigma[3]
			endif

			make /N=(numberOfDataPoints) /O
tmp_DispFitHalfWave,tmp_DispFitHalfWaveSD
			tmp_DispFitHalfWave[WaveEntryIndex]=W_coef[2]
			tmp_DispFitHalfWaveSD[WaveEntryIndex]=W_sigma[2]
			if(DispFitHalf)
			   print "HalfMax is ", W_coef[2]," +-", W_sigma[2]
			endif
			wavestats /Q tmp_SamplingDiffWave
			make /N=(numberOfDataPoints) /O tmp_DispAvgWave
			tmp_DispAvgWave[WaveEntryIndex]=V_avg
			if(DispAvg)
			   print "Average Difference is ", V_avg
			endif
			if(DispFirstDetect)
			   GetDifferentTValues(DispFirstDetect)
			   tmp_DispFirstDetectWave[WaveEntryIndex]=FirstDetect
			endif
			if(DispSelectPPM)
```

```
				GetSamplePatternSeparate()
				SetScale/P x -FinalValveTime,TemporalResolution,
tmp_SamplingM1Wave
					SetScale/P x -FinalValveTime,TemporalResolution,
tmp_SamplingP1Wave
				SetScale/P x -FinalValveTime,TemporalResolution,
tmp_SamplingM2Wave
					SetScale/P x -FinalValveTime,TemporalResolution,
tmp_SamplingP2Wave
				SetScale/P x -FinalValveTime,TemporalResolution,
tmp_SamplingM3Wave
					SetScale/P x -FinalValveTime,TemporalResolution,
tmp_SamplingP3Wave
				SetScale/P x -FinalValveTime,TemporalResolution,
tmp_SamplingM4Wave
					SetScale/P x -FinalValveTime,TemporalResolution,
tmp_SamplingP4Wave

					duplicate /O tmp_SamplingP1Wave,
$("tmp_SamplingP1Wave"+num2str(CurrentTask)+"_"+num2str(WhichAnimal)+"_"+num2s
tr(j))
					duplicate /O tmp_SamplingP2Wave,
$("tmp_SamplingP2Wave"+num2str(CurrentTask)+"_"+num2str(WhichAnimal)+"_"+num2s
tr(j))
					duplicate /O tmp_SamplingP3Wave,
$("tmp_SamplingP3Wave"+num2str(CurrentTask)+"_"+num2str(WhichAnimal)+"_"+num2s
tr(j))
					duplicate /O tmp_SamplingP4Wave,
$("tmp_SamplingP4Wave"+num2str(CurrentTask)+"_"+num2str(WhichAnimal)+"_"+num2s
tr(j))
				duplicate /O tmp_SamplingM1Wave,
$("tmp_SamplingM1Wave"+num2str(CurrentTask)+"_"+num2str(WhichAnimal)+"_"+num2
str(j))
				duplicate /O tmp_SamplingM2Wave,
$("tmp_SamplingM2Wave"+num2str(CurrentTask)+"_"+num2str(WhichAnimal)+"_"+num2
str(j))
				duplicate /O tmp_SamplingM3Wave,
$("tmp_SamplingM3Wave"+num2str(CurrentTask)+"_"+num2str(WhichAnimal)+"_"+num2
str(j))
				duplicate /O tmp_SamplingM4Wave,
$("tmp_SamplingM4Wave"+num2str(CurrentTask)+"_"+num2str(WhichAnimal)+"_"+num2
str(j))

				DoWindow/K
$("SamplSepEx"+Num2str(CurrentTask)+"_"+num2str(WhichAnimal)+"_"+num2str(j))
				display /K=1
$("tmp_SamplingP1Wave"+num2str(CurrentTask)+"_"+num2str(WhichAnimal)+"_"+num2s
tr(j)),
$("tmp_SamplingM1Wave"+num2str(CurrentTask)+"_"+num2str(WhichAnimal)+"_"+num2
```

```
str(j)) as "Task "+num2str(CurrentTask)+" & Animal "+ num2str(WhichAnimal)+" Trials: "+num2str(j)
			appendtograph $("tmp_SamplingM3Wave"+num2str(CurrentTask)+"_"+num2str(WhichAnimal)+"_"+num2str(j)), $("tmp_SamplingM2Wave"+num2str(CurrentTask)+"_"+num2str(WhichAnimal)+"_"+num2str(j))
			appendtograph $("tmp_SamplingM4Wave"+num2str(CurrentTask)+"_"+num2str(WhichAnimal)+"_"+num2str(j)), $("tmp_SamplingP2Wave"+num2str(CurrentTask)+"_"+num2str(WhichAnimal)+"_"+num2str(j))
			appendtograph $("tmp_SamplingP4Wave"+num2str(CurrentTask)+"_"+num2str(WhichAnimal)+"_"+num2str(j)), $("tmp_SamplingP3Wave"+num2str(CurrentTask)+"_"+num2str(WhichAnimal)+"_"+num2str(j))
			label bottom "Time after odor onset (ms)"
			label left "Sampling (in %)"
			ModifyGraph width=150,height=30
			DoWindow/C $("SamplSepEx"+Num2str(CurrentTask)+"_"+num2str(WhichAnimal)+"_"+num2str(j))
			ModifyGraph lsize($("tmp_SamplingM1Wave"+num2str(CurrentTask)+"_"+num2str(WhichAnimal))+"_"+num2str(j))=3
			ModifyGraph lsize($("tmp_SamplingM2Wave"+num2str(CurrentTask)+"_"+num2str(WhichAnimal))+"_"+num2str(j))=2
			ModifyGraph lstyle($("tmp_SamplingM4Wave"+num2str(CurrentTask)+"_"+num2str(WhichAnimal))+"_"+num2str(j))=2

			ModifyGraph rgb($("tmp_SamplingP1Wave"+num2str(CurrentTask)+"_"+num2str(WhichAnimal))+"_"+num2str(j))=(0,65280,0)
			ModifyGraph rgb($("tmp_SamplingP2Wave"+num2str(CurrentTask)+"_"+num2str(WhichAnimal))+"_"+num2str(j))=(0,65280,0)
			ModifyGraph rgb($("tmp_SamplingP4Wave"+num2str(CurrentTask)+"_"+num2str(WhichAnimal))+"_"+num2str(j))=(0,65280,0)
			ModifyGraph rgb($("tmp_SamplingP3Wave"+num2str(CurrentTask)+"_"+num2str(WhichAnimal))+"_"+num2str(j))=(0,65280,0)
			ModifyGraph lsize($("tmp_SamplingP1Wave"+num2str(CurrentTask)+"_"+num2str(WhichAnimal))+"_"+num2str(j))=3
			ModifyGraph lsize($("tmp_SamplingP2Wave"+num2str(CurrentTask)+"_"+num2str(WhichAnimal))+"_"+num2str(j))=2
```

```
                    ModifyGraph
lstyle($("tmp_SamplingP4Wave"+num2str(CurrentTask)+"_"+num2str(WhichAnimal))+"_"+
num2str(j))=2

RecentDisplayWindowsList=RecentDisplayWindowsList+"SamplSepEx"+Num2str(Current
Task)+"_"+num2str(WhichAnimal)+"_"+num2str(j)+";"
                 endif
                    WhichIsTheTask[WaveEntryIndex]="Task "+Num2str(CurrentTask)+";
No "+Num2str(j)
                 j+=1
                 WaveEntryIndex+=1
               while(j<round(TrialsPerTask[CurrentTask-1]/TrialsAveraged+0.49))
               CurrentTask+=1
             while(CurrentTask<EndTask+0.1)
          endif
      // Now duplicate the generic results from Animal <WHICHANIMAL> to a more general
wave:
      duplicate /o  tmp_DispFitMaxWave,
$("tmp_DispFitMaxWave"+num2str(WhichAnimal))
      duplicate /o  tmp_DispFitMaxWaveSD,
$("tmp_DispFitMaxWaveSD"+num2str(WhichAnimal))
      duplicate /o  tmp_DispFitHalfWave,
$("tmp_DispFitHalfWave"+num2str(WhichAnimal))
      duplicate /o  tmp_DispFitHalfWaveSD,
$("tmp_DispFitHalfWaveSD"+num2str(WhichAnimal))
      duplicate /o  tmp_DispFitSlopeWave,
$("tmp_DispFitSlopeWave"+num2str(WhichAnimal))
      duplicate /o  tmp_DispFitSlopeWaveSD,
$("tmp_DispFitSlopeWaveSD"+num2str(WhichAnimal))
      duplicate /o  tmp_DispAvgWave,$("tmp_AvgDiffWave"+num2str(WhichAnimal))
      duplicate /o
tmp_DispFirstDetectWave,$("tmp_DispFirstDetectWave"+num2str(WhichAnimal))

      if(DispFitMax)
         SetScale/P x 1,1,"", $("tmp_DispFitMaxWave"+num2str(WhichAnimal))
         appendtograph /W=FittedMaximaTmp
$("tmp_DispFitMaxWave"+num2str(WhichAnimal))
         ModifyGraph  /W=FittedMaximaTmp
rgb($("tmp_DispFitMaxWave"+num2str(WhichAnimal)))=(GROUPCOLORRED[GroupOfAn
imal[WhichAnimal]],GROUPCOLORGREEN[GroupOfAnimal[WhichAnimal]],GROUPCOL
ORBLUE[GroupOfAnimal[WhichAnimal]])
         ErrorBars /W=FittedMaximaTmp
$("tmp_DispFitMaxWave"+num2str(WhichAnimal))
Y,wave=($("tmp_DispFitMaxWaveSD"+num2str(WhichAnimal)),$("tmp_DispFitMaxWaveS
D"+num2str(WhichAnimal)))
   //    SetAxis /W=FittedMaximaTmp left 0.5,1
         Label  /W=FittedMaximaTmp left "Fit Max";Label /W=FittedMaximaTmp bottom
"Task number"
         SetAxis /W=FittedMaximaTmp Bottom 1, numberOfDataPoints
         ModifyGraph /W=FittedMaximaTmp nticks(bottom)=min(20,numberOfDataPoints)
```

```
        endif
        if(DispFitSlope)
           SetScale/P x 1,1,"", $("tmp_DispFitSlopeWave"+num2str(WhichAnimal))
           appendtograph /W=FittedSlopeTmp
$("tmp_DispFitSlopeWave"+num2str(WhichAnimal))
           ModifyGraph  /W=FittedSlopeTmp
rgb($("tmp_DispFitSlopeWave"+num2str(WhichAnimal)))=(GROUPCOLORRED[GroupOf
Animal[WhichAnimal]],GROUPCOLORGREEN[GroupOfAnimal[WhichAnimal]],GROUPCO
LORBLUE[GroupOfAnimal[WhichAnimal]])
           ErrorBars /W=FittedSlopeTmp $("tmp_DispFitSlopeWave"+num2str(WhichAnimal))
Y,wave=($("tmp_DispFitSlopeWaveSD"+num2str(WhichAnimal)),$("tmp_DispFitSlopeWav
eSD"+num2str(WhichAnimal)))
    //     SetAxis /W=FittedSlopeTmp left 0,1
           Label /W=FittedSlopeTmp  left "Fit Slope";Label /W=FittedSlopeTmp bottom "Task
number"
           SetAxis /W=FittedSlopeTmp Bottom 1, numberOfDataPoints
           ModifyGraph /W=FittedSlopeTmp nticks(bottom)=min(20,numberOfDataPoints)
        endif
        if(DispFitHalf)
           SetScale/P x 1,1,"",  $("tmp_DispFitHalfWave"+num2str(WhichAnimal))
           appendtograph /W=FittedHalfTmp
$("tmp_DispFitHalfWave"+num2str(WhichAnimal))
           ModifyGraph  /W=FittedHalfTmp
rgb($("tmp_DispFitHalfWave"+num2str(WhichAnimal)))=(GROUPCOLORRED[GroupOfAn
imal[WhichAnimal]],GROUPCOLORGREEN[GroupOfAnimal[WhichAnimal]],GROUPCOL
ORBLUE[GroupOfAnimal[WhichAnimal]])
           ErrorBars /W=FittedHalfTmp  $("tmp_DispFitHalfWave"+num2str(WhichAnimal))
Y,wave=($("tmp_DispFitHalfWaveSD"+num2str(WhichAnimal)),
$("tmp_DispFitHalfWaveSD"+num2str(WhichAnimal)))
    //     SetAxis  /W=FittedHalfTmp left 0,1
           Label /W=FittedHalfTmp left "Fit Half";Label /W=FittedHalfTmp bottom "Task
number"
           SetAxis  /W=FittedHalfTmp Bottom 1,numberOfDataPoints
           ModifyGraph  /W=FittedHalfTmp nticks(bottom)=min(20,numberOfDataPoints)
        endif
        if(DispAvg)
           SetScale/P x 1,1,"",  $("tmp_AvgDiffWave"+num2str(WhichAnimal))
           appendtograph /W=AvgDiffTmp  $("tmp_AvgDiffWave"+num2str(WhichAnimal))
           ModifyGraph  /W=AvgDiffTmp
rgb($("tmp_AvgDiffWave"+num2str(WhichAnimal)))=(GROUPCOLORRED[GroupOfAnima
l[WhichAnimal]],GROUPCOLORGREEN[GroupOfAnimal[WhichAnimal]],GROUPCOLORB
LUE[GroupOfAnimal[WhichAnimal]])
    //     SetAxis  /W=AvgDiffTmp left 0,1
           Label /W=AvgDiffTmp   left "Avg";Label /W=AvgDiffTmp  bottom "Task number"
           SetAxis  /W=AvgDiffTmp Bottom 1, numberOfDataPoints
           ModifyGraph  /W=AvgDiffTmp nticks(bottom)=min(20,numberOfDataPoints)
        endif
        if(DispFirstDetect)
           SetScale/P x 1,1,"",  $("tmp_DispFirstDetectWave"+num2str(WhichAnimal))
```

```
         appendtograph /W=FirstDetectTmp
$("tmp_DispFirstDetectWave"+num2str(WhichAnimal))
         ModifyGraph  /W=FirstDetectTmp
rgb($("tmp_DispFirstDetectWave"+num2str(WhichAnimal)))=(GROUPCOLORRED[Group
OfAnimal[WhichAnimal]],GROUPCOLORGREEN[GroupOfAnimal[WhichAnimal]],GROUP
COLORBLUE[GroupOfAnimal[WhichAnimal]])
         Label /W=FirstDetectTmp   left "FirstDetect(ms)";Label /W=FirstDetectTmp  bottom
"Task number"
         SetAxis  /W=FirstDetectTmp Bottom 1, numberOfDataPoints
         ModifyGraph  /W=FirstDetectTmp nticks(bottom)=min(20,numberOfDataPoints)
      endif
//
*****************************************************************************************************
*********************
//
*****************************************************************************************************
*********************
// up to here : sample / head analysis
// now: lick analysis
//
*****************************************************************************************************
*********************
//
*****************************************************************************************************
*********************


      CurrentTask=StartTask
      WaveEntryIndex=0
          if(DispLSingle+DispLFirstDetect+DispLPM)
               do
                  j=0
                  do
                     FinalValveTime=FinalValveTImeArray[CurrentTask-1]
                     startTrials=j*TrialsAveraged+1
                     endTrials=min(TrialsPerTask[CurrentTask-1],startTrials+TrialsAveraged)
                     GetLickPattern()
                     SetScale/P x -FinalValveTime,TemporalResolution, tmp_LickMWave
                     SetScale/P x -FinalValveTime,TemporalResolution, tmp_LickPWave

                     duplicate /O tmp_LickPWave,
$("tmp_LickPWave"+num2str(CurrentTask)+"_"+num2str(WhichAnimal)+"_"+num2str(j))
                     duplicate /O tmp_LickMWave,
$("tmp_LickMWave"+num2str(CurrentTask)+"_"+num2str(WhichAnimal)+"_"+num2str(j))
                     if(DispLPM)
                        DoWindow/K
$("LickExmpl"+Num2str(CurrentTask)+"_"+num2str(WhichAnimal)+"_"+num2str(j))
                        display /K=1
$("tmp_LickPWave"+num2str(CurrentTask)+"_"+num2str(WhichAnimal)+"_"+num2str(j)),
$("tmp_LickMWave"+num2str(CurrentTask)+"_"+num2str(WhichAnimal)+"_"+num2str(j))
```

```
as "Task "+num2str(CurrentTask)+" & Animal "+ num2str(WhichAnimal)+" Trials: 
"+num2str(j)
                label bottom "Time after odor onset (ms)"
                label left "Lick (in %)"
                ModifyGraph width=150,height=30
                DoWindow/C 
$("LickExmpl"+Num2str(CurrentTask)+"_"+num2str(WhichAnimal)+"_"+num2str(j))
                ModifyGraph 
rgb($("tmp_LickPWave"+num2str(CurrentTask)+"_"+num2str(WhichAnimal))+"_"+num2str
(j))=(0,65280,0)

RecentDisplayWindowsList=RecentDisplayWindowsList+"LickExmpl"+Num2str(CurrentTa
sk)+"_"+num2str(WhichAnimal)+"_"+num2str(j)+";"
            endif
            duplicate /O tmp_LickPWave, tmp_LickDiffWave
            tmp_LickDiffWave=tmp_LickPWave-tmp_LickMWave
            duplicate /O tmp_LickDiffWave, 
$("tmp_LickDiff"+num2str(CurrentTask)+"_"+num2str(WhichAnimal)+"_"+num2str(j))
            if(DispLSingle)
                appendtograph /W=$("LickDiffExmpl"+num2str(WhichAnimal)) 
$("tmp_LickDiff"+num2str(CurrentTask)+"_"+num2str(WhichAnimal)+"_"+num2str(j))
                label /W=$("LickDiffExmpl"+num2str(WhichAnimal))  bottom "Time 
after odor onset (ms)"
                label /W=$("LickDiffExmpl"+num2str(WhichAnimal))  left "Lick 
difference (in %Points)"
                ModifyGraph  /W=$("LickDiffExmpl"+num2str(WhichAnimal)) 
rgb=(0,0,0)
            endif
//          K0 = 0;
//          CurveFit/H="1000" /Q Sigmoid tmp_LickDiffWave /D
//          make /N=(numberOfDataPoints) /O 
tmp_DispFitMaxWave,tmp_DispFitMaxWaveSD
//          tmp_DispFitMaxWave[WaveEntryIndex]=W_coef[1]
//          tmp_DispFitMaxWaveSD[WaveEntryIndex]=W_sigma[1]
//          if(DispFitMax)
//              print "Max is ", W_coef[1]," +-", W_sigma[1]
//          endif
//
//          make /N=(numberOfDataPoints) /O 
tmp_DispFitSlopeWave,tmp_DispFitSlopeWaveSD
//          tmp_DispFitSlopeWave[WaveEntryIndex]=W_coef[3]
//          tmp_DispFitSlopeWaveSD[WaveEntryIndex]=W_sigma[3]
//          if(DispFitSlope)
//              print "Slope is ", W_coef[3]," +-", W_sigma[3]
//          endif
//
//          make /N=(numberOfDataPoints) /O 
tmp_DispFitHalfWave,tmp_DispFitHalfWaveSD
//          tmp_DispFitHalfWave[WaveEntryIndex]=W_coef[2]
//          tmp_DispFitHalfWaveSD[WaveEntryIndex]=W_sigma[2]
```

```
//			if(DispFitHalf)
//			   print "HalfMax is ", W_coef[2]," +-", W_sigma[2]
//			endif
//			wavestats /Q tmp_LickDiffWave
//			make /N=(numberOfDataPoints) /O tmp_DispAvgWave
//			tmp_DispAvgWave[WaveEntryIndex]=V_avg
//			if(DispAvg)
//			   print "Average Difference is ", V_avg
//			endif
			if(DispLFirstDetect)
			   GetDifferentTValuesLick(DispLFirstDetect)
			   tmp_DispFirstDetectWave[WaveEntryIndex]=FirstDetect
			endif
//			if(DispSelectPPM)
//			   GetSamplePatternSeparate()
//			   SetScale/P x -FinalValveTime,TemporalResolution, tmp_LickM1Wave
//			      SetScale/P x -FinalValveTime,TemporalResolution,
tmp_LickP1Wave
//			   SetScale/P x -FinalValveTime,TemporalResolution, tmp_LickM2Wave
//			      SetScale/P x -FinalValveTime,TemporalResolution,
tmp_LickP2Wave
//			   SetScale/P x -FinalValveTime,TemporalResolution, tmp_LickM3Wave
//			      SetScale/P x -FinalValveTime,TemporalResolution,
tmp_LickP3Wave
//			   SetScale/P x -FinalValveTime,TemporalResolution, tmp_LickM4Wave
//			      SetScale/P x -FinalValveTime,TemporalResolution,
tmp_LickP4Wave
//
//			      duplicate /O tmp_LickP1Wave,
$("tmp_LickP1Wave"+num2str(CurrentTask)+"_"+num2str(WhichAnimal)+"_"+num2str(j))
//			      duplicate /O tmp_LickP2Wave,
$("tmp_LickP2Wave"+num2str(CurrentTask)+"_"+num2str(WhichAnimal)+"_"+num2str(j))
//			      duplicate /O tmp_LickP3Wave,
$("tmp_LickP3Wave"+num2str(CurrentTask)+"_"+num2str(WhichAnimal)+"_"+num2str(j))
//			      duplicate /O tmp_LickP4Wave,
$("tmp_LickP4Wave"+num2str(CurrentTask)+"_"+num2str(WhichAnimal)+"_"+num2str(j))
//			  duplicate /O tmp_LickM1Wave,
$("tmp_LickM1Wave"+num2str(CurrentTask)+"_"+num2str(WhichAnimal)+"_"+num2str(j))
//			  duplicate /O tmp_LickM2Wave,
$("tmp_LickM2Wave"+num2str(CurrentTask)+"_"+num2str(WhichAnimal)+"_"+num2str(j))
//			  duplicate /O tmp_LickM3Wave,
$("tmp_LickM3Wave"+num2str(CurrentTask)+"_"+num2str(WhichAnimal)+"_"+num2str(j))
//			  duplicate /O tmp_LickM4Wave,
$("tmp_LickM4Wave"+num2str(CurrentTask)+"_"+num2str(WhichAnimal)+"_"+num2str(j))
//
//			   DoWindow/K
$("SamplSepEx"+Num2str(CurrentTask)+"_"+num2str(WhichAnimal)+"_"+num2str(j))
//			   display /K=1
$("tmp_LickP1Wave"+num2str(CurrentTask)+"_"+num2str(WhichAnimal)+"_"+num2str(j)),
$("tmp_LickM1Wave"+num2str(CurrentTask)+"_"+num2str(WhichAnimal)+"_"+num2str(j))
```

```
as "Task "+num2str(CurrentTask)+" & Animal "+ num2str(WhichAnimal)+" Trials:
"+num2str(j)
//			appendtograph
$("tmp_LickM3Wave"+num2str(CurrentTask)+"_"+num2str(WhichAnimal)+"_"+num2str(j)),
$("tmp_LickM2Wave"+num2str(CurrentTask)+"_"+num2str(WhichAnimal)+"_"+num2str(j))
//			appendtograph
$("tmp_LickM4Wave"+num2str(CurrentTask)+"_"+num2str(WhichAnimal)+"_"+num2str(j)),
$("tmp_LickP2Wave"+num2str(CurrentTask)+"_"+num2str(WhichAnimal)+"_"+num2str(j))
//			appendtograph
$("tmp_LickP4Wave"+num2str(CurrentTask)+"_"+num2str(WhichAnimal)+"_"+num2str(j)),
$("tmp_LickP3Wave"+num2str(CurrentTask)+"_"+num2str(WhichAnimal)+"_"+num2str(j))
//			label bottom "Time after odor onset (ms)"
//			label left "Lick (in %)"
//			ModifyGraph width=150,height=30
//			DoWindow/C
$("SamplSepEx"+Num2str(CurrentTask)+"_"+num2str(WhichAnimal)+"_"+num2str(j))
//			ModifyGraph
lsize($("tmp_LickM1Wave"+num2str(CurrentTask)+"_"+num2str(WhichAnimal))+"_"+num2
str(j))=3
//			ModifyGraph
lsize($("tmp_LickM2Wave"+num2str(CurrentTask)+"_"+num2str(WhichAnimal))+"_"+num2
str(j))=2
//			ModifyGraph
lstyle($("tmp_LickM4Wave"+num2str(CurrentTask)+"_"+num2str(WhichAnimal))+"_"+num
2str(j))=2
//
//			ModifyGraph
rgb($("tmp_LickP1Wave"+num2str(CurrentTask)+"_"+num2str(WhichAnimal))+"_"+num2st
r(j))=(0,65280,0)
//			ModifyGraph
rgb($("tmp_LickP2Wave"+num2str(CurrentTask)+"_"+num2str(WhichAnimal))+"_"+num2st
r(j))=(0,65280,0)
//			ModifyGraph
rgb($("tmp_LickP4Wave"+num2str(CurrentTask)+"_"+num2str(WhichAnimal))+"_"+num2st
r(j))=(0,65280,0)
//			ModifyGraph
rgb($("tmp_LickP3Wave"+num2str(CurrentTask)+"_"+num2str(WhichAnimal))+"_"+num2st
r(j))=(0,65280,0)
//			ModifyGraph
lsize($("tmp_LickP1Wave"+num2str(CurrentTask)+"_"+num2str(WhichAnimal))+"_"+num2
str(j))=3
//			ModifyGraph
lsize($("tmp_LickP2Wave"+num2str(CurrentTask)+"_"+num2str(WhichAnimal))+"_"+num2
str(j))=2
//			ModifyGraph
lstyle($("tmp_LickP4Wave"+num2str(CurrentTask)+"_"+num2str(WhichAnimal))+"_"+num
2str(j))=2
//
RecentDisplayWindowsList=RecentDisplayWindowsList+"SamplSepEx"+Num2str(Current
Task)+"_"+num2str(WhichAnimal)+"_"+num2str(j)+";"
```

```
//                    endif
                         WhichIsTheTask[WaveEntryIndex]="Task "+Num2str(CurrentTask)+";
No "+Num2str(j)
                    j+=1
                    WaveEntryIndex+=1
                 while(j<round(TrialsPerTask[CurrentTask-1]/TrialsAveraged+0.49))
                 CurrentTask+=1
              while(CurrentTask<EndTask+0.1)
          endif
       // Now duplicate the generic results from Animal <WHICHANIMAL> to a more general
wave:
//       duplicate /o  tmp_DispFitMaxWave,
$("tmp_DispFitMaxWave"+num2str(WhichAnimal))
//       duplicate /o  tmp_DispFitMaxWaveSD,
$("tmp_DispFitMaxWaveSD"+num2str(WhichAnimal))
//       duplicate /o  tmp_DispFitHalfWave,
$("tmp_DispFitHalfWave"+num2str(WhichAnimal))
//       duplicate /o  tmp_DispFitHalfWaveSD,
$("tmp_DispFitHalfWaveSD"+num2str(WhichAnimal))
//       duplicate /o  tmp_DispFitSlopeWave,
$("tmp_DispFitSlopeWave"+num2str(WhichAnimal))
//       duplicate /o  tmp_DispFitSlopeWaveSD,
$("tmp_DispFitSlopeWaveSD"+num2str(WhichAnimal))
//       duplicate /o  tmp_DispAvgWave,$("tmp_AvgDiffWave"+num2str(WhichAnimal))
       duplicate /o
tmp_DispFirstDetectWave,$("tmp_DispLFirstDetectWave"+num2str(WhichAnimal))

//       if(DispFitMax)
//          SetScale/P x 1,1,"", $("tmp_DispFitMaxWave"+num2str(WhichAnimal))
//          appendtograph /W=FittedMaximaTmp
$("tmp_DispFitMaxWave"+num2str(WhichAnimal))
//          ModifyGraph  /W=FittedMaximaTmp
rgb($("tmp_DispFitMaxWave"+num2str(WhichAnimal)))=(GROUPCOLORRED[GroupOfAn
imal[WhichAnimal]],GROUPCOLORGREEN[GroupOfAnimal[WhichAnimal]],GROUPCOL
ORBLUE[GroupOfAnimal[WhichAnimal]])
//          ErrorBars /W=FittedMaximaTmp
$("tmp_DispFitMaxWave"+num2str(WhichAnimal))
Y,wave=($("tmp_DispFitMaxWaveSD"+num2str(WhichAnimal)),$("tmp_DispFitMaxWaveS
D"+num2str(WhichAnimal)))
//    //      SetAxis /W=FittedMaximaTmp left 0.5,1
//          Label  /W=FittedMaximaTmp left "Fit Max";Label /W=FittedMaximaTmp bottom
"Task number"
//          SetAxis /W=FittedMaximaTmp Bottom 1, numberOfDataPoints
//          ModifyGraph /W=FittedMaximaTmp nticks(bottom)=min(20,numberOfDataPoints)
//       endif
//       if(DispFitSlope)
//          SetScale/P x 1,1,"", $("tmp_DispFitSlopeWave"+num2str(WhichAnimal))
//          appendtograph /W=FittedSlopeTmp
$("tmp_DispFitSlopeWave"+num2str(WhichAnimal))
```

```
//          ModifyGraph  /W=FittedSlopeTmp
rgb($("tmp_DispFitSlopeWave"+num2str(WhichAnimal)))=(GROUPCOLORRED[GroupOf
Animal[WhichAnimal]],GROUPCOLORGREEN[GroupOfAnimal[WhichAnimal]],GROUPCO
LORBLUE[GroupOfAnimal[WhichAnimal]])
//          ErrorBars /W=FittedSlopeTmp
$("tmp_DispFitSlopeWave"+num2str(WhichAnimal))
Y,wave=($("tmp_DispFitSlopeWaveSD"+num2str(WhichAnimal)),$("tmp_DispFitSlopeWav
eSD"+num2str(WhichAnimal)))
//    //      SetAxis /W=FittedSlopeTmp left 0,1
//          Label /W=FittedSlopeTmp  left "Fit Slope";Label /W=FittedSlopeTmp bottom
"Task number"
//          SetAxis /W=FittedSlopeTmp Bottom 1, numberOfDataPoints
//          ModifyGraph /W=FittedSlopeTmp nticks(bottom)=min(20,numberOfDataPoints)
//       endif
//       if(DispFitHalf)
//          SetScale/P x 1,1,"",  $("tmp_DispFitHalfWave"+num2str(WhichAnimal))
//          appendtograph /W=FittedHalfTmp
$("tmp_DispFitHalfWave"+num2str(WhichAnimal))
//          ModifyGraph  /W=FittedHalfTmp
rgb($("tmp_DispFitHalfWave"+num2str(WhichAnimal)))=(GROUPCOLORRED[GroupOfAn
imal[WhichAnimal]],GROUPCOLORGREEN[GroupOfAnimal[WhichAnimal]],GROUPCOL
ORBLUE[GroupOfAnimal[WhichAnimal]])
//          ErrorBars /W=FittedHalfTmp  $("tmp_DispFitHalfWave"+num2str(WhichAnimal))
Y,wave=($("tmp_DispFitHalfWaveSD"+num2str(WhichAnimal)),
$("tmp_DispFitHalfWaveSD"+num2str(WhichAnimal)))
//    //      SetAxis  /W=FittedHalfTmp left 0,1
//          Label /W=FittedHalfTmp left "Fit Half";Label /W=FittedHalfTmp bottom "Task
number"
//          SetAxis  /W=FittedHalfTmp Bottom 1,numberOfDataPoints
//          ModifyGraph  /W=FittedHalfTmp nticks(bottom)=min(20,numberOfDataPoints)
//       endif
//       if(DispAvg)
//          SetScale/P x 1,1,"",  $("tmp_AvgDiffWave"+num2str(WhichAnimal))
//          appendtograph /W=AvgDiffTmp  $("tmp_AvgDiffWave"+num2str(WhichAnimal))
//          ModifyGraph  /W=AvgDiffTmp
rgb($("tmp_AvgDiffWave"+num2str(WhichAnimal)))=(GROUPCOLORRED[GroupOfAnima
l[WhichAnimal]],GROUPCOLORGREEN[GroupOfAnimal[WhichAnimal]],GROUPCOLORB
LUE[GroupOfAnimal[WhichAnimal]])
//    //      SetAxis  /W=AvgDiffTmp left 0,1
//          Label /W=AvgDiffTmp   left "Avg";Label /W=AvgDiffTmp  bottom "Task number"
//          SetAxis  /W=AvgDiffTmp Bottom 1, numberOfDataPoints
//          ModifyGraph  /W=AvgDiffTmp nticks(bottom)=min(20,numberOfDataPoints)
//       endif
      if(DispLFirstDetect)
         SetScale/P x 1,1,"",  $("tmp_DispLFirstDetectWave"+num2str(WhichAnimal))
         appendtograph /W=LFirstDetectTmp
$("tmp_DispLFirstDetectWave"+num2str(WhichAnimal))
         ModifyGraph  /W=LFirstDetectTmp
rgb($("tmp_DispLFirstDetectWave"+num2str(WhichAnimal)))=(GROUPCOLORRED[Grou
```

```
pOfAnimal[WhichAnimal]],GROUPCOLORGREEN[GroupOfAnimal[WhichAnimal]],GROU
PCOLORBLUE[GroupOfAnimal[WhichAnimal]])
          Label /W=LFirstDetectTmp   left "FirstDetect(ms)";Label /W=LFirstDetectTmp
bottom "Task number"
          SetAxis  /W=LFirstDetectTmp Bottom 1, numberOfDataPoints
          ModifyGraph  /W=LFirstDetectTmp nticks(bottom)=min(20,numberOfDataPoints)
       endif

//
*******************************************************************************************************
**********************
//
*******************************************************************************************************
**********************
// Lick analysis done
//
*******************************************************************************************************
**********************
//
*******************************************************************************************************
**********************



    endif // AnimalGroup>0
    WhichAnimal+=1
   While(WhichAnimal<WhichANimalStop+0.2)
End

Function ButtonProc_IndKeepAndLayout(ctrlName) : ButtonControl
   String ctrlName
   string /g recentdisplaywindowslist,keepName
   string tmpGraphNameStr
   variable i=0,numberOfGraphs
   do
      tmpGraphNameStr=stringFromList(i,recentdisplaywindowslist)
      i+=1
   while(stringMatch(tmpGraphNameStr,"")<1)
   NumberOfGraphs=i-1
   i=0
   NewLayout/C=1 as keepName+" Layout"
   DoWindow/C $(keepName+"_Layout")
   do
      tmpGraphNameStr=stringFromList(i,recentdisplaywindowslist)
      DuplicateGraph(tmpGraphNameStr,keepName+"_")
      ModifyGraph /W=$(keepName+"_"+tmpGraphNameStr) width=150,height=30
      DoWindow /B $(keepName+"_"+tmpGraphNameStr)
      appendLayoutObject /W=$(keepName+"_Layout") graph
$(keepName+"_"+tmpGraphNameStr)
      i+=1
```

```
   while(i<NumberOfGraphs)

end

Function ButtonProc_AddLayoutToScrap(ctrlName) : ButtonControl
   String ctrlName
   string /g keepName
   if (WinType("Scrap") != 0)
                // use the previous report notebook, but clear out all the text
   else
                // make a new notebook window
        NewNotebook/F=1/N=Scrap
   endif
   Notebook $("Scrap") selection={endOfFile, endOfFile}
   AppendToScrap("\r\r\r")
   Notebook Scrap picture={$(keepName+"_Layout"), 1, 1}
   AppendToScrap("\r\r\r")
end


Function ButtonProc_CopyScrapToNB(ctrlName) : ButtonControl
   String ctrlName
   string /g keepName, AnalysisNotebookName
   if (WinType("Scrap") != 0)
                // use the previous report notebook, but clear out all the text
   else
                // make a new notebook window
        NewNotebook/F=1/N=Scrap
   endif
   DoWindow/F $("Scrap")
   Notebook $("Scrap") selection={startOfFile, endOfFile}
   DoIgorMenu "Edit", "Copy"
   if (WinType(AnalysisNotebookName) != 0)
                // use the previous report notebook, but clear out all the text
   else
                // make a new notebook window
        NewNotebook/F=1/N=AnalysisNotebookName
   endif
   Notebook $(AnalysisNotebookName) selection={endOfFile, endOfFile}
   DoWindow/F $AnalysisNotebookName
   DoIgorMenu "Edit", "Paste"
   Notebook Scrap picture={$(keepName+"_Layout"), 1, 1}
   Notebook $("Scrap") selection={startOfFile, endOfFile}
   Notebook $("Scrap") text=""
end


Function AppendToScrap(text)
string text
   if (WinType("Scrap") != 0)
```

```
			// use the previous report notebook, but clear out all the text
	else
			// make a new notebook window
	  NewNotebook/F=1/N=Scrap
	endif
	Notebook $("Scrap") defaultTab=36, statusWidth=238, pageMargins={72,72,72,72}
	Notebook $("Scrap") showRuler=0, rulerUnits=1, updating={1, 60}
	Notebook $("Scrap") newRuler=Normal, justification=0, margins={0,0,468},
spacing={0,0,0}, tabs={}, rulerDefaults={"Geneva",10,0,(0,0,0)}
	Notebook $("Scrap") newRuler=Title, justification=1, margins={0,0,468},
spacing={0,0,0}, tabs={}, rulerDefaults={"Geneva",14,1,(0,0,0)}
	Notebook $("Scrap") newRuler=ANOVAReportLine, justification=0, margins={0,0,468},
spacing={0,0,0}, tabs={135+1*8192,199+1*8192,278+1*8192,351+1*8192,414+1*8192},
rulerDefaults={"Geneva",10,0,(0,0,0)}
	Notebook $("Scrap") newRuler=ANOVADifTitles, justification=0, margins={0,0,468},
spacing={0,0,0}, tabs={99,217,306,360,405}, rulerDefaults={"Geneva",10,0,(0,0,0)}
	Notebook $("Scrap") newRuler=ANOVAmeanDifLine, justification=0, margins={0,0,486},
spacing={0,0,0}, tabs={117+3*8192,225+3*8192,297,360+3*8192,414+3*8192},
rulerDefaults={"Geneva",10,0,(0,0,0)}
	Notebook $("Scrap") selection={endOfFile, endOfFile}
	Notebook $("Scrap") text=text
end

Function HeadlineScrap(text)
string text
  AppendToScrap("")
  Notebook $("Scrap") text="\r"
  Notebook $("Scrap") ruler=Title, fSize=14, fStyle=1, textRGB=(50000,0,0 ), text=text+"\r"
  Notebook $("Scrap") ruler=Normal, fSize=10, textRGB=(0,0,0 ), text="\r"

end

Function FirstHeadlineScrap(text)
string text
  AppendToScrap("")
  Notebook $("Scrap") ruler=Normal, fSize=10, fStyle=0, textRGB=(0,0,0 ), text="\r"
  Notebook $("Scrap") text="\r"
  variable i
  i=0
  do
	AppendToScrap("============")
	i+=1
  while(i<13)
  Notebook $("Scrap") text="\r\r\r"
  Notebook $("Scrap") ruler=Title, fSize=18, fStyle=5, textRGB=(50000,0,0 ), text=text+"\r"
  Notebook $("Scrap") ruler=Normal, fSize=10, fStyle=0, textRGB=(0,0,0 ), text="\r"

End

#pragma rtGlobals=1	// Use modern global access method.
```

```
Window IndividualAnimalAnalysis() : Panel
   DoWindow /K IndividualAnimalAnalysis
   variable /g WhichAnimalStart, WhichAnimalStop,FinalValveTime,TemporalResolution
   string /g keepName=""
   PauseUpdate; Silent 1       // building window...
   NewPanel  /K=1 /W=(330,217.25,657.75,555.75) as "Individual Animal Analysis"
   SetVariable setvar0,pos={9,9},size={151,16},title="Start Task"
   SetVariable setvar0,limits={1,Inf,1},value= StartTask
   SetVariable setvar0_1,pos={164,9},size={151,16},title="End Task"
   SetVariable setvar0_1,limits={1,Inf,1},value= EndTask
   SetVariable setvar0_2,pos={9,31},size={151,16},title="Animal Number Start"
   SetVariable setvar0_2,limits={1,Inf,1},value= WhichAnimalStart
   SetVariable setvar0_3,pos={164,31},size={151,16},title="Animal Number Stop"
   SetVariable setvar0_3,limits={1,Inf,1},value= WhichAnimalStop
// SetVariable setvar0_3,pos={9,56},size={151,16},title="Number of S+ Valves"
// SetVariable setvar0_3,limits={1,Inf,1},value= NumSPlusValves
// SetVariable setvar0_4,pos={164,56},size={151,16},title="Number of S- Valves"
// SetVariable setvar0_4,limits={1,Inf,1},value= NumSMinusValves
   SetVariable setvar0_5,pos={9,116},size={115,16},title="Percent Correct "
   SetVariable setvar0_5,limits={0,1,1},value= DispPercCorr
   SetVariable setvar0_6,pos={9,132},size={115,16},title="Max              "
   SetVariable setvar0_6,limits={0,1,1},value= DispMax
   SetVariable setvar0_7,pos={9,148},size={115,16},title="Mean             "
   SetVariable setvar0_7,limits={0,1,1},value= DispAvg
   SetVariable setvar0_8,pos={9,164},size={115,16},title="Fit Max          "
   SetVariable setvar0_8,limits={0,1,1},value= DispFitMax
   SetVariable setvar0_9,pos={9,180},size={115,16},title="Fit Slope        "
   SetVariable setvar0_9,limits={0,1,1},value= DispFitSlope
   SetVariable setvar0_10,pos={9,196},size={115,16},title="Fit Half          "
   SetVariable setvar0_10,limits={0,1,1},value= DispFitHalf
   SetVariable setvar0_12,pos={9,212},size={115,16},title="Difference Smpl"
   SetVariable setvar0_12,limits={0,1,1},value= DispSingle
   SetVariable setvar0_13,pos={9,228},size={115,16},title="PlusMinus        "
   SetVariable setvar0_13,limits={0,1,1},value= DispPM
   SetVariable setvar0_17,pos={9,244},size={115,16},title="First Detect      "
   SetVariable setvar0_17,limits={0,5,1},value= DispFirstDetect
   SetVariable setvar0_19,pos={9,260},size={115,16},title="Select ++-       "
   SetVariable setvar0_19,limits={0,5,1},value= DispSelectPPM

   SetVariable setvar0_22,pos={9,276},size={115,16},title="Difference Lick"
   SetVariable setvar0_22,limits={0,1,1},value= DispLSingle
   SetVariable setvar0_23,pos={9,292},size={115,16},title="Lc PlusMinus      "
   SetVariable setvar0_23,limits={0,1,1},value= DispLPM
   SetVariable setvar0_27,pos={9,308},size={115,16},title="Lc First Detect    "
   SetVariable setvar0_27,limits={0,15,1},value= DispLFirstDetect

   SetVariable setvar0_14,pos={164,148},size={151,16}, value= keepName, title="Name
For saving"
```

```
    SetVariable setvar0_15,pos={164,164},size={151,16}, value=FinalValveTime,
title="Final Valve Time (ms)"
    SetVariable setvar0_16,pos={164,180},size={151,16}, value=TemporalResolution,
title="Tmp. Resol. (ms/unit)"
    Button
button0,pos={170,84},size={138,23},proc=ButtonProc_SpecifySPMValves,title="Specify S
+/- valves"
    Button
button0_4,pos={170,55},size={138,23},proc=ButtonProc_SpecifyTrialsPerTask,title="Speci
fy Trials/Task"
    Button
button0_1,pos={170,117},size={138,23},proc=ButtonProc_IndKeepAndLayout,title="Keep
Traces And Layout"
    Button
button0_2,pos={170,239},size={138,23},proc=ButtonProc_IndDisplayAndLayout,title="Disp
lay"
    Button button0_7,pos={170,269},size={138,23},proc=ButtonProc_Enlarge,title="Enlarge
Graph"
    Button button0_8,pos={170,299},size={138,23},proc=ButtonProc_freeGraph,title="Free
Graph"
    Button
button0_5,pos={155,210},size={80,15},proc=ButtonProc_AddLayoutToScrap,title="Layout-
>Scrap"
    Button
button0_6,pos={245,210},size={80,15},proc=ButtonProc_CopyScrapToNB,title="Scrap-
>NB"
    SetVariable setvar0_11,pos={9,53},size={151,16},title="Sampling time (units)"
    SetVariable setvar0_11,limits={1,Inf,1},value= LengthOfTimeSampling
EndMacro

//
****************************************************************************************************************************
***********************************************************
// *************************** Specification of Valves
//
****************************************************************************************************************************
***********************************************************

// ************************** Windows for Sepcification of Valves

Window MainSpecifyValves() : Panel
    DoWindow /K MainSpecifyValves
    PauseUpdate; Silent 1       // building window...
    NewPanel /K=1 /W=(330,217.25,657,332) as "Main Window Specify Valves"
    SetDrawLayer UserBack
    SetDrawEnv fsize= 16,fstyle= 1
    DrawText 61,35,"This is valves for Task "+num2str(CurrentTask)
    SetVariable setvar0_3,pos={9,56},size={151,16},title="Number of S+ Valves"
    SetVariable setvar0_3,limits={1,Inf,1},value= NumSPlusValvesTmp
    SetVariable setvar0_4,pos={164,56},size={151,16},title="Number of S- Valves"
```

```
   SetVariable setvar0_4,limits={1,Inf,1},value= NumSMinusValvesTmp
   Button
button0,pos={9,82},size={150,23},proc=ButtonProc_SpecifyValvesSingle,title="Specify
valves for Task "+num2str(CurrentTask)
   Button
button0_3,pos={170,82},size={138,23},proc=ButtonProc_LookAtOdorvalve,title="Look at
Odor valve"
EndMacro

Window SpecifyValves() : Panel
   DoWindow /K SpecifyValves
   variable i
   PauseUpdate; Silent 1       // building window...
   NewPanel  /K=1 /W=(400,50,600,110+max(NumSPLusValvesTmp,
NumSMinusValvesTmp)*20) as "Specify Valves"
   i=0
   do
     $("tmpP_"+num2str(i)) = SPlusValves[CurrentTask-1][i]
     SetVariable $("setvar"+num2str(i)),pos={7,9+20*i},size={75,16},title="S + "+num2str(i)
     SetVariable $("setvar"+num2str(i)),limits={1,Inf,1},value= $("tmpP_"+num2str(i))
     i+=1
   while(i<NumSPlusValvesTmp)

   i=0
   do
     $("tmpM_"+num2str(i))=SMinusValves[CurrentTask-1][i]
     SetVariable
$("setvar"+num2str(i+NumSPlusValvesTmp)),pos={100,9+20*i},size={75,16},title="S -
"+num2str(i)
     SetVariable $("setvar"+num2str(i+NumSPlusValvesTmp)),limits={1,Inf,1},value=
$("tmpM_"+num2str(i))
     i+=1
   while(i<NumSMinusValvesTmp)
   Button InitAll,pos={50,20+20*max(NumSMinusValvesTmp,
NumSPlusValvesTmp)},size={86,32},proc=ButtonProc_SubmitValves,title="Submit"

EndMacro

// ************************** Buttons for Sepcification of Valves


Function ButtonProc_SpecifyValvesSingle(ctrlName) : ButtonControl
   String ctrlName
   variable /g NumSPlusValvesTmp, NuMSMinusValvesTmp, NumberOfTasks
   variable /g CurrentTask
   make /N=(NumberOfTasks) /O NumSPlusValves, NumSMinusValves
   NumSPlusValves[CurrentTask-1]=NumSPlusValvesTmp
   NumSMinusValves[CurrentTask-1]=NumSMinusValvesTmp
   Execute("SpecifyValves()")
End
```

```
Function ButtonProc_SpecifySPMValves(ctrlName) : ButtonControl
   String ctrlName
   variable /g NumSPlusValvesTmp, NuMSMinusValvesTmp, MAXVALVNUMBER,
MAXVALVNUMBER, NumberOfTasks
   variable /g CurrentTask,StartTask
   make /N=(NumberOfTasks) /O NumSPlusValves, NumSMinusValves
   CurrentTask=StartTask
   NumSPlusValvesTmp   = NumSPlusValves[CurrentTask-1]
   NumSMinusValvesTmp = NumSMinusValves[CurrentTask-1]
//  make /N=(NumberOfTasks,MAXVALVNUMBER) /O SPlusValves
//  make /N=(NumberOfTasks,MAXVALVNUMBER) /O SMinusValves
   variable i=0
   do
      print i
      variable /g $("tmpP_"+num2str(i))
         variable /g $("tmpM_"+num2str(i));
      i+=1
   while(i<MAXVALVNUMBER)
   execute("MainSpecifyValves()")
END


Function ButtonProc_SubmitValves(ctrlName) : ButtonControl
   String ctrlName
   DoWindow /K SpecifyValves
   DoWindow /K OdorValveCheckTable
   execute("Macro_SubbmitValves()")
End

proc Macro_SubbmitValves()
   DoWindow /K MainSpecifyValves
   DoWindow /K SpecifyValves
   variable i
   i=0
   do
    SMinusValves[CurrentTask-1][i]=$("tmpM_"+num2str(i))
    i+=1
   while(i<NumSMinusValves[CurrentTask-1])
   i=0
   do
    SPlusValves[CurrentTask-1][i]=$("tmpP_"+num2str(i))
    i+=1
   while(i<NumSPlusValves[CurrentTask-1])
   CurrentTask+=1
   if(CurrentTask<EndTask+0.1)
      MainSpecifyValves()
   endif
```

```
endmacro

Function ButtonProc_LookAtOdorvalve(ctrlName) : ButtonControl
   String ctrlName
   variable /g CurrentTask, WhichAnimal
   make /O OdorValve, SamplePattern, IsRewarded
   string /g IndividualTaskName, SPECIFICWAVEENDING

   DoWindow /K OdorValveCheckTable
   duplicate /o /T $IndividualTaskName, tmpWave1
   string PathSpecifier

   PathSpecifier=tmpWave1[WhichAnimal-1][CurrentTask-1]
   print pathSpecifier
   killwaves IsRewarded
   LoadWave/H/O/P=DataPath/K=0 ":"+PathSpecifier+"
Folder:IsRewarded."+SPECIFICWAVEENDING
   killwaves OdorValve
   LoadWave/H/O/P=DataPath/K=0 ":"+PathSpecifier+"
Folder:OdorValve."+SPECIFICWAVEENDING
   DoAlert 0,"Close Table after checking"
   execute("OdorValveCheckTable()")

End

Window OdorValveCheckTable() : Table
   PauseUpdate; Silent 1       // building window...
   Edit/K=1/W=(5.25,41.75,257.25,468.5) OdorValve,IsRewarded as "Check Which Valve
was rewarded"
EndMacro


//
*****************************************************************************************************************
*************************************************
// *************************** End Of Specification of Valves
//
*****************************************************************************************************************
*************************************************
//
*****************************************************************************************************************
*************************************************
// ***************************  Specification of Trial Numbers per Task
//
*****************************************************************************************************************
*************************************************

Function ButtonProc_SpecifyTrialsPerTask(ctrlName) : ButtonControl
   String ctrlName
   variable /g NumberOfTasks, TrialsAveraged
```

```
   make /N=(NumberOfTasks) /O TrialsPerTask, FinalValveTimeArray
   variable i=0
   do
     variable /g $("tmp_"+num2str(i))
     variable /g $("tmp2_"+num2str(i))
     i+=1
   while(i<NumberOfTasks)

   execute("MainSpecifyTrialsPerTask()")
END

Window MainSpecifyTrialsPerTask() : Panel
   DoWindow /K MainSpecifyTrialsPerTask
   variable i
   do
     variable $("tmp_"+num2str(i)) = TrialsPerTask[i]
     variable $("tmp2_"+num2str(i)) = FinalValveTimeArray[i]
     i+=1
   while(i<NumberOfTasks)
   PauseUpdate; Silent 1       // building window...
   NewPanel  /K=1 /W=(400,50,750,150+NumberOfTasks*20) as "Specify No Trials Per
Task & FV time  "
   i=0
   do
     SetVariable $("setvar"+num2str(i)),pos={7,9+20*i},size={175,16},title="# Trials for Task
"+num2str(i+1)
     SetVariable $("setvar"+num2str(i)),limits={1,Inf,1},value= $("tmp_"+num2str(i))
     SetVariable
$("setvar"+num2str(i+NumberOfTasks+2)),pos={200,9+20*i},size={100,16},title="FV time"
     SetVariable $("setvar"+num2str(i+NumberOfTasks+2)),limits={1,Inf,1},value=
$("tmp2_"+num2str(i))
     i+=1
   while(i<NumberOfTasks)
   SetVariable $("setvar"+num2str(i)),pos={80,9+20*i+10},size={175,16},title="# Trials
Averaged"
   SetVariable $("setvar"+num2str(i)),limits={1,Inf,1},value= TrialsAveraged

   Button
InitAll,pos={100,60+20*NumberOfTasks},size={86,32},proc=ButtonProc_SubmitTasks,title
="Submit"
END

Function ButtonProc_SubmitTasks(ctrlName) : ButtonControl
   String ctrlName
   DoWindow /K MainSpecifyTrialsPerTask
   execute("Macro_SubbmitTrialsPerTask()")
End

proc Macro_SubbmitTrialsPerTask()
   variable i
```

```
   i=0
   do
     TrialsPerTask[i]=$("tmp_"+num2str(i))
     FinalValveTimeArray[i]=$("tmp2_"+num2str(i))
     i+=1
   while(i<NumberOfTasks)
endmacro


//
****************************************************************************************************
***********************************************
// *************************** End Of Specification of Trial Numbers
//
****************************************************************************************************
***********************************************



Function ButtonProc_Enlarge(ctrlName) : ButtonControl
	String ctrlName
	variable i=0
	ModifyGraph width=648,height=432
End

Function ButtonProc_freeGraph(ctrlName) : ButtonControl
	String ctrlName
	ModifyGraph width=0,height=0
End

#pragma rtGlobals=1		// Use modern global access method.
Function LastLickTime()
WAVE LickPattern=LickPattern
WAVE isrewarded=Isrewarded
make /N=1000 /O StopLickTimes
make /N=300 /O SLTP=nan, SLTM=nan
variable countP=0, countM=0
variable i=1, j
do
	j=125
	do
		StopLickTimes[i]=125*20-500
		if(LickPattern[i][j]>0.5)
			StopLickTimes[i]=j*20-500
			j=-10
		endif
		j-=1
	while(j>-1)
	if(isrewarded[i])
		SLTP[countP]=StopLickTimes[i]
```

```
			countP+=1
		else
			SLTM[countM]=StopLickTimes[i]
			countM+=1
		endif
		i+=1
	while(i<301)
	make /O histP, histM
	Histogram/B={-500,,20,126} SLTM,histM
	Histogram/B={-500,,20,126} SLTP,histP
	display histM, histP
	ModifyGraph mode=5,rgb(histP)=(0,65280,0)
	ModifyGraph log(left)=1
	SetAxis left 0.5,200
end


Function LastSampleTime()
WAVE SamplePattern=SamplePattern
WAVE isrewarded=Isrewarded
make /N=1000 /O StopSampleTimes
make /N=300 /O SSTP=nan, SSTM=nan
variable countP=0, countM=0
variable i=1, j
do
	j=125
	StopSampleTimes[i]=125*20-500
	do
		if(SamplePattern[i][j]>0.5)
			StopSampleTimes[i]=j*20-500
			j=-10
		endif
		j-=1
	while(j>-1)
	if(isrewarded[i])
		SSTP[countP]=StopSampleTimes[i]
		countP+=1
	else
		SSTM[countM]=StopSampleTimes[i]
		countM+=1
	endif
	i+=1
while(i<301)
make /O histSP, histSM
Histogram/B={-500,20,126} SSTM,histSM
Histogram/B={-500,20,126} SSTP,histSP
display histSM, histSP
ModifyGraph mode=5,rgb(histSP)=(0,65280,0)
ModifyGraph log(left)=1
SetAxis left 0.5,200
```

```
end



MACRO LookAtCrossCorrel()
variable i=211
duplicate /O tmpCorrel,CorrelFinal
CorrelFinal=0
do
make /N=100/O tmpCorrel
tmpCorrel=tmpLickPattern[i][p+25]
wavestats/Q tmpCorrel
tmpCorrel-=v_avg
tmpCorrel/=v_sdev
Correlate tmpCorrel, tmpCorrel
CorrelFinal+=tmpCorrel
i+=1
while(i<402)
END

#pragma rtGlobals=1      // Use modern global access method.
#pragma rtGlobals=1     // Use modern global access method.

Window MultipleAnimalAnalysis() : Panel
   DoWindow /K MultipleAnimalAnalysis
   string /g keepName=""
   PauseUpdate; Silent 1       // building window...
   NewPanel  /K=1 /W=(330,217.25,657.75,530) as "Multiple Animal Analysis"
   SetVariable setvar0,pos={9,9},size={151,16},title="Start Task"
   SetVariable setvar0,limits={1,Inf,1},value= StartTask
   SetVariable setvar0_1,pos={164,9},size={151,16},title="End Task"
   SetVariable setvar0_1,limits={1,Inf,1},value= EndTask
   SetVariable setvar0_5_2,pos={9,84},size={115,16},title="ITI                 "
   SetVariable setvar0_5_2,limits={0,1,1},value= DispITI
   SetVariable setvar0_5_1,pos={9,100},size={115,16},title="Percent Licked "
   SetVariable setvar0_5_1,limits={0,1,1},value= DispPercLick
   SetVariable setvar0_5,pos={9,116},size={115,16},title="Percent Correct "
   SetVariable setvar0_5,limits={0,1,1},value= DispPercCorr
   SetVariable setvar0_6,pos={9,132},size={115,16},title="Max              "
   SetVariable setvar0_6,limits={0,1,1},value= DispMax
   SetVariable setvar0_7,pos={9,148},size={115,16},title="Mean             "
   SetVariable setvar0_7,limits={0,1,1},value= DispAvg
   SetVariable setvar0_8,pos={9,164},size={115,16},title="Fit Max          "
   SetVariable setvar0_8,limits={0,1,1},value= DispFitMax
   SetVariable setvar0_9,pos={9,180},size={115,16},title="Fit Slope         "
   SetVariable setvar0_9,limits={0,1,1},value= DispFitSlope
   SetVariable setvar0_10,pos={9,196},size={115,16},title="Fit Half            "
   SetVariable setvar0_10,limits={0,1,1},value= DispFitHalf
   SetVariable setvar0_14,pos={164,148},size={151,16}, value= keepName, title="Name
For saving"
```

```
    Button
button0_3,pos={170,28},size={138,23},proc=ButtonProc_SpecifyGroups,title="Specify
Animal Groups"
    Button
button0,pos={170,84},size={138,23},proc=ButtonProc_SpecifySPMValves,title="Specify S
+/- valves"
    Button
button0_1,pos={170,117},size={138,23},proc=ButtonProc_IndKeepAndLayout,title="Keep
Traces And Layout"
    Button
button0_2,pos={170,239},size={138,23},proc=ButtonProc_MultDisplayAndLayout,title="Dis
play"
    Button
button0_7,pos={155,210},size={80,15},proc=ButtonProc_AddLayoutToScrap,title="Layout-
>Scrap"
    Button
button0_6,pos={245,210},size={80,15},proc=ButtonProc_CopyScrapToNB,title="Scrap-
>NB"
    Button
button0_5,pos={170,180},size={138,23},proc=ButtonProc_MultDoStats,title="Statistics ..."
    Button
button0_4,pos={170,55},size={138,23},proc=ButtonProc_SpecifyTrialsPerTask,title="Speci
fy Trials&FV/Task"
    SetVariable setvar0_11,pos={9,53},size={151,16},title="Sampling time (units)"
    SetVariable setvar0_11,limits={1,Inf,1},value= LengthOfTimeSampling
    SetVariable setvar0_12,pos={9,212},size={115,16},title="Difference Smpl"
    SetVariable setvar0_12,limits={0,1,1},value= DispSingle
    SetVariable setvar0_13,pos={9,228},size={115,16},title="PlusMinus         "
    SetVariable setvar0_13,limits={0,1,1},value= DispPM
    SetVariable setvar0_17,pos={9,244},size={115,16},title="First Detect(Stats)"
    SetVariable setvar0_17,limits={0,5,1},value= DispFirstDetect
    SetVariable setvar0_177,pos={9,270},size={115,16},title="Only Correct ?"
    SetVariable setvar0_177,limits={0,5,1},value= CountOnlyCorrect

EndMacro

//
**************************************************************************************************************************
**************************************************
// ************************** Specification of Groups
//
**************************************************************************************************************************
**************************************************



Window GroupAnimals() : Panel
    DoWindow /K GroupAnimals
    variable i
    PauseUpdate; Silent 1       // building window...
```

```
   NewPanel  /K=1 /W=(400,50,540,110+NumberOfAnimals*20) as "Grouping Animals"
   i=1
   do
     $("tmpA_"+num2str(i)) = GroupOfAnimal[i]
     SetVariable $("setvar"+num2str(i)),pos={7,20*i-11},size={100,16},title="Animal #
"+num2str(i)
     SetVariable $("setvar"+num2str(i)),limits={0,Inf,1},value= $("tmpA_"+num2str(i))
     i+=1
   while(i<NumberOfAnimals+1)

   Button
InitAll,pos={35,20+20*NumberOfAnimals},size={86,32},proc=ButtonProc_SubmitAnimalGr
oups,title="Submit"

EndMacro

Function ButtonProc_SpecifyGroups(ctrlName) : ButtonControl
   String ctrlName
   execute("GroupAnimals()")
END


Function ButtonProc_SubmitAnimalGroups(ctrlName) : ButtonControl
   String ctrlName
   DoWindow /K GroupAnimals
   execute("Macro_SubmitAnimalGroups()")
End

proc Macro_SubmitAnimalGroups()
   variable i
   i=1
   do
     GroupOfAnimal[i]=$("tmpA_"+num2str(i))
     i+=1
   while(i<NumberOfAnimals+1)
   wavestats /Q GroupOfAnimal
   NumberOfGroups=V_max
endmacro

//
**********************************************************************************************************
**************************************************
// ************************** End Of Specification of Groups
//
**********************************************************************************************************
**************************************************

Function ButtonProc_MultDisplayAndLayout(ctrlName) : ButtonControl
   String ctrlName
   string /g IndividualTaskName
```

```
   string /g RecentDisplayWindowsList=""
   variable /g StartTask, EndTask, CurrentTask
   variable /g LengthOfTimeSampling, NumberOfTasks, NumberOfAnimals,
NumberOfGroups, WhichAnimal
   variable /g FinalValveTime,TemporalResolution, FirstDetect
   variable /g startTrials=0, endTrials=199
   variable /g DispPercCorr, DispMax, DispAvg, DispFitMax, DispFitSlope, DispFitHalf,
DispSingle, DispPM, TrialsAveraged, DispFirstDetect, DispPercLick, DispITI
   make /O W_coef, W_Sigma
   make /O /N=(LengthOfTimeSampling) tmp_SamplingPWave,tmp_SamplingMWave
   make /N=(NumberOfAnimals+1) /O GroupOfAnimal
   make /N=(NumberOfTasks) /O TrialsPerTask, FinalValveTimeArray

   variable /g MAXNUMBEROFGROUPS,MAXNUMBEROFTICKS
   make /N=(MAXNUMBEROFGROUPS) /O
GROUPCOLORRED,GROUPCOLORGREEN,GROUPCOLORBLUE

   make /N=(MAXNUMBEROFGROUPS) /o animalsPerGroup=0
   variable i,j,tmpLogic, AnimalCount
   variable WaveEntryIndex, numberOfDataPoints     // running from 0 to
NumberOfDataPoints-1
   // calculate number of data points in output waves (sampling rate (e.g. 100 trials)
summed up over tasks)
   i=StartTask-1
   do
      numberOfDataPoints+=round(TrialsPerTask[i]/TrialsAveraged+0.49)     // if trials are
averaged in 100 a piece but only 250 trials -> nevertheless three entries
      i+=1
   while(i<EndTask-1+0.1)
   print NumberOfDataPoints

   DoWindow /K SamplingDiffExmpl
   DoWindow /K FittedMaximaTmp
   DoWindow /K FittedSlopeTmp
   DoWindow /K FittedHalfTmp
   DoWindow /K AvgDiffTmp
   DoWindow /K FirstDetectTmp

   if(DispFitMax)
      display /W=(3,40,220,120)/K=1 as "Fitted Maxima"
//    SetAxis left 0.5,1
      DoWindow/C FittedMaximaTmp
      ModifyGraph width=150,height=30
      RecentDisplayWindowsList=RecentDisplayWindowsList+"FittedMaximaTmp;"
   endif
   if(DispFitSlope)
      display /W=(3,140,220,220)/K=1 as "Fitted Slope"
      DoWindow/C FittedSlopeTmp
      ModifyGraph width=150,height=30
      RecentDisplayWindowsList=RecentDisplayWindowsList+"FittedSlopeTmp;"
```

```
   endif
   if(DispFitHalf)
      display /W=(3,240,220,320)/K=1 as "Fitted HalfMaximum"
      DoWindow/C FittedHalfTmp
      ModifyGraph width=150,height=30
      RecentDisplayWindowsList=RecentDisplayWindowsList+"FittedHalfTmp;"
   endif
   if(DispAvg)
      display /W=(3,340,220,420)/K=1 as "Average Difference"
      DoWindow/C AvgDiffTmp
      ModifyGraph width=150,height=30
      RecentDisplayWindowsList=RecentDisplayWindowsList+"AvgDiffTmp;"
   endif
   if(DispFirstDetect)
      display /W=(3,240,220,320)/K=1 as "First Detect"
      DoWindow/C FirstDetectTmp
      ModifyGraph width=150,height=30
      RecentDisplayWindowsList=RecentDisplayWindowsList+"FirstDetectTmp;"
   endif
   if(DispSingle)
      display/K=1  as "Individual Sampling Differences"
      DoWindow/C SamplingDiff
      ModifyGraph width=150,height=30
      RecentDisplayWindowsList=RecentDisplayWindowsList+"SamplingDiff;"
   endif
   make /N=(NumberOfDataPoints,NumberOfGroups+1) /O
tmp2D_DispFitMaxWave=0,tmp2D_DispFitMaxWaveSEM=0
   make /N=(NumberOfDataPoints,NumberOfGroups+1) /O
tmp2D_DispAvgWave=0,tmp2D_DispAvgWaveSEM=0
   make /N=(NumberOfDataPoints,NumberOfGroups+1) /O
tmp2D_DispFitSlopeWave=0,tmp2D_DispFitSlopeWaveSEM=0
   make /N=(NumberOfDataPoints,NumberOfGroups+1) /O
tmp2D_DispFitHalfWave=0,tmp2D_DispFitHalfWaveSEM=0
   make /N=(NumberOfDataPoints,NumberOfAnimals+1) /O
tmp2D_DispFirstDetectWave=0,tmp2D_DispFirstDetectWaveSEM=0
   make /N=(numberOfDataPoints) /O /T WhichIsTheTask
   make /N=(NumberOfDataPoints,NumberOfAnimals+1) /O ForANOVA_FirstDetect,
ForANOVA_FitMax, ForANOVA_FitSlope, ForANOVA_FitHalf, ForANOVA_Avg

   AnimalCount = 1
   do
      WhichAnimal=AnimalCount
      CurrentTask=StartTask
      WaveEntryIndex=0
      animalsPerGroup[GroupOfAnimal[AnimalCount]]+=1
         if(GroupOfAnimal[AnimalCount]>0)
              do
                 j=0
                do
                            FinalValveTime=FinalValveTimeArray[CurrentTask-1]
```

```
                        startTrials=j*TrialsAveraged+1
                        endTrials=min(TrialsPerTask[CurrentTask-
1],startTrials+TrialsAveraged)
        print waveentryindex, WhichAnimal, CurrentTask, startTrials, endTrials
                        GetSamplePattern()
                      SetScale/P x -FinalValveTime,TemporalResolution,
tmp_SamplingMWave
                      SetScale/P x -FinalValveTime,TemporalResolution,
tmp_SamplingPWave

                        duplicate /O tmp_SamplingPWave, tmp_SamplingDiffWave
                        tmp_SamplingDiffWave=tmp_SamplingPWave-tmp_SamplingMWave
                        duplicate /O tmp_samplingDiffWave,
$("tmp_SamplingDiff"+num2str(CurrentTask)+"_"+num2str(AnimalCount))
                        if(DispSingle * GroupOfAnimal[AnimalCount])
                           appendtograph /W=SamplingDiff
$("tmp_SamplingDiff"+num2str(CurrentTask)+"_"+num2str(AnimalCount))
                           ModifyGraph  /W=SamplingDiff
rgb($("tmp_SamplingDiff"+num2str(CurrentTask)+"_"+num2str(AnimalCount)))=(GROUPC
OLORRED[GroupOfAnimal[AnimalCount]],GROUPCOLORGREEN[GroupOfAnimal[Anima
lCount]],GROUPCOLORBLUE[GroupOfAnimal[AnimalCount]])
                        endif
                        K0 = 0;
                        wavestats /Q tmp_SamplingDiffWave

W_coef[1]=0;W_coef[2]=TemporalResolution*LengthOfTimeSampling-
FinalValveTime;W_coef[3]=W_coef[2];
                        if((V_sdev>0.0001)*(DispFitSlope+DispFitMax+DispFitHalf))
                                    CurveFit/H="1000" /Q Sigmoid tmp_SamplingDiffWave
                        endif

tmp2D_DispFitMaxWave[WaveEntryIndex][GroupOfAnimal[AnimalCount]]+=W_coef[1]
                        ForANOVA_FitMax[WaveEntryIndex][AnimalCount]=W_coef[1]

tmp2D_DispFitMaxWaveSEM[WaveEntryIndex][GroupOfAnimal[AnimalCount]]+=W_coef[
1]^2

tmp2D_DispFitSlopeWave[WaveEntryIndex][GroupOfAnimal[AnimalCount]]+=W_coef[3]
                        ForANOVA_FitSlope[WaveEntryIndex][AnimalCount]=W_coef[3]

tmp2D_DispFitSlopeWaveSEM[WaveEntryIndex][GroupOfAnimal[AnimalCount]]+=W_coef
[3]^2

tmp2D_DispFitHalfWave[WaveEntryIndex][GroupOfAnimal[AnimalCount]]+=W_coef[2]
                        ForANOVA_FitHalf[WaveEntryIndex][AnimalCount]=W_coef[2]

tmp2D_DispFitHalfWaveSEM[WaveEntryIndex][GroupOfAnimal[AnimalCount]]+=W_coef[2
]^2
                        GetDifferentTValues(DispFirstDetect)
```

```
tmp2D_DispFirstDetectWave[WaveEntryIndex][GroupOfAnimal[AnimalCount]]+=FirstDete
ct
                        ForANOVA_FirstDetect[WaveEntryIndex][AnimalCount]=FirstDetect

tmp2D_DispFirstDetectWaveSEM[WaveEntryIndex][GroupOfAnimal[AnimalCount]]+=First
Detect^2

                        wavestats /Q tmp_SamplingDiffWave

tmp2D_DispAvgWave[WaveEntryIndex][GroupOfAnimal[AnimalCount]]+=V_avg

tmp2D_DispAvgWaveSEM[WaveEntryIndex][GroupOfAnimal[AnimalCount]]+=V_avg^2
                        ForANOVA_Avg[WaveEntryIndex][AnimalCount]=V_avg
                //      tmp_DispAvgWave[CurrentTask-StartTask]=V_avg
                          WhichIsTheTask[WaveEntryIndex]="Task
"+Num2str(CurrentTask)+"; No "+Num2str(j)

                    j+=1
                    WaveEntryIndex+=1
                while(j<round(TrialsPerTask[CurrentTask-1]/TrialsAveraged+0.49))
                CurrentTask+=1
             while(CurrentTask<EndTask+0.1)
          endif // AnimalGroup>0
       AnimalCount+=1
    While(AnimalCount<NumberOfAnimals+0.1)
    if(DispFitMax)
       i=0
       do
          tmp2D_DispFitMaxWave[][i]/=max(1,animalsPerGroup[i])

tmp2D_DispFitMaxWaveSEM[][i]=(animalsPerGRoup[i]<2?0:sqrt((tmp2D_DispFitMaxWav
eSEM[p][i]-animalsPerGroup[i]*tmp2D_DispFitMaxWave[p][i]^2)/(animalsPerGroup[i]-
1))/sqrt(animalsPerGroup[i]))
          make /N=(NumberOfDataPoints) /O tmpWave
          tmpWave=tmp2D_DispFitMaxWave[p][i]
          duplicate /O tmpWave, $("tmp_DispFitMaxWaveG"+num2str(i))
          SetScale/P x 1,1,"", $("tmp_DispFitMaxWaveG"+num2str(i))
          tmpWave=tmp2D_DispFitMaxWaveSEM[p][i]
          duplicate /O tmpWave, $("tmp_DispFitMaxWaveSEMG"+num2str(i))
          SetScale/P x 1,1,"", $("tmp_DispFitMaxWaveSEMG"+num2str(i))
          appendtograph /W=FittedMaximaTmp $("tmp_DispFitMaxWaveG"+num2str(i))
          ModifyGraph  /W=FittedMaximaTmp
rgb($("tmp_DispFitMaxWaveG"+num2str(i)))=(GROUPCOLORRED[i],GROUPCOLORGR
EEN[i],GROUPCOLORBLUE[i])
          ErrorBars /W=FittedMaximaTmp $("tmp_DispFitMaxWaveG"+num2str(i))
Y,wave=($("tmp_DispFitMaxWaveSEMG"+num2str(i)),$("tmp_DispFitMaxWaveSEMG"+n
um2str(i)))
          i+=1
       while(i<NumberOfGroups+1)
```

```
//      SetAxis /W=FittedMaximaTmp left 0.5,1
        Label  /W=FittedMaximaTmp left "Fit Max";Label /W=FittedMaximaTmp bottom "Task
number"
        SetAxis /W=FittedMaximaTmp Bottom 1, NumberOfDataPoints
        ModifyGraph /W=FittedMaximaTmp
nticks(bottom)=min(NumberOfDataPoints,MAXNUMBEROFTICKS)
    endif

    if(DispFitSlope)
        i=0
        do
            tmp2D_DispFitSlopeWave[][i]/=Max(1,animalsPerGroup[i])

tmp2D_DispFitSlopeWaveSEM[][i]=(animalsPerGRoup[i]<2?0:sqrt((tmp2D_DispFitSlopeW
aveSEM[p][i]-animalsPerGroup[i]*tmp2D_DispFitSlopeWave[p][i]^2)/(animalsPerGroup[i]-
1))/sqrt(animalsPerGroup[i]))
            make /N=(NumberOfDataPoints) /O tmpWave
            tmpWave=tmp2D_DispFitSlopeWave[p][i]
            duplicate /O tmpWave, $("tmp_DispFitSlopeWaveG"+num2str(i))
            SetScale/P x 1,1,"", $("tmp_DispFitSlopeWaveG"+num2str(i))
            tmpWave=tmp2D_DispFitSlopeWaveSEM[p][i]
            duplicate /O tmpWave, $("tmp_DispFitSlopeWaveSEMG"+num2str(i))
            SetScale/P x 1,1,"", $("tmp_DispFitSlopeWaveSEMG"+num2str(i))
            appendtograph /W=FittedSlopeTmp $("tmp_DispFitSlopeWaveG"+num2str(i))
            ModifyGraph  /W=FittedSlopeTmp
rgb($("tmp_DispFitSlopeWaveG"+num2str(i)))=(GROUPCOLORRED[i],GROUPCOLORG
REEN[i],GROUPCOLORBLUE[i])
            ErrorBars /W=FittedSlopeTmp $("tmp_DispFitSlopeWaveG"+num2str(i))
Y,wave=($("tmp_DispFitSlopeWaveSEMG"+num2str(i)),$("tmp_DispFitSlopeWaveSEMG"
+num2str(i)))
            i+=1
        while(i<NumberOfGroups+1)
        Label  /W=FittedSlopeTmp left "Fit Slope";Label /W=FittedSlopeTmp bottom "Task
number"
        SetAxis /W=FittedSlopeTmp Bottom  1, NumberOfDataPoints
        ModifyGraph /W=FittedSlopeTmp
nticks(bottom)=min(NumberOfDataPoints,MAXNUMBEROFTICKS)
    endif

duplicate /o tmp2D_DispFitHalfWaveSEM, TMPTEST
    if(DispFitHalf)
        i=0
        do
            tmp2D_DispFitHalfWave[][i]/=Max(1,animalsPerGroup[i])

tmp2D_DispFitHalfWaveSEM[][i]=(animalsPerGRoup[i]<2?0:sqrt((tmp2D_DispFitHalfWave
SEM[p][i]-animalsPerGroup[i]*tmp2D_DispFitHalfWave[p][i]^2)/(animalsPerGroup[i]-
1))/sqrt(animalsPerGroup[i]))
            make /N=(NumberOfDataPoints) /O tmpWave
            tmpWave=tmp2D_DispFitHalfWave[p][i]
```

```
         duplicate /O tmpWave, $("tmp_DispFitHalfWaveG"+num2str(i))
         SetScale/P x 1,1,"", $("tmp_DispFitHalfWaveG"+num2str(i))
         tmpWave=tmp2D_DispFitHalfWaveSEM[p][i]
         duplicate /O tmpWave, $("tmp_DispFitHalfWaveSEMG"+num2str(i))
         SetScale/P x 1,1,"", $("tmp_DispFitHalfWaveSEMG"+num2str(i))
         appendtograph /W=FittedHalfTmp $("tmp_DispFitHalfWaveG"+num2str(i))
         ModifyGraph  /W=FittedHalfTmp
rgb($("tmp_DispFitHalfWaveG"+num2str(i)))=(GROUPCOLORRED[i],GROUPCOLORGR
EEN[i],GROUPCOLORBLUE[i])
         ErrorBars /W=FittedHalfTmp $("tmp_DispFitHalfWaveG"+num2str(i))
Y,wave=($("tmp_DispFitHalfWaveSEMG"+num2str(i)),$("tmp_DispFitHalfWaveSEMG"+nu
m2str(i)))
         i+=1
      while(i<NumberOfGroups+1)
      Label  /W=FittedHalfTmp left "Fit Half";Label /W=FittedHalfTmp bottom "Task
number"
      SetAxis /W=FittedHalfTmp Bottom 1, NumberOfDataPoints
      ModifyGraph /W=FittedHalfTmp
nticks(bottom)=min(NumberOfDataPoints,MAXNUMBEROFTICKS)
   endif


   if(DispAvg)
      i=0
      do
         tmp2D_DispAvgWave[][i]/=Max(1,animalsPerGroup[i])

tmp2D_DispAvgWaveSEM[][i]=(animalsPerGRoup[i]<2?0:sqrt((tmp2D_DispAvgWaveSEM
[p][i]-animalsPerGroup[i]*tmp2D_DispAvgWave[p][i]^2)/(animalsPerGroup[i]-
1))/sqrt(animalsPerGroup[i]))
         make /N=(NumberOfDataPoints) /O tmpWave
         tmpWave=tmp2D_DispAvgWave[p][i]
         duplicate /O tmpWave, $("tmp_DispAvgWaveG"+num2str(i))
         SetScale/P x 1,1,"", $("tmp_DispAvgWaveG"+num2str(i))
         tmpWave=tmp2D_DispAvgWaveSEM[p][i]
         duplicate /O tmpWave, $("tmp_DispAvgWaveSEMG"+num2str(i))
         SetScale/P x 1,1,"", $("tmp_DispAvgWaveSEMG"+num2str(i))
         appendtograph /W=AvgDiffTmp $("tmp_DispAvgWaveG"+num2str(i))
         ModifyGraph  /W=AvgDiffTmp
rgb($("tmp_DispAvgWaveG"+num2str(i)))=(GROUPCOLORRED[i],GROUPCOLORGREE
N[i],GROUPCOLORBLUE[i])
         ErrorBars /W=AvgDiffTmp $("tmp_DispAvgWaveG"+num2str(i))
Y,wave=($("tmp_DispAvgWaveSEMG"+num2str(i)),$("tmp_DispAvgWaveSEMG"+num2st
r(i)))
         i+=1
      while(i<NumberOfGroups+1)
      Label  /W=AvgDiffTmp left "Fit Half";Label /W=FittedHalfTmp bottom "Task number"
      SetAxis /W=AvgDiffTmp Bottom 1, NumberOfDataPoints
      ModifyGraph /W=AvgDiffTmp
nticks(bottom)=min(NumberOfDataPoints,MAXNUMBEROFTICKS)
```

```
   endif

   if(DispPercCorr)
      DisplayPercentageCorrectGroups()
   endif

   if(DispPercLick)
      DisplayPercentageLickedGroups()
   endif

   if(DispITI)
      DisplayITIGroups()
   endif

   if(DispFirstDetect)
      i=0
      do
         tmp2D_DispFirstDetectWave[][i]/=Max(1,animalsPerGroup[i])

tmp2D_DispFirstDetectWaveSEM[][i]=(animalsPerGRoup[i]<2?0:sqrt((tmp2D_DispFirstDet
ectWaveSEM[p][i]-
animalsPerGroup[i]*tmp2D_DispFirstDetectWave[p][i]^2)/(animalsPerGroup[i]-
1))/sqrt(animalsPerGroup[i]))
         make /N=(NumberOfDataPoints) /O tmpWave
         tmpWave=tmp2D_DispFirstDetectWave[p][i]
         duplicate /O tmpWave, $("tmp_DispFirstDetectWaveG"+num2str(i))
         SetScale/P x StartTask,1,"", $("tmp_DispFirstDetectWaveG"+num2str(i))
         tmpWave=tmp2D_DispFirstDetectWaveSEM[p][i]
         duplicate /O tmpWave, $("tmp_DispFirstDetectWaveSEMG"+num2str(i))
         SetScale/P x 1,1,"", $("tmp_DispFirstDetectWaveSEMG"+num2str(i))
         appendtograph /W=FirstDetectTmp $("tmp_DispFirstDetectWaveG"+num2str(i))
         ModifyGraph  /W=FirstDetectTmp
rgb($("tmp_DispFirstDetectWaveG"+num2str(i)))=(GROUPCOLORRED[i],GROUPCOLOR
GREEN[i],GROUPCOLORBLUE[i])
         ErrorBars /W=FirstDetectTmp $("tmp_DispFirstDetectWaveG"+num2str(i))
Y,wave=($("tmp_DispFirstDetectWaveSEMG"+num2str(i)),$("tmp_DispFirstDetectWaveS
EMG"+num2str(i)))
         i+=1
      while(i<NumberOfGroups+1)
      Label  /W=FirstDetectTmp left "FirstDetect (ms)";Label /W=FirstDetectTmp bottom
"Task number"
      SetAxis /W=FirstDetectTmp Bottom 1, NumberOfDataPoints
      ModifyGraph /W=FirstDetectTmp
nticks(bottom)=min(NumberOfDataPoints,MAXNUMBEROFTICKS)
   endif


End
```

```
Function DisplayPercentageCorrectGroups()
   variable /g WhichAnimal, CurrentTask,StartTask,EndTask,WhichAnimalStart,
WhichAnimalStop, NumberOfAnimals, MAXNUMBEROFGROUPS
   variable /g LengthOfTImeSampling,
TrialsAveraged,NumberOfTasks,startTrials,endTrials, NumberOfGroups
   string /g RecentDisplayWindowsList
   make /N=(NumberOfAnimals+1) /O GroupOfAnimal
   make /N=(NumberOfTasks) /O TrialsPerTask, FinalValveTimeArray
   variable /g MAXNUMBEROFGROUPS,MAXNUMBEROFTICKS
   make /N=(MAXNUMBEROFGROUPS) /O
GROUPCOLORRED,GROUPCOLORGREEN,GROUPCOLORBLUE
   make /N=(MAXNUMBEROFGROUPS) /o animalsPerGroup=0


   make /O  /N=(NumberOfTasks) TrialsPerTask
   DoWindow /K PercentageCorrectTmp

   variable i,j,NumberOfDataPoints=0,WaveEntryIndex, AnimalCount, PercCorrTmp
   i=StartTask-1
   do
      numberOfDataPoints+=round(TrialsPerTask[i]/TrialsAveraged+0.49)     // if trials are
averaged in 100 a piece but only 250 trials -> nevertheless three entries
      i+=1
   while(i<EndTask-1+0.1)

//    make /N=(numberOfDataPoints) /O PercentCorrect, PercentCorrectTmp
   display /W=(3,240,220,320)/K=1 as "Percentage Correct"
   DoWindow/C PercentageCorrectTmp
   ModifyGraph width=150,height=30
   RecentDisplayWindowsList=RecentDisplayWindowsList+"PercentageCorrectTmp;"

   make /N=(NumberOfDataPoints,NumberOfGroups+1) /O
tmp2D_DispPercCorrWave=0,tmp2D_DisppercCorrWaveSEM=0
   make /N=(numberOfDataPoints) /O /T WhichIsTheTask
   make /N=(NumberOfDataPoints,NumberOfAnimals+1) /O ForANOVA_PercCorr

   AnimalCount = 1
   do
         if(GroupOfAnimal[AnimalCount]>0)
              WhichAnimal=AnimalCount

              CurrentTask=StartTask
              WaveEntryIndex=0
              animalsPerGroup[GroupOfAnimal[AnimalCount]]+=1
              do
                  j=0
                  GetLickPattern()
                 do
                     startTrials=j*TrialsAveraged+1
```

```
                    endTrials=min(TrialsPerTask[CurrentTask-1],startTrials+TrialsAveraged)
                    PercCorrTmp=CalculateIsCorrect()

tmp2D_DispPercCorrWave[WaveEntryIndex][GroupOfAnimal[AnimalCount]]+=PercCorrT
mp
                      ForANOVA_PercCorr[WaveEntryIndex][AnimalCount]=PercCorrTmp

tmp2D_DispPercCorrWaveSEM[WaveEntryIndex][GroupOfAnimal[AnimalCount]]+=PercC
orrTmp^2

                    j+=1
                    WaveEntryIndex+=1
                 while(j<round(TrialsPerTask[CurrentTask-1]/TrialsAveraged+0.49))
                 CurrentTask+=1
               while(CurrentTask<EndTask+0.1)
            endif // animalGroup>0
        AnimalCount+=1
      While(AnimalCount<NumberOfAnimals+0.1)
      i=0
      do
        tmp2D_DispPercCorrWave[][i]/=Max(1,animalsPerGroup[i])

tmp2D_DispPercCorrWaveSEM[][i]=(animalsPerGRoup[i]<2?0:sqrt((tmp2D_DispPercCorr
WaveSEM[p][i]-
animalsPerGroup[i]*tmp2D_DispPercCorrWave[p][i]^2)/(animalsPerGroup[i]-
1))/sqrt(animalsPerGroup[i]))
        make /N=(NumberOfDataPoints) /O tmpWave
        tmpWave=tmp2D_DispPercCorrWave[p][i]
        duplicate /O tmpWave, $("tmp_DispPercCorrWaveG"+num2str(i))
        SetScale/P x 1,1,"", $("tmp_DispPercCorrWaveG"+num2str(i))
        tmpWave=tmp2D_DispPercCorrWaveSEM[p][i]
        duplicate /O tmpWave, $("tmp_DispPercCorrWaveSEMG"+num2str(i))
        SetScale/P x 1,1,"", $("tmp_DispPercCorrWaveSEMG"+num2str(i))
        appendtograph /W=PercentageCorrectTmp
$("tmp_DispPercCorrWaveG"+num2str(i))
        ModifyGraph  /W=PercentageCorrectTmp
rgb($("tmp_DispPercCorrWaveG"+num2str(i)))=(GROUPCOLORRED[i],GROUPCOLORG
REEN[i],GROUPCOLORBLUE[i])
        ErrorBars /W=PercentageCorrectTmp $("tmp_DispPercCorrWaveG"+num2str(i))
Y,wave=($("tmp_DispPercCorrWaveSEMG"+num2str(i)),$("tmp_DispPercCorrWaveSEM
G"+num2str(i)))
        i+=1
      while(i<NumberOfGroups+1)
      Label  /W=PercentageCorrectTmp left "Percentage Correct";Label
/W=PercentageCorrectTmp bottom "Task number"
      SetAxis /W=PercentageCorrectTmp Bottom 1, NumberOfDataPoints
      ModifyGraph /W=PercentageCorrectTmp
nticks(bottom)=min(NumberOfDataPoints,MAXNUMBEROFTICKS)
```

```
//       PercentCorrect=0
//       PercentCorrectTmp=0
//       AnimalCount = 1
//       do
//           WhichAnimal = AnimalCount
//           if(GroupOfAnimal[AnimalCount]==GroupCount)
//              CurrentTask=StartTask
//              WaveEntryIndex=0
//              do
//                     GetLickPattern()
//                 j=0
//                 do
//                     startTrials=j*TrialsAveraged+1
//                     endTrials=min(TrialsPerTask[CurrentTask-1],startTrials+TrialsAveraged)
//                     PercentCorrectTmp[WaveEntryIndex]=CalculateIsCorrect()
//                     j+=1
//                     WaveEntryIndex+=1
//                 while(j<round(TrialsPerTask[CurrentTask-1]/TrialsAveraged+0.49))
//                 CurrentTask+=1
//              while(CurrentTask<EndTask+0.1)
//                  PercentCorrect+=PercentCorrectTmp
//           endif
//         AnimalCount+=1
//      While(AnimalCount<NumberOfAnimals+0.1)
//       if (AnimalsPerGroup[GroupCount]>0)
//             PercentCorrect/=AnimalsPerGroup[GroupCount]
//                duplicate /O PercentCorrect,
$("PercentCorrectTmpGroup_"+num2str(GroupCount))
//                appendtograph  $("PercentCorrectTmpGroup_"+num2str(GroupCount))
//       endif
//       GroupCount+=1
//   while(GroupCount<MAXNUMBEROFGROUPS+1)

END
Function DisplayPercentagelickedGroups()
   variable /g WhichAnimal, CurrentTask,StartTask,EndTask,WhichAnimalStart,
WhichAnimalStop, NumberOfAnimals, MAXNUMBEROFGROUPS
   variable /g LengthOfTImeSampling,
TrialsAveraged,NumberOfTasks,startTrials,endTrials, NumberOfGroups
   string /g RecentDisplayWindowsList
   make /N=(NumberOfAnimals+1) /O GroupOfAnimal
   make /N=(NumberOfTasks) /O TrialsPerTask, FinalValveTimeArray
   variable /g MAXNUMBEROFGROUPS,MAXNUMBEROFTICKS
   make /N=(MAXNUMBEROFGROUPS) /O
GROUPCOLORRED,GROUPCOLORGREEN,GROUPCOLORBLUE
   make /N=(MAXNUMBEROFGROUPS) /o animalsPerGroup=0
```

```
    make /O  /N=(NumberOfTasks) TrialsPerTask
    DoWindow /K PercentageLickedTmp

    variable i,j,NumberOfDataPoints=0,WaveEntryIndex, AnimalCount, PercLickTmp
    i=StartTask-1
    do
       numberOfDataPoints+=round(TrialsPerTask[i]/TrialsAveraged+0.49)     // if trials are
averaged in 100 a piece but only 250 trials -> nevertheless three entries
       i+=1
    while(i<EndTask-1+0.1)

    display /W=(3,240,220,320)/K=1 as "Percentage Licked"
    DoWindow/C PercentageLickedTmp
    ModifyGraph width=150,height=30
    RecentDisplayWindowsList=RecentDisplayWindowsList+"PercentageLickedTmp;"

    make /N=(NumberOfDataPoints,NumberOfGroups+1) /O
tmp2D_DispPercLickWave=0,tmp2D_DisppercLickWaveSEM=0
    make /N=(numberOfDataPoints) /O /T WhichIsTheTask
    make /N=(NumberOfDataPoints,NumberOfAnimals+1) /O ForANOVA_PercLick

    AnimalCount = 1
    do
          if(GroupOfAnimal[AnimalCount]>0)
               WhichAnimal=AnimalCount

               CurrentTask=StartTask
               WaveEntryIndex=0
               animalsPerGroup[GroupOfAnimal[AnimalCount]]+=1
               do
                   j=0
                   GetLickPattern()
                   do
                       startTrials=j*TrialsAveraged+1
                       endTrials=min(TrialsPerTask[CurrentTask-1],startTrials+TrialsAveraged)
                       PercLickTmp=CalculateIsLicked()

tmp2D_DispPercLickWave[WaveEntryIndex][GroupOfAnimal[AnimalCount]]+=PercLickTm
p
                           ForANOVA_PercLick[WaveEntryIndex][AnimalCount]=PercLickTmp

tmp2D_DispPercLickWaveSEM[WaveEntryIndex][GroupOfAnimal[AnimalCount]]+=PercLi
ckTmp^2

                       j+=1
                       WaveEntryIndex+=1
                  while(j<round(TrialsPerTask[CurrentTask-1]/TrialsAveraged+0.49))
                  CurrentTask+=1
               while(CurrentTask<EndTask+0.1)
           endif // animalGroup>0
```

```
      AnimalCount+=1
   While(AnimalCount<NumberOfAnimals+0.1)
    i=0
    do
       tmp2D_DispPercLickWave[][i]/=Max(1,animalsPerGroup[i])

tmp2D_DispPercLickWaveSEM[][i]=(animalsPerGRoup[i]<2?0:sqrt((tmp2D_DispPercLick
WaveSEM[p][i]-
animalsPerGroup[i]*tmp2D_DispPercLickWave[p][i]^2)/(animalsPerGroup[i]-
1))/sqrt(animalsPerGroup[i]))
       make /N=(NumberOfDataPoints) /O tmpWave
       tmpWave=tmp2D_DispPercLickWave[p][i]
       duplicate /O tmpWave, $("tmp_DispPercLickWaveG"+num2str(i))
       SetScale/P x 1,1,"", $("tmp_DispPercLickWaveG"+num2str(i))
       tmpWave=tmp2D_DispPercLickWaveSEM[p][i]
       duplicate /O tmpWave, $("tmp_DispPercLickWaveSEMG"+num2str(i))
      SetScale/P x 1,1,"", $("tmp_DispPercLickWaveSEMG"+num2str(i))
      appendtograph /W=PercentageLickedTmp $("tmp_DispPercLickWaveG"+num2str(i))
      ModifyGraph  /W=PercentageLickedTmp
rgb($("tmp_DispPercLickWaveG"+num2str(i)))=(GROUPCOLORRED[i],GROUPCOLORG
REEN[i],GROUPCOLORBLUE[i])
      ErrorBars /W=PercentageLickedTmp $("tmp_DispPercLickWaveG"+num2str(i))
Y,wave=($("tmp_DispPercLickWaveSEMG"+num2str(i)),$("tmp_DispPercLickWaveSEMG
"+num2str(i)))
      i+=1
    while(i<NumberOfGroups+1)
    Label  /W=PercentageLickedTmp left "Percentage Licked";Label
/W=PercentageLickedTmp bottom "Task number"
    SetAxis /W=PercentageLickedTmp Bottom 1, NumberOfDataPoints
    ModifyGraph /W=PercentageLickedTmp
nticks(bottom)=min(NumberOfDataPoints,MAXNUMBEROFTICKS)


END

Function DisplayITIGroups()
   variable /g WhichAnimal, CurrentTask,StartTask,EndTask,WhichAnimalStart,
WhichAnimalStop, NumberOfAnimals, MAXNUMBEROFGROUPS
   variable /g LengthOfTImeSampling,
TrialsAveraged,NumberOfTasks,startTrials,endTrials, NumberOfGroups
   string /g RecentDisplayWindowsList
   make /N=(NumberOfAnimals+1) /O GroupOfAnimal
   make /N=(NumberOfTasks) /O TrialsPerTask, FinalValveTimeArray
   variable /g MAXNUMBEROFGROUPS,MAXNUMBEROFTICKS
   make /N=(MAXNUMBEROFGROUPS) /O
GROUPCOLORRED,GROUPCOLORGREEN,GROUPCOLORBLUE
   make /N=(MAXNUMBEROFGROUPS) /o animalsPerGroup=0


   make /O  /N=(NumberOfTasks) TrialsPerTask
```

```
   DoWindow /K ITITmp

   variable i,j,NumberOfDataPoints=0,WaveEntryIndex, AnimalCount, ITITmp
   i=StartTask-1
   do
      numberOfDataPoints+=round(TrialsPerTask[i]/TrialsAveraged+0.49)     // if trials are
averaged in 100 a piece but only 250 trials -> nevertheless three entries
      i+=1
   while(i<EndTask-1+0.1)

   display /W=(3,240,220,320)/K=1 as "ITI"
   DoWindow/C ITITmp
   ModifyGraph width=150,height=30
   RecentDisplayWindowsList=RecentDisplayWindowsList+"ITITmp;"

   make /N=(NumberOfDataPoints,NumberOfGroups+1) /O
tmp2D_DispITIWave=0,tmp2D_DispITIWaveSEM=0
   make /N=(numberOfDataPoints) /O /T WhichIsTheTask
   make /N=(NumberOfDataPoints,NumberOfAnimals+1) /O ForANOVA_ITI

   AnimalCount = 1
   do
         if(GroupOfAnimal[AnimalCount]>0)
              WhichAnimal=AnimalCount

              CurrentTask=StartTask
              WaveEntryIndex=0
              animalsPerGroup[GroupOfAnimal[AnimalCount]]+=1
              do
                  j=0
                  GetTimes()
                  do
                      startTrials=j*TrialsAveraged+1
                      endTrials=min(TrialsPerTask[CurrentTask-1],startTrials+TrialsAveraged)
                      ITITmp=CalculateTimes()

tmp2D_DispITIWave[WaveEntryIndex][GroupOfAnimal[AnimalCount]]+=ITITmp
                          ForANOVA_ITI[WaveEntryIndex][AnimalCount]=ITITmp

tmp2D_DispITIWaveSEM[WaveEntryIndex][GroupOfAnimal[AnimalCount]]+=ITITmp^2
                      j+=1
                      WaveEntryIndex+=1
                  while(j<round(TrialsPerTask[CurrentTask-1]/TrialsAveraged+0.49))
                  CurrentTask+=1
              while(CurrentTask<EndTask+0.1)
          endif // animalGroup>0
      AnimalCount+=1
   While(AnimalCount<NumberOfAnimals+0.1)
    i=0
    do
```

```
        tmp2D_DispITIWave[][i]/=Max(1,animalsPerGroup[i])

tmp2D_DispITIWaveSEM[][i]=(animalsPerGRoup[i]<2?0:sqrt((tmp2D_DispITIWaveSEM[p]
[i]-animalsPerGroup[i]*tmp2D_DispITIWave[p][i]^2)/(animalsPerGroup[i]-
1))/sqrt(animalsPerGroup[i]))
        make /N=(NumberOfDataPoints) /O tmpWave
        tmpWave=tmp2D_DispITIWave[p][i]
        duplicate /O tmpWave, $("tmp_DispITIWaveG"+num2str(i))
        SetScale/P x 1,1,"", $("tmp_DispITIWaveG"+num2str(i))
        tmpWave=tmp2D_DispITIWaveSEM[p][i]
        duplicate /O tmpWave, $("tmp_DispITIWaveSEMG"+num2str(i))
       SetScale/P x 1,1,"", $("tmp_DispPercLickWaveSEMG"+num2str(i))
       appendtograph /W=ITITmp $("tmp_DispITIWaveG"+num2str(i))
       ModifyGraph  /W=ITITmp
rgb($("tmp_DispITIWaveG"+num2str(i)))=(GROUPCOLORRED[i],GROUPCOLORGREEN
[i],GROUPCOLORBLUE[i])
       ErrorBars /W=ITITmp $("tmp_DispITIWaveG"+num2str(i))
Y,wave=($("tmp_DispITIWaveSEMG"+num2str(i)),$("tmp_DispITIWaveSEMG"+num2str(i)
))
       i+=1
     while(i<NumberOfGroups+1)
     Label  /W=ITITmp left "ITI";Label /W=ITITmp bottom "Task number"
     SetAxis /W=ITITmp Bottom 1, NumberOfDataPoints
     ModifyGraph /W=ITITmp
nticks(bottom)=min(NumberOfDataPoints,MAXNUMBEROFTICKS)


END

Function GetTimes()
    string /g IndividualTaskName,SPECIFICWAVEENDING
    variable /g CurrentTask, WhichAnimal, LengthOfTimeSampling, NumberOfTasks,
MAXVALVNUMBER, CurrentTaskNumberOfTrials
    duplicate /o /T $IndividualTaskName, tmpWave1
    variable i, tmpLogic,j, numPTrials=0, numMTrials=0
    string PathSpecifier
    make /N=(NumberOfTasks) /O NumSPlusValves, NumSMinusValves
    make /O /T TrialTime

    PathSpecifier=tmpWave1[WhichAnimal-1][CurrentTask-1]
    killwaves TrialTime
    LoadWave/H/O/P=DataPath/K=0 ":"+PathSpecifier+"
Folder:TrialTime."+SPECIFICWAVEENDING

END


Function CalculateTimes()
    variable /g
CurrentTask,CurrentTaskNumberOfTrials,NumberOfTasks,startTrials,endTrials
```

```
   make /N=(CurrentTaskNumberOfTrials) /O /T TrialTime
   variable i,numTrials=0, deltaTTmp, deltaT=0
   i=startTrials
   do
         deltaTTmp=ConvertTimeToNum(TrialTime[i])-ConvertTimeToNum(TrialTime[i-1])
         if((deltaTTmp<60*20)*(deltaTTmp>0)) // ITI shorter than 20 min
              numTrials+=1
              deltaT+=deltaTTmp
         endif
      i+=1
   while(i<endTrials)
   return deltaT/numTrials
END

Function ConvertTimeToNum(str)
string str
   variable h,m,s
   sscanf str, "%f:%f:%f", h,m,s
   return h*3600+m*60+s

end

#pragma rtGlobals=1                  // Use modern global access method.

function StartAnalysis()  // creates Notebook file
        string /g BaseName, AnalysisName, AnalysisNotebookName =
baseName+"_NoteBook"
        variable /g StartTask, EndTask, WhichAnimal
        variable /g DispPercCorr, DispMax, DispAvg, DispFitMax, DispFitSlope, DispFitHalf,
DispSingle, DispPM, DispFirstDetect
        // kills old notebook (if any), creates new one and renames it so that title sort of
matches
        // the Name of Analysis and the actual name is based on base name
        DoWindow /K $AnalysisNotebookName
        NewNotebook
/N=$AnalysisNotebookName/F=1/V=1/K=0/W=(5.25,40.25,504.75,335)
        Notebook $AnalysisNotebookName defaultTab=20, statusWidth=238,
pageMargins={72,72,72,72}
        Notebook $AnalysisNotebookName font="Arial", fSize=10, fStyle=0,
textRGB=(0,0,0)
        Notebook $AnalysisNotebookName defaultTab=36, statusWidth=238,
pageMargins={72,72,72,72}
        Notebook $AnalysisNotebookName showRuler=0, rulerUnits=1, updating={1, 60}
        DoWindow/T/B $AnalysisNotebookName, AnalysisName + " Notebook"
        DoWindow/F $AnalysisNotebookName
end

#pragma rtGlobals=1                  // Use modern global access method.
#include <Concatenate Waves>
Function statUTest(wv1,wv2 )
```

```
string wv1,wv2
variable j=1,ji,jt,t,rank,n,c,U1,U2,N1,N2,muU,sigmaU,Z

n=numpnts($wv1)+numpnts($wv2)
duplicate /O $wv1,wv
duplicate /o $wv1, wv1Which
duplicate /o $wv2, wv2Which

wv1Which=1
wv2Which=2
duplicate /o wv1Which, wvWhich

//wavestats wv
//wavestats $wv2

ConcatenateWaves("wv",wv2)
ConcatenateWaves("wvWhich","wv2Which")

Sort wv, wvWhich, wv

duplicate /O wv, ranks
ranks=0
// now sorted, put ranks instead of number
rank=1
j=0
do
  ji=wv[j]
  c=0
  do
        c+=1
        j+=1
  while((wv[j]==ji)*(j<n))
  ji=j-c
  rank=(j+j-c+1)/2
  do
        ranks[ji]=rank
        ji+=1
  while(ji<j+1)

while(j<n)

N1=numpnts($wv1)
N2=numpnts($wv2)

duplicate /O wvWhich, tmp
tmp=(wvWhich<2)*ranks                         // calculating U for first var
U1=N1*N2+N1*(N1+1)/2-sum(tmp,-inf,inf)
tmp=(wvWhich>1)*ranks
U2=N1*N2+N2*(N2+1)/2-sum(tmp,-inf,inf)
U1=min(abs(U1),abs(U2))
```

```
muU=N1*N2/2
sigmaU=sqrt(N1*N2*(N1+N2+1)/12)
Z=abs((U1-muU)/sigmaU)
//print Z, (1-2*IntNorm(Z))
return (1-2*IntNorm(Z))

end

function StartStats()
make/D/N=20000/O Norm
SetScale/P x 0,0.0003,"", Norm
Norm=1/sqrt(2*pi)*exp(-x^2/2)
Integrate /T Norm
END

function IntNorm(x1)
variable x1
WAVE Norm=Norm
return Norm(x1)
end


// calculates whether wv1 and wv2 are differentially deviating from 1 (below 1)
function StatSortTest(wv1,wv2)
string wv1,wv2
	variable n1,n2,N,M
	duplicate /o $wv1, tmp
	tmp=(tmp<0.995)
	n1=sum(tmp,-inf,inf)
	duplicate /o $wv2, tmp
	tmp=(tmp<0.995)
	n2=sum(tmp,-inf,inf)
	M=min(n1,n2)
	N=n1+n2
	return statSortTestHlp(N,M)
end

// calculates whether wv1 and wv2 are differentially deviating from 1 (below 1)
function StatSortTestLick(wv1,wv2)
string wv1,wv2
	variable n1,n2,N,M
	duplicate /o $wv1, tmp
	tmp=(tmp<0.005)
	n1=sum(tmp,-inf,inf)
	duplicate /o $wv2, tmp
	tmp=(tmp<0.005)
	n2=sum(tmp,-inf,inf)
	M=min(n1,n2)
	N=n1+n2
	return statSortTestHlp(N,M)
```

```
end

// calculates whether wv1 and wv2 are differentially deviating from 1 (below 1)
function StatSortTestBin(wv1,wv2)
string wv1,wv2
	variable n1,n2,N,M
	duplicate /o $wv1, tmp
	tmp=(tmp<0.04)
	n1=sum(tmp,-inf,inf)
	duplicate /o $wv2, tmp
	tmp=(tmp<0.04)
	n2=sum(tmp,-inf,inf)
	M=min(n1,n2)
	N=n1+n2
	return statSortTestHlp(N,M)
end



end
// N balls can be placed in two bowls; calculate probability that >=M balls are in bowl 1
function statSortTestHlp(N,M )
variable N,M
variable i=0,sumTot=0, sumPart=0
do
//	sumTot+=binomial(N,i)
	sumPart+=binomial(N,i)
	i+=1
while(i<=M)
//do
//	sumTot+=binomial(N,i)
//	i+=1
//while(i<=N)
return sumPart/2^N //print sumTot, 2^N, sumPart, sumPart/sumTot
end

#pragma rtGlobals=1		// Use modern global access method.
#pragma version = 1.00
#pragma igorVersion = 4.00

#include <SaveRestoreWindowCoords>
//MODIFIED ANOVA

//************************************
// one-way and two-way ANOVA
//
// One-way ANOVA is pretty complete:
//    Handles data sets of different sizes.
//    Uses Levene's median test to check homogeneity of variance.
//    Offers Student-Newman-Keuls or Tukey HSD test for pair-wise comparisons.
```

```
//	Offers Dunnett's test for pairwise comparisons to a control.
//	Does NOT do LSD or Bonferroni's test for pair-wise comparisons because my reading
indicates that,
//		while they are common tests, they are obsolete and really shouldn't be used any
more.
//
// Two-way ANOVA may be useful to some, but is not really a full-fledged two-way ANOVA
solution.
//	It handles MxN data with one sample per cell without interaction effects.
//	It handles LxMxN data with interaction effects.
//	Option to apply one of several transformations before doing ANOVA.
//	Does NOT handle:
//		Missing data.
//		Pair-wise comparisons.
//		Data sets with variable number of samples in each cell.
//		ANCOVA
//		MANOVA
//***********************************

//***********************************
// Possible future enhancements:
// 1) ANOVA for curve fitting, especially for line and poly fits
// 2) Sequential polynomial fits.
// 3) Stepwise regression.
//***********************************


Menu "Analysis"
	Submenu "ANOVA - ATS"
		"One-way", DoOneWayANOVAPanel()
		"Two-way", DoTwoWayANOVAPanel()
		"Unload ANOVA Package", Execute/P "DELETEINCLUDE
<ANOVA_ATS>";Execute/P "COMPILEPROCEDURES "
	end
end

Function DoOneWayANOVAPanel()
	initANOVAGlobals()		// this has  been written so that if variables exist, it
doesn't over-write them

	if (WinType("ANOVAOneWayPanel") != 7)
		fANOVAOneWayPanel()
	else
		DoWindow/F ANOVAOneWayPanel
	endif
end

Function DoTwoWayANOVAPanel()
	// two-way has no globals to initialize (at least not yet)
```

```
	if (WinType("ANOVATwoWayPanel") != 7)
		fTwoWayANOVAPanel()
	else
		DoWindow/F ANOVATwoWayPanel
	endif
end

static Function initANOVAGlobals()

	String saveDF = GetDatafolder(1)
	SetDatafolder root:
	NewDatafolder/O/S Packages
	NewDatafolder/O/S WM_ANOVA

	Variable/G UseMedianForLeveneTest = 1			// set to zero to use absolute
deviations from the mean, set to one for absolute deviations from the median

	SVAR/Z WaveBaseName
	if (!SVAR_Exists(WaveBaseName))
		String/G WaveBaseName="wave"
	endif

	NVAR/Z WaveSourceMode
	if (!NVAR_Exists(WaveSourceMode))
		Variable/G WaveSourceMenuMode = 1
	endif
	NVAR/Z LeveneChecked
	if (!NVAR_Exists(LeveneChecked))
		Variable/G LeveneChecked = 1
	endif
	NVAR/Z TukeyHSDChecked
	if (!NVAR_Exists(TukeyHSDChecked))
		Variable/G TukeyHSDChecked = 1
	endif
	NVAR/Z SNKChecked
	if (!NVAR_Exists(SNKChecked))
		Variable/G SNKChecked = 0
	endif
	NVAR/Z DunnetsChecked
	if (!NVAR_Exists(DunnetsChecked))
		Variable/G DunnetsChecked = 0
	endif

	String/G TargetWindow=WinName(0,3)

	SetDatafolder $saveDF
end

static Function/S SetANOVAFolder(BaseName)
	String BaseName
```

```
	String saveDF = GetDatafolder(1)
	SetDatafolder root:Packages:WM_ANOVA:
	NewDatafolder/O/S $(BaseName+"_ANOVA")
	return saveDF
end


//****************************
// ONE-WAY ANOVA
//****************************

// Does an 1-way ANOVA report. Expects data in the top table window.
// Each treatment will be in a separate wave. All waves in the table will be analyzed.

static Function TukeyHSDTest(MeansWave, nSamplesWave,
meanSquareWithinTreatments, DFWithinTreatments)
	Wave MeansWave, nSamplesWave
	Variable meanSquareWithinTreatments, DFWithinTreatments

	// MeansWave and nSamplesWave won't have NaN's because they are the result of
NaN-free processing
	Variable nTreatments = numpnts(MeansWave)
	if (numpnts(nSamplesWave) != nTreatments)
		return -1
	endif

	// Do paired comparisons of means
	Make/O/D/N=(nTreatments, nTreatments) MeanDif, Qstat, PairProb, MeanDifSE

	MeanDif = abs(MeansWave[p] - MeansWave[q])
	Variable i

	// Tukey HSD test
	// the following assumes equal variances, uses the pooled variance estimate
	MeanDifSE = (p==q) ? NaN :
sqrt(meanSquareWithinTreatments*((1/nSamplesWave[p])+(1/nSamplesWave[q])))
	Qstat = (p==q) ? NaN :
abs(MeanDif[p][q]/sqrt(meanSquareWithinTreatments/(2/(1/nSamplesWave[p] +
1/nSamplesWave[q]))))
	PairProb = (p==q) ? NaN :1-StudentizedRangeP(Qstat, nTreatments,
DFWithinTreatments)

	return 0
end

static Function DunnettTest(MeansWave, nSamplesWave, meanSquareWithinTreatments,
ControlTreatment)
	Wave MeansWave, nSamplesWave
	Variable meanSquareWithinTreatments, ControlTreatment
```

```
	// MeansWave and nSamplesWave won't have NaN's because they are the result of NaN-free processing
	Variable nTreatments = numpnts(MeansWave)
	if (numpnts(nSamplesWave) != nTreatments)
		return -1
	endif
	Variable DF = sum(nSamplesWave, -inf, inf)-nTreatments
	// Do paired comparisons of means
	Make/O/D/N=(nTreatments) MeanDif, Qstat, PairProb, MeanDifSE

	MeanDif = abs(MeansWave[p] - MeansWave[ControlTreatment])
	Variable i

	// Tukey HSD test
	// the following assumes equal variances, uses the pooled variance estimate
	MeanDifSE = (p==ControlTreatment) ? NaN : sqrt(meanSquareWithinTreatments*((1/nSamplesWave[p])+(1/nSamplesWave[ControlTreatment])))
	Qstat = (p==ControlTreatment) ? NaN : abs(MeanDif[p]/sqrt(meanSquareWithinTreatments/(1/(1/nSamplesWave[p] + 1/nSamplesWave[ControlTreatment]))))
	PairProb = (p==ControlTreatment) ? NaN :1-DunnettP(Qstat, nSamplesWave, DF)
	Variable/G ControlItem=ControlTreatment

	return 0
end

static Function NewmanKeulsTest(MeansWave, nSamplesWave, meanSquareWithinTreatments, DFWithinTreatments)
	Wave MeansWave, nSamplesWave
	Variable meanSquareWithinTreatments, DFWithinTreatments

	// MeansWave and nSamplesWave won't have NaN's because they are the result of NaN-free processing
	Variable nTreatments = numpnts(MeansWave)
	if (numpnts(nSamplesWave) != nTreatments)
		return -1
	endif

	Make/O/D/N=(nTreatments, nTreatments) MeanDif, Qstat, PairProb, MeanDifSE, Sig05, Sig01
	Make/O/D/N=(nTreatments) meanSortIndex
	MeanDif = abs(MeansWave[p] - MeansWave[q])
	MeanDifSE = (p==q) ? NaN : sqrt(meanSquareWithinTreatments*((1/nSamplesWave[p])+(1/nSamplesWave[q])))

	MakeIndex MeansWave, meanSortIndex			// sort in descending order
	// these values are guaranteed insignificant
	PairProb = 1
```

```
	QStat = 0
	sig05=0
	sig01=0

	Variable i,j,ii,jj
	Variable treatmentsSpanned, QQstat
	for (i = 0; i < nTreatments; i += 1)
		ii = meanSortIndex[i]
		for (j = nTreatments-1; j > i; j -= 1)
			jj = meanSortIndex[j]
			treatmentsSpanned = j-i+1
			Qstat[ii][jj] =
abs(MeanDif[ii][jj]/sqrt(meanSquareWithinTreatments/(2/(1/nSamplesWave[ii] +
1/nSamplesWave[jj]))))
			PairProb[ii][jj] = 1-StudentizedRangeP(Qstat[ii][jj], treatmentsSpanned,
DFWithinTreatments)
		endfor
	endfor
	Variable indexZero = meanSortIndex[0]
	for (i = nTreatments-1; i > 0; i -= 1)
		ii = meanSortIndex[i]
		if (PairProb[indexZero][ii] < .05)
			sig05[indexZero][ii] = 1
		else
			break
		endif
		if (PairProb[indexZero][ii] < .01 && PairProb[indexZero][meanSortIndex[i+1]]
< 0.1)
			sig01[indexZero][ii] = 1
		endif
	endfor
	for (i = 1; i < nTreatments; i += 1)
		ii = meanSortIndex[i]
		for (j = nTreatments-1; j > i; j -= 1)
			jj = meanSortIndex[j]
			if (sig01[meanSortIndex[i-1]][jj] && PairProb[ii][jj] < 0.01)
				sig01[ii][jj] = 1
			endif
			if (sig05[meanSortIndex[i-1]][jj] && PairProb[ii][jj] < 0.05)
				sig05[ii][jj] = 1
			else
				break
			endif
		endfor
	endfor
end

static Function ANOVAMedian(w)
	Wave w
```

```
	Duplicate/O w, MedianWave__
	Sort MedianWave__, MedianWave__			// sort puts any NaN's down at the 
end
	WaveStats/Q MedianWave__
//	Variable MidPoint = V_npnts/2 - 0.5
	Variable MidPoint = numpnts(MedianWave__)/2 - 0.5
	Variable retValue = MedianWave__[MidPoint]
	KillWaves/Z MedianWave__
	return retValue
end

static Function LeveneTestHOV(TreatmentWaves, LeveneFValue)
	String TreatmentWaves
	Variable &LeveneFValue
	
	NVAR/Z UseMedianForLeveneTest = 
root:Packages:WM_ANOVA:UseMedianForLeveneTest
	if (!NVAR_Exists(UseMedianForLeveneTest))
		Variable/G root:Packages:WM_ANOVA:UseMedianForLeveneTest=1
		NVAR/Z UseMedianForLeveneTest = 
root:Packages:WM_ANOVA:UseMedianForLeveneTest
	endif

	Variable i
	Variable nTreatments = ItemsInList(TreatmentWaves)
	Variable ClassMean
	String oneWave, oneWaveName, newList="", newWaveName
	for (i = 0; i < nTreatments; i += 1)
		oneWave = StringFromList(i, TreatmentWaves)
		Wave w = $oneWave
		oneWaveName = NameOfWave(w)			// just the name, not the path 
which is included in the TreatmentWaves list
		newWaveName = oneWaveName+"_d"
		Duplicate/O w, $newWaveName
		Wave wd = $newWaveName
		if (UseMedianForLeveneTest)
			ClassMean = ANOVAMedian(w)
		else
			WaveStats/Q w
			ClassMean = V_avg
		endif
		wd = abs(w[p]-ClassMean)
		newList += newWaveName+";"
	endfor
	Do1wayANOVA(newList, 0, 0, 0, 0,0)
	NVAR Fvalue
	LeveneFValue = Fvalue
	NVAR probF
	return probF
end
```

```
Function Do1wayANOVAOnWindow(WindowName, DoTukey, DoSNK, DoDunnett,
ControlColumn, DoLevene)
	String WindowName
	Variable DoTukey			// do Tukey HSD test for significance of all
pairwise comparisons
	Variable DoSNK				// do Newman Keuls test for significance of
all pairwise comparisons
	Variable DoDunnett			// do Dunnett's test for significance of all
treatments compared to a control
	Variable ControlColumn		// which table column is the control
	Variable DoLevene			//do Levene's test for homogeneity of variance

	if (strlen(WindowName) == 0)
		WindowName = WinName(0,3)
	endif
	if (strlen(WindowName) == 0)
		DoAlert 0, "Table does not exist for ANOVA"
		return -1
	endif
	String TreatmentWaves = ListWavesFromWindowFullPath(WindowName)
	if (strlen(TreatmentWaves) == 0)
		DoAlert 0, "No waves in window "+WindowName
		return -2
	endif

	initANOVAGlobals()
	String saveDF = SetANOVAFolder(WindowName)
	String/G theWindow = WindowName
	if (Do1wayANOVA(TreatmentWaves, DoTukey, DoSNK, DoDunnett,
ControlColumn, DoLevene) == 0)
		Do1WayANOVAReport(TreatmentWaves)
	endif

	SetDatafolder $saveDF
end

Function Do1wayANOVAOnBaseName(BaseName, DoTukey, DoSNK, DoDunnett,
ControlColumn, DoLevene)
	String BaseName
	Variable DoTukey			// do Tukey HSD test for significance of all
pairwise comparisons
	Variable DoSNK				// do Newman Keuls test for significance of
all pairwise comparisons
	Variable DoDunnett			// do Dunnett's test for significance of all
treatments compared to a control
	Variable ControlColumn		// which table column is the control
	Variable DoLevene			//do Levene's test for homogeneity of variance

	if (strlen(BaseName) == 0)
```

```
		DoAlert 0, "No base name provided for making a list of waves for ANOVA."
		return -1
	endif
	String TreatmentWaves = ListWavesFromBaseNameFullPath(BaseName)
	if (strlen(TreatmentWaves) == 0)
		DoAlert 0, "No waves using base name "+BaseName
		return -2
	endif

	initANOVAGlobals()
	String saveDF = SetANOVAFolder(BaseName)
	String/G theBaseName = BaseName
	if (Do1wayANOVA(TreatmentWaves, DoTukey, DoSNK, DoDunnett,
ControlColumn, DoLevene) == 0)
		Do1WayANOVAReport(TreatmentWaves)
	endif

	SetDatafolder $saveDF
end

Function Do1wayANOVA(TreatmentWaves, DoTukey, DoSNK, DoDunnett, ControlItem,
DoLevene)
	String TreatmentWaves		// list of waves to be included in ANOVA
	Variable DoTukey			// do Tukey HSD test for significance of all
pairwise comparisons
	Variable DoSNK				// do Newman Keuls (also known as
Student-Newman-Keuls or SNK) test for significance of all pairwise comparisons
	Variable DoDunnett			// do Dunnett's test for significance of all
treatments compared to a control
	Variable ControlItem		// which item in list of waves is the control
	Variable DoLevene			// do Levene's test for homogeneity of variance

	String WName
	Variable i,j
	Variable nTreatments = ItemsInList(TreatmentWaves)
	if (nTreatments < 2)
		DoAlert 0, "To do ANOVA, you must have two or more 1D waves. Each wave
should contain the data for one treatment."
		return -1
	endif
	for (i = 0; i < nTreatments; i += 1)
		WName = StringFromList(i, TreatmentWaves)
		Wave/Z w = $WName
		if (!WaveExists(w))
			DoAlert 0, "One-way ANOVA can't be done: the wave "+WName+"
does not exist."
			return -1
		endif
		if (WaveDims(w) > 1)
```

```
				DoAlert 0, "One-way ANOVA can't be done: the wave "+WName+"
has more than one dimension. Only 1D waves can be used."
				return -1
			endif
		endfor

		Make/O/D/N=(nTreatments) sumX, meanX, sumX2, meanSquareX,
sumRes2,variance, numSamples
		Variable totalN=0
		for (i = 0; i < nTreatments; i += 1)
			WName = StringFromList(i, TreatmentWaves)
			Wave/Z w = $WName
			Duplicate/O w, squares, resSquares
			squares = squares^2
			WaveStats/Q w
//			numSamples[i] = numpnts(w)
			numSamples[i] = V_npnts
//			sumX[i] = sum(w, -inf, inf)
			sumX[i] = V_avg*V_npnts
			meanX[i] = sumX[i]/numSamples[i]
//			sumX2[i] = sum(squares, -inf, inf)
			WaveStats/Q squares
			sumX2[i] = V_avg*V_npnts
			meanSquareX[i] = sumX[i]^2/numSamples[i]
//			meanSquareX[i] = V_avg
			resSquares = (w[p]-meanX[i])^2
//			sumRes2[i] = sum(resSquares,-inf,inf)
			WaveStats/Q resSquares
			sumRes2[i] = V_avg*V_npnts
			variance[i] = sumRes2[i]/(numSamples[i]-1)
			totalN += numSamples[i]
		endfor

		KillWaves/Z resSquares, squares

		// the use of the sum function below is OK because NaN's have already been
ignored
		Variable/G DFBetweenTreatments = nTreatments-1
		Variable/G DFWithinTreatments = totalN - nTreatments
		Variable/G sumSqBetweenTreatments = sum(meanSquareX, -inf, inf) - sum(sumX,-
inf, inf)^2/totalN
		Variable/G sumSqWithinTreatments = sum(sumX2,-inf,inf) - sum(meanSquareX,-
inf,inf)
		Variable/G meanSqBetweenTreatments =
sumSqBetweenTreatments/DFBetweenTreatments
		Variable/G meanSqWithinTreatments =
sumSqWithinTreatments/DFWithinTreatments
		Variable/G Fvalue = meanSqBetweenTreatments/meanSqWithinTreatments
		Variable/G probF = Fprob(Fvalue, DFBetweenTreatments, DFWithinTreatments)
```

```
	String saveDF = GetDatafolder(1)

	if (DoLevene)
		Variable LeveneP, LeveneF
		NewDatafolder/O/S LeveneTest
		LeveneP = LeveneTestHOV(TreatmentWaves, LeveneF)
		SetDatafolder $saveDF
		KillDatafolder LeveneTest
		Variable/G LeveneHOVP = LeveneP
		Variable/G LeveneHOVF = LeveneF
	endif

	if (DoTukey)
		NewDatafolder/O/S TukeyHSD
		TukeyHSDTest(meanX, numSamples, meanSqWithinTreatments,
DFWithinTreatments)
		SetDatafolder $saveDF
	else
		if (DatafolderExists("TukeyHSD"))
			KillDatafolder TukeyHSD
		endif
	endif

	if (DoSNK)
		NewDatafolder/O/S NewmanKeuls
		NewmanKeulsTest(meanX, numSamples, meanSqWithinTreatments,
DFWithinTreatments)
		SetDatafolder $saveDF
	else
		if (DatafolderExists("NewmanKeuls"))
			KillDatafolder NewmanKeuls
		endif
	endif

	if (DoDunnett)
		NewDatafolder/O/S Dunnett
		DunnettTest(meanX, numSamples, meanSqWithinTreatments, ControlItem)
		SetDatafolder $saveDF
	else
		if (DatafolderExists("Dunnett"))
			KillDatafolder Dunnett
		endif
	endif

	return 0
end

Function Do1WayANOVAReport(TreatmentWaves)
	String TreatmentWaves
//	SVAR AnalysisNotebookName = root:AnalysisNotebookName
```

```
SVAR/Z theWindow
String NameBase
if (SVAR_Exists(theWindow))
	NameBase = theWindow
else
	NameBase = GetDatafolder(0)
	Variable nameLen = strlen(NameBase)
	if (CmpStr(NameBase[0] , "'") == 0)
		NameBase = NameBase[1, nameLen-1]
		nameLen -= 1
	endif
	if (CmpStr(NameBase[nameLen-1] , "'") == 0)
		NameBase = NameBase[0, nameLen-2]
		nameLen -= 1
	endif
	if (CmpStr(NameBase[nameLen-6, nameLen-1], "_ANOVA") == 0)
		NameBase = NameBase[0, nameLen-7]
	endif
	NameBase = CleanupName(NameBase, 0 )
endif
Variable nTreatments = ItemsInList(TreatmentWaves)

Variable DataExists=1

NVAR/Z DFBetweenTreatments
if (!NVAR_Exists(DFBetweenTreatments))
	DataExists=0
endif
NVAR/Z DFWithinTreatments
if (!NVAR_Exists(DFWithinTreatments))
	DataExists=0
endif
NVAR/Z sumSqBetweenTreatments
if (!NVAR_Exists(sumSqBetweenTreatments))
	DataExists=0
endif
NVAR/Z sumSqWithinTreatments
if (!NVAR_Exists(sumSqWithinTreatments))
	DataExists=0
endif
NVAR/Z meanSqBetweenTreatments
if (!NVAR_Exists(meanSqBetweenTreatments))
	DataExists=0
endif
NVAR/Z meanSqWithinTreatments
if (!NVAR_Exists(meanSqWithinTreatments))
	DataExists=0
endif
NVAR/Z Fvalue
if (!NVAR_Exists(Fvalue))
```

```
			DataExists=0
		endif
		NVAR/Z probF
		if (!NVAR_Exists(probF))
			DataExists=0
		endif

		if (!DataExists)
			DoAlert 0,"Some data required for the ANOVA report is missing. Perhaps the
analysis hasn't been done."
			return -1
		endif

		String nbName = "Scrap"
		if (WinType(nbName) != 0)
			// use the previous report notebook, but clear out all the text
		else
			// make a new notebook window
			NewNotebook/F=1/N=$nbName
		endif

		Wave sumX, meanX,variance, numSamples

		ANOVAReportSetup(nbName)
		Notebook $nbName ruler=Normal, fSize=12, fStyle=1, text="One-way ANOVA
Report\r"
		Notebook $nbName ruler=Normal, text="\r"
		Notebook $nbName fStyle=1, text="Treatments:\r"
		Notebook $nbName ruler=ANOVADifTitles,fStyle=-1,
text="Treatment\tName\tSamples\tSum\tMean\tvariance\r"
		Notebook $nbName ruler=ANOVAmeanDifLine

		String wName
		String text
		Variable i, j
		for (i = 0; i < nTreatments; i += 1)
			wName = StringFromList(i, TreatmentWaves)
			Wave/Z w = $(wName)
			if (!WaveExists(w))
				break
			endif
			wName = NameOfWave(w)
			sprintf text, "%d\t%s\t%d\t%6.4g\t%6.4g\t%6.4g\r", i, wName,
numSamples[i], sumX[i], meanX[i], variance[i]
			Notebook $nbName fStyle=-1, text=text
		endfor
		Notebook $nbName text="\r"

		NVAR/Z LeveneHOVP
		NVAR/Z LeveneHOVF
```

```
	if (NVAR_Exists(LeveneHOVP))
		Notebook $nbName fStyle=1, text="Homogeneity of Variance:\r"
		String PLevel
		sprintf PLevel, "Levene's test F = %.4g,  P = %.4g\r", LeveneHOVF,
LeveneHOVP
		Notebook $nbName fStyle=-1, text=PLevel
		if (LeveneHOVP < 0.01)
			Notebook $nbName fStyle=-1, text="Levene's test indicates variability
in variance at better than 1% significance level.\r"
			Notebook $nbName fStyle=-1, text="View pairwise comparisons with
caution.\r"
		elseif (LeveneHOVP < 0.05)
			Notebook $nbName fStyle=-1, text="Levene's test indicates variability
in variance at better than 5% significance level.\r"
			Notebook $nbName fStyle=-1, text="View pairwise comparisons with
caution.\r"
		else
			Notebook $nbName fStyle=-1, text="Levene's test indicates
homogeneous variance at better than 1% significance level.\r"
		endif
	endif

	Notebook $nbName text="\r"
	Notebook $nbName fStyle=1, text="Analysis of Variance:\r"
	Notebook $nbName ruler=ANOVAReportLine, fStyle=-1, text="\tDF\tSum
Squares\tMean Square\tF\tprob\r"

	sprintf text, "Between Treatments\t%d\t%8.4g\t%8.4g\t%8.4g\t%8.3g\r",
DFBetweenTreatments, sumSqBetweenTreatments, meanSqBetweenTreatments, Fvalue,
probF
	Notebook $nbName text=text
	sprintf text, "Within Treatments\t%d\t%8.4g\t%8.4g\r", DFWithinTreatments,
sumSqWithinTreatments, meanSqWithinTreatments
	Notebook $nbName text=text
	Notebook $nbName text="\r"
	sprintf text, "Total\t%d\t%8.4g\t%8.4g\r",
DFBetweenTreatments+DFWithinTreatments,
sumSqBetweenTreatments+sumSqWithinTreatments,
meanSqBetweenTreatments+meanSqWithinTreatments
	Notebook $nbName text=text
	Notebook $nbName text="\r"

	Duplicate/O numSamples, numSamplesSquared
	numSamplesSquared = numSamples^2
	Variable totalSamples = sum(numSamples, -inf, inf)
	Variable reducedAveSamples = (totalSamples-sum(numSamplesSquared,-inf,
inf)/totalSamples)/(nTreatments-1)

	sprintf text, "Total samples = %d\r", totalSamples
	Notebook $nbName text=text
```

```
	sprintf text, "Sample mean = %6.4g\r", sum(sumX, -inf, inf)/totalSamples
	Notebook $nbName text=text
	sprintf text, "Variance of sample mean = %6.4g\r",
meanSqBetweenTreatments/totalSamples
	Notebook $nbName text=text
	sprintf text, "Treatment component of variance = %6.4g\r",
(meanSqBetweenTreatments-meanSqWithinTreatments)/reducedAveSamples
	Notebook $nbName text=text

	Notebook $nbName text="\r"

	String saveDF = GetDatafolder(1)
	if (DatafolderExists("TukeyHSD") || DatafolderExists("NewmanKeuls") ||
DatafolderExists("Dunnett"))
		Notebook $nbName fStyle=1, text="Pairwise treatment differences:\r"
		if (DatafolderExists("TukeyHSD"))
			SetDatafolder TukeyHSD

			Notebook $nbName fStyle=-1, text="Tukey HSD test:\r"
			Notebook $nbName ruler=ANOVADifTitles,
text="Treatments\tDifference of Means\tStandard Error\tSpan\tSRT\tprob\r"
			Notebook $nbName ruler=ANOVAmeanDifLine

			Wave MeanDif, Qstat, PairProb, MeanDifSE
			for (i = 0; i < nTreatments-1; i += 1)
				for (j = i+1; j < nTreatments; j += 1)
					Notebook $nbName fStyle=-1
					sprintf text, "%d,
%d\t%6.4g\t%6.4g\t%d\t%6.4g\t%6.4g", i,j, MeanDif[i][j], MeanDifSE[i][j], nTreatments,
Qstat[i][j], PairProb[i][j]
					Notebook $nbName text=text
					if (PairProb[i][j] < 0.05)
						Notebook $nbName text="*"
					endif
					if (PairProb[i][j] < 0.01)
						Notebook $nbName text="*"
					endif
					Notebook $nbName text="\r"
				endfor
			endfor

			SetDatafolder $saveDF
		endif

		if (DatafolderExists("NewmanKeuls"))
			SetDatafolder NewmanKeuls

			Notebook $nbName fStyle=-1, text="Newman Keuls test:\r"
			Notebook $nbName ruler=ANOVADifTitles,
text="Treatments\tDifference of Means\tStandard Error\tSpan\tSRT\tprob\r"
```

```
				Notebook $nbName ruler=ANOVAmeanDifLine

				Wave MeanDif, Qstat, PairProb, MeanDifSE, meanSortIndex
				Wave sig01,sig05
				Variable ii,jj,treatmentsSpanned

				for (ii = 0; ii < nTreatments; ii += 1)
					i = meanSortIndex[ii]
					for (jj = nTreatments-1; jj > ii; jj -= 1)
						j = meanSortIndex[jj]
						treatmentsSpanned = jj-ii+1
						Notebook $nbName fStyle=-1
						sprintf text, "%d,
%d\t%6.4g\t%6.4g\t%d\t%6.4g\t%6.4g", i,j, MeanDif[i][j], MeanDifSE[i][j],
treatmentsSpanned, Qstat[i][j], PairProb[i][j]
						Notebook $nbName text=text
						if (sig05[i][j])
							Notebook $nbName text="*"
						endif
						if (sig01[i][j])
							Notebook $nbName text="*"
						endif
						Notebook $nbName text="\r"
					endfor
				endfor

				SetDatafolder $saveDF
			endif

			if (DatafolderExists("Dunnett"))
				SetDatafolder Dunnett

				NVAR ControlItem
				Wave MeanDif, Qstat, PairProb, MeanDifSE
				Notebook $nbName fStyle=-1, text="Dunnett's test:\r"
				Notebook $nbName fStyle=-1, text="Control is treatment
"+num2istr(ControlItem)+"\r"
				Notebook $nbName ruler=ANOVADifTitles,
text="Treatments\tDifference of Means\tStandard Error\tt\tprob\r"
				Notebook $nbName ruler=ANOVAmeanDifLine

				for (i = 0; i < nTreatments; i += 1)
					if (i == ControlItem)
						continue
					endif
					sprintf text, "%d, %d\t%6.4g\t%6.4g\t%6.4g\t%6.4g",
i,ControlItem, MeanDif[i], MeanDifSE[i], Qstat[i], PairProb[i]
					Notebook $nbName text=text
					if (PairProb[i] < 0.05)
						Notebook $nbName text="*"
```

```
				endif
				if (PairProb[i] < 0.01)
					Notebook $nbName text="*"
				endif
				Notebook $nbName text="\r"
			endfor
		endif

		Notebook $nbName text="* = significant at 5% level; ** = significant at 1% 
level\r\r\r"
	endif

//	Notebook $nbName selection={startOfFile, startOfFile}
//	Notebook $nbName text=""
	DoWindow/F $nbName
end

static Function ANOVAReportSetup(nb)
	String nb

	Notebook $nb defaultTab=36, statusWidth=238, pageMargins={72,72,72,72}
	Notebook $nb showRuler=0, rulerUnits=1, updating={1, 60}
	Notebook $nb newRuler=Normal, justification=0, margins={0,0,468}, 
spacing={0,0,0}, tabs={}, rulerDefaults={"Geneva",10,0,(0,0,0)}
	Notebook $nb newRuler=ANOVATitle, justification=1, margins={0,0,468}, 
spacing={0,0,0}, tabs={}, rulerDefaults={"Geneva",14,1,(0,0,0)}
	Notebook $nb newRuler=ANOVAReportLine, justification=0, margins={0,0,468}, 
spacing={0,0,0}, tabs={135+1*8192,199+1*8192,278+1*8192,351+1*8192,414+1*8192}, 
rulerDefaults={"Geneva",10,0,(0,0,0)}
	Notebook $nb newRuler=ANOVADifTitles, justification=0, margins={0,0,468}, 
spacing={0,0,0}, tabs={99,217,306,360,405}, rulerDefaults={"Geneva",10,0,(0,0,0)}
	Notebook $nb newRuler=ANOVAmeanDifLine, justification=0, margins={0,0,486}, 
spacing={0,0,0}, tabs={117+3*8192,225+3*8192,297,360+3*8192,414+3*8192}, 
rulerDefaults={"Geneva",10,0,(0,0,0)}
end


//****************************
// some utility functions
//****************************

Function/S ListWavesFromWindowFullPath(WindowName)
	String WindowName

	if (strlen(WindowName) == 0)
		WindowName = WinName(0,3)
	endif
	if (strlen(WindowName) == 0)
		return ""
	endif
```

```
	String theList=""
	Variable i=0
	do
		Wave/Z w = WaveRefIndexed(WindowName, i, 1)
		if (!WaveExists(w))
			break
		endif
		theList += GetWavesDatafolder(w, 2)+";"
		i += 1
	while (1)

	if (strlen(theList) == 0)
		return "_None_"
	endif
	return theList
end

Function/S ListWavesFromBaseNameFullPath(BaseName)
	String BaseName			// expected to have one or more *'s in it: a string suitable
for use in WaveList

	String NameOnlyList = WaveList(BaseName, ";", "")
	String returnList = ""
	String OneWaveName
	Variable i, lasti = ItemsInList(NameOnlyList)
	for (i = 0; i < lasti; i += 1)
		OneWaveName = StringFromList(i, NameOnlyList)
		Wave/Z w = $OneWaveName
		if (WaveExists(w))
			returnList += GetWavesDatafolder(w, 2)+";"
		endif
	endfor

	return returnList
end

Function/S ListWavesFromWindowNameOnly(WindowName)
	String WindowName

	if (strlen(WindowName) == 0)
		WindowName = WinName(0,3)
	endif
	if (strlen(WindowName) == 0)
		return ""
	endif

	String theList=""
	Variable i=0
	do
```

```
		Wave/Z w = WaveRefIndexed(WindowName, i, 1)
		if (!WaveExists(w))
			break
		endif
		theList += NameOfWave(w)+";"
		i += 1
	while (1)

	if (strlen(theList) == 0)
		return "_None_"
	endif
	return theList
end



//**********************
// Utility functions
//**********************

// probability that an F value as high as F might happen by chance.
Function Fprob(F, num, denom)
	Variable F, num, denom

	Variable alpha = denom/(denom+num*F)

	return betai(denom/2, num/2, alpha)
end

// F value corresponding to a probility prob
Function Ff(prob, num, denom)
	Variable prob, num, denom

	Variable high = 1000
	Variable Low = 0
	Variable FHigh = Fprob(high, num, denom)
	Variable FLow = Fprob(low, num, denom)
	Variable Next, FNext
	do
		Next = (High+Low)/2
		FNext = Fprob(Next, num, denom)
		if (FNext < prob)
			High = Next
		else
			Low = Next
		endif
	while (abs(High-Low) > .001)
	return Next
end
```

```
//************************
// One-way ANOVA Control Panel
//************************

Function fANOVAOneWayPanel()

	String fmt="NewPanel/K=1/W=(%s) as \"One-way ANOVA\""
	Execute
WC_WindowCoordinatesSprintf("ANOVAOneWayPanel",fmt,20,43,390,301,1)	// pixels
	DoWindow/C ANOVAOneWayPanel

	NVAR/Z WaveSourceMenuMode =
root:Packages:WM_ANOVA:WaveSourceMenuMode
	Variable WaveSourceMode
	if (NVAR_Exists(WaveSourceMenuMode))
		WaveSourceMode = WaveSourceMenuMode
	else
		WaveSourceMode = 1
	endif
	PopupMenu
ANOVAWaveSourceMenu,pos={9,20},size={165,20},proc=ANOVAWaveSourceMenuProc,
title="Waves From"
	PopupMenu ANOVAWaveSourceMenu,mode=WaveSourceMode,value= #"\"Target
Window;Table;Graph;Base Name\""

	PopupMenu ANOVAWinMenu,pos={195,20},size={163,20},title="Which Table?"
	PopupMenu ANOVAWinMenu,mode=1,bodyWidth= 90,value= #"\"Top
Table;\"+WinList(\"*\", \";\", \"WIN:2\")",proc=ANOVAWindowMenuProc

	CheckBox ANOVATukeyHSDCheck,pos={71,110},size={70,14},title="Tukey
HSD",variable=root:Packages:WM_ANOVA:TukeyHSDChecked

	CheckBox ANOVAsnkCheck,pos={71,133},size={92,14},title="Newman-
Keuls",variable=root:Packages:WM_ANOVA:SNKChecked

	CheckBox ANOVADunnettCheck,pos={71,157},size={215,14},title="Dunnett's Test
(Compare with a control)"
	CheckBox
ANOVADunnettCheck,variable=root:Packages:WM_ANOVA:DunnetsChecked,
proc=DunnettsCheckProc

	CheckBox ANOVA_LevenesTestCheck,pos={44,57},size={210,14},title="Levene's
Test of Homogeneity of Variance"
	CheckBox
ANOVA_LevenesTestCheck,variable=root:Packages:WM_ANOVA:LeveneChecked

	GroupBox ANOVA_PairWiseBox,pos={45,90},size={276,117},title="Paired
Comparisons"
```

```
	PopupMenu ANOVA_ControlMenu,pos={125,176},size={144,20},title="Control is",proc=ANOVA_ControlMenuProc
	PopupMenu ANOVA_ControlMenu,mode=1,bodyWidth= 90,value= #"WaveList(\"*\",\";\",\"\")"

	SetVariable ANOVASetBaseName,pos={211,22},size={148,15},title="Base Name:",proc=ANOVASetBaseNameProc
	SetVariable ANOVASetBaseName,limits={-Inf,Inf,1},value= root:Packages:WM_ANOVA:WaveBaseName,bodyWidth= 90

	SetVariable ANOVAShowTarget pos={211,22},size={140,15},title="Target:"
	SetVariable ANOVAShowTarget value= root:Packages:WM_ANOVA:TargetWindow, noedit=1,frame=0

	Button ANOVADoItButton,pos={12,223},size={80,20},title="Do It",proc=ANOVADoItButtonProc

	Button ANOVACancelButton,pos={200,223},size={60,20},title="Cancel",proc=ANOVACancelButtonButtonProc

	Button ANOVAHelpButton,pos={280,223},size={80,20},title="Help", proc = OneWayANOVAHelp

	ControlInfo/W=ANOVAOneWayPanel ANOVAWaveSourceMenu
	ANOVAWaveSourceMenuProc("",V_value, S_value)
	ControlInfo/W=ANOVAOneWayPanel ANOVADunnettCheck
	DunnettsCheckProc("",V_value)

	SetWindow ANOVAOneWayPanel,hook=ANOVAOneWayPanelHookFunction
End

Function OneWayANOVAHelp(ctrlName) : ButtonControl
	String ctrlName

	Button ANOVAHelpButton, title="Looking..."
	DisplayHelpTopic/K=1 "One-way ANOVA"
	Button ANOVAHelpButton, title="Help"
end

Function ANOVAOneWayPanelHookFunction(infoStr)
	String infoStr

	String EventType = StringByKey("EVENT", infoStr)
	if (WC_WindowCoordinatesHook(infoStr))
		return 1
	endif
	if (CmpStr(EventType, "activate") == 0)
		SVAR/Z TargetWindow = root:Packages:WM_ANOVA:TargetWindow
		if (!SVAR_Exists(TargetWindow))
```

```
			String/G root:Packages:WM_ANOVA:TargetWindow
			SVAR/Z TargetWindow = root:Packages:WM_ANOVA:TargetWindow
		endif
		if (CmpStr(TargetWindow, WinName(0,3)) != 0)// target window has changed
			TargetWindow = WinName(0,3)
			ControlInfo/W=ANOVAOneWayPanel ANOVAWaveSourceMenu
			if (CmpStr("Target Window", S_value) == 0)
				ANOVAWaveSourceMenuProc("",V_value, S_value)
			endif
		endif
	endif
end

// From HR, Smoothing Control Panel.ipf
//	SetPopupToMatch(panelName, popupName, popupContentsList, matchText)
//	Selects the string value of the popup menu to matchText and returns the menu item
number.
//	If matchText is not a valid menu item, returns 0.
//	popupContentsList is the contents of the menu as a semicolon-separated list.
static Function SetPopupToMatch(panelName, popupName, popupContentsList,
matchText)
	String panelName
	String popupName
	String popupContentsList
	String matchText

	Variable menuItemNumber
	menuItemNumber = WhichListItem(matchText, popupContentsList) + 1
	if (menuItemNumber <= 0)
		return 0
	endif
	PopupMenu $popupName, win=$panelName, mode=menuItemNumber
	return menuItemNumber
End

Function SaveControlWaveForWindow(windowName, theWaveName)
	String windowName
	String theWaveName

	SVAR/Z
ControlWaveForWindow=root:Packages:WM_ANOVA:ControlWaveForWindow
	if (!SVAR_Exists(ControlWaveForWindow))
		String/G root:Packages:WM_ANOVA:ControlWaveForWindow = ""
		SVAR/Z
ControlWaveForWindow=root:Packages:WM_ANOVA:ControlWaveForWindow
	endif
	ControlWaveForWindow = ReplaceStringByKey(windowName,
ControlWaveForWindow, theWaveName)
end
```

```
Function/S GetControlWaveForWindow(windowName)
	String windowName

	SVAR/Z
ControlWaveForWindow=root:Packages:WM_ANOVA:ControlWaveForWindow
	if (!SVAR_Exists(ControlWaveForWindow))
		return ""
	else
		return StringByKey(windowName, ControlWaveForWindow)
	endif
end

Function ANOVAWaveSourceMenuProc(ctrlName,popNum,popStr)
	String ctrlName
	Variable popNum	// 1=Table, 2=Graph, 3=Base name
	String popStr

	String selectedWindow=""
	Variable ControlWaveMenuSet = 0
	String ConrolMenuList=""
	String WindowMenuContents=""

	strswitch(popStr)
		case "Target Window":
		case "Table":
		case "Graph":
			SetVariable ANOVASetBaseName,disable=1

			strswitch (popStr)
				case "Target Window":
					SetVariable ANOVAShowTarget,disable=0
					PopupMenu ANOVAWinMenu, disable=1
					String/G root:Packages:WM_ANOVA:TargetWindow
					SVAR/Z TargetWindow =
root:Packages:WM_ANOVA:TargetWindow
					TargetWindow = WinName(0,3)
					selectedWindow = TargetWindow
					break
				case "Table":
					SetVariable ANOVAShowTarget,disable=1
					PopupMenu ANOVAWinMenu, disable=0, title="Which
Table?"
					PopupMenu ANOVAWinMenu, value=#"WinList(\"*\",
\";\",\"WIN:2\")"
					WindowMenuContents = WinList("*", ";","WIN:2")
					SVAR/Z SelectedTable =
root:Packages:WM_ANOVA:SelectedTable
					if (!SVAR_Exists(SelectedTable))
						String/G
root:Packages:WM_ANOVA:SelectedTable
```

```
					SVAR/Z SelectedTable = 
root:Packages:WM_ANOVA:SelectedTable
					SelectedTable = WinName(0,2)
				endif
				selectedWindow = SelectedTable
				break
			case "Graph":
				SetVariable ANOVAShowTarget,disable=1
				PopupMenu ANOVAWinMenu, disable=0, title="Which 
Graph?"
				PopupMenu ANOVAWinMenu, value=#"WinList(\"*\", 
\";\",\"WIN:1\")"
				WindowMenuContents = WinList("*", ";","WIN:1")
				SVAR/Z SelectedGraph = 
root:Packages:WM_ANOVA:SelectedGraph
				if (!SVAR_Exists(SelectedGraph))
					String/G 
root:Packages:WM_ANOVA:SelectedGraph
					SVAR/Z SelectedGraph = 
root:Packages:WM_ANOVA:SelectedGraph
					SelectedGraph = WinName(0,2)
				endif
				selectedWindow = SelectedGraph
				break
		endswitch

		if (strlen(selectedWindow) > 0)
			if (SetPopupToMatch("ANOVAOneWayPanel", 
"ANOVAWinMenu", WindowMenuContents, selectedWindow) == 0)
				PopupMenu ANOVAWinMenu, mode=1
			endif
		endif

		PopupMenu ANOVA_ControlMenu, 
value=ListWavesFromANOVAPanelWindow()
		String ControlWave = GetControlWaveForWindow(selectedWindow)
		if (strlen(ControlWave) > 0)
			ControlWaveMenuSet = 
SetPopupToMatch("ANOVAOneWayPanel", "ANOVA_ControlMenu", 
ListWavesFromANOVAPanelWindow(), ControlWave)
		endif
		if (ControlWaveMenuSet == 0)
			PopupMenu ANOVA_ControlMenu,mode=1
			ControlInfo ANOVA_ControlMenu
			ControlWave = S_value
		endif
		SaveControlWaveForWindow(selectedWindow, ControlWave)
		break
	case "Base Name":
		SetVariable ANOVASetBaseName,disable=0
```

```
				SetVariable ANOVAShowTarget,disable=1
				PopupMenu ANOVAWinMenu, disable=1
				PopupMenu ANOVAWinMenu, value=#"WinList(\"*\", \";\",\"WIN:1\")"
				PopupMenu ANOVA_ControlMenu,
value=ListWavesFromANOVAPanelBaseName()
				ConrolMenuList = ListWavesFromANOVAPanelBaseName()
				SVAR/Z ControlWaveForBaseName =
root:Packages:WM_ANOVA:ControlWaveForBaseName
				if (SVAR_Exists(ControlWaveForBaseName))
					ControlWaveMenuSet =
SetPopupToMatch("ANOVAOneWayPanel", "ANOVA_ControlMenu",
ListWavesFromANOVAPanelBaseName(), ControlWaveForBaseName)
				endif
				if (ControlWaveMenuSet == 0)
					PopupMenu ANOVA_ControlMenu,mode=1
					ControlInfo ANOVA_ControlMenu
					ControlWave = S_value
					String/G
root:Packages:WM_ANOVA:ControlWaveForBaseName
					SVAR ControlWaveForBaseName =
root:Packages:WM_ANOVA:ControlWaveForBaseName
					ControlWaveForBaseName = ControlWave
				endif
				break
			default:
				break
	endswitch
End

Function/S ListWavesFromANOVAPanelWindow()

	String theWindow=""

	ControlInfo/W=ANOVAOneWayPanel ANOVAWaveSourceMenu
	if (CmpStr(S_value, "Target Window") == 0)
		SVAR/Z TargetWindow = root:Packages:WM_ANOVA:TargetWindow
		if (SVAR_Exists(TargetWindow))
			theWindow = TargetWindow
		endif
	else
		ControlInfo/W=ANOVAOneWayPanel ANOVAWinMenu
		theWindow = S_value
	endif
	return ListWavesFromWindowNameOnly(theWindow)
end

Function/S ListWavesFromANOVAPanelBaseName()

	SVAR/Z BaseName = root:Packages:WM_ANOVA:WaveBaseName
	String theList
```

```
	if (SVAR_Exists(BaseName))
		theList = WaveList(BaseName, ";", "")
	else
		theList = "_None_"
	endif

	return theList
end

Function ANOVAWindowMenuProc(ctrlName,popNum,popStr) : PopupMenuControl
	String ctrlName
	Variable popNum	// 1=Table, 2=Graph, 3=Base name
	String popStr

	String ControlWave = GetControlWaveForWindow(popStr)
	Variable ControlWaveMenuWasSet = 0
	if (strlen(ControlWave) > 0)
		ControlWaveMenuWasSet = SetPopupToMatch("ANOVAOneWayPanel",
"ANOVA_ControlMenu", ListWavesFromANOVAPanelWindow(), ControlWave)
	endif
		if (ControlWaveMenuWasSet == 0)
			PopupMenu ANOVA_ControlMenu,mode=1
			ControlInfo ANOVA_ControlMenu
			ControlWave = S_value
		endif
	SaveControlWaveForWindow(popStr, ControlWave)

	ControlInfo ANOVAWaveSourceMenu
	if (CmpStr(S_value, "Table") == 0)
		String/G root:Packages:WM_ANOVA:SelectedTable
		SVAR SelectedTable = root:Packages:WM_ANOVA:SelectedTable
		SelectedTable = popStr
	elseif (CmpStr(S_value, "Graph") == 0)
		String/G root:Packages:WM_ANOVA:SelectedGraph
		SVAR SelectedGraph = root:Packages:WM_ANOVA:SelectedGraph
		SelectedGraph = popStr
	endif
end


Function DunnettsCheckProc(ctrlName,checked) : CheckBoxControl
	String ctrlName
	Variable checked

	PopupMenu ANOVA_ControlMenu,disable= checked ? 0 : 2
End

Function ANOVACancelButtonButtonProc(ctrlName) : ButtonControl
	String ctrlName
```

```
	DoWindow/K ANOVAOneWayPanel
End

Function ANOVADoItButtonProc(ctrlName) : ButtonControl
	String ctrlName

	NVAR/Z DunnetsChecked=root:Packages:WM_ANOVA:DunnetsChecked
	NVAR/Z LeveneChecked=root:Packages:WM_ANOVA:LeveneChecked
	NVAR/Z SNKChecked=root:Packages:WM_ANOVA:SNKChecked
	NVAR/Z TukeyHSDChecked=root:Packages:WM_ANOVA:TukeyHSDChecked
	if (!NVAR_Exists(DunnetsChecked) || !NVAR_Exists(LeveneChecked) ||
!NVAR_Exists(SNKChecked) || !NVAR_Exists(TukeyHSDChecked))
		DoAlert 0, "BUG: Data for ANOVA checkboxes is missing."
		return 0
	endif
	Variable ControlColumn = 0
	if (DunnetsChecked)
		ControlInfo ANOVA_ControlMenu
		ControlColumn = V_value-1
	endif

	String theWindow=""

	ControlInfo ANOVAWaveSourceMenu
	strswitch (S_Value)
		case "Table":
		case "Graph":
			ControlInfo ANOVAWinMenu
			theWindow = S_Value
		// DROP THROUGH
		case "Target Window":
			Do1wayANOVAOnWindow(theWindow, TukeyHSDChecked,
SNKChecked, DunnetsChecked, ControlColumn, LeveneChecked)
			break
		case "Base Name":
			SVAR/Z WaveBaseName =
root:Packages:WM_ANOVA:WaveBaseName
			if (!SVAR_Exists(WaveBaseName))
				DoAlert 0, "BUG: Data for ANOVA is missing
(WaveBaseName)."
				return 0
			endif
			Do1wayANOVAOnBaseName(WaveBaseName, TukeyHSDChecked,
SNKChecked, DunnetsChecked, ControlColumn, LeveneChecked)
			break
		default:
			DoAlert 0, "BUG: ANOVA Wave source menu gives unknown
selection."
			break
	endswitch
```

```
End

Function ANOVASetBaseNameProc(ctrlName,varNum,varStr,varName) :
SetVariableControl
	String ctrlName
	Variable varNum
	String varStr
	String varName

	ControlUpdate ANOVA_ControlMenu
	PopupMenu ANOVA_ControlMenu, mode=1
End

Function ANOVA_ControlMenuProc(ctrlName,popNum,popStr) : PopupMenuControl
	String ctrlName
	Variable popNum
	String popStr

	String selectedWindow
	ControlInfo ANOVAWaveSourceMenu
	strswitch (S_value)
		case "Target Window":
			selectedWindow = WinName(0,3)
			break
		case "Table":
		case "Graph":
			ControlInfo ANOVAWinMenu
			selectedWindow = S_value
			break
	endswitch
	SaveControlWaveForWindow(selectedWindow, popStr)
End
//**********************
// Two-way ANOVA
//**********************

Function TwoWayANOVAWrapper(dataWave, transformation)
	Wave/Z dataWave
	Variable transformation

	if (!WaveExists(dataWave))
		DoAlert 0, "The data wave ("+NameOfWave(datawave)+") for two-way
factorial ANOVA does not exist."
		return -1
	endif
	String BaseName = NameOfWave(dataWave)
	String saveDF = GetDatafolder(1)
	NewDatafolder/O/S $(BaseName+"ANOVA2WF")
```

```
	Variable err=0
	if (WaveDims(dataWave) == 2)
		err = DoTwoWayANOVARandomizedBlocks(dataWave, transformation)
	elseif (WaveDims(dataWave) == 3)
		err = DoTwoWayANOVAFactorial(dataWave, transformation)
	else
		DoAlert 0,"Data wave for two-way ANOVA must be either 2D or 3D"
		err = 1
	endif
	if (!err)
		DoTwoWayANOVAReport(dataWave)
	endif

	SetDatafolder $saveDF
end

Function DoTwoWayANOVARandomizedBlocks(dataWave, transformation)
	Wave dataWave			// a JxK matrix wave containing observationa. Each row
is a block, each column a treatment
	Variable transformation		// 0: none; 1: square root; 2: log; 3: reciprocal
square root; 4: reciprocal; 5: arcsin sqrt

	Variable i,j,k
	Variable dataDims = WaveDims(dataWave)
	if (dataDims != 2)
		DoAlert 0, "Data for two-way ANOVA for randomized block design must be a
2D matrix wave."
		return -1
	endif

	Variable nRows = DimSize(dataWave, 0)
	Variable nColumns = DimSize(dataWave, 1)

	if (nRows < 2)
		DoAlert 0, "Data wave for two-way ANOVA for randomized block design
must have at least 2 rows."
		return -1
	endif
	if (nColumns < 2)
		DoAlert 0, "Data wave for two-way ANOVA for randomized block design
must have at least 2 columns."
		return -1
	endif

	if (transformation)
		Duplicate/O dataWave, transformedData
		 if (doTransformation(dataWave, transformation, transformedData))
			DoAlert 0, "The data contain values that could not be tranformed by
the chosen transformation."
			return -1
```

```
			endif
			Wave dataWave = transformedData
		endif

		Variable/G TransformationUsed = transformation

		Make/D/O/N=(nRows) ANOVA_Temp_Rows, RowTotal, RowAverage
		Make/D/O/N=(nColumns) ANOVA_Temp_Columns, ColumnTotal, ColumnAverage
		Duplicate/O dataWave, dataSquares
		dataSquares = dataWave^2

		Variable/G RowSS = 0
		for (i = 0; i < nRows; i += 1)
			ANOVA_Temp_Columns = dataWave[i][p]
			RowTotal[i] = sum(ANOVA_Temp_Columns, -inf, inf)
			RowSS += RowTotal[i]^2
		endfor
		Variable/G ColumnSS = 0
		RowAverage = RowTotal/nColumns
		for (i = 0; i < nColumns; i += 1)
			ANOVA_Temp_Rows = dataWave[p][i]
			ColumnTotal[i] = sum(ANOVA_Temp_Rows, -inf, inf)
			ColumnSS += ColumnTotal[i]^2
		endfor
		ColumnAverage = ColumnTotal/nRows
		Variable/G GrandTotal = sum(ColumnTotal, -inf, inf)
		Variable/G GrandAverage = GrandTotal/(nColumns*nRows)
		Variable/G Correction = GrandTotal^2/(nColumns*nRows)
		Variable/G TotalSS = sum(dataSquares, -inf, inf) - Correction
		RowSS = RowSS/nColumns - Correction
		ColumnSS = ColumnSS/nRows - Correction
		Variable/G ResidualSS = TotalSS - RowSS - ColumnSS
		Variable/G RowDF = nRows - 1
		Variable/G RowMS = RowSS/RowDF
		Variable/G ColumnDF = nColumns - 1
		Variable/G ColumnMS = ColumnSS/ColumnDF
		Variable/G ResidualDF = RowDF*ColumnDF
		Variable/G ResidualMS = ResidualSS/ResidualDF
		Variable/G TotalDF = RowDF+ColumnDF+ResidualDF
		Variable/G TotalMS = TotalSS/TotalDF
		Variable/G RowResidualFValue = RowMS/ResidualMS
		Variable/G ColumnResidualFValue = ColumnMS/ResidualMS
		Variable/G RowResidualFProb = Fprob(RowResidualFValue, RowDF, ResidualDF)
		Variable/G ColumnResidualFProb = Fprob(ColumnResidualFValue, ColumnDF,
ResidualDF)

		// Make residual and predicted data sets
		Duplicate/O dataWave, Residuals
		Duplicate/O dataWave, PredictedValues
		PredictedValues = RowAverage[p] + ColumnAverage[q] - GrandAverage
```

```
	Residuals = dataWave - PredictedValues

	if (transformation)
		unTransform(ColumnAverage, transformation, ColumnAverage)
		unTransform(RowAverage, transformation, RowAverage)
	endif

	return 0
end

CONSTANT noTransform=0
CONSTANT sqrtTransform=1
CONSTANT logTransform=2
CONSTANT recipSqrtTransform=3
CONSTANT reciprocalTransform=4
CONSTANT arcsinSqrtTransform=5

Function doTransformation(dataWave, transform, transformedData)
	Wave dataWave
	Variable transform
	Wave transformedData

	WaveStats/Q dataWave
	switch(transform)
		case noTransform:
			transformedData = dataWave			// shouldn't call this
function in this case; included for completeness
			break
		case sqrtTransform:
			if (V_min < 0)
				return -1
			endif
			transformedData = sqrt(dataWave)
			break
		case logTransform:
			if (V_min <= 0)
				return -1
			endif
			transformedData = log(dataWave)
			break
		case recipSqrtTransform:
			if (V_min <= 0)
				return -1
			endif
			transformedData = 1/sqrt(dataWave)
			break
		case reciprocalTransform:
			if (V_min <= 0)
				return -1
			endif
```

```
				transformedData = 1/dataWave
				break
			case arcsinSqrtTransform:
				if ( (V_min < 0) || (V_max > 100) )
					return -1
				endif
//				transformedData = asin(sqrt(dataWave))
				transformedData = asin(sqrt(dataWave/100))*180/pi
				break
		endswitch
end

Function unTransform(dataWave, transform, transformedData)
		Wave dataWave
		Variable transform
		Wave transformedData

		switch(transform)
			case noTransform:
				transformedData = dataWave				// shouldn't call this
function in this case; included for completeness
				break
			case sqrtTransform:
				transformedData = dataWave*dataWave
				break
			case logTransform:
				transformedData = 10^(dataWave)
				break
			case recipSqrtTransform:
				transformedData = 1/(dataWave*dataWave)
				break
			case reciprocalTransform:
				transformedData = 1/dataWave
				break
			case arcsinSqrtTransform:
				transformedData = 100*(sin(dataWave*pi/180)^2)
				break
		endswitch
end

Function unTransformNumber(inValue, transform)
		Variable inValue
		Variable transform

		Variable transformedData
		switch(transform)
			case noTransform:
				transformedData = inValue			// shouldn't call this function
in this case; included for completeness
				break
```

```
		case sqrtTransform:
			transformedData = inValue*inValue
			break
		case logTransform:
			transformedData = 10^(inValue)
			break
		case recipSqrtTransform:
			transformedData = 1/(inValue*inValue)
			break
		case reciprocalTransform:
			transformedData = 1/inValue
			break
		case arcsinSqrtTransform:
			transformedData = 100*(sin(inValue*pi/180)^2)
			break
	endswitch

	return transformedData
end

// A useful web site: psychology 207B at SUNY Buffalo, taught by Erwin M. Segal
// Segal's page:
// http://www.acsu.buffalo.edu/~segal/
// Course syllabus:
// http://wings.buffalo.edu/soc-sci/psychology/courses/segal/207B/207SYLs99.html

Function DoTwoWayANOVAFactorial(dataWave, transformation)
	Wave dataWave		// a JxKxn matrix wave containing observations. Each
row and column is a treatment, each layer is a repeat
	Variable transformation		// 0: none; 1: square root; 2: log; 3: reciprocal
square root; 4: reciprocal; 5: arcsin sqrt

	Variable i,j,k
	Variable dataDims = WaveDims(dataWave)
	if (dataDims != 3)
		DoAlert 0, "Data for two-way ANOVA for a factorial design must be a 3D
wave."
		return -1
	endif

	Variable nRows = DimSize(dataWave, 0)
	Variable nColumns = DimSize(dataWave, 1)
	Variable nLayers = DimSize(dataWave, 2)

	if (nRows < 2)
		DoAlert 0, "Data for two-way ANOVA for a factorial design must have at least
2 rows."
		return -1
	endif
	if (nColumns < 2)
```

```
		DoAlert 0, "Data for two-way ANOVA for a factorial design must have at least 2 columns."
		return -1
	endif
	if (nLayers < 2)
		DoAlert 0, "Data for two-way ANOVA for a factorial design must have at least 2 layers. Your data could be analyzed as a randomized block design."
		return -1
	endif

	if (transformation)
		Duplicate/O dataWave, transformedData
		 if (doTransformation(dataWave, transformation, transformedData))
			DoAlert 0, "The data contain values that could not be tranformed by the chosen transformation."
			return -1
		 endif
		 Wave dataWave = transformedData
	endif

	Make/D/O/N=(nRows, nColumns) LayerAverages, LayerSums, LayerSquaresSummed, LayerSquaresSummedBy_n

	// Within cells computations
	LayerSums = 0
	LayerSquaresSummed = 0
	for (i = 0; i < nLayers; i += 1)
		LayerSums += dataWave[p][q][i]
		LayerSquaresSummed += dataWave[p][q][i]^2
	endfor
	LayerAverages = LayerSums/nLayers
	LayerSquaresSummedBy_n = LayerSquaresSummed/nLayers
	Duplicate/O LayerSums, ANOVA_Temp
	ANOVA_Temp = LayerSums^2
	Variable WithinCellsSumSquare = sum(ANOVA_Temp, -inf, inf)/nLayers
		// Segal class notes (1)

	DoTwoWayANOVARandomizedBlocks(LayerAverages, 0)		// 0 means apply no transformation (it's already been done, if requested)

	Variable/G TransformationUsed = transformation

	NVAR RowSS, ColumnSS, ResidualSS, TotalSS
	NVAR RowMS, ColumnMS, ResidualMS, TotalMS
	NVAR RowDF, ColumnDF, ResidualDF, TotalDF
	NVAR GrandTotal

	RowSS *= nLayers
	ColumnSS *= nLayers
	ResidualSS *= nLayers
```

```
	RowMS *= nLayers
	ColumnMS *= nLayers
	ResidualMS *= nLayers
	GrandTotal *= nLayers

	Variable/G InteractionSS = ResidualSS
	Variable/G InteractionMS = ResidualMS
	Variable/G InteractionDF = ResidualDF
	KillVariables/Z ResidualSS,ResidualMS,ResidualDF

	Duplicate/O dataWave, ANOVA_Temp
	ANOVA_Temp = dataWave^2
	Variable SumDataSquares = sum(ANOVA_Temp, -inf, inf)
			// Segal class notes (4)
	Variable/G ErrorSS = SumDataSquares - WithinCellsSumSquare
	Variable/G TotalSS = SumDataSquares -
GrandTotal^2/(nRows*nColumns*nLayers)

	Variable/G ErrorDF = nRows*nColumns*(nLayers-1)
	Variable/G TotalDF = nRows*nColumns*nLayers -1		// yes- it's correct to
not have parens around nLayers-1

	Variable/G ErrorMS = ErrorSS/ErrorDF
	Variable/G TotalMS = TotalSS/TotalDF

	// F statistics
	Variable/G RowFValue = RowMS/ErrorMS
	Variable/G ColumnFValue = ColumnMS/ErrorMS
	Variable/G InteractionFValue = InteractionMS/ErrorMS

	// F probabilities
	Variable/G RowFProb = Fprob(RowFValue, RowDF, ErrorDF)
	Variable/G ColumnFProb = Fprob(ColumnFValue, ColumnDF, ErrorDF)
	Variable/G InteractionFProb = Fprob(InteractionFValue, InteractionDF, ErrorDF)

	NVAR GrandAverage
	Wave PredictedValues
	Wave Residuals, RowAverage, ColumnAverage
	Duplicate/O DataWave, FactResiduals
	Duplicate/O RowAverage, RowEffects
	RowEffects = RowAverage - GrandAverage
	Duplicate/O ColumnAverage, ColumnEffects
	ColumnEffects = ColumnAverage - GrandAverage
	Duplicate/O PredictedValues, PredictedValuesNoInteraction
	Duplicate/O PredictedValues, InteractionEffects
	InteractionEffects = LayerAverages - RowAverage[p] - ColumnAverage[q] +
GrandAverage
	PredictedValues = GrandAverage + RowEffects[p] + ColumnEffects[q] +
InteractionEffects[p][q]
	FactResiduals = DataWave - PredictedValues[p][q]
```

```
	Duplicate/O PredictedValues, ResidualsFromMeans
	ResidualsFromMeans = LayerAverages - PredictedValuesNoInteraction
	return 0
end

Function DoTwoWayANOVAReport(dataWave)
	Wave dataWave

//	SVAR AnalysisNotebookName = root:AnalysisNotebookName
	String nbName = "Scrap"
	if (WinType(nbName) != 0)
		// use the previous report notebook, but clear out all the text
	else
		// make a new notebook window
		NewNotebook/F=1/N=$nbName
	endif

	Variable i,j,k
	Variable nRows=DimSize(dataWave, 0)
	Variable nColumns=DimSize(dataWave, 1)
	Variable nLayers=DimSize(dataWave, 2)

	String CDimName = GetDimLabel(dataWave, 1, -1)
	String RDimName = GetDimLabel(dataWave, 0, -1)
	String dimName

	TwoWayFactorialReportSetup(nbName)

	Variable Transformation
	NVAR/Z TransformationUsed
	if (!NVAR_Exists(TransformationUsed))
		Transformation = 0
	else
		Transformation = TransformationUsed
	endif

	String text=""
	if (WaveDims(dataWave) == 3)
		text = "Two-way ANOVA, Factorial Design\r"
	else
		text = "Two-way ANOVA, Randomized Block Design\r"
	endif
	Notebook $nbName ruler=Normal, fSize=14, fStyle=1, text=text
	Notebook $nbName ruler=Normal, text="\r"
	Notebook $nbName ruler=Normal, text="Data wave: "
	Notebook $nbName fStyle=1, text=NameOfWave(dataWave)+"\r"
	string theFolder = GetWavesDatafolder(dataWave, 1)
	if (CmpStr(theFolder, "root:") != 0)
		Notebook $nbName fStyle=-1, text="Datafolder: "
		Notebook $nbName fStyle=1, text=theFolder+"\r"
```

```
	endif
	if (WaveDims(dataWave) > 2)
		Notebook $nbName fStyle=-1, text="Replications per cell: "
		Notebook $nbName fStyle=1, text=num2istr(nLayers)
	endif
	Notebook $nbName fStyle=-1, text="\r"
	if (Transformation)
		Notebook $nbName fStyle=-1,ruler=Normal, text="Transformation applied: "
		switch (transformation)
			case sqrtTransform:
				text = "square root: Y' = sqrt(Y)"
				break
			case logTransform:
				text = "log: Y' = log(Y)"
				break
			case recipSqrtTransform:
				text = "reciprocal square root: Y' = 1/sqrt(Y)"
				break
			case reciprocalTransform:
				text = "reciprocal: Y' = 1/Y"
				break
			case arcsinSqrtTransform:
				text = "arcsin square root: Y' = arcsin( sqrt(Y/100) )"
				break
		endswitch
		Notebook $nbName fStyle=1, text=text
		Notebook $nbName text="\r\r"
		Notebook $nbName ruler=Normal, fStyle=2, text="All numbers in this report
are based on transformed values, unless otherwise noted.\r"
		Notebook $nbName ruler=Normal, fStyle=-1
	endif
	Notebook $nbName ruler=Normal, text="\r"


	// Column averages
	Wave/Z ColumnAverage, ColumnEffects
	if (WaveExists(ColumnAverage))
		text = "Column "
		if (strlen(CDimName) > 0)
			text += "("+CDimName+") "
		endif
		text += "Averages\r"
		Notebook $nbName ruler=Normal, fStyle=1, text=text
		Notebook $nbName ruler=ANOVAReportLine, fStyle=-1
		text = "\tMean"
		if (WaveExists(ColumnEffects))
			text += "\tEffect"
		endif
		if (Transformation)
			text += "\tDe-transformed Mean"
```

```
		endif
		text += "\r"
		Notebook $nbName text = text
		for (i = 0; i < nColumns; i += 1)
			dimName = GetDimLabel(dataWave, 1, i)
			if (strlen(dimName) > 0)
				text = dimName
			else
				text = "Column "+num2istr(i)
			endif
			text += "\t"+num2str(ColumnAverage[i])
			if (WaveExists(ColumnEffects))
				text += "\t"+num2str(ColumnEffects[i])
			endif
			if (Transformation)
				text += "\t"+num2str(unTransformNumber(ColumnAverage[i],
Transformation))
			endif
			text += "\r"
			Notebook $nbName text=text
		endfor
		Notebook $nbName text="\r"
	endif

	// Row averages
	Wave/Z RowAverage, RowEffects
	if (WaveExists(RowAverage))
		text = "Row "
		if (strlen(RDimName) > 0)
			text += "("+RDimName+") "
		endif
		text += "Averages\r"
		Notebook $nbName ruler=Normal, fStyle=1, text=text

		Notebook $nbName ruler=ANOVAReportLine, fStyle=-1
		text = "\tMean"
		if (WaveExists(RowEffects))
			text += "\tEffect"
		endif
		if (Transformation)
			text += "\tDe-transformed Mean"
		endif
		text += "\r"
		Notebook $nbName text = text
		for (i = 0; i < nRows; i += 1)
			dimName = GetDimLabel(dataWave, 0, i)
			if (strlen(dimName) > 0)
				text = dimName
			else
				text = "Row "+num2istr(i)
```

```
				endif
				text += "\t"+num2str(RowAverage[i])
				if (WaveExists(RowEffects))
					text += "\t"+num2str(RowEffects[i])
				endif
				if (Transformation)
					text += "\t"+num2str(unTransformNumber(RowAverage[i],
Transformation))
				endif
				text += "\r"
				Notebook $nbName text=text
			endfor
			Notebook $nbName text="\r"
		endif

		NVAR/Z GrandAverage
		if (NVAR_Exists(GrandAverage))
			Notebook $nbName ruler=Normal, fStyle=1, text="Grand Average:\t"
			Notebook $nbName ruler=Normal, fStyle=-1,
text=num2str(GrandAverage)+"\r\r"
		endif

		//ANOVA itself, including main effects
		String stars=""
		NVAR/Z RowSS, ColumnSS,InteractionSS,ResidualSS,ErrorSS,TotalSS
		NVAR/Z RowDF, ColumnDF,InteractionDF,ResidualDF,ErrorDF,TotalDF
		NVAR/Z RowMS, ColumnMS,InteractionMS,ResidualMS,ErrorMS,TotalMS
		if (WaveDims(dataWave) > 2)
			NVAR/Z RowF = RowFValue
			NVAR/Z ColumnF = ColumnFValue
			NVAR/Z InteractionF = InteractionFValue
			NVAR/Z RowP = RowFProb
			NVAR/Z ColumnP = ColumnFProb
			NVAR/Z InteractionP = InteractionFProb
		else
			NVAR/Z RowF = RowResidualFValue
			NVAR/Z ColumnF = ColumnResidualFValue
			NVAR/Z RowP = RowResidualFProb
			NVAR/Z ColumnP = ColumnResidualFProb
		endif

		Notebook $nbName ruler=Normal, fStyle=1, text ="Analysis of Variance\r"
		Notebook $nbName ruler=ANOVATwoWayTitles, fStyle=-1,
text="Source\tSS\tDF\tMean Square\tF\tProb\r"

		if (strlen(CDimName) > 0)
			dimName = CDimName
		else
			dimName = "Columns"
		endif
```

```
	if (ColumnP < .01)
		stars = "**"
	elseif (ColumnP < .05)
		stars = "*"
	else
		stars = ""
	endif
	sprintf text,"%s\t%8.4g\t%d\t%8.4g\t%8.4g\t%8.4g%s\r", dimName, ColumnSS, 
ColumnDF, ColumnMS, ColumnF, ColumnP,stars
	Notebook $nbName ruler=ANOVATwoWayLine, text=text

	if (strlen(RDimName) > 0)
		dimName = RDimName
	else
		dimName = "Rows"
	endif
	if (RowP < .01)
		stars = "**"
	elseif (RowP < .05)
		stars = "*"
	else
		stars = ""
	endif
	sprintf text,"%s\t%8.4g\t%d\t%8.4g\t%8.4g\t%8.4g%s\r", dimName, RowSS, 
RowDF, RowMS, RowF, RowP,stars
	Notebook $nbName ruler=ANOVATwoWayLine, text=text

	String ComparisonLine=""
	if (NVAR_Exists(InteractionSS))
		if (InteractionP < .01)
			stars = "**"
		elseif (InteractionP < .05)
			stars = "*"
		else
			stars = ""
		endif
		sprintf text,"%s\t%8.4g\t%d\t%8.4g\t%8.4g\t%8.4g\r", "Interaction", 
InteractionSS, InteractionDF, InteractionMS, InteractionF, InteractionP
		Notebook $nbName ruler=ANOVATwoWayLine, text=text
		sprintf text,"%s\t%8.4g\t%d\t%8.4g\r", "Error", ErrorSS, ErrorDF, ErrorMS
		Notebook $nbName ruler=ANOVATwoWayLine, text=text
		ComparisonLine = "Error"
	else
		sprintf text,"%s\t%8.4g\t%d\t%8.4g\r", 
"Residual",ResidualSS,ResidualDF,ResidualMS
		Notebook $nbName ruler=ANOVATwoWayLine, text=text
		ComparisonLine = "Residual"
	endif

	Notebook $nbName  text="\r"
```

```
	sprintf text,"%s\t%8.4g\t%d\t%8.4g\r\r", "Total", TotalSS, TotalDF, TotalMS
	Notebook $nbName ruler=ANOVATwoWayLine, text=text

	Notebook $nbName  ruler=Normal, text="*\tsignificant at greater than 95% level.\r"

	Notebook $nbName  ruler=Normal, text="**\tsignificant at greater than 99% level.\r"

	Notebook $nbName ruler=Normal, text="F values and probabilities represent
comparison with the "+ComparisonLine+" line.\r\r"

//	Notebook $nbName selection={startOfFile, startOfFile}			// set selection
to top of window
//	Notebook $nbName text=""
	// force the notebook to display the selection by pretending to insert text
	DoWindow/F $nbName
end

Function TwoWayFactorialReportSetup(nb)
	String nb

	Notebook $nb defaultTab=36, statusWidth=238, pageMargins={72,72,72,72}
	Notebook $nb showRuler=0, rulerUnits=1, updating={1, 60}
	Notebook $nb newRuler=Normal, justification=0, margins={0,0,468},
spacing={0,0,0}, tabs={}, rulerDefaults={"Geneva",10,0,(0,0,0)}
	Notebook $nb newRuler=ANOVATitle, justification=1, margins={0,0,468},
spacing={0,0,0}, tabs={}, rulerDefaults={"Geneva",14,1,(0,0,0)}
	Notebook $nb newRuler=ANOVATwoWayTitles, justification=0, margins={0,0,468},
spacing={0,0,0}, tabs={90,171,216,306,360,405}, rulerDefaults={"Geneva",10,0,(0,0,0)}
	Notebook $nb newRuler=ANOVATwoWayLine, justification=0, margins={0,0,486},
spacing={0,0,0}, tabs={99+3*8192,172,235+3*8192,306+3*8192,370+3*8192},
rulerDefaults={"Geneva",10,0,(0,0,0)}
	Notebook $nb newRuler=ANOVAReportLine, justification=0, margins={0,0,468},
spacing={0,0,0}, tabs={135+1*8192,220+1*8192,320+1*8192,351+1*8192,414+1*8192},
rulerDefaults={"Geneva",10,0,(0,0,0)}
end

Function/S List2Dand3DWavesForANOVA()

	String theList = ""
	Variable i = 0
	do
		Wave/Z w = WaveRefIndexed("", i, 4)
		if (!WaveExists(w))
			break
		endif
		if ( (WaveDims(w) == 2) || (WaveDims(w) == 3) )
			if (DimSize(w, 0) >= 2)
				if (DimSize(w,1) >= 2)
					theList += NameOfWave(w)+";"
				endif
```

```
				endif
			endif
			i += 1
		while (1)

		if (strlen(theList) == 0)
			theList = "None Available"
		endif

		return theList
end

Function fTwoWayANOVAPanel()

		String fmt="NewPanel/K=1/W=(%s) as \"Two-way ANOVA\""
		Execute
WC_WindowCoordinatesSprintf("ANOVATwoWayPanel",fmt,14,58,379,251,1)	// pixels
		DoWindow/C ANOVATwoWayPanel

		PopupMenu ANOVATwoWayDataMenu,pos={19,24},size={189,20},title="Wave
containing data:"
		PopupMenu ANOVATwoWayDataMenu,mode=1,value=
#"List2Dand3DWavesForANOVA()"
		PopupMenu ANOVATwoWayDataMenu proc=ANOVATwoWayDataMenuProc

		PopupMenu
ANOVATwoWayTransformMenu,pos={19,62},size={176,20},title="Apply Transformation:"
		PopupMenu ANOVATwoWayTransformMenu,mode=1,value= #"\"None;Square
Root;Log;Reciprocal Square Root;Reciprocal;Arcsin Square Root\""

		Button
ANOVATwoWayDoItButton,pos={19,131},size={80,20},proc=ANOVATwoWayDoItButtonPr
oc,title="Do It"

		Button
ANOVATwoWayCancelButton,pos={210,131},size={60,20},proc=ANOVATwoWayCancelB
uttonProc,title="Cancel"

		Button
ANOVATwoWayHelpButton,pos={280,131},size={80,20},proc=TwoWayANOVAHelp,title="
Help"

		ControlInfo ANOVATwoWayDataMenu
		ANOVATwoWayDataMenuProc("",V_value,S_value)

		SetWindow ANOVATwoWayPanel,hook=ANOVATwoWayPanelHookFunction
End

Function TwoWayANOVAHelp(ctrlName) : ButtonControl
		String ctrlName
```

```
	Button ANOVATwoWayHelpButton, title="Looking..."
	DisplayHelpTopic/K=1 "Two-way ANOVA"
	Button ANOVATwoWayHelpButton, title="Help"
end

Function ANOVATwoWayPanelHookFunction(infoStr)
	String infoStr

	String EventType = StringByKey("EVENT", infoStr)
	if (WC_WindowCoordinatesHook(infoStr))
		return 1
	endif
	if (CmpStr(EventType, "activate") == 0)
		ControlUpdate /W=ANOVATwoWayPanel ANOVATwoWayDataMenu
		ControlInfo/W=ANOVATwoWayPanel ANOVATwoWayDataMenu
		ANOVATwoWayDataMenuProc("",V_value,S_value)
	endif
end

Function ANOVATwoWayDoItButtonProc(ctrlName) : ButtonControl
	String ctrlName

	ControlInfo ANOVATwoWayDataMenu
	Wave w = $S_value
	ControlInfo ANOVATwoWayTransformMenu
	Variable transform = V_value-1
	TwoWayANOVAWrapper(w, transform)
End

Function ANOVATwoWayCancelButtonProc(ctrlName) : ButtonControl
	String ctrlName

	DoWindow/K ANOVATwoWayPanel
End

Function ANOVATwoWayDataMenuProc(ctrlName,popNum,popStr) : PopupMenuControl
	String ctrlName
	Variable popNum
	String popStr

	Wave/Z w=$popStr
	if (WaveExists(w))
		Button ANOVATwoWayDoItButton,disable=0
	else
		Button ANOVATwoWayDoItButton,disable=2
	endif
	ANOVA2WAddTextToPanel(w)
End
```

```
Function ANOVA2WAddTextToPanel(w)
	Wave/Z w

	SetDrawLayer/K/W=ANOVATwoWayPanel ProgBack
	SetDrawLayer/W=ANOVATwoWayPanel ProgBack
	if (WaveExists(w))
		if (WaveDims(w) == 2)
			SetDrawEnv fsize= 10
			DrawText 21,106,"2D wave selected: will perform randomized
blocks analysis"
			SetDrawEnv fsize= 10
			DrawText 21,123,"(no interaction effects considered)"
		else
			SetDrawEnv fsize= 10
			DrawText 21,106,"3D wave selected: will perform factorial analysis"
			SetDrawEnv fsize= 10
			DrawText 21,123,"(interaction effects are considered)"
		endif
	else
		SetDrawEnv fsize= 10
		DrawText 21,106,"No 2D or 3D waves available in the current datafolder."
	endif
end



Function CalcRMAnova(w,g)
	wave/Z w,g  // w should be 2-dimensional: rows being subjects, columns being
trials; g is 1 dimensional containing group labels

	// checks
	variable NumGroups, NumSubjects, numTrials
	NumSubjects = Dimsize(g,0)
	wavestats /Q g
	NumGroups = V_max					// assuming group labels are 1,2,...3
; 0 is reserved for "not associated with group,ignore"
	NumTrials = Dimsize(w,1)
	make /O /N=(NumGroups+1) RMASubjectsPerGroup=0, RMAtmpG=0
	variable N=0,SumX=0, SumX2=0, C=0, SST=0, SSGroup=0, SSGroupE=0,
SSTrial=0, SSTrialGroup=0, SSTrialGroupE=0
	variable i=0, j=0,tmp
	do
		j=0
		do
			N+=(g[i]>0)*1
			SumX+=(g[i]>0)*w[i][j]
			SumX2+=(g[i]>0)*w[i][j]^2
			j+=1
		while(j<NumTrials)
		i+=1
```

```
	while(i<NumSubjects)
	C=SumX^2/N
	SST=SumX2-C
	i=0
	do
		j=0
		do
			RMAtmpG[g[i]]+=w[i][j]
			j+=1
		while(j<NumTrials)
		RMASubjectsPerGroup[g[i]]+=1
		i+=1
	while(i<NumSubjects)
	RMAtmpG=RMAtmpG^2/(RMASubjectsPerGroup*NumTrials) // each sqaure
weighed by the number of entries
	SSGroup=sum(RMAtmpG,1,inf)-C

	i=0
	do
		j=0
		tmp=0
		do
			tmp+=w[i][j]
			j+=1
		while(j<NumTrials)
		SSGroupE+=(g[i]>0)*tmp^2
		i+=1
	while(i<NumSubjects)
	SSGroupE=SSGroupE/NumTrials - C - SSGroup

	j=0
	do
		i=0
		tmp=0
		do
			tmp+=(g[i]>0)*w[i][j]
			i+=1
		while(i<NumSubjects)
		SSTrial+=tmp^2
		j+=1
	while(j<NumTrials)
	SSTrial=SSTrial/(NumSubjects-RMASubjectsPerGroup[0]) -C


	j=0
	do
		RMAtmpG=0
		i=0
		do
			RMAtmpG[g[i]]+=(g[i]>0)*w[i][j]
```

```
                i+=1
            while(i<NumSubjects)
            RMAtmpG=RMAtmpG^2
            RMAtmpG=RmAtmpG/RMASubjectsPerGroup
            SSTrialGroup+=sum(RMAtmpG,1,inf)
            j+=1
        while(j<NumTrials)
        SSTrialGroup=SSTrialGroup-C-SSGroup-SSTrial
        SSTrialGroupE=SST-SSGroup-SSGroupE-SSTrial-SSTrialGroup

        variable GroupDF, MSGroup, GroupDFE=0, MSGroupE, FGroup, pGroup
        GroupDF=NumGroups-1;MSGroup=SSGroup/(NumGroups-1)
        i=1
        do
            GroupDFE+=RMASubjectsPerGroup[i]-1
            i+=1
        while(i<NumGroups+1)
        MSGroupE=SSGroupE/GroupDFE
        FGroup=MSGroup/MSGroupE
        pGroup=    Fprob(FGroup, GroupDF, GroupDFE)
        print " DF Group : ", GroupDF, "MSGroup : ", MSGroup
        print " DFE Group: ", GroupDFE, "MSGroupE : ", MSGroupE
        print " F : ", FGroup, " ; p : ", pGroup

        variable TrialDF, MSTrial, TrialGroupDF, MSTrialGroup, TrialGroupDFE=0,
MSTrialGroupE, FTrial, pTrial, FTrialGroup, pTrialGroup
        TrialDF=NumTrials-1
        MSTrial=SSTrial/TrialDF
        TrialGroupDF=TrialDF*GroupDF
        MSTrialGroup=SSTrialGroup/TrialGroupDF
        i=1
        do
            TrialGroupDFE+=(RMASubjectsPerGroup[i]-1)*TrialDF
            i+=1
        while(i<NumGroups+1)
        MSTrialGroupE=SSTrialGroupE/TrialGroupDFE
        FTrial=MSTrial/MSTrialGroupE
        pTrial=Fprob(FTrial, TrialDF, TrialGroupDFE)
        print " DF Trial : ", TrialDF, "MSTrial : ", MSTrial
        print " F : ", FTrial, " ; p : ", pTrial
        FTrialGroup=MSTrialGroup/MSTrialGroupE
        pTrialGroup=Fprob(FTrialGroup, TrialGroupDF, TrialGroupDFE)
        print " DF Trial : ", TrialGroupDF, "MSTrial : ", MSTrialGroup
        print " DFE Trial: ", TrialGroupDFE, "MSTrialGroupE : ", MSTrialGroupE
        print " F : ", FTrialGroup, " ; p : ", pTrialGroup


print NumGroups
print N, SUmX, SumX2, C, SSGroup, SSGroupE, SSTrial, SSTrialGroup, SSTrialGroupE
```

```
	String nbName = "Scrap"
	if (WinType(nbName) != 0)
		// use the previous report notebook, but clear out all the text
	else
		// make a new notebook window
		NewNotebook/F=1/N=$nbName
	endif

	TwoWayFactorialReportSetup(nbName)
	String text=""
	text = "Two-way Repeated Measure ANOVA\r"
	Notebook $nbName ruler=Normal, fSize=14, fStyle=1, text=text
	Notebook $nbName ruler=Normal, text="\r"
	Notebook $nbName fStyle=-1, text="\r"
	Notebook $nbName ruler=Normal, text="\r"

	//ANOVA itself, including main effects
	String stars="",dimName

	Notebook $nbName ruler=Normal, fStyle=1, text ="Analysis of Variance\r"
	Notebook $nbName ruler=ANOVATwoWayTitles, fStyle=-1,
text="Source\tSS\tDF\tMean Square\tF\tProb\r"

	dimName = "Groups"
	if (pGroup < .01)
		stars = "**"
	elseif (pGroup < .05)
		stars = "*"
	else
		stars = ""
	endif
	sprintf text,"%s\t%8.4g\t%d\t%8.4g\t%8.4g\t%8.4g%s\r", dimName, SSGroup,
GroupDF, MSGroup, FGroup, PGroup,stars
	Notebook $nbName ruler=ANOVATwoWayLine, fSize=10, fStyle=0, text=text
	dimName = "Time"
	if (pTrial < .01)
		stars = "**"
	elseif (pTrial < .05)
		stars = "*"
	else
		stars = ""
	endif
	sprintf text,"%s\t%8.4g\t%d\t%8.4g\t%8.4g\t%8.4g%s\r", dimName, SSTrial,
TrialDF, MSTrial, FTrial, pTrial,stars
	Notebook $nbName ruler=ANOVATwoWayLine, fSize=10, fStyle=0, text=text

	String ComparisonLine=""
	if (pTrialGroup < .01)
		stars = "**"
	elseif (pTrialGroup < .05)
```

```
			stars = "*"
		else
			stars = ""
		endif
		sprintf text,"%s\t%8.4g\t%d\t%8.4g\t%8.4g\t%8.4g%s\r", "Interaction", 
SSTrialGroup, TrialGroupDF, MSTrialGroup, FTrialGroup, pTrialGroup, stars
		Notebook $nbName ruler=ANOVATwoWayLine, text=text
		sprintf text,"%s\t%8.4g\t%d\t%8.4g\r", "Error", SSTrialGroupE, TrialGroupDFE, 
MSTrialGRoupE
		Notebook $nbName ruler=ANOVATwoWayLine, text=text
		ComparisonLine = "Error"

		Notebook $nbName  text="\r"
		sprintf text,"%s\t%8.4g\t%d\t%8.4g\r\r", "Total", SST, 
TrialDF+GroupDF+GroupDFE+TrialGRoupDF+TrialGroupDFE, 
SST/(TrialDF+GroupDF+GroupDFE+TrialGRoupDF+TrialGroupDFE)
		Notebook $nbName ruler=ANOVATwoWayLine, text=text

		Notebook $nbName  ruler=Normal, text="*\tsignificant at greater than 95% level.\r"

		Notebook $nbName  ruler=Normal, text="**\tsignificant at greater than 99% level.\r"

		Notebook $nbName ruler=Normal, text="F values and probabilities represent 
comparison with the "+ComparisonLine+" line.\r\r"

		DoWindow/F $nbName

End
```

4 – Behavior experiment: training template

```
#pragma rtGlobals=1                    // Use modern global access method.

// B e f o r e   starting the program with STARTUP, save Igor Experiment under a new
name !!!!


//=========================Start Macros =========================
Macro Startup()
        Initialization()
        MainPanel()
        ValvePanel()
        DisplayPanel()
        Trials()
        Table0()
        Table1()
        Table3()
        // Initialize/test valves
        OpenValve(128*valve128+64*valve64+32*valve32+16*valve16+8*valve8+4*valve4
+2*valve2+valve1) // operate valves
        OperateWaterFinal(4*RF+1*FV) // operate valves
        sleep 00:00:01
        InitValves()
Endmacro
// ==========================================================

Macro Initialization()
        //String Variables Definition: SaveFileName controls the data destination | Whatsup
analises what is going on within the interaction animal-olfactometer
        string /g SaveFileName, WhatsUp,TrainingMode
        // Variables related with counting
        variable /g TrialNumber, BlockNumber,NumTrialsINBlock=20, FirstTrialINBlock
        variable /g ReinforcementTime=70, SamplePercent=0.22, ITIinSeconds=4
        // Variables related with time counting
        variable /g count, timerNum, timerNum2, timerNumAccurate, runTime, lickTime,
sampleTime, bbTmp, ilTmp, runTimeAccurate, count2
        // Variables related to the operating odor valves, Final Valve and Reinforcement
Valve
        variable /g valve1=1, valve2=1, valve4=1,  valve8=1, valve16=1, valve32=1,
valve64=1, valve128=1, FV=1, RF=1
        // Variables within the Training procedure: Control of Beam Break
        variable /g nBeam, ITICount, IsITI=0
        variable /g SPlusValve=2, SMinusValve=1, SPlus2nd=4, SPlus3rd=16,
SPlus4th=64, SMinus2nd=8, SMinus3rd=32,SMinus4th=128
        variable /g SPlusControlValve, SMinusControlValve, SPlusCount, SMinusCount
        variable /g lick, beam

        // Wave Specifications
        make /N=1000 /O OdorValve=0, OdorValveTemp=0, Reward=-1, Accepted=-1
```

```
	make /T /N=1000 /O TrialTime=""
	make /N=(1000,125) /O LickPattern, samplePattern
	make /O /N=125, lTmp, sTmp, ttmp
	make /N=50 /O
NumMaxCorrTM,NumMaxCorrTP,NumMaxCorrCM,NumMaxCorrCP
	make /T /O /N=(2,11) Responsewave=""
	make /O /N=300000 lickWaveTmp, sampleWaveTmp
	make /N=125 /O sampleAverage, lickAverage
	make /N=1000 /O averageMark
	make /N=20 /O dummyT1, dummyT2, dummyT3, dummyC1, dummyC2, dummyC3
	make /O/N=50 CorrSPlus=0, CorrSMin=0, CorrCP=0, CorrCM=0, CorrPercent=-1

	// PORT DEFINITIONS  => see Hardware section of macros
	// 773 = Beam
	// 772 = Lick
	// 768 = Odor valves
	// 770 = Final valve & water valve

	// Generate path for data storage
	savefilename = igorInfo(1)
	NewPath/O SaveData "C:Training_Logfiles:"

	// Make ValveResponse1..2..4..8..16..32..64..128_0/1
	variable c = 1
	do
		make /N=1000 /O
$("ValveResponse"+num2str(c)+"_0"),$("ValveResponse"+num2str(c)+"_1")
		// appendtograph
$("ValveResponse"+num2str(c)+"_0"),$("ValveResponse"+num2str(c)+"_1")
		$("ValveResponse"+num2str(c)+"_0")=-100
		$("ValveResponse"+num2str(c)+"_1")=-100
		c*=2
	while (c<255)

	// define OdorValve assignments for pseudorandomized session
	dummyT1(0)=1; dummyT1(1)=1; dummyT1(2)=0;dummyT1(3)=1; dummyT1(4)=0
	dummyT1(5)=0; dummyT1(6)=1; dummyT1(7)=0;dummyT1(8)=1; dummyT1(9)=0
	dummyT1(10)=1; dummyT1(11)=0; dummyT1(12)=0;dummyT1(13)=1;
dummyT1(14)=0
	dummyT1(15)=1; dummyT1(16)=1; dummyT1(17)=0;dummyT1(18)=1;
dummyT1(19)=0

	dummyT2(0)=1; dummyT2(1)=0; dummyT2(2)=0;dummyT2(3)=1; dummyT2(4)=0
	dummyT2(5)=1; dummyT2(6)=1; dummyT2(7)=0;dummyT2(8)=1; dummyT2(9)=0
	dummyT2(10)=1; dummyT2(11)=0; dummyT2(12)=1;dummyT2(13)=1;
dummyT2(14)=0
	dummyT2(15)=1; dummyT2(16)=0; dummyT2(17)=0;dummyT2(18)=1;
dummyT2(19)=0

	dummyT3(0)=1; dummyT3(1)=0; dummyT3(2)=1;dummyT3(3)=1; dummyT3(4)=0
```

```
        dummyT3(5)=1; dummyT3(6)=0; dummyT3(7)=0;dummyT3(8)=1; dummyT3(9)=0
        dummyT3(10)=1; dummyT3(11)=1; dummyT3(12)=0;dummyT3(13)=1;
dummyT3(14)=0
        dummyT3(15)=0; dummyT3(16)=1; dummyT3(17)=0;dummyT3(18)=1;
dummyT3(19)=0

        dummyC1(0)=1; dummyC1(1)=0; dummyC1(2)=2;dummyC1(3)=1; dummyC1(4)=0
        dummyC1(5)=0; dummyC1(6)=1; dummyC1(7)=3;dummyC1(8)=1; dummyC1(9)=0
        dummyC1(10)=1; dummyC1(11)=2; dummyC1(12)=1;dummyC1(13)=0;
dummyC1(14)=0
        dummyC1(15)=1; dummyC1(16)=3; dummyC1(17)=0;dummyC1(18)=1;
dummyC1(19)=0

        dummyC2(0)=1; dummyC2(1)=2; dummyC2(2)=1;dummyC2(3)=0; dummyC2(4)=0
        dummyC2(5)=1; dummyC2(6)=3; dummyC2(7)=0;dummyC2(8)=1; dummyC2(9)=0
        dummyC2(10)=1; dummyC2(11)=3; dummyC2(12)=0;dummyC2(13)=1;
dummyC2(14)=0
        dummyC2(15)=0; dummyC2(16)=1; dummyC2(17)=2;dummyC2(18)=0;
dummyC2(19)=1

        dummyC3(0)=1; dummyC3(1)=3; dummyC3(2)=0;dummyC3(3)=1; dummyC3(4)=0
        dummyC3(5)=0; dummyC3(6)=1; dummyC3(7)=2;dummyC3(8)=0; dummyC3(9)=1
        dummyC3(10)=1; dummyC3(11)=0; dummyC3(12)=2;dummyC3(13)=1;
dummyC3(14)=0
        dummyC3(15)=0; dummyC3(16)=1; dummyC3(17)=3;dummyC3(18)=1;
dummyC3(19)=0
Endmacro

//========================== Hardware ===============================

Function InPortB(portNo)
variable portNo
return ReadPort(portNo)
End

Function OutPortB(portNo, ByteVal)
variable portNo, ByteVal
WritePort(portNo, ByteVal)
End

FUNCTION BeamBroken()
   if (InPOrtB(773)==1)
        return 0
   else
        return 1
   endif
END

FUNCTION IsLicked()
  if (inPortB(772)==1)
```

```
        return 1
    else
        return 0
    endif
END

FUNCTION OpenValve(valveNo)
variable valveNo
  OutPortB(768,valveNo)
END

FUNCTION CloseValve(valveNo)
variable valveNo
  OutPortB(768,0) // allways shuts off all
END

FUNCTION OpenWaterValve()
  OutPortB(770,5)
END

FUNCTION CloseWaterValve()
  OutPortB(770,0)
END

FUNCTION OpenFinalValve()
  OutPortB(770,6)
END

FUNCTION CloseFinalValve()
  OutPortB(770,0)
END

FUNCTION OperateWaterFinal(valveNo)
variable valveNo
  OutPortB(770,valveNo)
END

FUNCTION InitValves()
        OutportB(772,0x9B)
        OutPortB(771,0x80)  // what ist port 771?
        OutPortB(768,0)
END

//
==================================================================
============

//
==================================================================
============
```

```
// Entry point to start training session for MEMORY
MACRO StartMemory()
	variable cc,dd
	TrainingMode = "Memory"
	cc=0
	do
		dd=0
		do
			OdorValveTemp[cc*20+dd+1]=$("dummyT"+num2str(cc+1))[dd]
			dd+=1
		while(dd<20)
		NumMaxCorrTM[cc+1]=10
		NumMaxCorrTP[cc+1]=10
		NumMaxCorrCM[cc+1]=0
		NumMaxCorrCP[cc+1]=0
		cc+=1
	while(cc<3)
	do
		dd=0
		do

	OdorValveTemp[cc*20+dd+1]=$("dummyC"+num2str(mod(cc,3)+1))[dd]
			dd+=1
		while(dd<20)
		NumMaxCorrTM[cc+1]=8
		NumMaxCorrTP[cc+1]=8
		NumMaxCorrCM[cc+1]=2
		NumMaxCorrCP[cc+1]=2
		cc+=1
	while(cc<20)
	OdorValve=SPlusValve*(OdorValveTemp==1)+SMinusValve*(OdorValveTemp==0)
+SPlusControlValve*(OdorValveTemp==3) + SMinusControlValve*(OdorValveTemp==2)
	Reward=(OdorValveTemp==1)
	Start()
ENDMACRO

// Entry point to start training session in pseudorandomized mode
MACRO StartTraining_PseuRand()
	variable cc,dd
	TrainingMode = "Pseudo Randomized"
	cc=0
	do	// first two blocks of OdorValve assigned out of dummyT  1  2  3
		dd=0
		do
			OdorValveTemp[cc*20+dd+1]=$("dummyT"+num2str(cc+1))[dd]
			dd+=1
		while(dd<20)
		NumMaxCorrTM[cc+1]=10 // number max correct trials minus
		NumMaxCorrTP[cc+1]=10 // number max correct trials plus
```

```
            NumMaxCorrCM[cc+1]=0
            NumMaxCorrCP[cc+1]=0
            cc+=1
      while(cc<3)

      do // blocks 4 to 10
            dd=0
            do

      OdorValveTemp[cc*20+dd+1]=$("dummyT"+num2str(mod(cc,3)+1))[dd] +
(mod(cc,3)*2)
                  dd+=1
            while(dd<20)
            NumMaxCorrTM[cc+1]=10
            NumMaxCorrTP[cc+1]=10
            NumMaxCorrCM[cc+1]=0
            NumMaxCorrCP[cc+1]=0
            cc+=1
      while(cc<10)

      do // blocks 11 to 50
            dd=0
            do

      OdorValveTemp[cc*20+dd+1]=$("dummyT"+num2str(mod(cc,3)+1))[dd] +
((mod(cc+1,4))*2)
                  dd+=1
            while(dd<20)
            NumMaxCorrTM[cc+1]=10
            NumMaxCorrTP[cc+1]=10
            NumMaxCorrCM[cc+1]=0
            NumMaxCorrCP[cc+1]=0
            cc+=1
      while(cc<50)          // total number of 1000 trials

      OdorValve=SPlusValve*(OdorValveTemp==1)+SMinusValve*(OdorValveTemp==0)
+SPlus2nd*(OdorValveTemp==3) + SMinus2nd*(OdorValveTemp==2)
      OdorValve=OdorValve+SPlus3rd*(OdorValveTemp==5) +
SMinus3rd*(OdorValveTemp==4)
      OdorValve=OdorValve+SPlus4th*(OdorValveTemp==7) +
SMinus4th*(OdorValveTemp==6)
      Reward=(OdorValveTemp==1)+(OdorValveTemp==3)+(OdorValveTemp==5)+(Odo
rValveTemp==7)
      Start()
ENDMACRO

// Entry point for training session in  fully randomized mode
MACRO StartTraining_Rand()
      variable i,cc,dd, countP, countM, larnull, smanull, randsub
      TrainingMode = "Fully Randomized"
```

```
silent 1
OdorValveTemp=enoise(1)  // random number between -1 and +1 assigned
// assing binary values and sort out stretches >3 times the same number
larnull = 0
smanull = 0
i=0
do
	if (OdorValveTemp[i]>0)
		OdorValveTemp[i] = 1
		larnull +=1
		smanull = 0
	else
		OdorValveTemp[i] = 0
		smanull +=1
		larnull=0
	endif
	if (larnull >3)
		randsub = round(enoise(0.5)+1.5) // random determination of which of the preceding stretch to change (either -1 or -2)
		OdorValveTemp[i-randsub] = 0
		larnull = 0
		smanull = 0
	endif
	if (smanull >3)
		randsub = round(enoise(0.5)+1.5) // random determination of which of the preceding stretch to change (either -1 or -2)
		OdorValveTemp[i-randsub] = 1
		smanull = 0
		larnull = 0
	endif
	i+=1
while (i<1000)

cc=0   //block
do
	dd=0
	CountP=0
	CountM=0
	do
		CountP+=mod(OdorValveTemp[cc*20+dd+1],2)
		CountM+=1-mod(OdorValveTemp[cc*20+dd+1],2)
		dd+=1
	while (dd<20)
	NumMaxCorrTM[cc+1]=CountM
	NumMaxCorrTP[cc+1]=CountP
	NumMaxCorrCM[cc+1]=0
	NumMaxCorrCP[cc+1]=0
	cc+=1
while(cc<50)
```

```
	cc = 3
	do // blocks 4 to 10
		dd=0
		do
			OdorValveTemp[cc*20+dd+1]=OdorValveTemp[cc*20+dd+1] +
(mod(cc,3)*2)
			dd+=1
		while(dd<20)
		cc+=1
	while(cc<10)

	do // blocks 11 to 50
		dd=0
		do
			OdorValveTemp[cc*20+dd+1]=OdorValveTemp[cc*20+dd+1] +
((mod(cc+1,4))*2)
			dd+=1
		while(dd<20)
		cc+=1
	while(cc<50)		// total number of 1000 trials

	OdorValve=SPlusValve*(OdorValveTemp==1)+SMinusValve*(OdorValveTemp==0)
+SPlus2nd*(OdorValveTemp==3) + SMinus2nd*(OdorValveTemp==2)
	OdorValve=OdorValve+SPlus3rd*(OdorValveTemp==5) +
SMinus3rd*(OdorValveTemp==4)
	OdorValve=OdorValve+SPlus4th*(OdorValveTemp==7) +
SMinus4th*(OdorValveTemp==6)
	Reward=(OdorValveTemp==1)+(OdorValveTemp==3)+(OdorValveTemp==5)+(Odo
rValveTemp==7)

	Start()
ENDMACRO

// Entry point for training session in  fully randomized mode
MACRO StartTraining_FullRand()
	variable i,cc,dd, countP, countM, larnull, smanull, randsub
	TrainingMode = "Fully Randomized"
	silent 1
	OdorValveTemp=enoise(1)  // random number between -1 and +1 assigned
	// assing binary values and sort out stretches >3 times the same number
	larnull = 0
	smanull = 0
	i=0
	do
		if (OdorValveTemp[i]>0)
			OdorValveTemp[i] = 1
			larnull +=1
			smanull = 0
		else
			OdorValveTemp[i] = 0
```

```
				smanull +=1
				larnull=0
			endif
			if (larnull >4)
				randsub = round(enoise(0.5)+1.5) // random determination of which of
the preceding stretch to change (either -1 or -2)
				OdorValveTemp[i-randsub] = 0
				larnull = 0
				smanull = 0
			endif
			if (smanull >4)
				randsub = round(enoise(0.5)+1.5) // random determination of which of
the preceding stretch to change (either -1 or -2)
				OdorValveTemp[i-randsub] = 1
				smanull = 0
				larnull = 0
			endif
			i+=1
		while (i<1000)

		cc=0
		CountP=0
		CountM=0
			do
		OdorValveTemp[cc]=OdorValveTemp[cc]+((mod(cc,4)*2))
			cc+=1
		while(cc<1000)		// total number of 1000 trials

		OdorValve=SPlusValve*(OdorValveTemp==1)+SMinusValve*(OdorValveTemp==0)
+SPlus2nd*(OdorValveTemp==3) + SMinus2nd*(OdorValveTemp==2)
		OdorValve=OdorValve+SPlus3rd*(OdorValveTemp==5) +
SMinus3rd*(OdorValveTemp==4)
		OdorValve=OdorValve+SPlus4th*(OdorValveTemp==7) +
SMinus4th*(OdorValveTemp==6)
		Reward=(OdorValveTemp==1)+(OdorValveTemp==3)+(OdorValveTemp==5)+(Odo
rValveTemp==7)

		Start()
ENDMACRO

// activated by entry point macros which get started by START button
MACRO Start()
		Print "Reinforcement time", reinforcementtime
		TrialNumber=1
		BlockNumber=1
		FirstTrialINBlock=1
		Responsewave[0][10]="S+";Responsewave[1][10]="S--"; //
Responsewave[2][10]="C --";Responsewave[3][10]="C +"
		SetBackground checkBeam();
		ctrlbackground period=0,start
```

```
ENDMACRO

MACRO DisplayResults()
	string Wana
	wana =
"ValveResponse"+num2str(OdorValve[TrialNumber])+"_"+num2str(Reward[TrialNumber])
	$wana[TrialNumber] = Accepted[TrialNumber]
	//SetAxis bottom 0,TrialNumber
ENDMACRO

//=========================== Save Data ===========================

MACRO SaveAll()
	Save/C /O /P=SaveData LickPattern as SaveFileName+"_Lick.dat"
	Save/C /O /P=SaveData SamplePattern as SaveFileName+"_Sample.dat"
	Save/C /O /P=SaveData CorrCM as SaveFileName+"_CorrCm.dat"
	Save/C /O /P=SaveData CorrCP as SaveFileName+"_CorrCp.dat"
	Save/C /O /P=SaveData CorrSMin as SaveFileName+"_Corrsmin.dat"
	Save/C /O /P=SaveData CorrSPlus as SaveFileName+"_CorrSPlus.dat"
	Save/C /O /P=SaveData OdorValveTemp as SaveFileName+"_O.dat"
	Save/C /O /P=SaveData Accepted as SaveFileName+"_Accepted.dat"
	Save/C /O /P=SaveData Reward as SaveFileName+"_Rewarded.dat"
	Save/C /O /P=SaveData OdorValve as SaveFileName+"_OdorValve.dat"

Print "Your data was successfully saved"
Print "Location: C:Training_logfiles"
ENDMACRO

FUNCTION NewBlock()
	execute("NewBlockMacro()")
END

MACRO NewBlockMacro()
	variable c
	SPlusCount=0
	SMinusCount=0
	ResponseWave=""
	c=1
	c=0
	CorrSPlus[BlockNumber]=CorrSPlus[BlockNumber]/NumMaxCorrTP[BlockNumber]
*100
	CorrSMin[BlockNumber]=CorrSMin[BlockNumber]/NumMaxCorrTM[BlockNumber]*
100
	CorrCP[BlockNumber]=CorrCP[BlockNumber]/NumMaxCorrCP[BlockNumber]*100
	CorrCM[BlockNumber]=CorrCM[BlockNumber]/NumMaxCorrCM[BlockNumber]*100

	FirstTrialInBlock=TrialNumber
	BlockNumber+=1
	Save/J/M="\r\n"/W /O /P=SaveData OdorValve,Reward,Accepted,TrialTime as
SaveFileName+"_tmp1.dat"
```

```
	Save/C /O /P=SaveData LickPattern as SaveFileName+"_tmpLick"
	Save/C /O /P=SaveData SamplePattern as SaveFileName+"_tmpSample"
	// SaveAll()
ENDMACRO


//======================== Training Procedure =====================

// OdorValve contains infromation about the valve that has to open for the next odor
// Reward is True if the next presentation is (potentially) rewarded
// TrialNumber contains the number of the trial (1-... (approx 200))
// Accepted[TrialNumber] contains 1 if the stimulus was accepted (criterion met), 0 if it was not accpeted and -1 if it wasnt sampled properly (not counted)
// lickPattern[TrialNumber][xx] contains the lickPattern (1 for licking at this paticular time)
// samplePattern[TrialNumber][xx] contains the samplePattern (1 for sampling at this paticular time)
// Rewarded[TrialNumber]
// time is measured in approximately 3 ms blocks, an accurate timer that is updated only everz 3 ms ensures that

FUNCTION TrialBegin()

	WAVE Reward=Reward
	WAVE CorrSPLus=CorrSPlus
	WAVE CorrSMin=CorrSMin
	WAVE CorrCM=CorrCM
	WAVE CorrCP=CorrCP
	WAVE Accepted=Accepted
	WAVE lickPattern=lickPattern
	WAVE samplePattern=samplePattern
	WAVE OdorValve=OdorValve
	WAVE /T ResponseWave=ResponseWave
	WAVE lickwavetmp = lickwavetmp
	WAVE samplewavetmp = samplewavetmp
	NVAR TrialNumber=TrialNumber
	NVAR SPlusValve=SPlusValve
	NVAR SPlus2nd=SPlus2nd
	NVAR SPlus3rd=SPlus3rd
	NVAR SPlus4th=SPlus4th
	NVAR SMinusValve=SMinusValve
	NVAR SMinus2nd=SMinus2nd
	NVAR SMinus3rd=SMinus3rd
	NVAR SMinus4th=SMinus4th
	NVAR SPlusControlValve=SPlusControlValve
	NVAR SMinusControlValve=SMinusControlValve
	NVAR ITICount=ITICount
	SVAR WhatsUp=WhatsUp
	NVAR IsITI=IsITI
	NVAR ITIinSeconds=ITIinSeconds
	NVAR SPlusCount=SPlusCount
```

```
	NVAR SMinusCount=SMinusCount
	NVAR BlockNumber=BlockNumber
	variable count, timerNum, timerNum2, timerNumAccurate, runTime, lickTime,
sampleTime, bbTmp, ilTmp, runTimeAccurate, count2, NumTrialsINBlock=20, CountPLus,
CountMinus

    lickwavetmp=0; samplewavetmp=0

	print "=============================================="
	print "TrialBegin", Trialnumber
	timerNum2=startMSTimer
	timerNum=startMSTimer
	timerNumAccurate=startMSTimer
	runTime=0
	lickTime=0
	sampleTime=0
	OpenFinalValve()						//Final Valve Opens
	ApplyOdor(OdorValve[TrialNumber])				//Odour is Applied

	count=0
	do
		runTime+=stopMSTimer(timerNum)
	timerNum=startMSTimer
		bbTmp=BeamBroken()
		sampleTime+=bbTmp
		ilTmp = IsLicked()
		lickTime+=ilTmp
		lickWaveTmp[count]=ilTmp
		sampleWaveTmp[count]=bbTmp
		count+=1
		if (runTime/1000>3)
		  runTimeAccurate+=stopMSTimer(timerNumAccurate)
		  timerNumAccurate=startMSTimer
		  runTime=0
		endif
	while (runTimeAccurate/1000<500 )	// 500 ms diversion valve
	closeFinalValve()		//Final Valve Closes

	do
		runTime+=stopMSTimer(timerNum)
	timerNum=startMSTimer
		bbTmp=BeamBroken()
		sampleTime+=bbTmp
		ilTmp = IsLicked()
		lickTime+=ilTmp
		lickWaveTmp[count]=ilTmp
		sampleWaveTmp[count]=bbTmp
		count+=1
		if (runTime/1000>3)
		  runTimeAccurate+=stopMSTimer(timerNumAccurate)
```

```
			timerNumAccurate=startMSTimer
			runTime=0
		endif
	while (runTimeAccurate/1000<2500 ) // 2000 ms odor application
	print " time Elapsed is ", stopMSTimer(timerNum2)/1000
	print "time licked     : ", lickTime/count*100, " % "
	print "time sampled : ", sampleTime/count*100, " % "
	print " number of counts ", count

	count2=0
	do
	  wavestats /Q /R=[count2*count/125, (count2+1)*count/125] lickWaveTmp
	  lickPattern[TrialNumber][count2]=V_avg
	  wavestats /Q /R=[count2*count/125, (count2+1)*count/125] sampleWaveTmp
	  samplePattern[TrialNumber][count2]=V_avg
	  count2+=1
	while(count2<125)
	EmptyTimers()

	Accepted[TrialNumber]=IsCriterion() // -1 if too short, 1 if lick ok, 0 if no lick
	print "Accepted trialnumber",Accepted[TrialNumber]
	Execute("TimeStamp()")

	if(Reward[TrialNumber] && Accepted[TrialNumber]==1)
	print "Now Reward"
	  RewardIt()
	endif

	ShutDownOdor(OdorValve[TrialNumber])
	//Odour Valve Closes

	if (Accepted[TrialNumber]==1)

	if((Odorvalve[TrialNumber]==SPlusValve)+(Odorvalve[TrialNumber]==SPlus2nd)+(
Odorvalve[TrialNumber]==SPlus3rd)+(Odorvalve[TrialNumber]==SPlus4th))
				ResponseWave[0][SPlusCount]="+"
				SPlusCount+=1
				CorrSPlus[BlockNumber]+=1
		endif

	if((Odorvalve[TrialNumber]==SMinusValve)+(Odorvalve[TrialNumber]==SMinus2nd)
+(Odorvalve[TrialNumber]==SMinus3rd)+(Odorvalve[TrialNumber]==SMinus4th))
				ResponseWave[1][SMinusCount]="--"
				SMinusCount+=1
		endif
		if(Odorvalve[TrialNumber]==SPlusControlValve)
				ResponseWave[0][SPlusCount]="+C"
				SPlusCount+=1
				CorrCP[BlockNumber]+=1
		endif
```

```
		if(Odorvalve[TrialNumber]==SMinusControlValve)
			ResponseWave[1][SMinusCount]="--C"
			SMinusCount+=1
		endif
	endif

	if (Accepted[TrialNumber]==0)

	if((Odorvalve[TrialNumber]==SPlusValve)+(Odorvalve[TrialNumber]==SPlus2nd)+(
Odorvalve[TrialNumber]==SPlus3rd)+(Odorvalve[TrialNumber]==SPlus4th))
			ResponseWave[0][SPlusCount]="--"
			SPlusCount+=1
		endif

	if((Odorvalve[TrialNumber]==SMinusValve)+(Odorvalve[TrialNumber]==SMinus2nd)
+(Odorvalve[TrialNumber]==SMinus3rd)+(Odorvalve[TrialNumber]==SMinus4th))
			ResponseWave[1][SMinusCount]="+"
			SMinusCount+=1
			CorrSMin[BlockNumber]+=1
		endif
		if(Odorvalve[TrialNumber]==SPlusControlValve)
			ResponseWave[0][SPlusCount]="--C"
			SPlusCount+=1
		endif
		if(Odorvalve[TrialNumber]==SMinusControlValve)
			ResponseWave[1][SMinusCount]="+C"
			SMinusCount+=1
			CorrCM[BlockNumber]+=1
		endif
	endif

	if (Accepted[TrialNumber]>-1)
		execute("DisplayResults()")
		TrialNumber+=1
			if(Mod(TrialNumber, NumTrialsINBlock)==1)
				NewBlock()
			endif
		print "advanced a trial - now ITI"
		ITICount=0
		WhatsUp="ITI"
		IsITI=1
		ctrlbackground period=60,start
	else
		IsITI=0
		ctrlbackground period=0,start
	endif
END

//==========================Background Functions - Related to the Training
Procedure =====================
```

```
Function checkBeam()
	NVAR nBeam=nBeam
	NVAR IsITI=IsITI
	SVAR WhatSUp = WhatSUp
	NVAR ITICount=ITICount
	NVAR ITIinSeconds=ITIinSeconds

	if(IsITI)
		if (ITICount>ITIinSeconds&&BeamBroken()==0)
			ctrlbackground period=60,stop
			ctrlbackground period=0,start
			WhatsUp="Wait For Beam Break"
			IsITI=0
		else
			ITICount+=1
			WhatsUp="Inter trial interval - "+ num2str(ITICount)
		endif
	else
		if (BeamBroken())
			nBeam+=1
			TrialBegin()
		endif
	endif
	return 0
End

FUNCTION RewardIt()
  NVAR ReinforcementTime=ReinforcementTime
  variable timerNum, timerNumAccurate, runTime
  timerNum=startMSTimer
  OpenWaterValve()
  do
	runTime+=stopMSTimer(timerNum)
	timerNum=startMSTimer
  while(runTime/1000<ReinforcementTime)
  CloseWaterValve()
  runTime+=stopMSTimer(timerNum)
END

FUNCTION ApplyOdor(ValveNo)
variable ValveNo
 OpenValve(ValveNo)
END

FUNCTION ShutDownOdor(ValveNo)
variable ValveNo
 CloseValve(ValveNo)
END
```

```
FUNCTION IsCriterion()
  WAVE lickPattern, samplePattern, lTmp, sTmp
  NVAR TrialNumber=TrialNumber
  variable /g SamplePercent=0.22

  lTMp = 0
  sTmp = 0
  lTmp=lickPattern[TrialNumber][p]
  sTmp=samplePattern[TrialNumber][p]

  variable lick1, lick2, lick3, lick4, sampled
  wavestats /Q /R=[25,49] lTmp;  lick1=(V_avg>0)
  wavestats /Q /R=[50,74] lTmp;  lick2=(V_avg>0)
  wavestats /Q /R=[75,99] lTmp;  lick3=(V_avg>0)
  wavestats /Q /R=[100,124] lTmp;  lick4=(V_avg>0)
  wavestats /Q sTmp; sampled = V_avg
  if(sampled<samplepercent)
    return -1              // too short sample
  else
    Print (lick1>0?1:0),(lick2>0?1:0),(lick3>0?1:0),(lick4>0?1:0)
    return (lick1+lick2+lick3+lick4>2?1:0)            // if more than 2 time bins show licking,
return 1 otherwise 0
  endif
END

FUNCTION EmptyTimers()
variable i,k
  do
    k=StopMSTimer(i)
    i+=1
  while(i<10)
END

Macro TimeStamp()
        TrialTime[TrialNumber]=time()
end

//========================== Statistics ==============================

MACRO DisplaySimple()
        duplicate /O CorrSMin, CorrSMinDup
        duplicate /O CorrSMin, PercentCorr
        CorrSMinDup=100-CorrSMinDup
        display CorrSPlus, CorrSMinDup
        label left "% accepted"
        Label bottom "Block Number"
        ModifyGraph
mode=4,marker(CorrSPlus)=8,rgb(CorrSPlus)=(0,52224,0);DelayUpdate
        ModifyGraph marker(CorrSMinDup)=17
        SetAxis Bottom 1, blockNumber-1
```

```
	DoWindow/C/T TrainingTrialsGraph,"Training Trials"
	PercentCorr=(CorrSMin+CorrSPlus)/2
	appendtograph PercentCorr
	ModifyGraph mode=4,marker(PercentCorr)=16,rgb(PercentCorr)=(0,0,0)
	duplicate /O CorrCM, CorrCMDup
	CorrCMDup=100-CorrCMDup
	display CorrCP CorrCMDup
	label left "% accepted"
	Label bottom "Block Number"
	ModifyGraph mode=4,marker(CorrCP)=8,rgb(CorrCP)=(0,52224,0);DelayUpdate
	ModifyGraph marker(CorrCMDup)=17
	SetAxis Bottom 1, blockNumber-1
	DoWindow/C/T ControlTrialsGraph,"Control Trials"
ENDMACRO

MACRO AverageMarked()
	make /N=125 /O sampleAverage=0, lickAverage=0
	string lickWaveName="LickAVG", sampleWaveName="SampleAVG"
	variable howMany
	variable cc
		wavestats /Q averagemark
		howMany = V_avg*V_npnts
		cc=0
			do
				sampleAverage+=AverageMark[cc]*SamplePattern[cc][p]
				lickAverage+=AverageMark[cc]*LickPattern[cc][p]
				cc+=1
			while (cc<TrialNumber+10)
		sampleAverage/=HowMany
		lickAverage/=HowMany
		duplicate /O lickAverage, $(lickWaveName)
		duplicate /O sampleAverage, $(sampleWaveName)
		display $(lickWaveName)
		display $(sampleWaveName)
ENDMACRO

MACRO AverageAllSPlus()
	averageMark = (OdorValveTemp==1)*(Accepted>-1)
	AverageMarked()
ENDMACRO

MACRO AverageAllSMinus()
	averageMark = (OdorValveTemp==0)*(Accepted>-1)
	AverageMarked()
ENDMACRO

MACRO AverageAllCorrSPlus()
	averageMark = (OdorValveTemp==1)*(Accepted==1)
	AverageMarked()
ENDMACRO
```

```
MACRO AverageAllCorrSMinus()
	averageMark = (OdorValveTemp==0)*(Accepted==0)
	AverageMarked()
ENDMACRO

MACRO AverageAllInCorrSPlus()
	averageMark = (OdorValveTemp==1)*(Accepted==0)
	AverageMarked()
ENDMACRO

MACRO AverageAllInCorrSMinus()
	averageMark = (OdorValveTemp==0)*(Accepted==1)
	AverageMarked()
ENDMACRO


//========================== Panel Specifications ====================

													//Main Panel//

Window MainPanel() : Panel
	PauseUpdate; Silent 1		// building window...
	NewPanel /W=(3,57,614,261) as "MainPanel"
	SetDrawLayer UserBack
	SetDrawEnv fsize= 14,fstyle= 1
	DrawText 89,26,"Training Programm - 4 Odour Pairs"
	DrawRect 393,106,565,190
	ValDisplay BlockNumber,pos={12,49},size={91,15},title="BlockNumber"
	ValDisplay BlockNumber,limits={0,0,0},barmisc={0,1000},value= #"BlockNumber"
	SetVariable SamplePercent,pos={5,74},size={134,16},title="Required SampleTime"
	SetVariable SamplePercent,limits={-1,1,0},value= SamplePercent
	SetVariable WhatsUp,pos={265,50},size={116,16},title=" ",value= WhatSUp
	SetVariable ITIInSeconds,pos={321,76},size={63,16},title="ITI / s"
	SetVariable ITIInSeconds,limits={0,inf,0},value= ITIInSeconds
	ValDisplay TrialNumber,pos={130,50},size={91,15},title="TrialNumber"
	ValDisplay TrialNumber,limits={0,0,0},barmisc={0,1000},value= #" TrialNumber"
	SetVariable ReinforcementTime,pos={150,75},size={155,16},title="Reinforcment
Time / ms"
	SetVariable ReinforcementTime,limits={-inf,inf,0},value= ReinforcementTime
	SetVariable SPlusValve,pos={3,109},size={87,16},title="S+ Valve 1"
	SetVariable SPlusValve,limits={0,256,0},value= SPlusValve
	SetVariable SMinusValve,pos={112,109},size={86,16},title="S- Valve 1"
	SetVariable SMinusValve,limits={0,256,0},value= SMinusValve
	SetVariable SPlusValve02,pos={2,128},size={88,16},title="S+ Valve 2"
	SetVariable SPlusValve02,limits={0,260,0},value= SPlus2nd
	SetVariable SMinusValve02,pos={113,128},size={85,16},title="S- Valve 2"
	SetVariable SMinusValve02,limits={0,260,0},value= SMinus2nd
	SetVariable SPlusValve03,pos={2,148},size={88,16},title="S+ Valve 3"
	SetVariable SPlusValve03,limits={0,260,0},value= SPlus3rd
```

```
	SetVariable SMinusValve03,pos={112,148},size={86,16},title="S- Valve 3"
	SetVariable SMinusValve03,limits={0,260,0},value= SMinus3rd
	SetVariable SPlusValve04,pos={2,167},size={88,16},title="S+ Valve 4"
	SetVariable SPlusValve04,limits={0,260,0},value= SPlus4th
	SetVariable SMinusValve04,pos={112,168},size={86,16},title="S- Valve 4"
	SetVariable SMinusValve04,limits={0,260,0},value= SMinus4th
	Button
GoButton,pos={490,139},size={49,25},proc=ButtonProc_Resume,title="Resume"
	Button
StopButton,pos={418,139},size={51,25},proc=ButtonProc_STOP,title="STOP"
	Button
Start_pSUEDO_RANDOMIZED_sESSION,pos={217,104},size={151,25},proc=ButtonProc
_StartPseudRand,title="START Pseudo Randomized"
	Button
Start_fully_randomized_session,pos={216,135},size={152,24},proc=ButtonProc_StartRand
omized,title="START Randomized"
	Button
StartMemoryTrainning,pos={217,164},size={150,27},proc=ButtonProc_StartMemory,title="
START Memory"
	SetVariable TrainingMode,pos={418,49},size={148,16},title="Training Mode "
	SetVariable TrainingMode,value= TrainingMode
	Button SaveData,pos={433,75},size={97,21},proc=ButtonProc_SaveAll,title="Save
data"
EndMacro

// ================= START training ====================
Function ButtonProc_StartPseudRand(ctrlName) : ButtonControl
	String ctrlName
	execute("StartTraining_PseuRand()")
End

Function ButtonProc_StartRandomized(ctrlName) : ButtonControl
	String ctrlName
	execute ("StartTraining_FullRand()")
End

Function ButtonProc_StartMemory(ctrlName) : ButtonControl
	String ctrlName
	Execute ("StartMemory()")
End

// ================= resume training ====================
Function ButtonProc_Resume(ctrlName) : ButtonControl
	String ctrlName
	SetBackground checkBeam();
	ctrlbackground period=0,start

	print "Program Resarted"

End
```

```
// ================ STOP training ===================
Function ButtonProc_STOP(ctrlName) : ButtonControl
	String ctrlName
	SVAR WhatsUp=WhatsUp
	WhatsUp = "Stopped"
	Execute ("SaveAll()")
	ctrlbackground period=60,stop
End


Function ButtonProc_SaveAll(ctrlName) : ButtonControl
	String ctrlName
	execute ("SaveAll()")
End


//  ======================= ValvePanel ===============================

Window ValvePanel() : Panel
	PauseUpdate; Silent 1		// building window...
	NewPanel /W=(622,57,823,261)
	CheckBox valve1,pos={32,18},size={51,14},proc=CheckProc_valve1,title="Valve1"
	CheckBox valve1,value= 1
	CheckBox valve2,pos={32,38},size={51,14},proc=CheckProc_valve2,title="Valve2"
	CheckBox valve2,value= 1
	CheckBox valve4,pos={32,56},size={51,14},proc=CheckProc_valve4,title="Valve4"
	CheckBox valve4,value= 1
	CheckBox valve8,pos={32,73},size={51,14},proc=CheckProc_valve8,title="Valve8"
	CheckBox valve8,value= 1
	CheckBox
valve16,pos={32,92},size={57,14},proc=CheckProc_valv16,title="Valve16"
	CheckBox valve16,value= 1
	CheckBox
valve32,pos={32,109},size={57,14},proc=CheckProc_valve32,title="Valve32"
	CheckBox valve32,value= 1
	CheckBox
valve64,pos={111,19},size={57,14},proc=CheckProc_valve64,title="Valve64"
	CheckBox valve64,value= 1
	CheckBox
valve128,pos={111,38},size={63,14},proc=CheckProc_valve128,title="Valve128"
	CheckBox valve128,value= 1
	CheckBox FV,pos={111,56},size={31,14},proc=CheckProc_FV,title="FV",value= 1
	CheckBox RF,pos={111,73},size={32,14},proc=CheckProc_RF,title="RF",value= 1
	ValDisplay lick,pos={113,92},size={50,15},title="Lick"
	ValDisplay lick,limits={0,0,0},barmisc={0,1000},value= #"lick"
	ValDisplay Beam,pos={111,109},size={50,15},title="Beam"
	ValDisplay Beam,limits={0,0,0},barmisc={0,1000},value= #"beam"
	Button Check,pos={19,146},size={50,20},proc=ButtonProc_Check,title="Check"
	Button Apply,pos={78,147},size={50,20},proc=ButtonProc_Apply,title="Apply"
```

```
	Button Init,pos={136,147},size={51,20},proc=ButtonProc_Off,title="Off"
EndMacro

Function ButtonProc_Check(ctrlName) : ButtonControl
	String ctrlName
	NVAR lick, beam
	lick = IsLicked()
	beam = BeamBroken()
End

Function ButtonProc_Apply(ctrlName) : ButtonControl
	String ctrlName
	NVAR valve1,valve2,valve4,valve8,valve16,valve32,valve64,valve128, RF, FV
	variable outBits
	outBits=128*valve128+64*valve64+32*valve32+16*valve16+8*valve8+4*valve4+2*
valve2+valve1
	OpenValve(outBits) // operate valves
	outBits=4*RF+1*FV
	OperateWaterFinal(outBits) // operate valves
End

Function ButtonProc_Off(ctrlName) : ButtonControl
	String ctrlName
	CloseValve(0)
	CloseFinalValve()
End

Function CheckProc_valve1(ctrlName,checked) : CheckBoxControl
	String ctrlName
	Variable checked
	NVAR valve1
	valve1=checked
End

Function CheckProc_valve2(ctrlName,checked) : CheckBoxControl
	String ctrlName
	Variable checked
	NVAR valve2
	valve2=checked
End

Function CheckProc_valve4(ctrlName,checked) : CheckBoxControl
	String ctrlName
	Variable checked
	NVAR valve4
	valve4=checked
End

Function CheckProc_valve8(ctrlName,checked) : CheckBoxControl
	String ctrlName
```

```
	Variable checked
	NVAR valve8
	valve8=checked
End

Function CheckProc_valv16(ctrlName,checked) : CheckBoxControl
	String ctrlName
	Variable checked
	NVAR valve16
	valve16=checked
End

Function CheckProc_valve32(ctrlName,checked) : CheckBoxControl
	String ctrlName
	Variable checked
	NVAR valve32
	valve32=checked
End

Function CheckProc_valve64(ctrlName,checked) : CheckBoxControl
	String ctrlName
	Variable checked
	NVAR valve64
	valve64=checked
End

Function CheckProc_valve128(ctrlName,checked) : CheckBoxControl
	String ctrlName
	Variable checked
	NVAR valve128
	valve128=checked
End


Function CheckProc_FV(ctrlName,checked) : CheckBoxControl
	String ctrlName
	Variable checked
	NVAR FV
	FV=checked
End

Function CheckProc_RF(ctrlName,checked) : CheckBoxControl
	String ctrlName
	Variable checked
	NVAR RF
	RF=checked
End
```

```
// ********************************************** Display Panel
**************************************************

Window DisplayPanel() : Panel
	PauseUpdate; Silent 1		// building window...
	NewPanel /W=(2,294,418,463)
	SetVariable LickWaveName,pos={4,10},size={139,16},title="Lick Pattern Name"
	SetVariable LickWaveName,value= lickWaveName
	SetVariable SampleWaveName,pos={156,11},size={166,16},title="Sample Pattern
Name"
	SetVariable SampleWaveName,value= sampleWaveName
	Button
buttonAvMark,pos={253,83},size={156,25},proc=ButtonProc_AvMarked,title="Average
Marked waves"
	Button
buttonSimpleDisplay,pos={265,48},size={125,24},proc=ButtonProc_DisplaySimple,title="Di
splay Simple Result"
	Button
buttonAvPLus,pos={17,34},size={100,25},proc=ButtonProc_AvSPlus,title="Average S+ "
	Button
buttonAvMinus,pos={128,34},size={101,25},proc=ButtonProc_AvSMinus,title="Average S-"
	Button
buttonAvCorrMinus,pos={129,66},size={100,26},proc=ButtonProc_AvCorrSMinus,title="Av
erage Corr S-"
	Button
buttonAvCorrPlus,pos={16,65},size={101,26},proc=ButtonProc_AvCorrSPlus,title="Averag
e Corr S+"
	Button
buttonAvInCorrPlus,pos={17,98},size={102,26},proc=ButtonProc_AvInCorrSPlus,title="Ave
rage Incorr S+"
	Button
buttonAvInCorrMinus,pos={130,97},size={100,27},proc=ButtonProc_AvInCorrSMinus,title=
"Average Incorr S-"
EndMacro

Function ButtonProc_AvMarked(ctrlName) : ButtonControl
	String ctrlName
	execute("AverageMarked()")
End

Function ButtonProc_DisplaySimple(ctrlName) : ButtonControl
	String ctrlName
	execute("DisplaySimple()")
End

Function ButtonProc_AvSPlus(ctrlName) : ButtonControl
	String ctrlName
	execute("AverageAllSPlus()")
End
```

```
Function ButtonProc_AvSMinus(ctrlName) : ButtonControl
	String ctrlName
	execute("AverageAllSMinus()")
End

Function ButtonProc_AvCorrSPlus(ctrlName) : ButtonControl
	String ctrlName
	execute("AverageAllCorrSPlus()")
End

Function ButtonProc_AvCorrSMinus(ctrlName) : ButtonControl
	String ctrlName
	execute("AverageAllCorrSMinus()")
End

Function ButtonProc_AvInCorrSPlus(ctrlName) : ButtonControl
	String ctrlName
	execute("AverageAllInCorrSPlus()")
End

Function ButtonProc_AvInCorrSMinus(ctrlName) : ButtonControl
	String ctrlName
	execute("AverageAllInCorrSMinus()")
End



//  ************************* Other Panels and Tables *********************************

Window Trials() : Panel
	PauseUpdate; Silent 1		// building window...
	NewPanel /W=(831,57,1134,263)
	ValDisplay valdisp0,pos={33,32},size={256,114},font="Arial",fSize=100
	ValDisplay valdisp0,limits={0,0,0},barmisc={0,1000},value= #"TrialNumber"
EndMacro


Window Table0() : Table
	PauseUpdate; Silent 1		// building window...
	Edit/W=(393,220.25,653.25,701)
OdorValve,Reward,Accepted,TrialTime,averageMark,OdorValveTemp
	ModifyTable
format(Point)=1,width(Point)=21,width(OdorValve)=51,width(Reward)=50
	ModifyTable width(Accepted)=60,width(TrialTime)=62,width(averageMark)=59
EndMacro

Window Table1() : Table
	PauseUpdate; Silent 1		// building window...
	Edit/W=(660.75,220.25,952.5,705.5) CorrSPlus,CorrSMin,CorrCP,CorrCM
```

```
	ModifyTable
format(Point)=1,width(Point)=23,width(CorrSPlus)=57,width(CorrSMin)=54
	ModifyTable width(CorrCP)=50,width(CorrCM)=51
EndMacro

Window Table2() : Table
	PauseUpdate; Silent 1		// building window...
	Edit/W=(105.75,151.25,800.25,506)
dummyT1,dummyT2,dummyT3,dummyC1,dummyC2,dummyC3
	ModifyTable format(Point)=1
EndMacro

Window Table3() : Table
	PauseUpdate; Silent 1		// building window...
	Edit/W=(396.75,593.75,954,708.5) Responsewave
	ModifyTable format(Point)=1,width(Point)=20,width(Responsewave)=47
EndMacro

//========================= Test Functions
========================================

Function myHelloFunc()
	print "hello"
	NVAR gHelloCnt = gHelloCnt
	gHelloCnt += 1
	if( gHelloCnt >= 4 )
		Print "background task finished"
		return 1	// time to quit
	else
		return 0
	endif
End


Macro WaterRealeseTest()
	variable i

	Print "Test Beggin"
	Print "The Reinforcement Time is:", ReinforcementTime
	i=1
	do
		Print "", i
		Rewardit()
		i+=1
		sleep 00:00:01
	while (i<11)
	Print "Test Finnished"
Endmacro
```

```
Function SetVarProc(ctrlName,varNum,varStr,varName) : SetVariableControl
	String ctrlName
	Variable varNum
	String varStr
	String varName
	NVAR ReinforcementTime=ReinforcementTime

End


// ====================== Still needed?

Window Graph1() : Graph
	PauseUpdate; Silent 1		// building window...
	Display /W=(354,301.25,847.5,503)
ValveResponse1_0,ValveResponse1_1,ValveResponse2_0
	AppendToGraph
ValveResponse2_1,ValveResponse4_0,ValveResponse4_1,ValveResponse8_0
	AppendToGraph
ValveResponse8_1,ValveResponse16_0,ValveResponse16_1,ValveResponse32_0
	AppendToGraph
ValveResponse32_1,ValveResponse64_0,ValveResponse64_1,ValveResponse128_0
	AppendToGraph ValveResponse128_1,ttmp
	ModifyGraph
mode(ValveResponse1_0)=3,mode(ValveResponse1_1)=3,mode(ValveResponse2_0)=3
	ModifyGraph
mode(ValveResponse2_1)=3,mode(ValveResponse4_0)=3,mode(ValveResponse4_1)=3
	ModifyGraph
mode(ValveResponse8_0)=3,mode(ValveResponse8_1)=3,mode(ValveResponse16_0)=3
	ModifyGraph
mode(ValveResponse16_1)=3,mode(ValveResponse32_0)=3,mode(ValveResponse32_1)
=3
	ModifyGraph
mode(ValveResponse64_0)=3,mode(ValveResponse64_1)=3,mode(ValveResponse128_
0)=3
	ModifyGraph mode(ValveResponse128_1)=3
	ModifyGraph
marker(ValveResponse1_0)=17,marker(ValveResponse1_1)=17,marker(ValveResponse2
_0)=19
	ModifyGraph
marker(ValveResponse2_1)=19,marker(ValveResponse4_0)=16,marker(ValveResponse4
_1)=16
	ModifyGraph
marker(ValveResponse8_0)=5,marker(ValveResponse8_1)=5,marker(ValveResponse16_
0)=8
	ModifyGraph
marker(ValveResponse16_1)=8,marker(ValveResponse32_0)=23,marker(ValveResponse
32_1)=23
```

```
	ModifyGraph marker(ValveResponse64_0)=24,marker(ValveResponse64_1)=24,marker(ValveResponse128_0)=15
	ModifyGraph marker(ValveResponse128_1)=15
	ModifyGraph rgb(ValveResponse1_1)=(0,52224,0),rgb(ValveResponse2_1)=(0,52224,0)
	ModifyGraph rgb(ValveResponse4_1)=(0,52224,0),rgb(ValveResponse8_1)=(0,52224,0)
	ModifyGraph rgb(ValveResponse16_1)=(0,52224,0),rgb(ValveResponse32_1)=(0,52224,0)
	ModifyGraph rgb(ValveResponse64_1)=(0,52224,0),rgb(ValveResponse128_1)=(0,52224,0)
	ModifyGraph nticks(left)=2
	SetAxis left -0.2,1.2
	SetAxis bottom 0,22
	CheckBox V1_IsRew,pos={0,1},size={40,14},value= 0
	CheckBox V2_IsRew,pos={60,1},size={40,14},value= 0
	CheckBox V3_IsRew,pos={110,1},size={40,14},value= 0
	CheckBox V4_IsRew,pos={170,1},size={40,14},value= 0
	CheckBox V5_IsRew,pos={220,1},size={40,14},value= 0
	CheckBox V6_IsRew,pos={280,1},size={40,14},value= 0
	CheckBox V7_IsRew,pos={330,1},size={40,14},value= 0
	CheckBox V8_IsRew,pos={390,1},size={40,14},value= 0
	CheckBox V9_IsRew,pos={440,1},size={40,14},value= 0
	CheckBox V10_IsRew,pos={500,1},size={40,14},value= 0
	CheckBox V11_IsRew,pos={0,20},size={40,14},value= 0
	CheckBox V12_IsRew,pos={60,20},size={40,14},value= 0
	CheckBox V13_IsRew,pos={110,20},size={40,14},value= 0
	CheckBox V14_IsRew,pos={170,20},size={40,14},value= 0
	CheckBox V15_IsRew,pos={220,20},size={40,14},value= 0
	CheckBox V16_IsRew,pos={280,20},size={40,14},value= 0
	CheckBox V17_IsRew,pos={330,20},size={40,14},value= 0
	CheckBox V18_IsRew,pos={390,20},size={40,14},value= 0
	CheckBox V19_IsRew,pos={440,20},size={40,14},value= 0
	CheckBox V20_IsRew,pos={500,20},size={40,14},value= 0
EndMacro

Window Table4() : Table
	PauseUpdate; Silent 1		// building window...
	Edit/W=(0,42.5,957,711.5) dummyT1
	ModifyTable format(Point)=1
EndMacro
```

5 – Behavior experiment: pre-training template

```
#pragma rtGlobals=1		// Use modern global access method.

//==================== Start Macros =====================
Macro Startup()

MainPanel()
Panel0()
Results()
Welcome()
Endmacro

MACRO Start()
	// Init
	variable /g  nBeam, IsITI, ITICount, ITIinSeconds, BB_counter, LickCounter,
GlobalStage, Corr, Incorr
	variable /g valve1, valve2, valve4, valve8, valve16, valve32, valve64, valve128,
ValveNo
	variable /g  Count0, Count1, Count2, Count3, Count4, Count5, Count6, Count7,
Count8
	variable /g bb0, bb1, bb2, bb3, bb4, bb5, bb6, bb7, bb8
	variable /g corr0, corr1, corr2, corr3, corr4, corr5, corr6, corr7, corr8
	variable /g incorr0, incorr1, incorr2, incorr3, incorr4, incorr5, incorr6, incorr7, incorr8
	string /g	WhatSUp
	make /o/n=4 RemLick
	// Panel init
	SetBackground CheckBeam();
	ctrlbackground period=0,start
	Print "New Experiment"
	Print "	**********************************************	"

ENDMACRO
//==================== Hardware ======================

Function InPortB(portNo)
variable portNo
return ReadPort(portNo)
End

Function OutPortB(portNo, ByteVal)
variable portNo, ByteVal
WritePort(portNo, ByteVal)
End


Function BeamBroken()
   if (InPOrtB(773)==1)
	return 0
   else
```

```
          return 1
    endif
End


Function IsLicked()
  if (inPortB(772)==1)
          return 1
    else
          return 0
    endif
End

Function EmptyTimers()
variable i,k

  do
    k=StopMSTimer(i)
    i+=1
  while(i<10)
End

Function OpenValve(valveNo)
variable valveNo
  OutPortB(768,valveNo)
End

Function CloseValve(valveNo)
variable valveNo
  OutPortB(768,0)
End

Function OpenWaterValve()
  OutPortB(770,5)
End

Function CloseWaterValve()
  OutPortB(770,0)
End

Function OpenFinalValve()
  OutPortB(770,2)
End

Function CloseFinalValve()
  OutPortB(770,0)
End

Function OperateWaterFinal(ValveNo)
variable ValveNo
```

```
OutPortB(770,ValveNo)
End

//==================== Pre_Training Procedure ===============

Function PreTraining()
	NVAR LickCounter=LickCounter
	NVAR GlobalStage=GlobalStage
	NVAR BB_Counter=BB_Counter
	NVAR Corr=Corr
	NVAR Incorr=Incorr
	NVAR Count0=Count0, Count1=Count1, Count2=Count2, Count3=Count3,
Count4=Count4, Count5=Count5, Count6=Count6, Count7=Count7, Count8=Count8
	NVAR bb0=bb0, bb1=bb1, bb2=bb2, bb3=bb3, bb4=bb4, bb5=bb5, bb6=bb6,
bb7=bb7, bb8=bb8
	NVAR corr0=corr0, corr1=corr1, corr2=corr2, corr3=corr3, corr4=corr4, corr5=corr5,
corr6=corr6, corr7=corr7, corr8=corr8
	NVAR incorr0=incorr0, incorr1=incorr1, incorr2=incorr2, incorr3=incorr3,
incorr4=incorr4, incorr5=incorr5, incorr6=incorr6, incorr7=incorr7, incorr8=incorr8
	Wave RemLick=RemLick
	Variable TimerNum, TimerNum2, TimerNumAccurate, runTimeAccurate, i, dummy,
runTime, LickTime, kk, ww, cc,dd, aa, bb

	Switch (GlobalStage)

	case 0:
		if (BeamBroken()==1)
		bb0+=1
		corr0+=1
		Count0+=1
		RewardIt()
			Print " Correct Trial", Time()
			Print "Phase 0, Trial Number", Count0
			Print "InterTrial Interval"
			Print "--------------------------------------------------------"
			InterTrialIntervalPre1()
			ctrlbackground period=60, start
		endif
	break
	case 1:
	If (BeamBroken()==1)
		bb1+=1
		do
			if(CheckIsLicked()==1)
			Count1+=1
			corr1+=1
			Print " Correct Trial", Time()
			Print "Phase 1, Trial Number", Count1
			Print "InterTrial Interval"
			Print "--------------------------------------------------------"
```

```
                CorrectTrial()
                ctrlbackground period=60, start
        endif
        while (BeamBroken())
endif
break
case 2:
        If (BeamBroken())
        bb2+=1
                do
                OpenValve(8)
                CheckIsLicked()
                        If (CheckIsLicked()==1)
                                LickCounter+=1
                                Count2+=1
                                Print " Correct Trial", Time()
                                Print "Phase 2, Trial Number", LickCounter
                                Print "InterTrial Interval"
                                Print "------------------------------------------------------------"
                                CloseValve(8)
                                Corr2+=1
                                CorrectTrial()
                                ctrlbackground period=60, start
                        else
                                InCorrectTrial()
                                incorr2=incorr
                                ctrlbackground period=0, start
                        endif
        while(BeamBroken())
endif
break
case 3:
        If (BeamBroken())
        bb3+=1
                do
                aa=StartMSTimer
                OpenFinalValve()
                OpenValve(8)
                        do
                                bb+=StopMSTimer(aa)
                                aa=StartMSTimer
                        while (bb/1000<100)
                CloseFinalValve()
                EmptyTimers()
                CheckIsLicked()
                        If (CheckIsLicked()==1)
                                do
                                        dummy=stopmstimer(i)
                                        i+=1
                                while (i<10)
```

```
                        timerNum2=startmstimer
                        timernum=startmstimer
                        timernumaccurate=startmstimer
                        runtimeaccurate=0
                        runtime=0
                        licktime=0
                        Silent(1)
                            do
                            runtime+=stopmstimer(timernum)
                            timernum=startmstimer
                                if (runtime/1000>100)

runtimeaccurate+=stopmstimer(timernumaccurate)
                                timernumaccurate=startmstimer
                                runtime=0
                                endif
                            while(runtimeaccurate/1000<250)
                        runtimeaccurate=0
                        LickCounter+=1
                        Count3+=1
                        Print " Correct Trial", time()
                        Print "Phase 3, Trail Number", LickCounter
                        Print "InterTrial Interval"
                        Print "------------------------------------------------------------"
                        Closevalve(8)
                        corr3+=1
                        CorrectTrial()
                        ctrlbackground period=60, start
                    else
                        IncorrectTrial()
                        incorr3=incorr
                        ctrlbackground period=0, start
                    endif
                while (BeamBroken())
            endif
        break
        case 4:
            if (BeamBroken())
            bb4+=1
                do
                aa=StartMSTimer
                OpenFinalValve()
                OpenValve(8)
                    do
                        bb+=StopMSTimer(aa)
                        aa=StartMSTimer
                    while (bb/1000<200)
                CloseFinalValve()
                EmptyTimers()
                CheckIsLicked()
```

```
                              If (CheckIsLicked()==1)
                                    do
                                          dummy=stopmstimer(i)
                                          i+=1
                                    while (i<10)
                                    timerNum2=startmstimer
                                    timernum=startmstimer
                                    timernumaccurate=startmstimer
                                    runtimeaccurate=0
                                    runtime=0
                                    licktime=0
                                    Silent(1)
                                          do
                                          runtime+=stopmstimer(timernum)
                                          timernum=startmstimer
                                                if (runtime/1000>100)

runtimeaccurate+=stopmstimer(timernumaccurate)
                                                timernumaccurate=startmstimer
                                                runtime=0
                                                endif
                                          while(runtimeaccurate/1000<500)
                                    runtimeaccurate=0
                                    LickCounter+=1
                                    Count4+=1
                                    Print " Correct Trial", time()
                                    Print "Phase 4, Trail Number", LickCounter
                                    Print "InterTrial Interval"
                                    Print "-------------------------------------------------------"
                                    Closevalve(8)
                                    corr4+=1
                                    CorrectTrial()
                                    ctrlbackground period=60, start
                              else
                                    InCorrectTrial()
                                    incorr4=incorr
                                    ctrlbackground period=0, start
                              endif
                        while (BeamBroken())
                  endif
            break
            Case 5:
                  If (BeamBroken())
                  bb5+=1
                        do
                        aa=StartMSTimer
                        OpenFinalValve()
                        OpenValve(8)
                              do
                                    bb+=StopMSTimer(aa)
```

```
                        aa=StartMSTimer
                while (bb/1000<250)
        CloseFinalValve()
        EmptyTimers()
        CheckIsLicked()
                If (CheckIsLicked()==1)
                        do
                                dummy=stopmstimer(i)
                                i+=1
                        while (i<10)
                        timerNum2=startmstimer
                        timernum=startmstimer
                        timernumaccurate=startmstimer
                        runtimeaccurate=0
                        runtime=0
                        licktime=0
                        Silent(1)
                                do
                                runtime+=stopmstimer(timernum)
                                timernum=startmstimer
                                        if (runtime/1000>100)

runtimeaccurate+=stopmstimer(timernumaccurate)
                                        timernumaccurate=startmstimer
                                        runtime=0
                                        endif
                                while(runtimeaccurate/1000<750)
                        runtimeaccurate=0
                        LickCounter+=1
                        Count5+=1
                        Print " Correct Trial", time()
                        Print "Phase 5, Trail Number", LickCounter
                        Print "InterTrial Interval"
                        Print "---------------------------------------------------------"
                        Closevalve(8)
                        corr5+=1
                        CorrectTrial()
                        ctrlbackground period=60, start
                else
                        InCorrectTrial()
                        incorr5=incorr
                        ctrlbackground period=0, start
                endif
        while (BeamBroken())
    endif
break
case 6:
    if (BeamBroken())
    bb6+=1
        do
```

```
aa=StartMSTimer
OpenFinalValve()
OpenValve(8)
        do
                bb+=StopMSTimer(aa)
                aa=StartMSTimer
        while (bb/1000<300)
CloseFinalValve()
EmptyTimers()
CheckIsLicked()
        if (CheckIsLicked()==1)
                do
                        dummy=stopmstimer(i)
                        i+=1
                while (i<10)
                timerNum2=startmstimer
                timernum=startmstimer
                timernumaccurate=startmstimer
                runtimeaccurate=0
                runtime=0
                licktime=0
                Silent(1)
                RemLick=0
                i=0
                do
                        do
                        runtime+=stopmstimer(timernum)
                        timernum=startmstimer
                                if (IsLicked()==1)
                                RemLick[i]=1
                                endif
                                        if (runtime/1000>100)

runtimeaccurate+=stopmstimer(timernumaccurate)
                                        timernumaccurate=startmstimer
                                        runtime=0
                                        endif
                        while(runtimeaccurate/1000<(500*(i+1)))
                        i+=1
                while (i<2)
                        if (sum(RemLick)>=2)
                                runtimeaccurate=0
                                LickCounter+=1
                                Count6+=1
                                Print " Correct Trial", time()
                                Print "Phase 6, Trail Number", LickCounter
                                Print "InterTrial Interval"
                                Print "---------------------------------------------
-----------"

                                Closevalve(8)
```

```
                                        corr6+=1
                                        CorrectTrial()
                                        ctrlbackground period=60, start
                                else
                                        InCorrectTrial()
                                        incorr6=incorr
                                        ctrlbackground period=60, start
                                endif
                else
                        InCorrectTrial()
                        incorr6=incorr
                        ctrlbackground period=0, start
                endif
        while (BeamBroken())
    endif
break
case 7:
    if (BeamBroken())
    bb7+=1
    do
        aa=StartMSTimer
        OpenFinalValve()
        OpenValve(8)
            do
                bb+=StopMSTimer(aa)
                aa=StartMSTimer
            while (bb/1000<400)
        CloseFinalValve()
        EmptyTimers()
        CheckIsLicked()
            if (CheckIsLicked()==1)
                do
                    dummy=stopmstimer(i)
                    i+=1
                while (i<10)
                timerNum2=startmstimer
                timernum=startmstimer
                timernumaccurate=startmstimer
                runtimeaccurate=0
                runtime=0
                licktime=0
                Silent(1)
                RemLick=0
                i=0
                    do
                        do
                        runtime+=stopmstimer(timernum)
                        timernum=startmstimer
                            if (IsLicked()==1)
                                RemLick[i]=1
```

```
                                        endif
                                            if (runtime/1000>100)

        runtimeaccurate+=stopmstimer(timernumaccurate)

        timernumaccurate=startmstimer
                                                runtime=0
                                            endif
                                    while(runtimeaccurate/1000<(500*(i+1)))
                                    i+=1
                                while (i<2)
                                    if (sum(RemLick)>=2)
                                    runtimeaccurate=0
                                    LickCounter+=1
                                    Count7+=1
                                    Print " Correct Trial". time()
                                    Print "Phase 7, Trail Number", LickCounter
                                    Print "InterTrial Interval"
                                    Print "-----------------------------------------------
-----------"

                                    Closevalve(8)
                                    corr7+=1
                                    CorrectTrial()
                                    ctrlbackground period=60, start
                                    else
                                        InCorrectTrial()
                                        incorr7=incorr
                                        ctrlbackground period=0, start
                                endif
                        else
                            InCorrectTrial()
                            incorr7=incorr
                        ctrlbackground period=0, start
                        endif
                    while (BeamBroken())
                endif
        break
        case 8:
                if (BeamBroken())
                bb8+=1
                    do
                    aa=StartMSTimer
                    OpenFinalValve()
                    OpenValve(8)
                        do
                            bb+=StopMSTimer(aa)
                            aa=StartMSTimer
                        while (bb/1000<500)
                    CloseFinalValve()
                    EmptyTimers()
```

```
                CheckIsLicked()
                        if (CheckIsLicked()==1)
                                do
                                        dummy=stopmstimer(i)
                                        i+=1
                                while (i<10)
                                timerNum2=startmstimer
                                timernum=startmstimer
                                timernumaccurate=startmstimer
                                runtimeaccurate=0
                                runtime=0
                                licktime=0
                                Silent(1)
                                RemLick = 0
                                i=0
                                        do
                                                do
                                                        runtime+=stopmstimer(timernum)
                                                        timernum=startmstimer
                                                                if (IsLicked()==1)
                                                                RemLick[i] = 1
                                                                endif
                                                                        if (runtime/1000>100)

        runtimeaccurate+=stopmstimer(timernumaccurate)

        timernumaccurate=startmstimer
                                                                                runtime=0
                                                                        endif
                                                while(runtimeaccurate/1000<(500*(i+1)))
                                                i +=1
                                        while(i<3)
                                                if (sum(RemLick)>2)
                                                runtimeaccurate=0
                                                LickCounter+=1
                                                Count8+=1
                                                Print " Correct Trial", time()
                                                Print "Phase 8, Trail Number", LickCounter
                                                Print "InterTrial Interval"
                                                Print "-----------------------------------------------
-----------"

                                                Closevalve(8)
                                                corr8+=1
                                                CorrectTrial()
                                                ctrlbackground period=60, start
                                                else
                                                        InCorrectTrial()
                                                        incorr8=incorr
                                                        ctrlbackground period=0, start
```

```
                                        endif

                            else
                                    InCorrectTrial()
                                    incorr8=incorr
                                    ctrlbackground period=0, start
                            endif
                    while (BeamBroken())
            endif
        break
Endswitch

if (LickCounter>19)
        GlobalStage+=1
        LickCounter=0
        BB_Counter=0
        Corr=0
        Incorr=0
elseif (Globalstage==8&&LickCounter>19)
        Ctrlbackground period=0, stop
        CloseValve(0)
        Print "End of the Pre-Training"
        Print "==================================="
KillBackground
endif

if (Count0>4)
        GlobalStage=1
        LickCounter=0
        BB_Counter=0
        Corr=0
endif

if (Count1>14)
        GlobalStage=2
        LickCounter=0
        BB_Counter=0
        Corr=0
        Print "Now Phase 2"
endif
End

//=========== Background Functions - Related to the Pretraining Procedure
============

Function CheckBeam()
        NVAR nBeam=nBeam
        SVAR WhatsUp=WhatsUp
        NVAR IsITI=IsITI
        NVAR ITICount=ITICount
```

```
	NVAR ITIinSeconds=ITIinSeconds

	if(IsITI)
		if (ITICount>ITIinSeconds)
			ctrlbackground period=60,stop
			ctrlbackground period=0,start
			IsITI=0
		else
			ITICount+=1
			WhatsUp="Inter trial interval - "+ num2str(ITICount)
		endif
	else
		if (BeamBroken()==1)
		nBeam+=1
		PreTraining()
		endif
	endif
	return 0
End

Function RewardIt()
variable timerNum, runTime

  EmptyTimers()
  timerNum=startMSTimer
  OpenWaterValve()
  do
	runTime+=stopMSTimer(timerNum)
	timerNum=startMSTimer
  while(runTime/1000<125)
  CloseWaterValve()
  runTime+=stopMSTimer(timerNum)
  EmptyTimers()
End

Function CorrectTrial()
variable kk, ww

If (BeamBroken())
	OpenwaterValve()
	EmptyTimers()
	kk=StartMSTimer
		do
		ww+=StopMSTimer(kk)
		kk=StartMSTimer
		while (ww/1000<125)
	CloseWaterValve()
	InterTrialIntervalPre()
endif
End
```

```
Function InCorrectTrial()
variable cc, kk, ww, dd
NVAR Incorr=Incorr

If (BeamBroken())
	EmptyTimers()
	cc=StartMSTimer
	kk=StartMSTimer
		if(CheckIsLicked()==1)
			EmptyTimers()
			Pretraining()
		endif
else
	do
		do
		ww+=StopMSTimer(kk)
		kk=StartMSTimer
		while (ww/1000<1500&&IsLicked()==0&&BeamBroken())
	dd+=StopMSTimer(cc)
	cc=StartMSTimer
	while (dd/1000<1500)
CloseValve(8)
Incorr+=1
Print " Incorrect Trial", Time()
Print "------------------------------------------------------------"
InterTrialIntervalPre()
endif
END

Function InterTrialIntervalPre()
variable cc, dd

EmptyTimers()
cc=StartMSTimer
	do
		dd+=StopMSTimer(cc)
		cc=StartMSTimer
	while (dd/1000<1500)
End

Function InterTrialIntervalPre1()
variable cc, dd

EmptyTimers()
cc=StartMSTimer
	do
		dd+=StopMSTimer(cc)
		cc=StartMSTimer
	while (dd/1000<1000)
```

```
END

Function CheckIsLicked()
variable cc, dd

EmptyTimers()
cc=StartMSTimer
	do
		dd+=StopMSTimer(cc)
		cc=StartMSTimer
	while (dd/1000<10)
	EmptyTimers()
If (IsLicked()==1&&BeamBroken())
	return 1
else
	return 0
endif
End

// ==================  Panel Specifications ==================

Window MainPanel() : Panel
	PauseUpdate; Silent 1		// building window...
	NewPanel /W=(4,57,294,388) as "Controls"
	ModifyPanel cbRGB=(60928,60928,60928), frameStyle=4, frameInset=4
	SetDrawLayer UserBack
	SetDrawEnv fname= "Times New Roman",fsize= 32,fstyle= 3,textxjust= 1,textyjust=
1
	DrawText 142,22,"PRE TRAINING"
	Button Resetbutton0,pos={156,118},size={65,26},proc=ButtonProc,title="RESET"
	Button Resetbutton0,fSize=14,fStyle=1
	Button
bStart,pos={82,118},size={59,26},proc=ButtonProc_2,title="START",fSize=14
	Button bStart,fStyle=1
	ValDisplay valdisp0,pos={130,46},size={69,15},title="Phase",fStyle=3
	ValDisplay valdisp0,limits={0,0,0},barmisc={0,1000},value= #"GlobalStage"
	ValDisplay valdisp1,pos={92,66},size={107,15},title="Trial Number",fStyle=3
	ValDisplay valdisp1,limits={0,0,0},barmisc={0,1000},value= #"LickCounter"
	ValDisplay valdisp2,pos={102,87},size={97,15},disable=2,title="Odor Valve"
	ValDisplay valdisp2,fStyle=19,limits={0,0,0},barmisc={0,1000},value= #"8"
	GroupBox group0,pos={51,156},size={191,154},title="STAGE
SELECTION",fSize=13
	GroupBox group0,frame=0,fStyle=3
	Button Stage1,pos={75,208},size={69,24},proc=ButtonProc_1,title="PHASE 2"
	Button Stage1,fStyle=3
	Button stage2,pos={73,239},size={71,24},proc=ButtonProc_3,title="PHASE 3"
	Button stage2,fStyle=3
	Button stage3,pos={72,270},size={71,23},proc=ButtonProc_4,title="PHASE 4"
	Button stage3,fStyle=3
	Button Stage4,pos={153,182},size={72,22},proc=ButtonProc_5,title="PHASE 5"
```

```
	Button Stage4,fStyle=3
	Button stage5,pos={156,209},size={70,24},proc=ButtonProc_6,title="PHASE 6"
	Button stage5,fStyle=3
	Button stage6,pos={156,241},size={70,23},proc=ButtonProc_7,title="PHASE 7"
	Button stage6,fStyle=3
	Button stage7,pos={156,270},size={69,23},proc=ButtonProc_8,title="PHASE 8"
	Button stage7,fStyle=3
	Button stage8,pos={74,180},size={69,23},proc=ButtonProc_9,title="PHASE 1"
	Button stage8,fStyle=3
EndMacro

//==================== Buttons - Main Panel ===========================

Function ButtonProc(ctrlName) : ButtonControl
	String ctrlName
	variable /g GlobalStage, LickCounter
	variable /g BB_Counter, count0, count1, count2, count3, count4, count5, count6,
count7, count8, bb0, bb1, bb2, bb3, bb4, bb5, bb6, bb7, bb8
	Variable /g Corr, Incorr, corr0, corr1, corr2, corr3, corr4, corr5, corr6, corr7, corr8,
incorr0, incorr1, incorr2, incorr3, incorr4, incorr5, incorr6, incorr7, incorr8

	KillBackGround
	GlobalStage=0
	LickCounter=0
	BB_Counter=0
	Corr=0
	Incorr=0
	count0=0
	count1=0
	count2=0
	count3=0
	count4=0
	count5=0
	count6=0
	count7=0
	count8=0
	bb0=0
	bb1=0
	bb2=0
	bb3=0
	bb4=0
	bb5=0
	bb6=0
	bb7=0
	bb8=0
	corr0=0
	corr1=0
	corr2=0
	corr3=0
	corr4=0
```

```
	corr5=0
	corr6=0
	corr7=0
	corr8=0
	incorr0=0
	incorr1=0
	incorr2=0
	incorr3=0
	incorr4=0
	incorr5=0
	incorr6=0
	incorr7=0
	incorr8=0
End


Function ButtonProc_2(ctrlName) : ButtonControl
	String ctrlName

	if (cmpstr(ctrlName, "bStart")==0)
		Button $ctrlName, title="STOP", rename=bstop
		execute "Start()"
	else
	Button $ctrlName, title="START", rename=bStart
	KillBackGround
	endif
End

Function ButtonProc_1(ctrlName) : ButtonControl
	String ctrlName
	NVAR GlobalStage=GlobalStage
	NVAR LickCounter=LickCounter

	GlobalStage=2
	LickCounter=0
	KillBackGround
End

Function ButtonProc_3(ctrlName) : ButtonControl
	String ctrlName
	NVAR GlobalStage=GlobalStage
	NVAR LickCounter=LickCounter

	GlobalStage=3
	LickCounter=0
	KillBackGround
End

Function ButtonProc_4(ctrlName) : ButtonControl
	String ctrlName
```

```
	NVAR GlobalStage=GlobalStage
	NVAR LickCounter=LickCounter

	GlobalStage=4
	LickCounter=0
	KillBackGround
End

Function ButtonProc_5(ctrlName) : ButtonControl
	String ctrlName
	NVAR GlobalStage=GlobalStage
	NVAR LickCounter=LickCounter

	GlobalStage=5
	LickCounter=0
	KillBackGround
End

Function ButtonProc_6(ctrlName) : ButtonControl
	String ctrlName
	NVAR GlobalStage=GlobalStage
	NVAR LickCounter=LickCounter

	GlobalStage=6
	LickCounter=0
	KillBackGround
End

Function ButtonProc_7(ctrlName) : ButtonControl
	String ctrlName
	NVAR GlobalStage=GlobalStage
	NVAR LickCounter=LickCounter

	GlobalStage=7
	LickCounter=0
	KillBackGround
End

Function ButtonProc_8(ctrlName) : ButtonControl
	String ctrlName
	NVAR GlobalStage=GlobalStage
	NVAR LickCounter=LickCounter

	GlobalStage=8
	LickCounter=0
	KillBackGround
End

Function ButtonProc_9(ctrlName) : ButtonControl
	String ctrlName
```

```
	NVAR GlobalStage=GlobalStage
	NVAR LickCounter=LickCounter

	GlobalStage=1
	LickCounter=0
	KillBackGround
End

Function CheckProc(ctrlName,checked) : CheckBoxControl
	String ctrlName
	Variable checked
	NVAR GlobalStage=GlobalStage
	NVAR LickCounter=LickCounter

	LickCounter=0
	GlobalStage=1

End

Function CheckProc_1(ctrlName,checked) : CheckBoxControl
	String ctrlName
	Variable checked
	NVAR GlobalStage=GlobalStage
	NVAR LickCounter=LickCounter

	GlobalStage=0
	LickCounter+=1
End

//==================== Valve Panel and Buttons Associated
====================

Window Panel0() : Panel
	PauseUpdate; Silent 1		// building window...
	NewPanel /W=(4,423,216,593) as "Initiate Valves"
	ModifyPanel frameStyle=4, frameInset=3
	Button Check,pos={13,131},size={50,20},proc=ButtonProc_Check,title="Check"
	CheckBox valve1,pos={10,6},size={51,14},proc=CheckProc_valve1,title="Valve1"
	CheckBox valve1,value= 1
	CheckBox valve2,pos={10,25},size={51,14},proc=CheckProc_valve2,title="Valve2"
	CheckBox valve2,value= 1
	CheckBox valve4,pos={10,43},size={51,14},proc=CheckProc_valve4,title="Valve4"
	CheckBox valve4,value= 1
	CheckBox valve8,pos={10,62},size={51,14},proc=CheckProc_valve8,title="Valve8"
	CheckBox valve8,value= 1
	CheckBox
valve16,pos={11,79},size={57,14},proc=CheckProc_valv16,title="Valve16"
	CheckBox valve16,value= 1
	CheckBox
valve32,pos={11,99},size={57,14},proc=CheckProc_valve32,title="Valve32"
```

```
	CheckBox valve32,value= 1
	CheckBox
valve64,pos={92,10},size={57,14},proc=CheckProc_valve64,title="Valve64"
	CheckBox valve64,value= 1
	CheckBox
valve128,pos={92,29},size={63,14},proc=CheckProc_valve128,title="Valve128"
	CheckBox valve128,value= 1
	Button Apply,pos={79,132},size={50,20},proc=ButtonProc_Apply,title="Apply"
	Button Init,pos={147,132},size={50,20},proc=ButtonProc_Init,title="Init"
	CheckBox FV,pos={92,47},size={31,14},proc=CheckProc_FV,title="FV",value= 1
	CheckBox RF,pos={93,64},size={32,14},proc=CheckProc_RF,title="RF",value= 1
	ValDisplay lick,pos={92,81},size={46,15},title="Lick"
	ValDisplay lick,limits={0,0,0},barmisc={0,1000},value= #"lick"
	ValDisplay Beam,pos={90,99},size={50,15},title="Beam"
	ValDisplay Beam,limits={0,0,0},barmisc={0,1000},value= #"beam"
	GroupBox group0,pos={9,123},size={192,36},frame=0
EndMacro


Function ButtonProc_Check(ctrlName) : ButtonControl
	String ctrlName
	variable /g lick, beam

	lick = IsLicked()
	beam = BeamBroken()
End

Function CheckProc_valve1(ctrlName,checked) : CheckBoxControl
	String ctrlName
	Variable checked
	NVAR valve1
	valve1=checked

End

Function CheckProc_valve2(ctrlName,checked) : CheckBoxControl
	String ctrlName
	Variable checked
	NVAR valve2
	valve2=checked

End

Function CheckProc_valve4(ctrlName,checked) : CheckBoxControl
	String ctrlName
	Variable checked
	NVAR valve4
	valve4=checked

End
```

```
Function CheckProc_valve8(ctrlName,checked) : CheckBoxControl
	String ctrlName
	Variable checked
	NVAR valve8
	valve8=checked

End

Function CheckProc_valv16(ctrlName,checked) : CheckBoxControl
	String ctrlName
	Variable checked
	NVAR valve16
	valve16=checked

End

Function CheckProc_valve32(ctrlName,checked) : CheckBoxControl
	String ctrlName
	Variable checked
	NVAR valve32
	valve32=checked

End

Function CheckProc_valve64(ctrlName,checked) : CheckBoxControl
	String ctrlName
	Variable checked
	NVAR valve64
	valve64=checked

End

Function CheckProc_valve128(ctrlName,checked) : CheckBoxControl
	String ctrlName
	Variable checked
	NVAR valve128
	valve128=checked

End


Function CheckProc_FV(ctrlName,checked) : CheckBoxControl
	String ctrlName
	Variable checked
	NVAR FV
	FV=checked

End
```

```
Function CheckProc_RF(ctrlName,checked) : CheckBoxControl
	String ctrlName
	Variable checked
	NVAR RF
	RF=checked

End

Function ButtonProc_Apply(ctrlName) : ButtonControl
	String ctrlName
	variable /g valve1,valve2,valve4,valve8,valve16,valve32,valve64,valve128, RF, FV

	OpenValve(128*valve128+64*valve64+32*valve32+16*valve16+8*valve8+4*valve4
+2*valve2+valve1)
	OperateWaterFinal(4*RF+2*FV)
End

Function ButtonProc_Init(ctrlName) : ButtonControl
	String ctrlName

	variable /g valve1, valve2, valve4,valve8,valve16,valve32,valve64,valve128, lick,
beam, FV, RF

	OutportB(772,0x9B)
	OutPortB(771,0x80)
End

//==================== Results Panel ===========

Window Results() : Panel
	PauseUpdate; Silent 1		// building window...
	NewPanel /W=(301,57,707,389)
	ModifyPanel cbRGB=(65534,65534,65534), frameStyle=4, frameInset=3
	SetDrawLayer UserBack
	DrawRect 224,149,288,172
	DrawRect 34,149,100,172
	DrawRect 98,149,165,172
	DrawRect 163,149,227,172
	DrawRect 287,149,353,172
	DrawRect 34,172,100,195
	DrawRect 98,172,164,195
	DrawRect 163,172,230,195
	DrawRect 227,172,290,195
	DrawRect 287,172,353,195
	DrawRect 98,191,165,214
	DrawRect 163,191,228,214
	DrawRect 227,191,289,214
	DrawRect 287,191,353,214
	DrawRect 34,213,100,236
	DrawRect 98,213,164,236
```

```
	DrawRect 163,213,229,236
	DrawRect 227,213,292,236
	DrawRect 287,213,353,236
	DrawRect 34,256,98,279
	DrawRect 98,279,126,302
	DrawRect 163,258,229,281
	DrawRect 227,257,293,280
	DrawRect 287,257,353,280
	DrawRect 34,278,100,301
	DrawRect 98,279,164,302
	DrawRect 163,278,229,301
	DrawRect 227,278,293,301
	DrawRect 287,278,353,301
	DrawRect 34,128,100,151
	DrawRect 98,128,164,151
	DrawRect 163,128,229,151
	DrawRect 227,128,293,151
	DrawRect 287,128,353,151
	DrawRect 34,235,100,258
	DrawRect 98,235,164,258
	DrawRect 163,235,229,258
	DrawRect 227,235,293,258
	DrawRect 287,235,353,258
	DrawRect 34,192,98,214
	DrawRect 97,301,163,324
	DrawRect 163,301,228,324
	DrawRect 227,301,289,324
	DrawRect 287,301,353,324
	DrawRect 34,301,98,324
	TitleBox title0,pos={102,8},size={217,24},title="SUMMARY OF THE 
RESULTS\\JC\\JC"
	TitleBox title0,fSize=14,frame=4,fStyle=3
	PopupMenu popup0,pos={22,58},size={65,21},title="No."
	PopupMenu popup0,mode=1,popvalue="1",value= #"\"1; 2; 3; 4; 5; 6; 7; 8; 9; 10; 
11; 12 ; 13; 14; 15; 16; 17; 18; 19; 20\""
	PopupMenu popup1,pos={232,58},size={152,21},title="Animal Line"
	PopupMenu popup1,mode=1,popvalue="WILDTYPE",value= #"\"WILDTYPE; 
NSTOM; MONC18; NR1\""
	PopupMenu popup2,pos={102,59},size={123,21},title="STRAIN"
	PopupMenu popup2,mode=1,popvalue="C57 BL6",value= #"\"C57 BL6\""
	GroupBox group0,pos={21,27},size={173,84},disable=1,title="FEATURES OF THE 
ANIMAL"
	GroupBox group0,fStyle=3
	GroupBox group1,pos={10,36},size={380,53},title="FEATUES OF THE 
ANIMAL",fStyle=3
	TabControl tab0,pos={33,129},size={64,20},tabLabel(0)="PHASE 0",value= 0
	TabControl tab0_1,pos={33,152},size={64,19},tabLabel(0)="PHASE 1",value= 0
	TabControl tab0_2,pos={33,173},size={64,17},tabLabel(0)="PHASE 2",value= 0
	TabControl tab0_3,pos={33,193},size={63,22},tabLabel(0)="PHASE 3",value= 0
	TabControl tab0_4,pos={33,213},size={62,20},tabLabel(0)="PHASE 4",value= 0
```

```
	TabControl tab0_5,pos={33,236},size={63,20},tabLabel(0)="PHASE 5",value= 0
	TabControl tab0_6,pos={33,259},size={62,20},tabLabel(0)="PHASE 6",value= 0
	TabControl tab0_7,pos={33,280},size={62,21},tabLabel(0)="PHASE 7",value= 0
	TabControl tab1,pos={105,110},size={51,20},fSize=8,tabLabel(0)="TRIALS",value=
0
	TabControl tab2,pos={165,110},size={60,19},fSize=8,tabLabel(0)="ATTEMPTS"
	TabControl tab2,value= 0
	TabControl tab3,pos={229,110},size={56,19},fSize=8,tabLabel(0)="CORRECT"
	TabControl tab3,value= 0
	TabControl tab4,pos={288,111},size={65,18},fSize=8,tabLabel(0)="INCORRECT"
	TabControl tab4,value= 0
	ValDisplay valdisp0,pos={120,133},size={35,15},frame=0,fStyle=3
	ValDisplay valdisp0,limits={0,0,0},barmisc={0,1000},value= #"Count0"
	TabControl tab0_8,pos={33,302},size={64,22},tabLabel(0)="PHASE 8",value= 0
	ValDisplay valdisp1,pos={120,155},size={35,15},frame=0,fStyle=3
	ValDisplay valdisp1,limits={0,0,0},barmisc={0,1000},value= #"Count1"
	ValDisplay valdisp2,pos={119,175},size={32,15},frame=0,fStyle=3
	ValDisplay valdisp2,limits={0,0,0},barmisc={0,1000},value= #"Count2"
	ValDisplay valdisp3,pos={119,194},size={25,15},frame=0,fStyle=3
	ValDisplay valdisp3,limits={0,0,0},barmisc={0,1000},value= #"Count3"
	ValDisplay valdisp4,pos={119,217},size={40,15},frame=0,fStyle=3
	ValDisplay valdisp4,limits={0,0,0},barmisc={0,1000},value= #"Count4"
	ValDisplay valdisp5,pos={119,239},size={24,15},frame=0,fStyle=3
	ValDisplay valdisp5,limits={0,0,0},barmisc={0,1000},value= #"Count5"
	ValDisplay valdisp6,pos={120,283},size={29,15},frame=0,fStyle=3
	ValDisplay valdisp6,limits={0,0,0},barmisc={0,1000},value= #"Count7"
	ValDisplay valdisp7,pos={120,262},size={23,15},frame=0,fStyle=3
	ValDisplay valdisp7,limits={0,0,0},barmisc={0,1000},value= #"Count6"
	ValDisplay valdisp8,pos={119,306},size={24,15},frame=0,fStyle=3
	ValDisplay valdisp8,limits={0,0,0},barmisc={0,1000},value= #"Count8"
	ValDisplay valdisp9,pos={182,134},size={42,15},frame=0,fStyle=3
	ValDisplay valdisp9,limits={0,0,0},barmisc={0,1000},value= #"bb0"
	ValDisplay valdisp10,pos={183,155},size={41,15},frame=0,fStyle=3
	ValDisplay valdisp10,limits={0,0,0},barmisc={0,1000},value= #"bb1"
	ValDisplay valdisp11,pos={245,132},size={41,15},frame=0,fStyle=3
	ValDisplay valdisp11,limits={0,0,0},barmisc={0,1000},value= #"corr0"
	ValDisplay valdisp12,pos={311,132},size={23,15},frame=0,fStyle=3
	ValDisplay valdisp12,limits={0,0,0},barmisc={0,1000},value= #"Incorr0"
	ValDisplay valdisp13,pos={244,154},size={35,15},frame=0,fStyle=3
	ValDisplay valdisp13,limits={0,0,0},barmisc={0,1000},value= #"corr1"
	ValDisplay valdisp14,pos={309,154},size={23,15},frame=0,fStyle=3
	ValDisplay valdisp14,limits={0,0,0},barmisc={0,1000},value= #"Incorr1"
	ValDisplay valdisp15,pos={182,174},size={42,15},frame=0,fStyle=3
	ValDisplay valdisp15,limits={0,0,0},barmisc={0,1000},value= #"bb2"
	ValDisplay valdisp16,pos={182,194},size={23,15},frame=0,fStyle=3
	ValDisplay valdisp16,limits={0,0,0},barmisc={0,1000},value= #"bb3"
	ValDisplay valdisp17,pos={183,216},size={22,15},frame=0,fStyle=3
	ValDisplay valdisp17,limits={0,0,0},barmisc={0,1000},value= #"bb4"
	ValDisplay valdisp18,pos={184,239},size={25,15},frame=0,fStyle=3
	ValDisplay valdisp18,limits={0,0,0},barmisc={0,1000},value= #"bb5"
```

```
	ValDisplay valdisp19,pos={185,262},size={20,15},frame=0,fStyle=3
	ValDisplay valdisp19,limits={0,0,0},barmisc={0,1000},value= #"bb6"
	ValDisplay valdisp20,pos={186,283},size={22,15},frame=0,fStyle=3
	ValDisplay valdisp20,limits={0,0,0},barmisc={0,1000},value= #"bb7"
	ValDisplay valdisp21,pos={187,306},size={20,15},frame=0,fStyle=3
	ValDisplay valdisp21,limits={0,0,0},barmisc={0,1000},value= #"bb8"
	ValDisplay valdisp22,pos={244,175},size={35,15},frame=0,fStyle=3
	ValDisplay valdisp22,limits={0,0,0},barmisc={0,1000},value= #"corr2"
	ValDisplay valdisp23,pos={244,195},size={26,15},frame=0,fStyle=3
	ValDisplay valdisp23,limits={0,0,0},barmisc={0,1000},value= #"corr3"
	ValDisplay valdisp24,pos={244,217},size={20,15},frame=0,fStyle=3
	ValDisplay valdisp24,limits={0,0,0},barmisc={0,1000},value= #"corr4"
	ValDisplay valdisp25,pos={244,239},size={20,15},frame=0,fStyle=3
	ValDisplay valdisp25,limits={0,0,0},barmisc={0,1000},value= #"corr5"
	ValDisplay valdisp25_1,pos={245,259},size={20,15},frame=0,fStyle=3
	ValDisplay valdisp25_1,limits={0,0,0},barmisc={0,1000},value= #"corr6"
	ValDisplay valdisp25_2,pos={244,281},size={20,15},frame=0,fStyle=3
	ValDisplay valdisp25_2,limits={0,0,0},barmisc={0,1000},value= #"corr7"
	ValDisplay valdisp25_3,pos={246,305},size={20,15},frame=0,fStyle=3
	ValDisplay valdisp25_3,limits={0,0,0},barmisc={0,1000},value= #"corr8"
	ValDisplay valdisp14_1,pos={308,174},size={28,15},frame=0,fStyle=3
	ValDisplay valdisp14_1,limits={0,0,0},barmisc={0,1000},value= #"Incorr2"
	ValDisplay valdisp14_2,pos={308,196},size={28,15},frame=0,fStyle=3
	ValDisplay valdisp14_2,limits={0,0,0},barmisc={0,1000},value= #"Incorr3"
	ValDisplay valdisp14_3,pos={307,218},size={28,15},frame=0,fStyle=3
	ValDisplay valdisp14_3,limits={0,0,0},barmisc={0,1000},value= #"Incorr4"
	ValDisplay valdisp14_4,pos={307,240},size={28,15},frame=0,fStyle=3
	ValDisplay valdisp14_4,limits={0,0,0},barmisc={0,1000},value= #"Incorr5"
	ValDisplay valdisp14_5,pos={306,263},size={28,15},frame=0,fStyle=3
	ValDisplay valdisp14_5,limits={0,0,0},barmisc={0,1000},value= #"Incorr6"
	ValDisplay valdisp14_6,pos={307,284},size={28,15},frame=0,fStyle=3
	ValDisplay valdisp14_6,limits={0,0,0},barmisc={0,1000},value= #"Incorr7"
	ValDisplay valdisp14_7,pos={307,306},size={28,15},frame=0,fStyle=3
	ValDisplay valdisp14_7,limits={0,0,0},barmisc={0,1000},value= #"Incorr8"
EndMacro

//==================== Test Macros =====================

Macro WaterReleaseTest()

Variable i, ReinforcementTime=90

Print "Test Beggin"
Print "The Reinforcement Time is", ReinforcementTime
i=1
	do
		print "", i
		Rewardit()
		i+=1
	while (i<11)
```

```
Print "Test Finnished"
Endmacro


//====================== Welcome Layout ================================


Window Welcome() : Layout
	PauseUpdate; Silent 1		// building window...
	Layout/C=1/B=(39168,0,0)/W=(5.25,42.5,643.5,493.25) as "Pre-Traning Program"
	TextBox/C/N=text0/T={17,72,116,144,180,216,252,288,324,360}/A=LB/X=0.00/Y=5
3.83
	AppendText "\\F'Times New Roman'\\Z14\\Zr125   \\Z16The Pre-Traning Program
was developed in the University Heidelberg."
	AppendText "   The operating software was developed by Daniel Nunes (PhD
student) who had an introductory"
	AppendText "   help in Igor programing by Prof. Dr. med. Thomas Kuner and Dr.
Andreas Schaefer. The running procedure was "
	AppendText "   designed by Dr. Nixon Abraham and Daniel Nunes (PhD student).\r
"
	AppendText "   These Pre-Training program is composed by eight phases which
have different levels of dificculty."
	AppendText "   In short, in the phase 0 (5 trials) the animal just needs to break the
beam to get the water reward (it learns where "
	AppendText "   is the water resouce). In the phase 1 (15 trials) the animal needs to
lick to get the reward."
	AppendText "   In phase 2 and the next ones there are 20 trials per phase (like in a
block of the Training program) and it is introduced "
	AppendText "   the odour valve (in this software the valve 8 is the operating one). In
phase 3 it is introduced the Final Valve"
	AppendText "   as well as the incorrect trials and the Inter Trial Interval increases.
The later phases have the same design as"
	AppendText "   phase 3, just the requirement gradually change in order to fit to the
Training program. In this sense, the "
	AppendText "   phase 8 is approximatelly equal to the Training program. In the end
the animal is able to perform the Training program.\r  "
	AppendText "         (Please close this window and click \"Repalce\" or \"Save\" to
procced with the program\")."
	ModifyLayout width(text0)=455.25,height(text0)=221.25
	ModifyLayout mag=1, units=1
	SetWindow kwTopWin,hook(WM_Tint)=TintedBackground#WM_TintWindowHook
	SetWindow kwTopWin,userdata(WM_Tint)=  "ProgBack:Tint0,;"
EndMacro
```
